# Integrative Analysis of DNA Methylation Identified 12 Signature Genes Specific to Metastatic ccRCC

**DOI:** 10.3389/fonc.2020.556018

**Published:** 2020-10-08

**Authors:** Siwei Qian, Si Sun, Lei Zhang, Shengwei Tian, Kai Xu, Guangyuan Zhang, Ming Chen

**Affiliations:** ^1^Department of Urology, Zhongda Hospital, Southeast University, Nanjing, China; ^2^Institute of Urology, School of Medicine, Southeast University, Nanjing, China; ^3^Department of Urology, Changzhou No. 2 People's Hospital, Changzhou, China

**Keywords:** clear cell renal cell carcinoma, data integration, DNA methylation, gene expresssion, ROC (receiver operating characteristic curve)

## Abstract

**Background:** Abnormal epigenetic alterations can contribute to the development of human malignancies. Identification of these alterations for early screening and prognosis of clear cell renal cell carcinoma (ccRCC) has been a highly sought-after goal. Bioinformatic analysis of DNA methylation data provides broad prospects for discovery of epigenetic biomarkers. However, there is short of exploration of methylation-driven genes of ccRCC.

**Methods:** Gene expression data and DNA methylation data in metastatic ccRCC were sourced from the Gene Expression Omnibus (GEO) database. Differentially methylated genes (DMGs) at 5′-C-phosphate-G- 3′ (CpG) sites and differentially expressed genes (DEGs) were screened and the overlapping genes in DMGs and DEGs were then subject to gene set enrichment analysis. Next, the weighted gene co-expression network analysis (WGCNA) was used to search hub DMGs associated with ccRCC. Cox regression and ROC analyses were performed to screen potential biomarkers and develop a prognostic model based on the screened hub genes.

**Results:** Three hundred and fourteen overlapping DMGs were obtained from two independent GEO datasets. The turquoise module contained 79 hub DMGs, which represent the most significant module screened by WGCNA. Furthermore, a total of 12 hub genes (*CETN3, DCAF7, GPX4, HNRNPA0, NUP54, SERPINB1, STARD5, TRIM52, C4orf3, C12orf51*, and *C17orf65*) were identified in the TCGA database by multivariate Cox regression analyses. All the 12 genes were then used to generate the model for diagnosis and prognosis of ccRCC. ROC analysis showed that these genes exhibited good diagnostic efficiency for metastatic and non-metastatic ccRCC. Furthermore, the prognostic model with the 12 methylation-driven genes demonstrated a good prediction of 5-year survival rates for ccRCC patients.

**Conclusion:** Integrative analysis of DNA methylation data identified 12 signature genes, which could be used as epigenetic biomarkers for prognosis of metastatic ccRCC. This prognostic model has a good prediction of 5-year survival for ccRCC patients.

## Background

Clear cell renal carcinoma (ccRCC) is the major type of renal tumor in the human urinary system (1). Many patients with ccRCC have distant metastasis to lymphoid or other organs (2). The 5-year survival rate was lower in patients with distant metastatic ccRCC than with non-metastatic ccRCC (3). Over the past decade, the therapeutic strategies for advanced ccRCC have evolved rapidly from a non-specific immune approach to targeted therapy against vascular endothelial growth factor (VEGF), and recently to novel immunotherapy. Multiple agents like immune checkpoint inhibitors (ICIs) and tyrosine kinase inhibitors (TKIs) show promising results in the clinical trials and have been approved as the second-line even the first-line treatment for advanced ccRCC (4–7). Moreover, targeted combination therapies have been explored as the first-line treatment in pioneer trials (8).

However, to enhance the efficacy and prognosis always underscores a need for new therapeutic targets and drug combinations (9). An increasing number of studies prove that epigenetic alterations such as DNA methylation and histone modifications are associated with cancer progression and occurrence. Therefore, they could be exploited as biomarkers for cancer diagnosis and prognosis (10–14). However, epigenetic biomarkers underlying metastatic ccRCC remain to be elucidated. Epigenetic biomarkers have been testified in certain malignancies and allowed their potential use in differentiation between metastatic and non- metastatic ccRCC. In this study, we profiled methylation patterns of metastatic and primary non-metastatic ccRCC and identified potential biomarkers associated with the cancer progression. We measured the gene methylation levels at CpG sites throughout the genome and found that a subset of genes presented with aberrant methylation and expression in metastatic ccRCC when compared with primary non-metastatic ccRCC. We further created the criteria to screen hub DMGs and defined the pathological stages of ccRCC accordingly. Finally, we applied the survival analysis to evaluate the hub DMGs as potential biomarkers for the prognosis of ccRCC patients.

## Methods

### Data Collection

The gene methylation datasets (GSE113501 and GSE105260) were downloaded from the GEO database. GSE113501 and GSE105260 were generated on the platform GPL13534 (Illumina HumanMethylation450 BeadChip). In total, 54 metastatic tumor samples and 96 non-metastatic tumor samples were included in the study. GSE105261 was used for screening of DEGs in non-metastatic and metastatic ccRCC. TCGA-KIRC dataset was used for analysis of correlation between gene expression and methylation levels. All ccRCC-related clinical data in this study were obtained from the GEO and the Cancer Genome Atlas (TCGA) database.

### Differential Methylation Analysis

First, the missing methylation values among all samples were removed. To identify differentially methylated CpGs, two-sample independent *t*-test was applied to compare primary non-metastatic ccRCC and metastatic ccRCC data. Bonferroni procedure, together with transformed β values, was used to adjust crude *p*-values in multiple comparisons. The β value was calculated by the formula β = *M*/(*M* + *U* + *a*), where *M* and *U* represent the methylated and unmethylated signal intensities, respectively. *a* is usually set as 100 to stabilize β values when *M* and *U* are too small (15). β average were used for evaluation of global and regional CpG methylations. The delta β value was calculated to evaluate the methylation difference between metastatic and non-metastatic ccRCC. *M* and β values were used to identify the DMGs and intergenic CpG sites. The adjusted *p* < 0.05 and delta β values in 4/10 quantile were used as the cutoff criteria to screen candidate hub genes or intergenic CpG sites. R packages “limma” and “lumi” were used for differential analysis and conversion between β and *M* values, respectively (16–22). The DMGs were classified into six types according to the methylation patterns.

## Gene Set Enrichment Analysis

Gene ontology (GO) and Kyoto Encyclopedia of Genes and Genomes (KEGG) pathway enrichment analyses were implemented by DAVID online tools (23, 24). *p* < 0.05 was considered statistically significant. The GO term enrichment analysis included three categories: biological process (BP), molecular function (MF), and cellular component (CC). R package “Venn Diagram” was used to illustrate the overlapping genes in DMGs and DEGs.

### Correlation Between DNA Methylation and Gene Expression

We examined the correlation between DNA methylation and gene expression based on the data from the TCGA database. Spearman correlation coefficients were used to evaluate the expression value and the methylation level at all genes CpG sites. The correlation was regarded significant if the absolute value of correlation coefficient was >0.3 and FDR was <0.05. Only the overlapping genes in DMGs and DEGs were evaluated for their biological functions by gene regulation analysis.

### Weighted Co-expression Network Construction Analysis (WCGNA)

R package “WGCNA” was used for constructing co-expression networks among genes across microarray samples (25). To begin with, the power of β (soft thresholding) was defined to ensure a standard scale-free network. An adjacency matrix was then constructed with the values of adjacencies between each pair of node genes in the network and their Pearson's correlation coefficients. The adjacency matrix was used to calculate the topological overlap matrix (TOM) and the corresponding dissimilarity (1-TOM). Finally, based on TOM-based dissimilarity measures, the dendrogram was constructed by average linkage hierarchical clustering. Highly similar modules were clustered and then merged with a height cut-off of 0.25.

### Identification of Modules Corresponding to Clinical Traits

By WGCNA, the modules most relevant to the clinical phenotypes of interest could be identified. Here, we differentiated clinical phenotypes by pathological stages. As a principal component of the gene expression matrix, module eigengene (ME) was used to identify modules corresponding to clinically significant traits. Gene significance was evaluated by log *p-*value of each gene in the linear regression between gene expression and pathological progression. The module significance was used to evaluate the correlation between module expression patterns and clinical traits (age, gender, survival time, and disease stage, etc.). In general, a module was regarded significantly correlating with certain clinical traits if its absolute module significance ranked the highest among others.

### Identification of DMGs Associated With Overall Survival Prognosis and Validation of Potential Epigenetic Biomarkers by Receiver Operating Characteristic (ROC) Analysis

To identify module genes associated with prognosis of ccRCC patients, we analyzed gene methylation data in the turquoise module from the Cancer Genome Atlas Kidney Renal Clear Cell Carcinoma (TCGA-KIRC) data collection. Univariate Cox regression was used to evaluate the prognostic power of 79 individual probes at CpG sites. Furthermore, multivariate Cox regression analysis was performed to evaluate whether all genes corresponding to individual CpG sites could be used as independent prognostic factors for patient's survival. *p* < 0.05 indicates statistical significance. ROC analysis was used to validate the predictive accuracy of potential epigenetic biomarkers, which were remarkably methylated in the multivariable Cox regression model. The area under the curve (AUC) was calculated to measure the quality of the classifier with R package “pROC”.

### Development and Evaluation of Prognostic Model

The genes with significant *p*-values by multivariate Cox regression were selected to develop a prognostic model. The risk score was calculated with regression coefficients multiplied by methylation levels. The median risk score was set as the cutoff value, by which 307 ccRCC patients from the TCGA database were divided into the high-risk group and the low-risk group. Clinical traits were used as an independent variable and the overall survival (OS) as the dependent variable to calculate the hazard ratio (HR). ROC curve was plotted with the R package “survivalROC” to confirm the predictive power of the 12-gene prognostic model and assess the probability of 1, 3, and 5-year OS for ccRCC patients.

## Results

### Identification of DMGs in Metastatic ccRCC

To identify potential DMG biomarkers for prognosis of ccRCC, we performed a DNA methylation profiling on 150 tumor tissues. All the relevant clinical data were collected from the GEO datasets: GSE113501 (including 28 metastatic ccRCC tissues and 87 non-metastatic ccRCC tissues) and GSE105260 (including 26 metastatic ccRCC tissues and 9 non-metastatic ccRCC tissues). In total, 237 CpG sites in GSE113501 and 462 CpG sites in GSE105260 were identified to have differential methylations (Supplementary Tables 1, 2), which corresponded to 198 and 355 genes, respectively (Supplementary Tables 3, 4). GO analysis showed that 552 DMGs were linked to the biological pathways that involved in transcriptional regulation of cancers, cell cycle and metabolism, such as bladder cancer (Supplementary Table 5).

**Figure 1:**
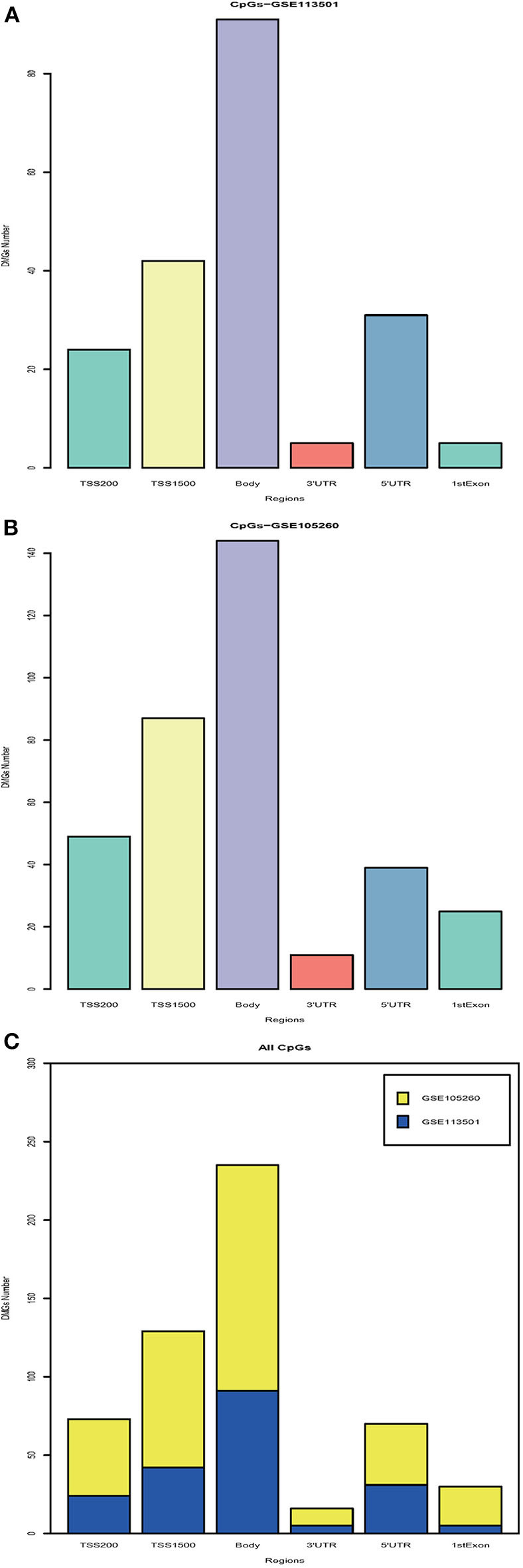
Integration analysis of DMGs. **(A)** Barplot for different region DMGs in GSE113501. The *y*-axis indicates the number of DMGs and the *x*-axis shows the positions around CpG islands: TSS1500, TSS200, 5′UTR, 1stExon, body, and 3′UTR. TSS1500 refers to 200–1,500 bases upstream of the transcriptional start site (TSS). TSS200 means 0–200 bases upstream of TSS. 5′UTR stands for the 5′untranslated region located between the TSS and the ATG start site. 1stExon is short for the first exon of the gene. Body is the region between ATG start site and stop codon. 3′UTR is short for 3′untranslated region that is between stop codon and poly-A tail. **(B)** Barplot for different region DMGs in GSE105260. **(C)** Barplot for all DMGs based on different regions. The blue module stands for DMGs in GSE113501. The yellow module represents for DMGs in GSE105260.

With the DNA methylation annotation file (GPL13534), we examined CpG methylation in different regions: TSS1500, TSS200, 5′UTR, 1stExon, body, and 3′UTR. Over 80 methylated CpG sites located in the body region (Figure 1A, GSE113501 and Figure 1B, GSE105260). Totally, more than 200 differentially methylated CpG sites located in the TSS1500 and body region (Figure 1C). By contrast, <25 methylated CpG sites appeared at 3′UTR region.

### DMGs Involved in Transcriptional Regulation

**Figure 2:**
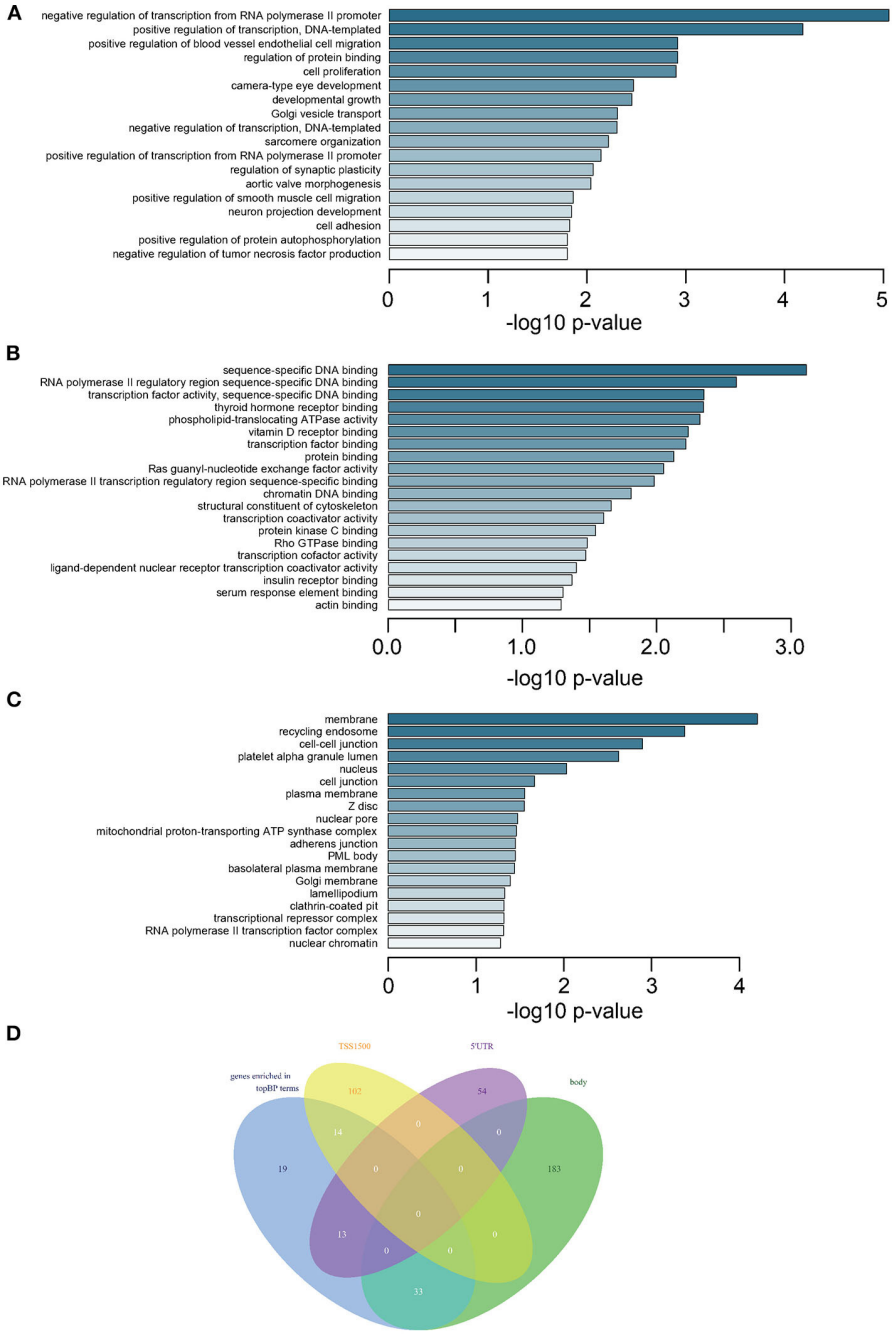
Enrichment analysis of DMGs and intersection of methylated CpGs in different regions. **(A)** Subontology of biological process (BP). **(B)** Subontology of molecular function (MF). **(C)** Subontology of cellular components (CC). **(A–C)** The *x*-axis indicates the log *p*-value. The *y*-axis is the enriched terms. **(D)** Venn plot for DMGs under BP terms based on different CpG methylation regions. The numbers on the diagram represent the DMG numbers in a specific region or multiple regions. The regions is as indicated.

GO analysis revealed that most DMGs were annotated to biological process (BP) and molecular function (MF) terms indicating transcriptional regulation: positive or negative regulation of transcription for various BPs (Figure 2A and Supplementary Table 6) and transcription factor bindings (Figure 2B). In addition, the ontology of cell component (CC) pointed to key cellular structures responsible for cell-cell interaction and cell activities (Figure 2C). Venn diagram showed that genes representing top BP terms were mostly methylated in the regions of body, 5′UTR, and TSS1500 (Figure 2D).

### Overlapping Genes in DMGs and DEGs

**Figure 3:**
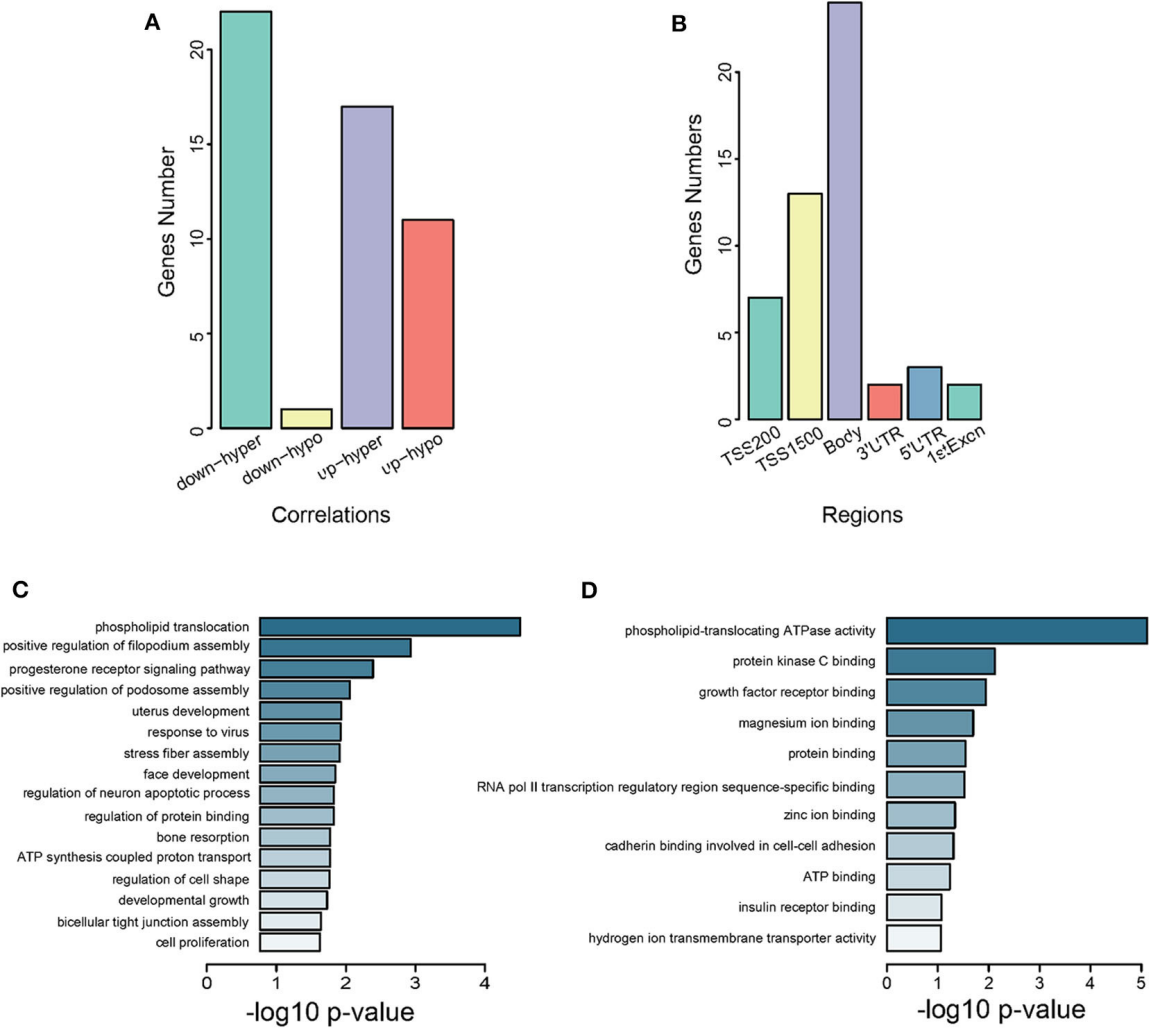
Characterization of intersected DMGs. **(A)** Grouping of intersected genes by methylation levels. Down-hyper represents downregulated hyper-methylated genes. Up-hypo represents upregulated hypo-methylated genes. Up-hyper represents upregulated hyper-methylated genes. Down-hypo represents downregulated hypo-methylated genes. The *y*-axis is the number of genes and the *x*-axis indicates different groups. **(B)** Grouping of intersected genes by CpG methylation regions. The *y*-axis is the number of intersected genes. The *x*-axis labels different gene regions: TSS1500, TSS200, 5′UTR, 1stExon, body, and 3′UTR. **(C)** Subontology of biological process (BP) for intersected genes. **(D)** Subontology of molecular function (MF) for intersected genes. **(C,D)** The *x*-axis indicates the log *p*-value. The *y*-axis is the enriched terms.

We identified 51 genes overlapping between DEGs from GSE105261 and DMGs from GSE105260 (Supplementary Figure 1 and Supplementary Table 7), which were classified into four types according to their methylation and expression levels, namely downregulated hypermethylated or hypomethylated, and upregulated hypermethylated or hypomethylated. Noticeably, downregulated hypomethylated genes accounted for a much lower proportion (Figure 3A). The CpG methylation of these genes mainly took place in the body and TSS1500 regions (Figure 3B). GO analysis also revealed that they were related to transcriptional factor assembly and receptor-mediated signaling (Figure 3C), and key protein bindings (Figure 3D). Moreover, 10 genes were screened against the TCGA-KIRC database, which showed a significant correlation between methylation level and expression level in ccRCC patients (Supplementary Figure 2).

### Identification of DMGs Associated With Metastatic ccRCC Throughout the Genome

**Figure 4:**
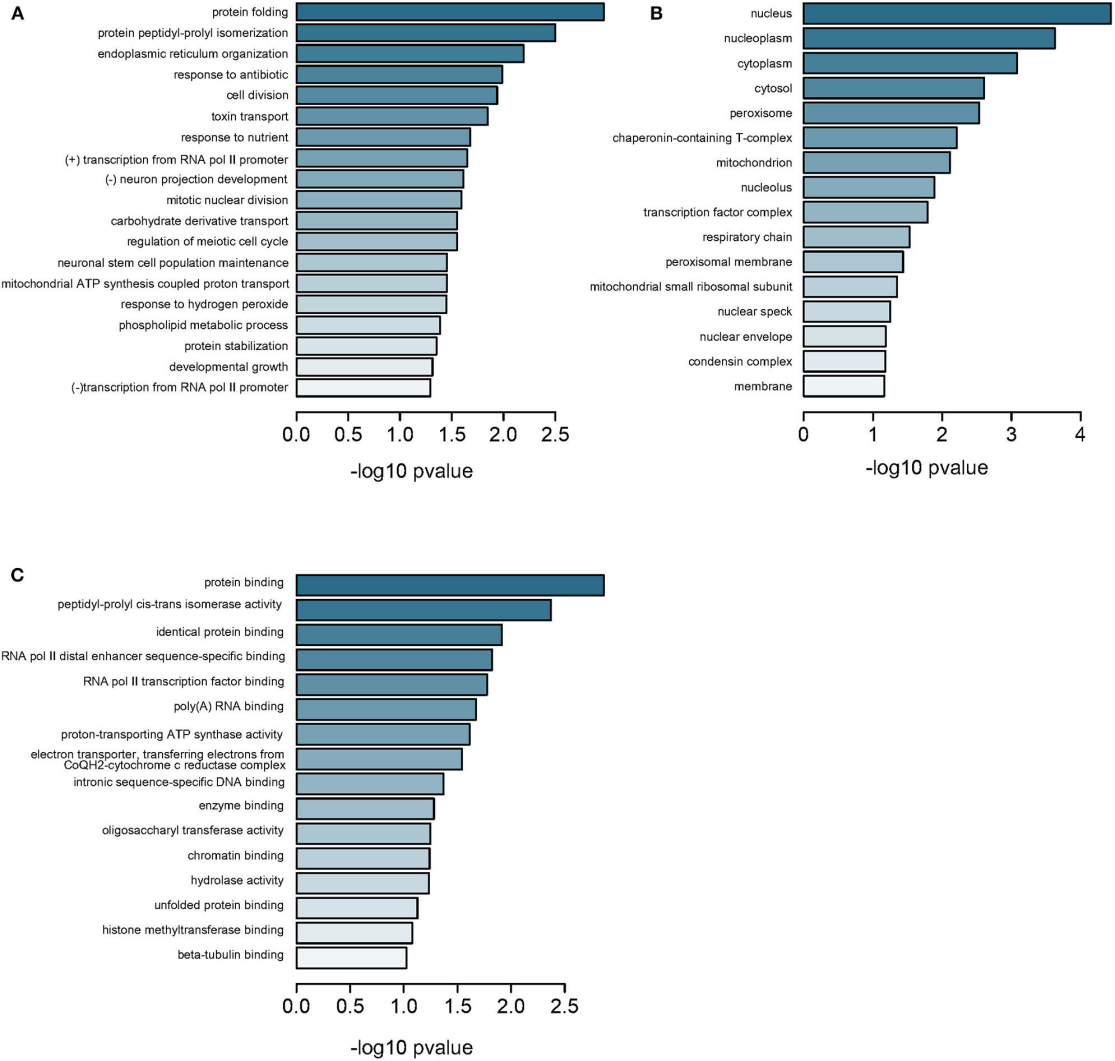
Enrichment analysis of DMGs in all regions. **(A)** Subontology of biological process (BP). **(B)** Subontology of cellular components (CC). **(C)** Subontology of molecular function (MF). **(A–C)** The *x*-axis indicates the log *p*-value. The *y*-axis is the enriched terms.

Further, we measured all CpG methylations throughout the genome and identified 6,300 genes from GSE113501 (Supplementary Table 8) and 799 genes from GSE105260 (Supplementary Table 9), respectively. Three hundred and fourteen genes were overlapped between the two subsets (Supplementary Figure 3). As previously, these genes were annotated to BPs relating to protein folding and transportation (Figure 4A and Supplementary Table 10) and CCs to components in nucleus (Figure 4B), as well as MFs to key element bindings (Figure 4C). When comparing the 314 DMGs with 3,002 DEGs filtered from GSE105261, we found 44 genes were overlapped (Supplementary Figures 4 and Supplementary Table 11). Most of them were downregulated-hypermethylated genes (Supplementary Figure 5), indicating their domination in the progress of ccRCC. The TCGA-KIRC dataset and Spearman test were applied for correlation analysis, which identified top 10 genes with significant correlation between methylation and expression (Supplementary Table 12 and Supplementary Figure 6).

### Identification of Hub DMGs

**Figure 5:**
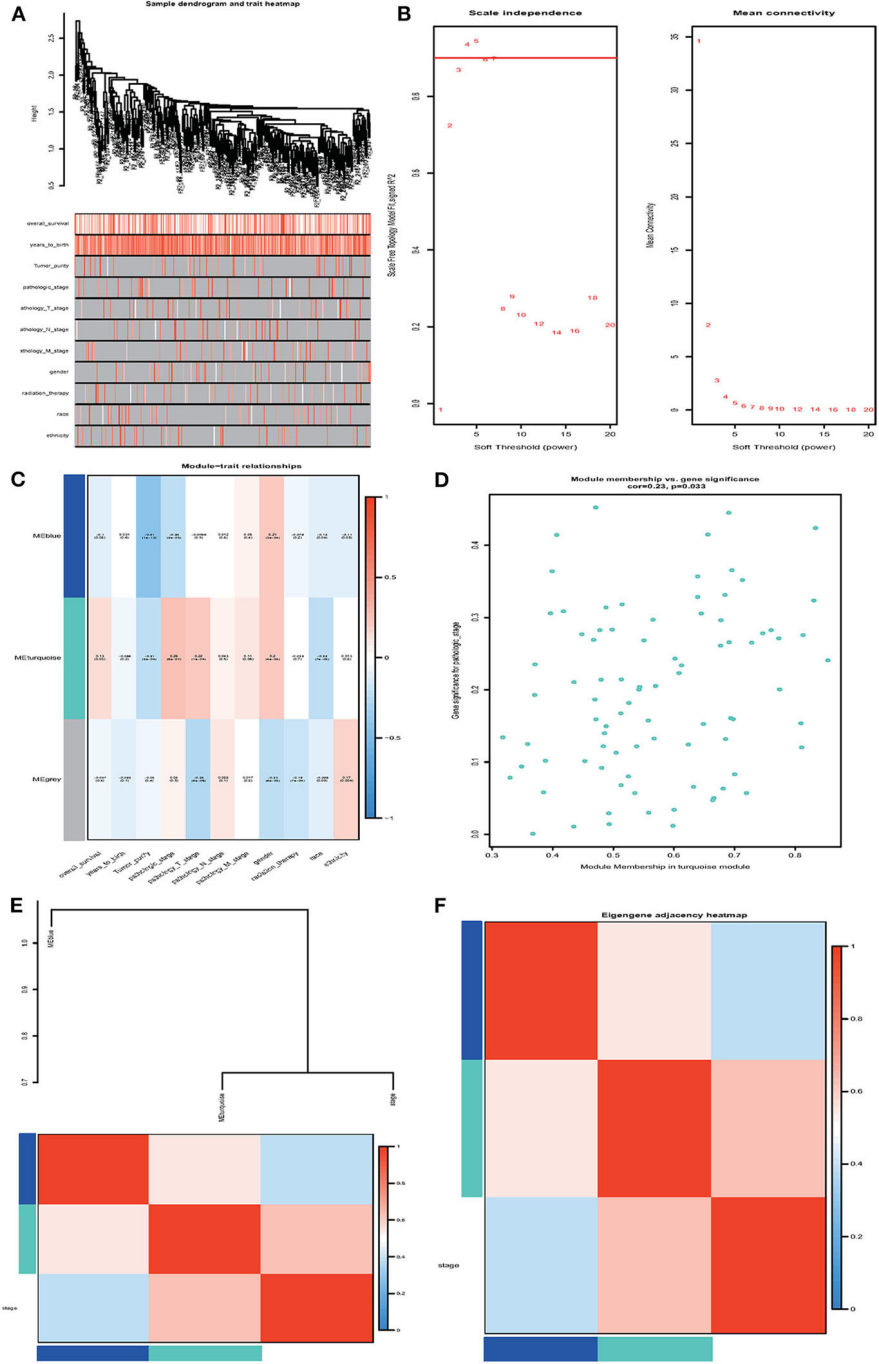
Weighted gene co-expression network analysis (WGCNA). **(A)** Sample dendrogram and trait heatmap. **(B)** Analysis of the scale-free fit index and the mean connectivity for various soft-thresholding powers (β). **(C)** Heatmap of correlation between module eigengenes and clinical information. **(D)** Module membership in the turquoise module. **(E,F)** The correlation between each module and clinical characteristic pathological stage was demonstrated based on Eigen gene. Blue represents a negative correlation, while red represents a positive correlation.

Next, we performed the WGCNA analysis to identify potential molecular modules that could characterize the pathological stages of ccRCC. The WGCNA network was constructed with 308 DMGs screened out by methylation level changes in all CpG island regions. The DMGs irrelevant to ccRCC clinical features were removed (Supplementary Table 13). The dendrogram and traits of all samples were illustrated (Figure 5A) and the soft threshold power was set as five to ensure a scale-free network (Figure 5B). Three WCGNA modules were identified as blue, turquoise, and gray (Figure 5C). The blue module showed to be negatively correlated with pathological stages while turquoise module was positively. The turquoise module harboring 79 genes was selected for further analysis as it was the most relevant to clinical traits (Supplementary Tables 14 and Data Sheet 2). The correlation coefficients with *p*-values, as well as heatmap of each module were calculated, respectively (Figures 5D–F) and the 79 relevant genes were considered as hub DMGs.

### Identification of 12 Signature DMGs

**Figure 6:**
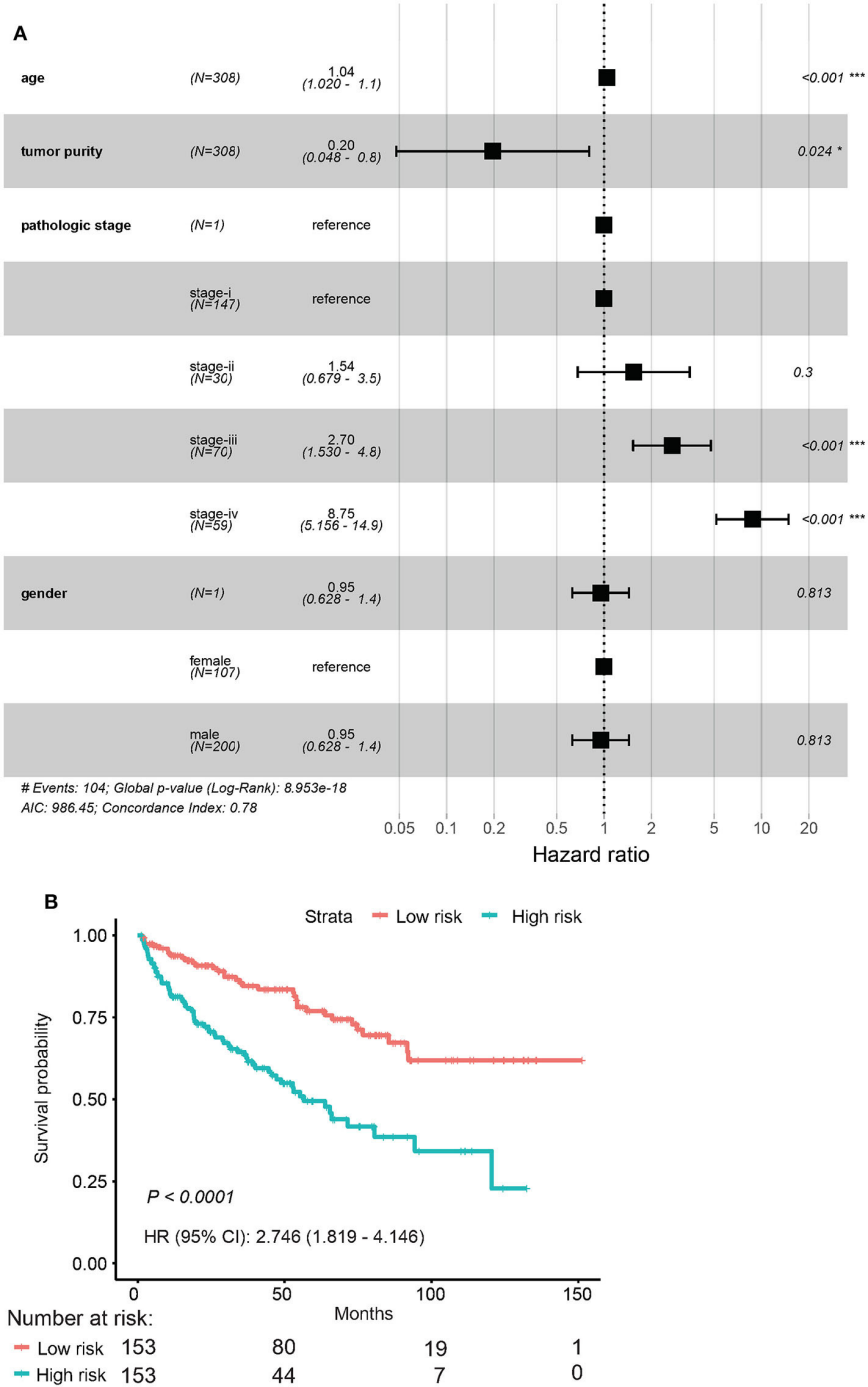
Evaluation of the 12-gene model. **(A)** Evaluation of hazard ratios by clinical traits. **(B)** Time–dependent ROC curve for high risk and low risk.

Among the 79 hub DMGs, 43 CpG sites were further identified as potential DNA methylation biomarkers for prediction of overall survival of ccRCC patients by univariate Cox regression analysis. The genes such as *TGDS, TCTE3, TOPBP1, SNX14*, and *PHIP* showed their expression were strongly associated with the overall survival in ccRCC patients (Supplementary Data Sheet 1). By contrast, the multivariate cox analysis revealed 12 DMGs (*CETN3, DCAF7, GPX4, HNRNPA0, NUP54, SERPINB1, STARD5, TRIM52, C4orf3, C12orf51, C17orf65*, and *C21orf45*) significantly affected overall survival of ccRCC patients (Table 1). The module' risk score was significant (Figure 6A) and survival analysis exhibited hypermethylation signature of the 12 genes significantly correlated with poor prognosis (*p* < 0.001) (Figure 6B). Moreover, we examined methylation levels of the 12 genes in normal kidney tissues. The results showed that there was no significant difference between normal kidney tissues and non-metastatic ccRCC kidney tissues, but for metastatic renal cancer. Above all, all the analyses suggested that the multi-gene methylation signature may be associated with ccRCC metastasis (Supplementary Data Sheet 3).

**Table 1:** The results of multivariate cox regression analysis.

**Variables**	**Overall Survival**	
	**HR (95% CI)**	***P***
Clinical parameters		
Gender	1.577 (0.62598–1.19)	0.369
Age, year	1.044 (1.01861–1.046)	2.73e−06^***^
Race	9.396 (0.03915–2.076)	0.215
Tumor purity	0.1471 (0.27283–2.591)	0.763
Pathologic stage	0.1484 (0.24456–0.491)	2.53e−09^***^
**Analysis parameters**		
CETN3	7.82E+35 (5.835e+07–1.049e+64)	0.012380^*^
DCAF7	1.42E+150 (9.363e+04–2.149e+295)	0.042654^*^
GPX4	6.37E−40 (1.197e−67–3.385e−12)	0.005592^**^
HNRNPA0	2.89E−38 (2.574e−65–3.255e−11)	0.006532^**^
NUP54	2.18E+57 (1.668e+15–2.838e+99)	0.007622^**^
SERPINB1	2.36E+08 (5.448e+02–1.022e+14)	0.003598^**^
STARD5	1.32E+16 (1.019e+00–1.704e+32)	0.049886^*^
TRIM52	4.91E+45 (4.245e+13–5.684e+77)	0.005222^**^
C4orf3	2.85E+68 (2.846e+11–2.856e+125)	0.018582^*^
C12orf51	1.60E−39 (6.178e−73–4.167e−06)	0.022873^*^
C17orf65(ASB16)	4.40E−59 (3.745e−89–5.165e−29)	0.000143^***^
C21orf45	2.47E−91 (2.804e−181–2.182e−01)	0.048339^*^

* *p < 0.05*,

** *p < 0.01*,

*** *p < 0.001*.

**Figure 7:**
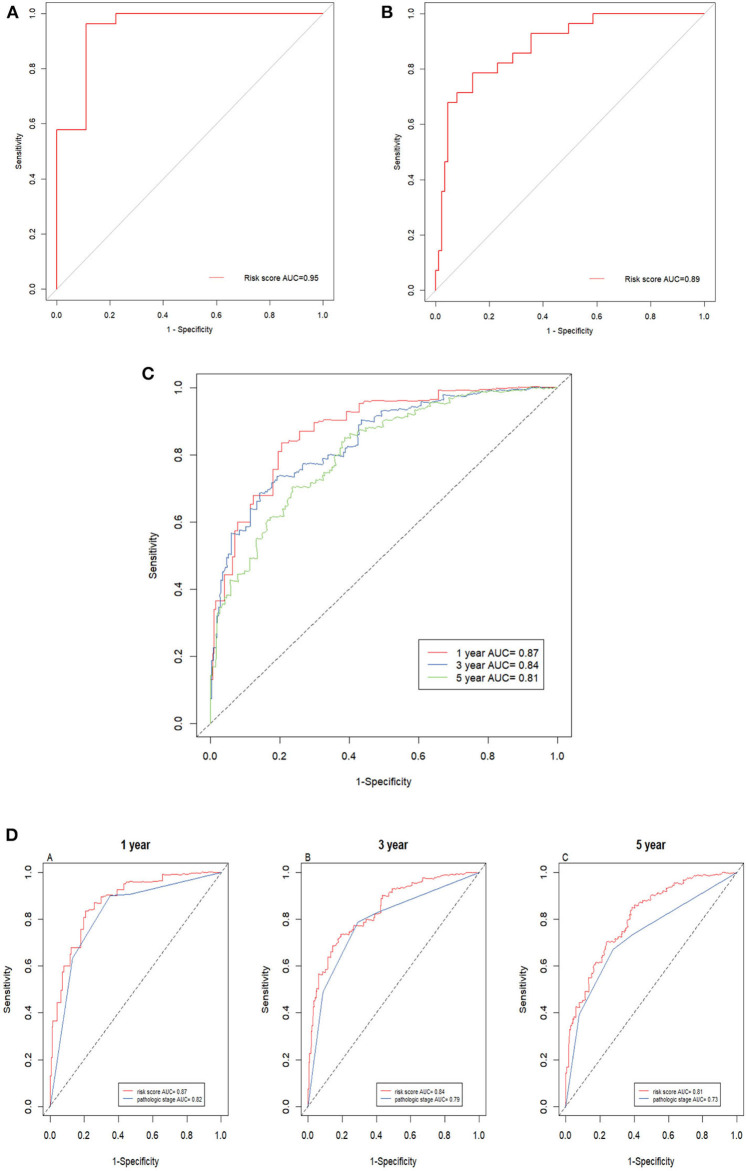
ROC curves of 12-gene signature. **(A)** ROC curves of the 12-gene signature in GSE105260 (AUC = 0.95). **(B)** ROC curves of the 12-gene signature in GSE105260 (AUC = 0.89). **(C)** Time–dependent ROC curve for OS, the AUC was assessed at 1, 3, and 5 year (AUC = 0.87, 0.84, and 0.81). **(D)** Time-dependent ROC curve analysis evaluates the accuracy of the 12-gene model.

Next, the ROC AUC was used to evaluate predictive powers of the prognostic models constructed with single signature gene or multiple signature genes. The ROC curves indicated that *C17orf65, HNRNPA0*, and S*TARD5* exhibited better diagnostic accuracy for differentiating metastatic and non-metastatic ccRCC cases in GSE105260 as the AUC value was >70% (Supplementary Data Sheet 4). By contrast, *C12orf51, C17orf65, SERPINBI*, and *TRIM52* demonstrated better diagnostic accuracy for differentiation of cases in GSE113501 (Supplementary Data Sheet 5). The ROC curve validated that the 12 signature genes as a whole had better diagnostic accuracy than individual signature genes in GSE105260 (Figure 7A). Also, the 12-gene signature could effectively diagnose the patients with metastatic ccRCC case in GSE113501 (Figure 7B).

The prediction powers for 1-, 3-, and 5-year overall survival were 0.87, 0.84, and 0.81, respectively, indicating that the prognostic model with the 12 signature genes had high sensitivity and specificity (Figure 7C). The values for predicting the 3- and 5-year survival were lower than that for 1-year, suggesting that the model was more accurate for short-term prediction than for long-term prediction. Additionally, as shown in Figure 7D, the 12-gene prognostic model was more powerful in the prediction than other model based on pathological stages.

## Discussion

ccRCC is the most common type of renal carcinoma in the world. Although the treatment strategies for ccRCC have been improved during the last decades, the management of metastatic ccRCC still remain challenging due to lack of eligible molecular targets. Therefore, discovery of specific markers for early diagnosis is essential for control of ccRCC progress. DNA methylation is an important driver of many of the distinct stages of cancer, to study the molecular mechanisms underlying the pathogenesis of ccRCC may be helpful for its diagnosis and treatment. To the best of our knowledge, there is no applicable markers for differentiating clinical stages of ccRCC. In the present study, we used the clinical data from GSE113501 and GSE105260 to profile genes associated with non-metastatic and metastatic ccRCC. Through WGCNA and ROC analysis, 12 genes were finally identified in the signature module associated with the clinical traits of ccRCC.

We first screened 237 differentially methylated CpG sites from the GSE113501 dataset and 462 CpG sites from the GSE105260 dataset, which were mapped to 552 genes. We examined the methylation patterns in specific regions: TSS1500, TSS200, 5′UTR, 1stExon, body, and 3′UTR, of which TSS1500 and body region showed remarkable methylation changes. GO analysis showed these DMGs were annotated to transcription landscape. In fact, DNA methylation and transcription factors (TFs) binding represent two components of the regulatory architecture and their interplay would underlie the clinical outcome of cancer development (26–33). By the WGCNA analysis, we screened 314 intersected DMGs between metastatic and non-metastatic ccRCC, which were associated with the biological processes and functions that underlined cancer metastasis and invasion. For example, dysregulation of cell cycle was also reported to associate with cancer progress and prognosis in the number of previous studies (33–38). In view of MFs, these DMGs involved in protein binding, peptidyl-prolyl cis-trans isomerase activity, identical protein binding and poly (A) RNA binding, etc. For example, the etiology of early-onset CRC was linked to ploy (A) RNA binding endorsed by a set of hub genes (39) and the metastasis of breast cancer resulted from LSD1 demethylation (40).

From GSE105261 and GSE105260, we further screened 44 intersected genes between DEGs (in GSE105261) and DMGs (in GSE105260). We classified these intersected genes into two categories: negatively and positively correlated between their methylation and expression levels. The majority of intersected genes fell into the negative correlation category, including downregulated-hypermethylated genes and upregulated-hypomethylated genes. Among these intersected genes, the methylation changes in 10 genes were the most significantly associated to the phenotypes of ccRCC when searching against the TCGA-KIRC dataset. However, some of them have been found to associate with other cancers via diversified mechanisms, for example, nuclear LDHA promoted HPV-induced cervical cancer development using a mechanism of H3K79 hypermethylation (41–44).

Finally, univariate and multivariate Cox regression analysis identified 43 CpG sites and 12 DMGs (*CETN3, DCAF7, GPX4, HNRNPA0, NUP54, SERPINB1, STARD5, TRIM52, C4orf3, C12orf51, C17orf65*, and *C21orf45*) that were highly correlated to patients' survival. Indeed, some genes are reported to contribute to the occurrence and progress of cancers. For example, GPX4 negatively correlated with prognosis of pan-cancer patients as the low methylation level at the upstream site leads to its higher expression in cancer tissues (45). On the contrary, hypermethylation of *SERPINB1* promoter resulted in enhanced neutrophil elastase (NE) activity, which was associated with poor outcome in prostate cancer (46). Other genes like *HNRNPA0, DCAF7*, and *TRIM52* functioned in diverse ways including abnormal changes in alternative splicing or activation of canonical signal pathways in relation with cancer progression (47–50).

Until recently, two research groups also screened signature genes for prognosis in ccRCC patients based on TCGA database. One group constructed a prognostic model with 7 signature genes (*IFI30, WNT5A, IRF9, AGER, PLAUR, TEK*, and *BID*), which were all immunity-related genes (IAGs) (50). The other group built the model with 5 lncRNAs (51). While in present study, we screened the signature genes from the standpoint of methylation alteration. Our model with 12 DMGs has the AUC value of 0.81 for 5-year survival prediction compared to the IGA-based model (0.751) and the lncRNA-based model (0.91). Our model could distinguish metastatic and non-metastatic pathologies, which could accompany traditional pathological tissue typing and provide more precise diagnosis and prognosis of ccRCC patients. Meanwhile, it is necessary to validate our model with more clinical data and the mechanisms by these signature DMGs in ccRCC.

## Conclusion

Taken together, we performed an integrative DNA methylation profiling and constructed a 12-gene model that signed in metastatic ccRCC. The model could be used for prognosis of patients with ccRCC and diagnosis of non-metastatic ccRCC and metastatic ccRCC. Aberrant methylation of the 12 genes would leave patients with ccRCC at higher risk of a poor prognosis.

## Data Availability Statement

The datasets generated for this study can be found in online repositories. The names of the repository/repositories and accession number(s) can be found in the article/Supplementary Material.

## Author Contributions

MC and GZ designed the study and were major contributors in editing the manuscript. SQ analyzed and interpreted the data and was major contributor in writing the manuscript. SS, LZ, ST, and KX performed analysis and contributed to writing the manuscript. All authors read and approved the final manuscript.

## Conflict of Interest

The authors declare that the research was conducted in the absence of any commercial or financial relationships that could be construed as a potential conflict of interest.
