# Supplementary figures and images for: Integrative Analysis of DNA Methylation Identified 12 Signature Genes Specific to Metastatic ccRCC
Source: Front Oncol. 2020 Oct 8;10:556018. doi: 10.3389/fonc.2020.556018 (PMC7578385; doi:10.3389/fonc.2020.556018)

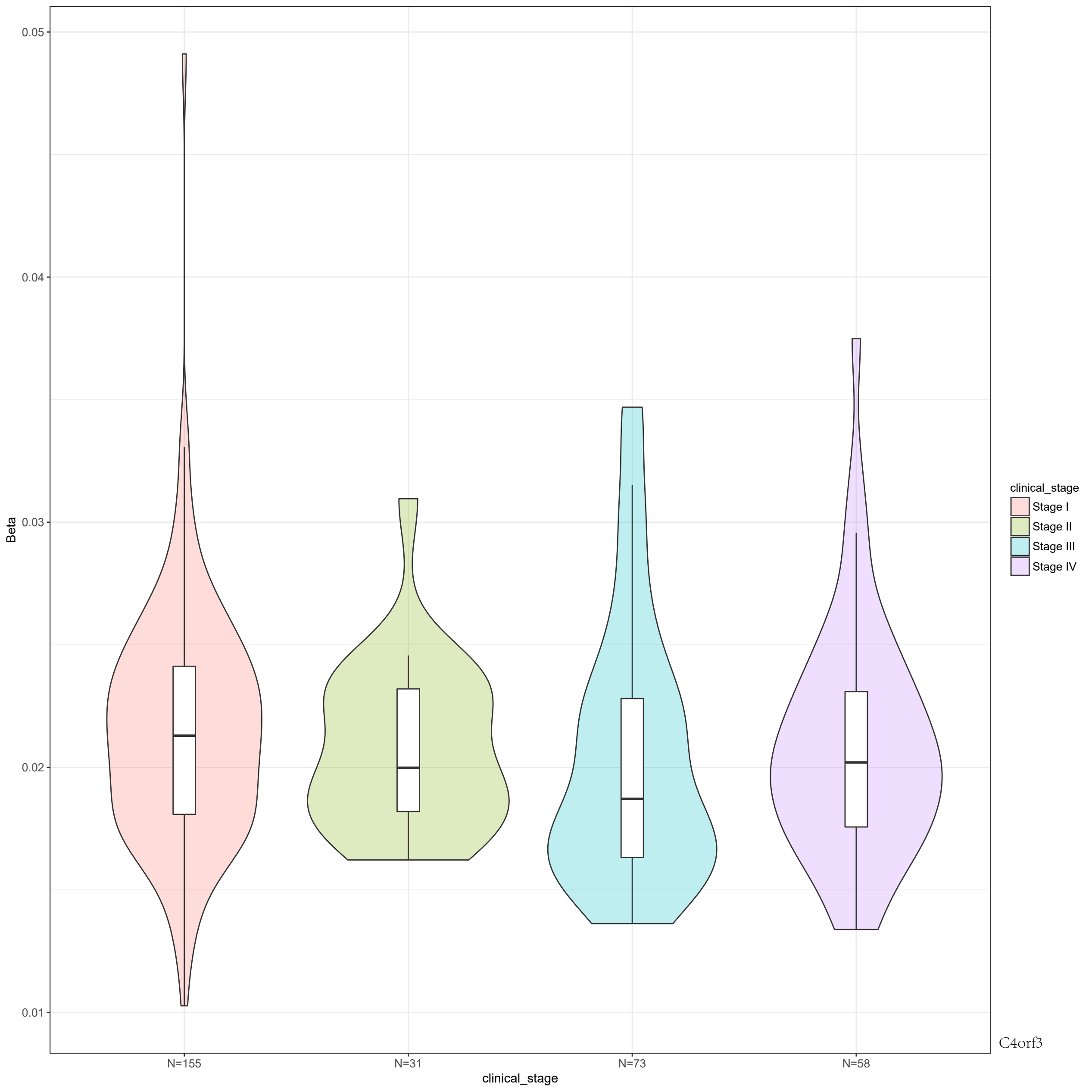

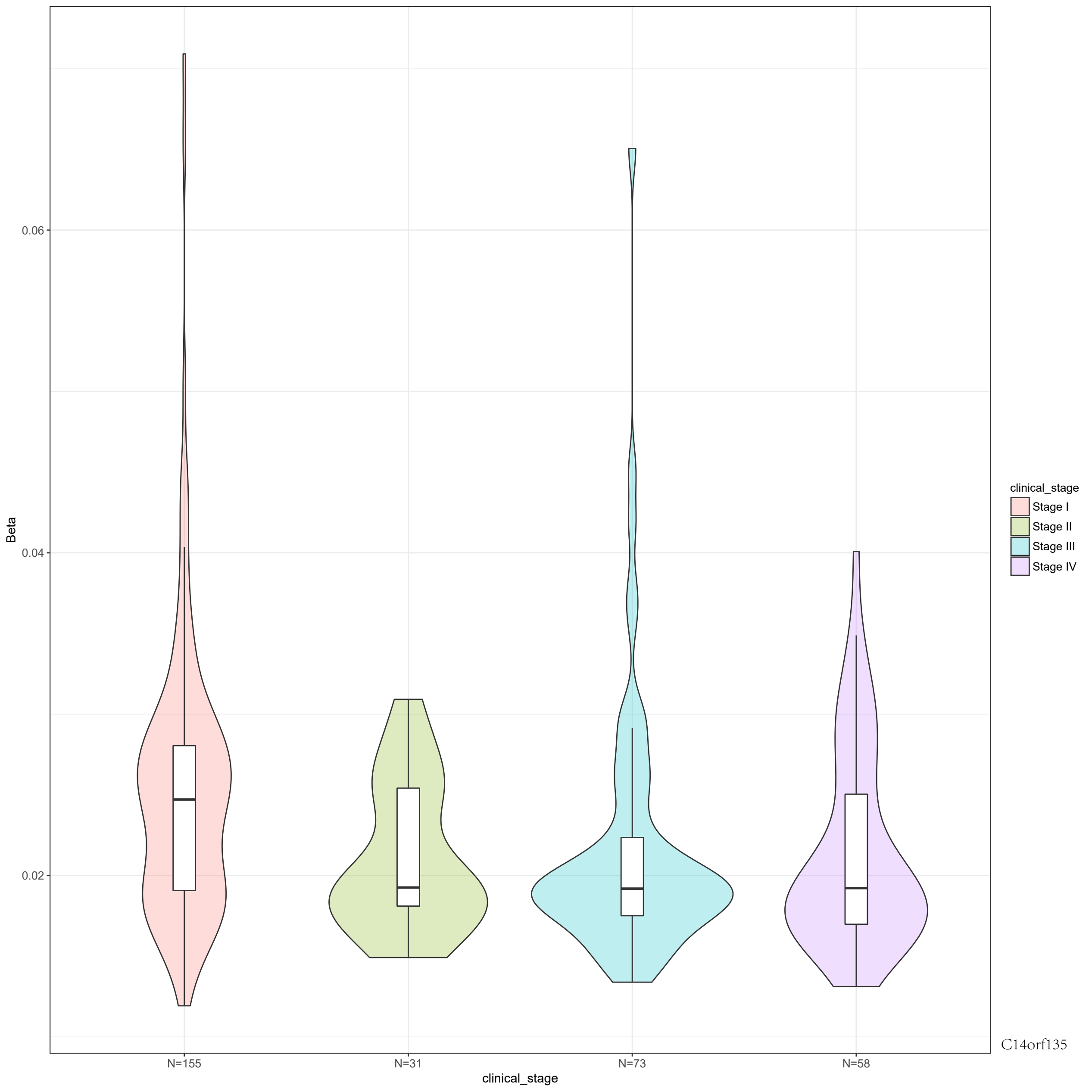

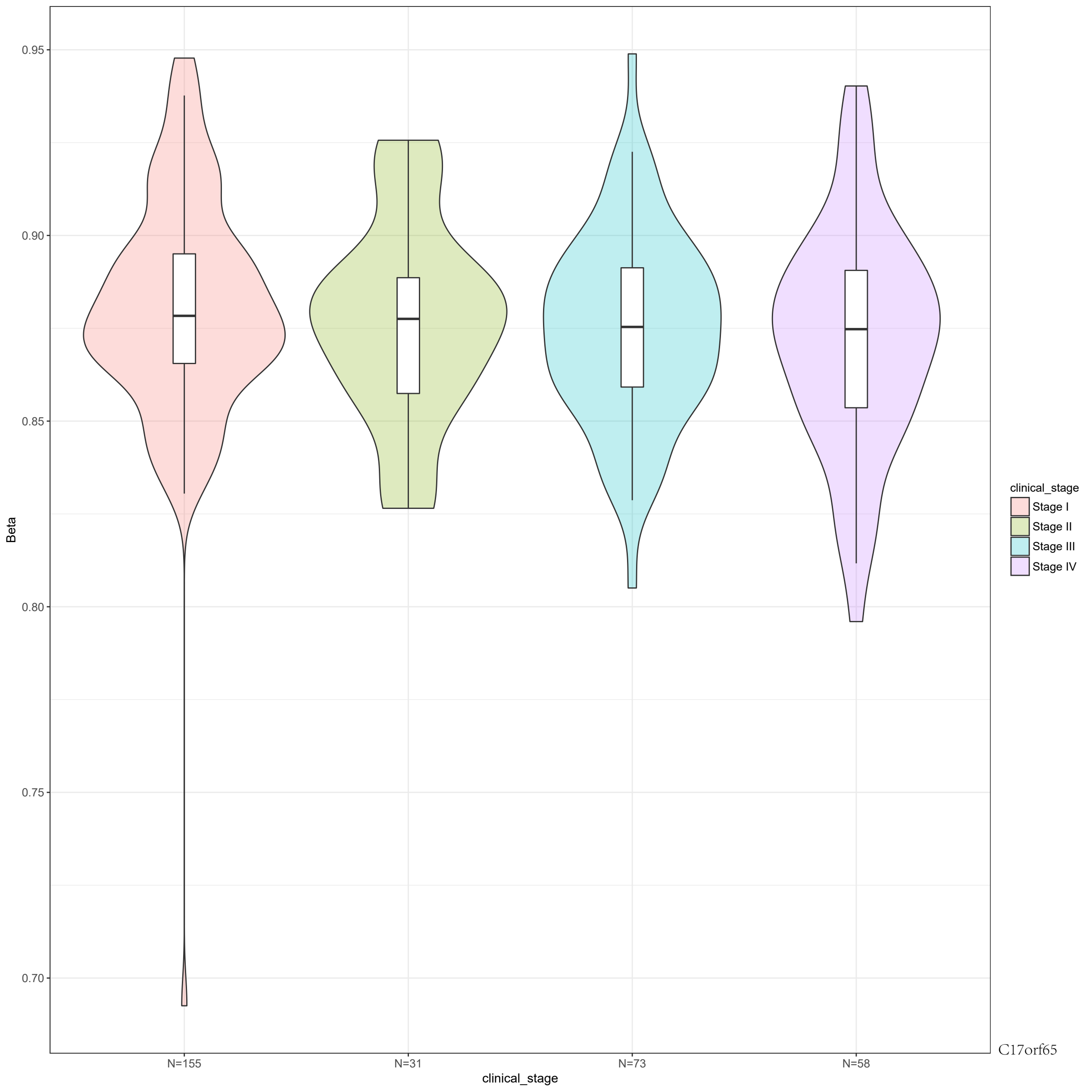

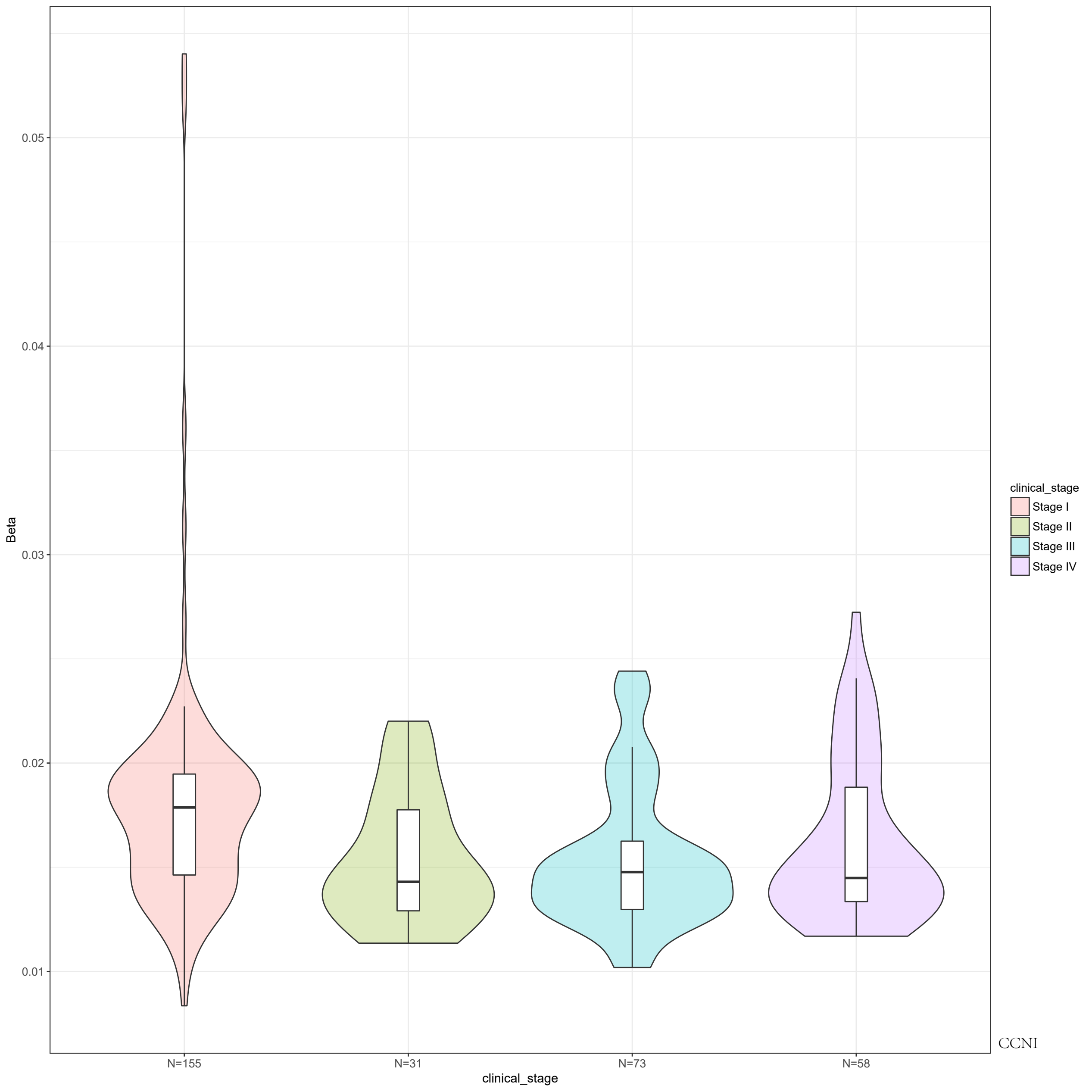

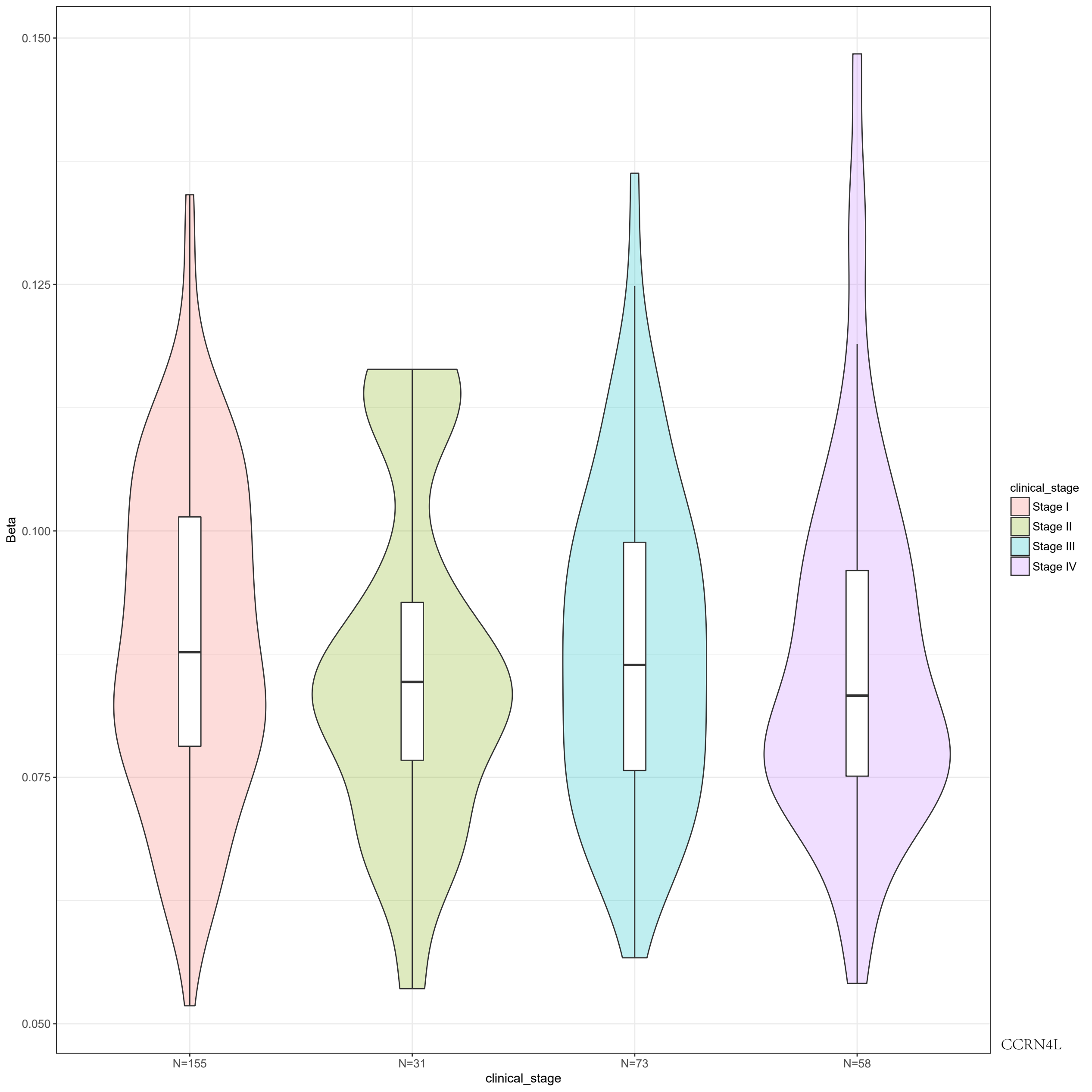

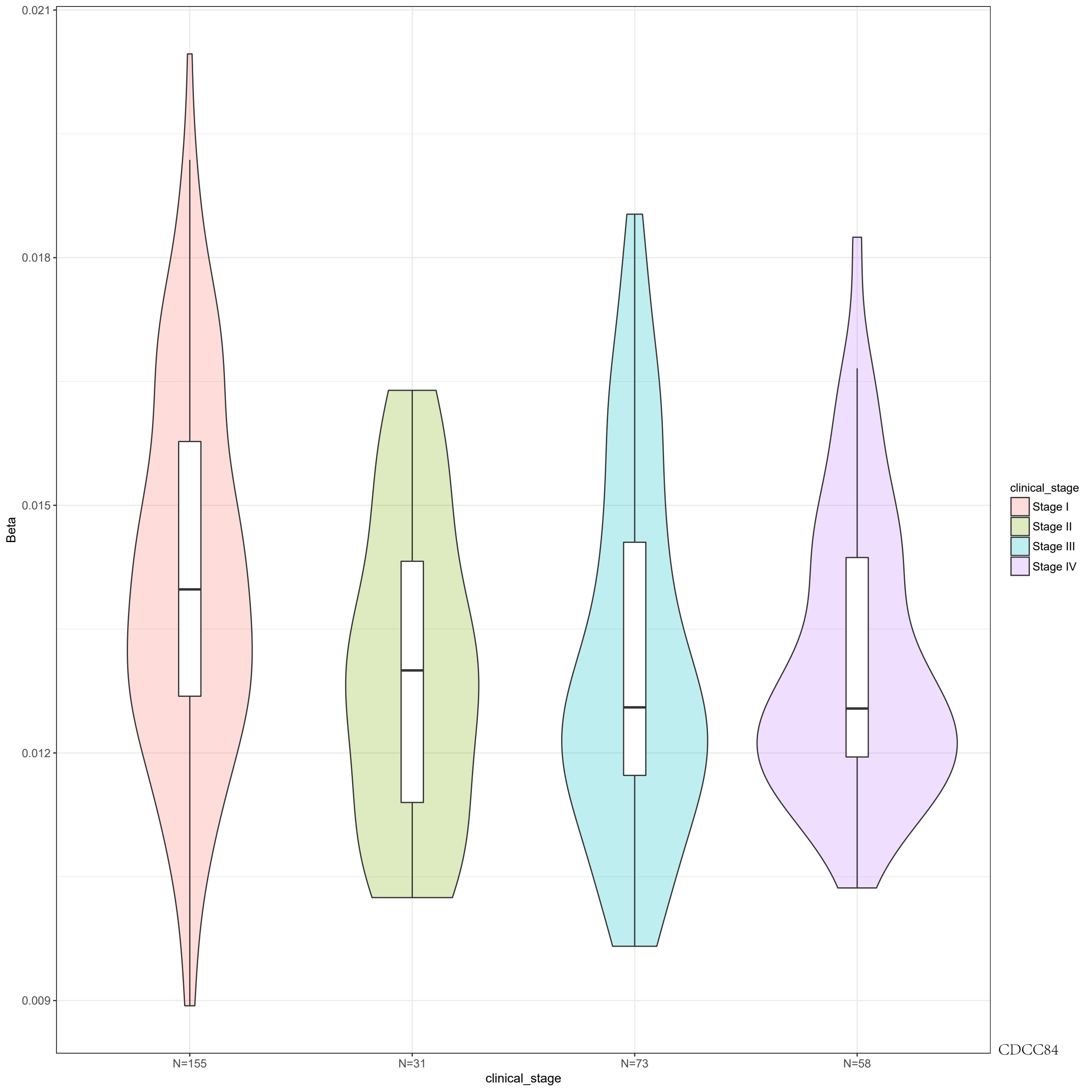

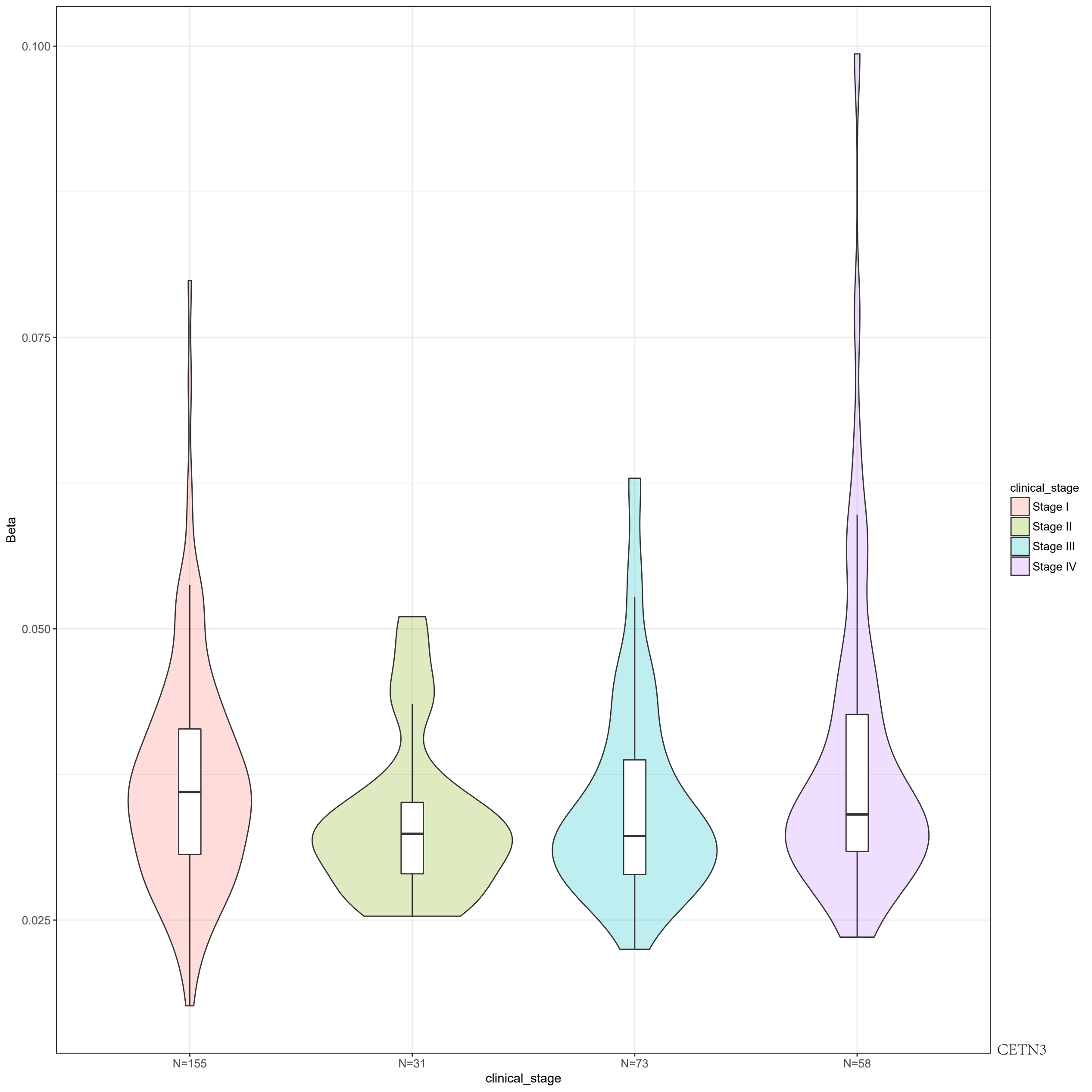

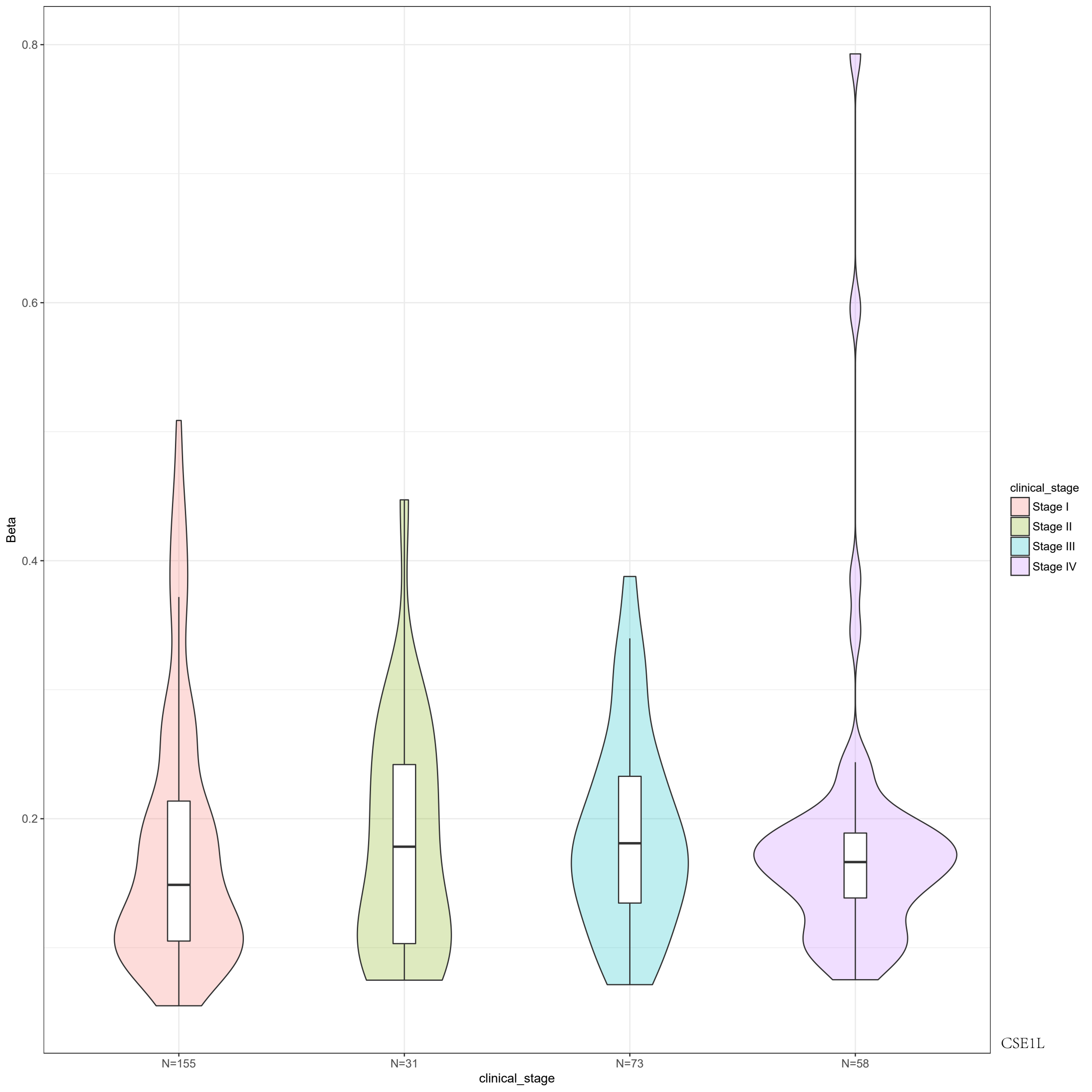

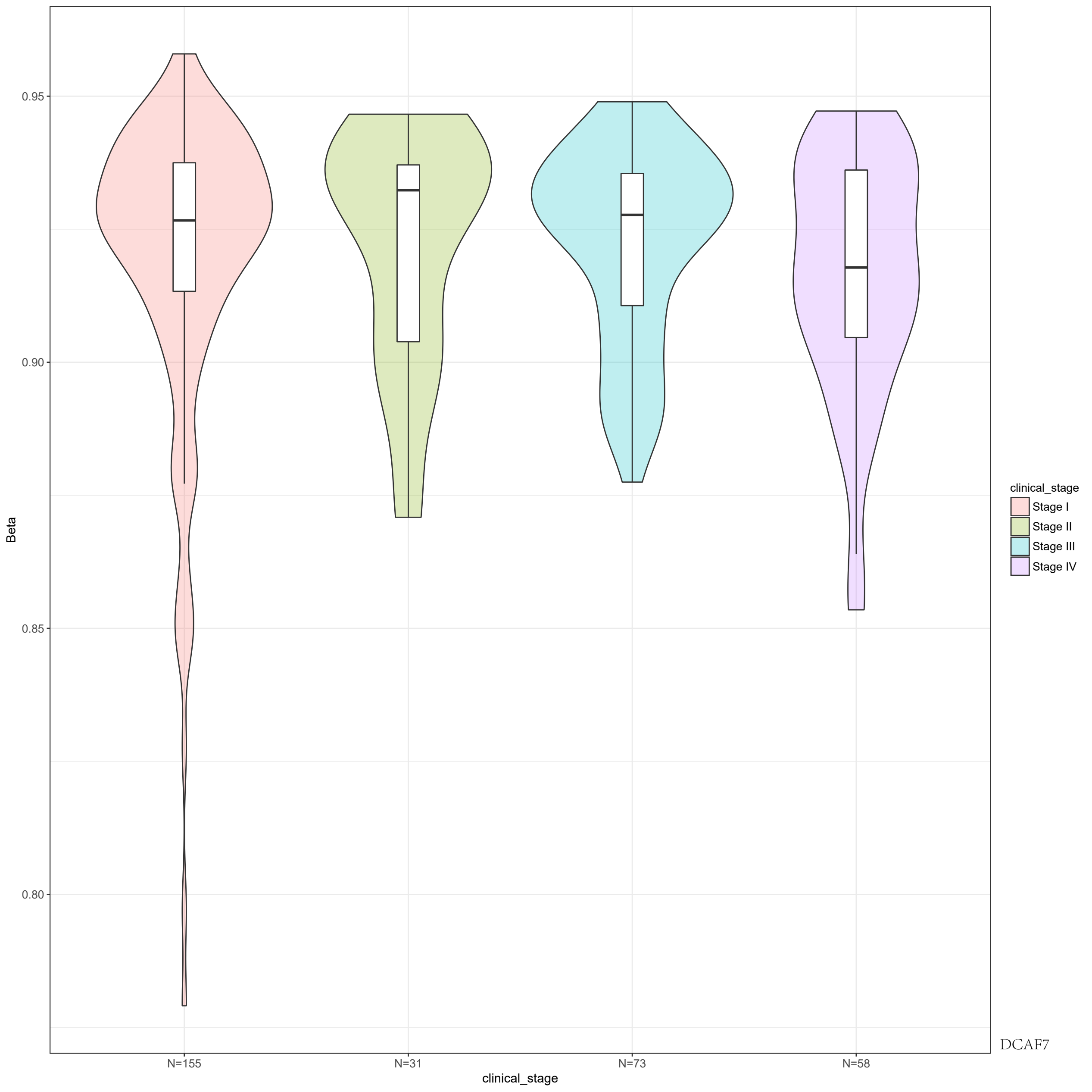

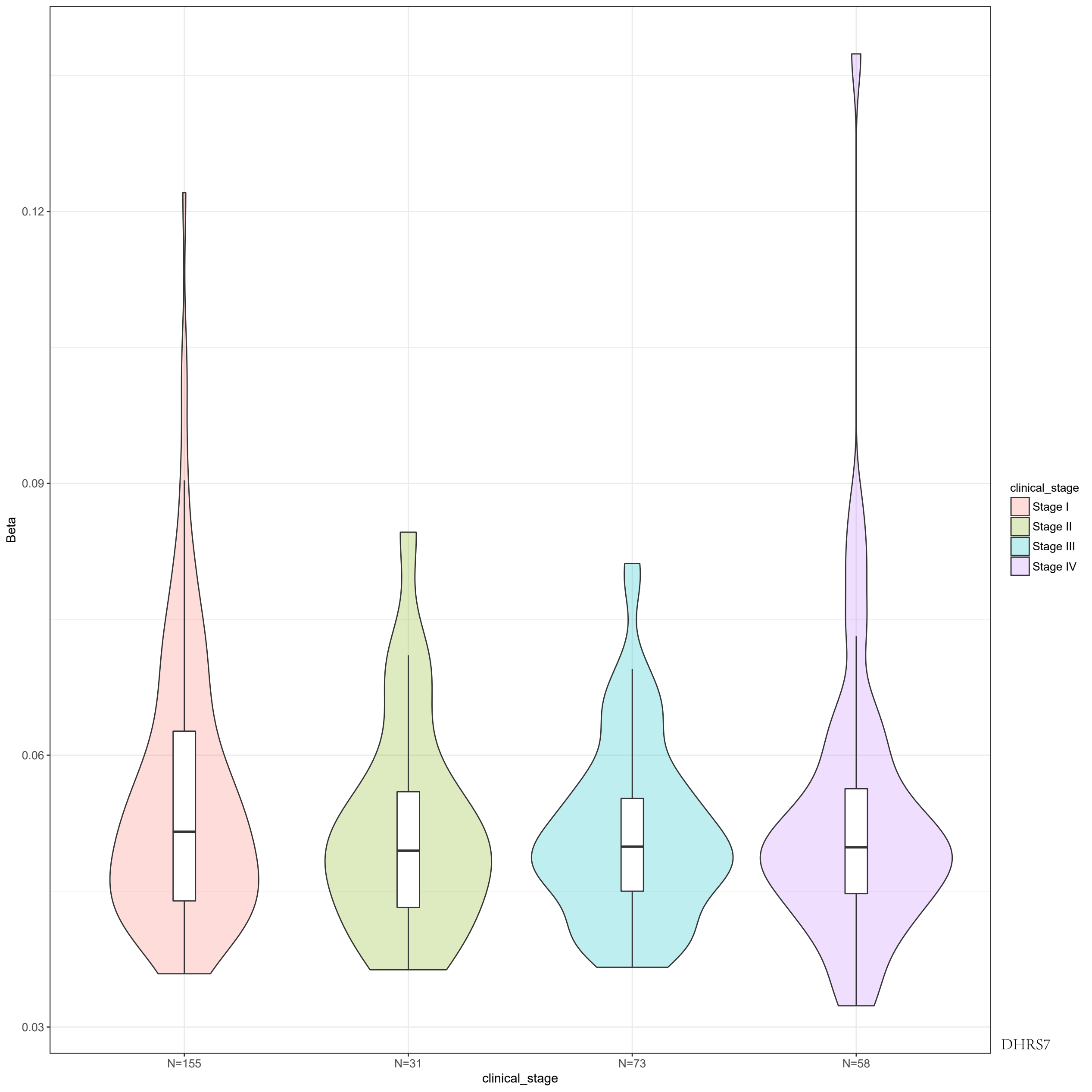

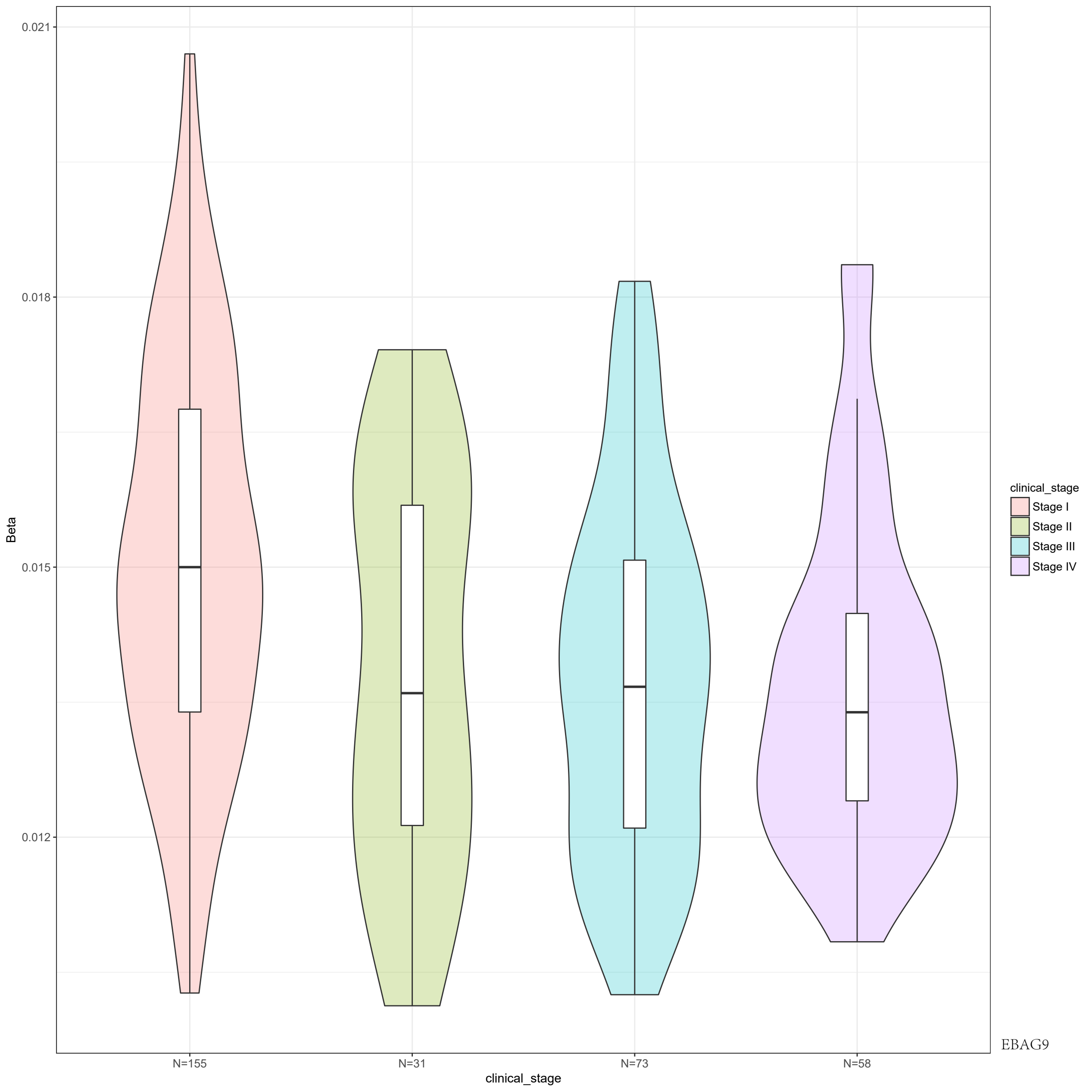

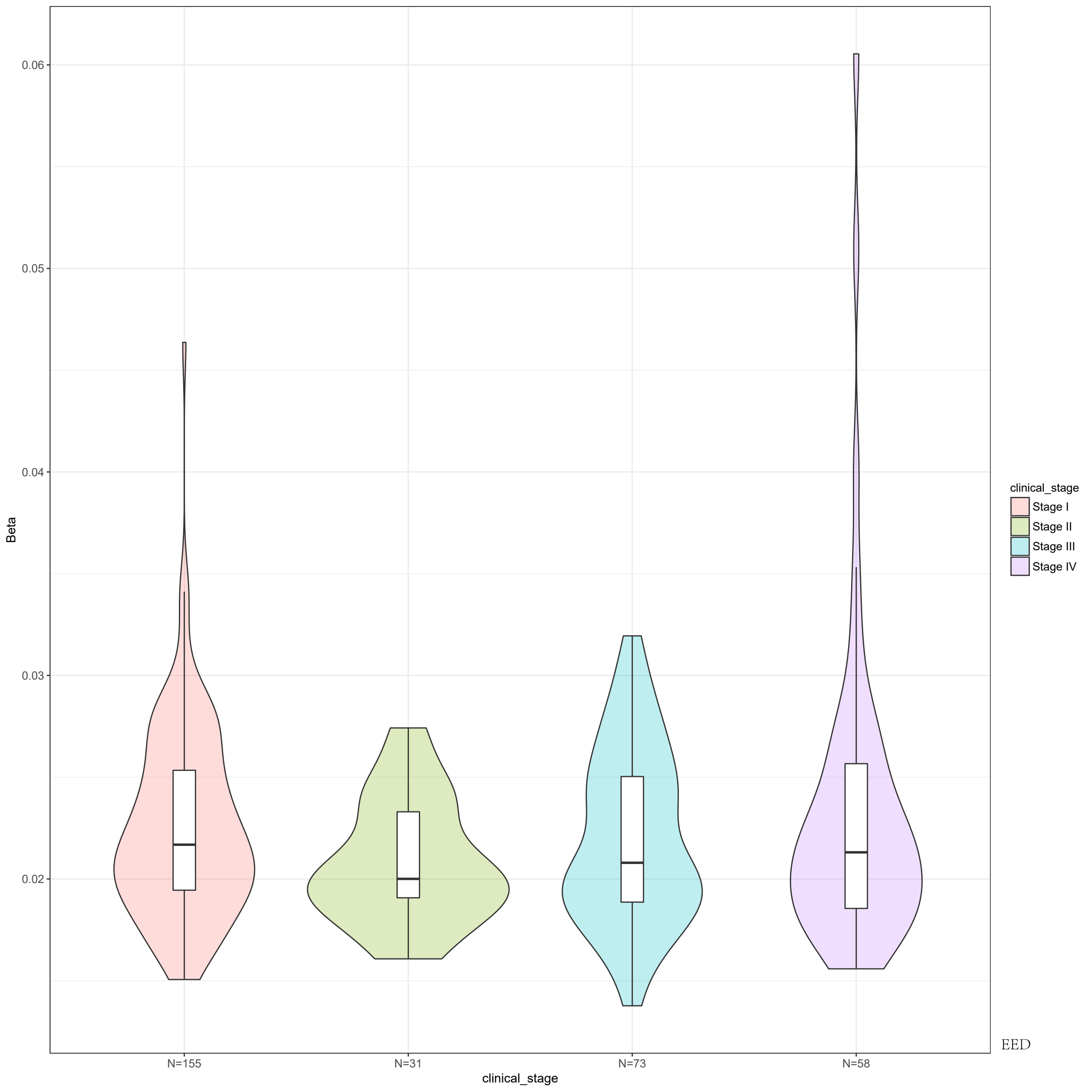

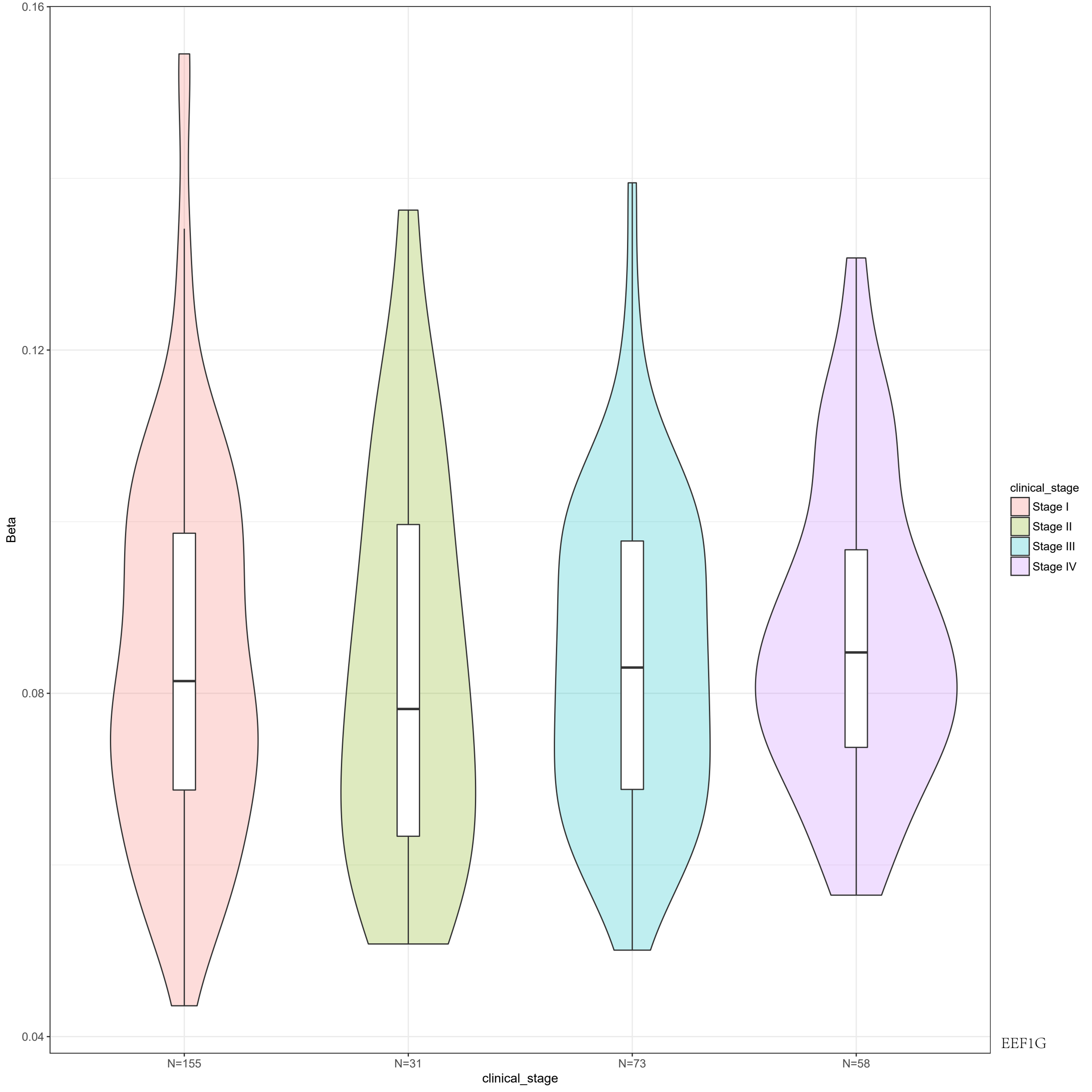

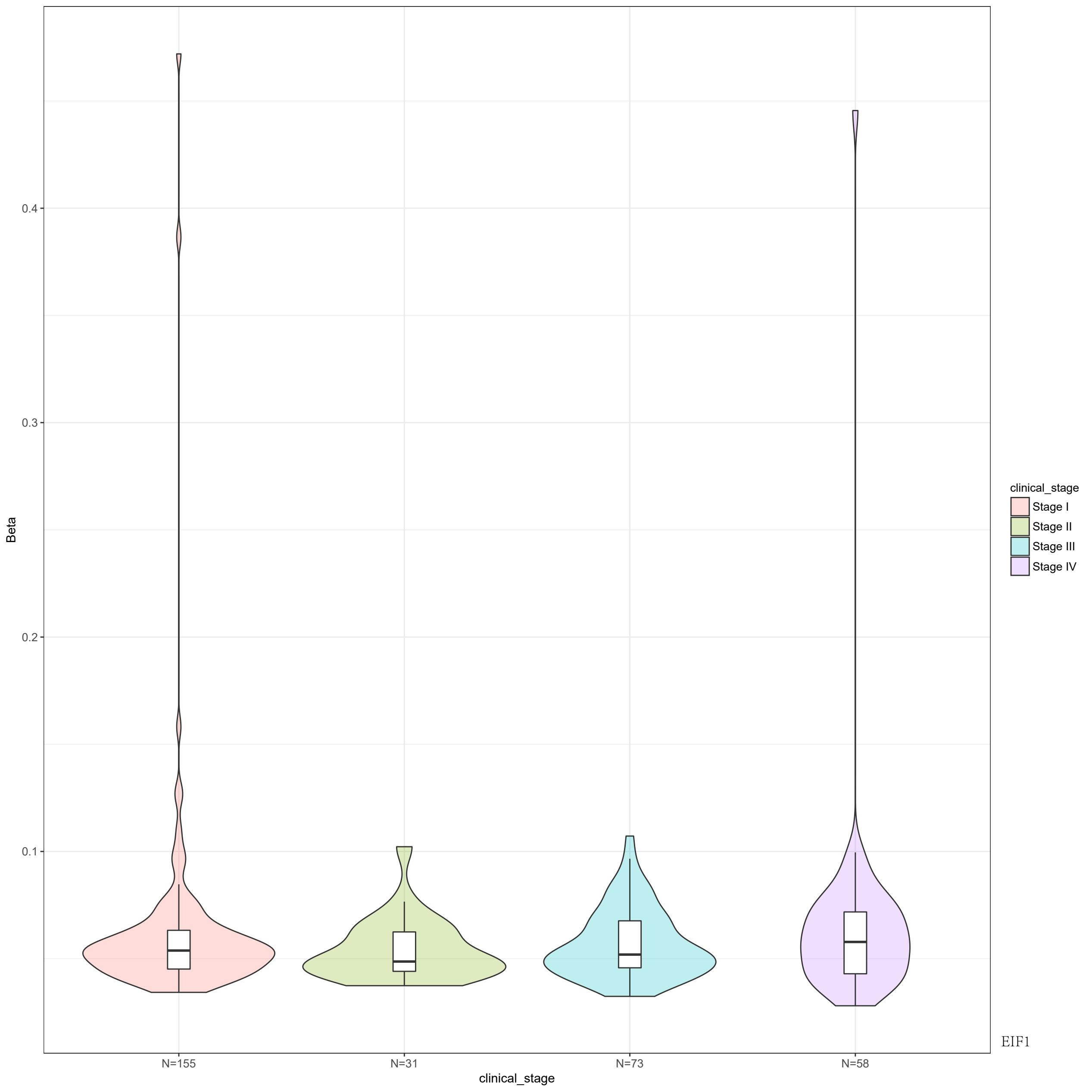

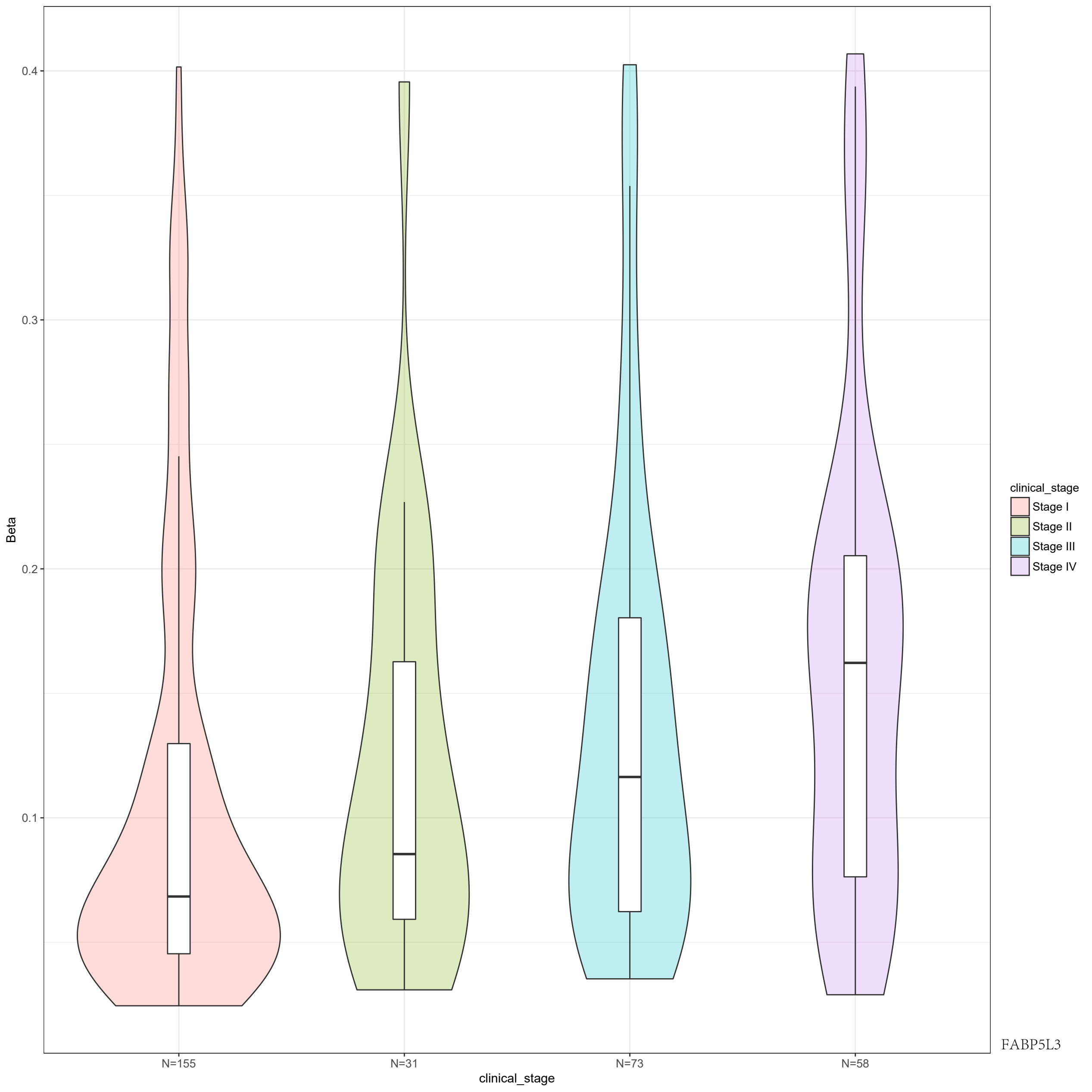

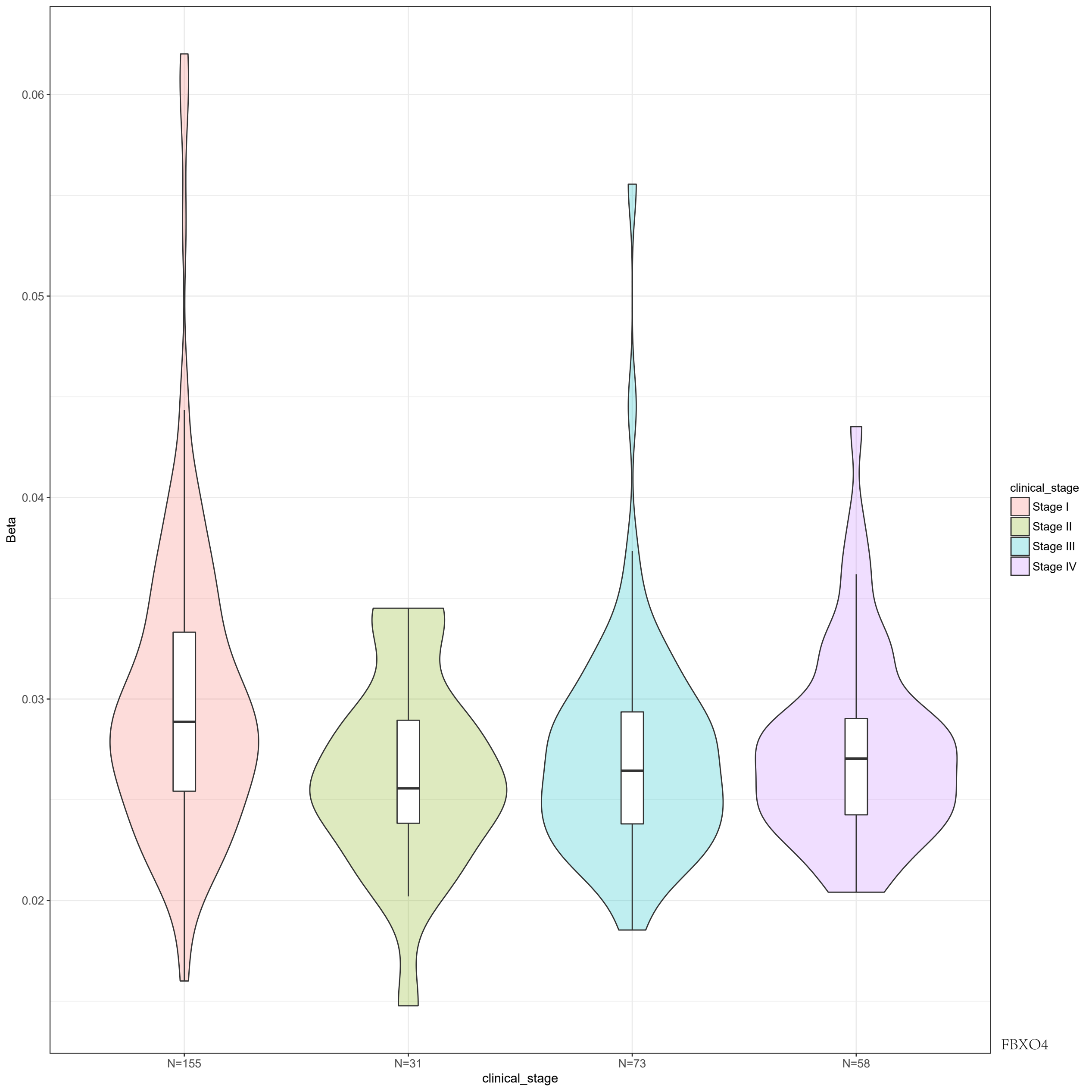

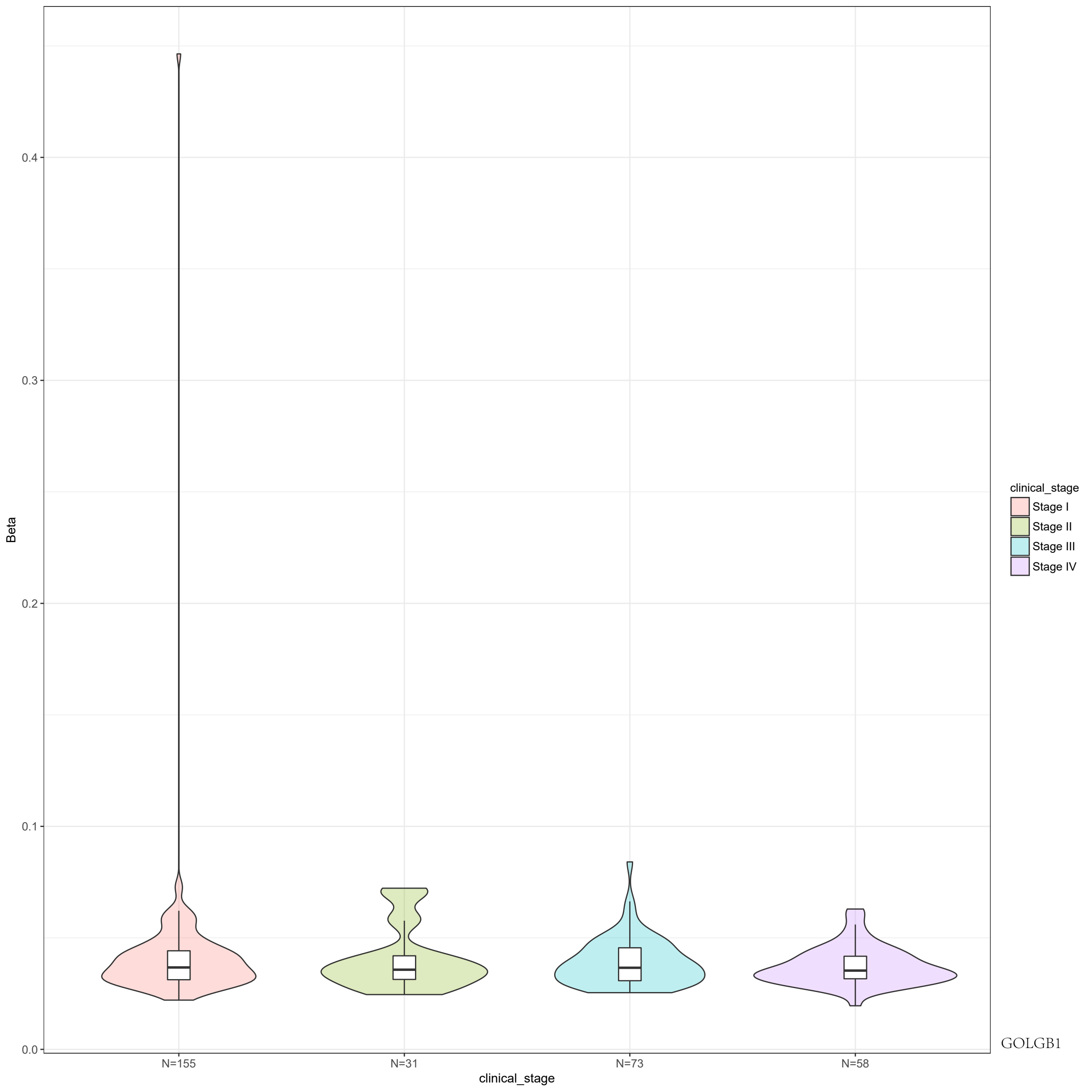

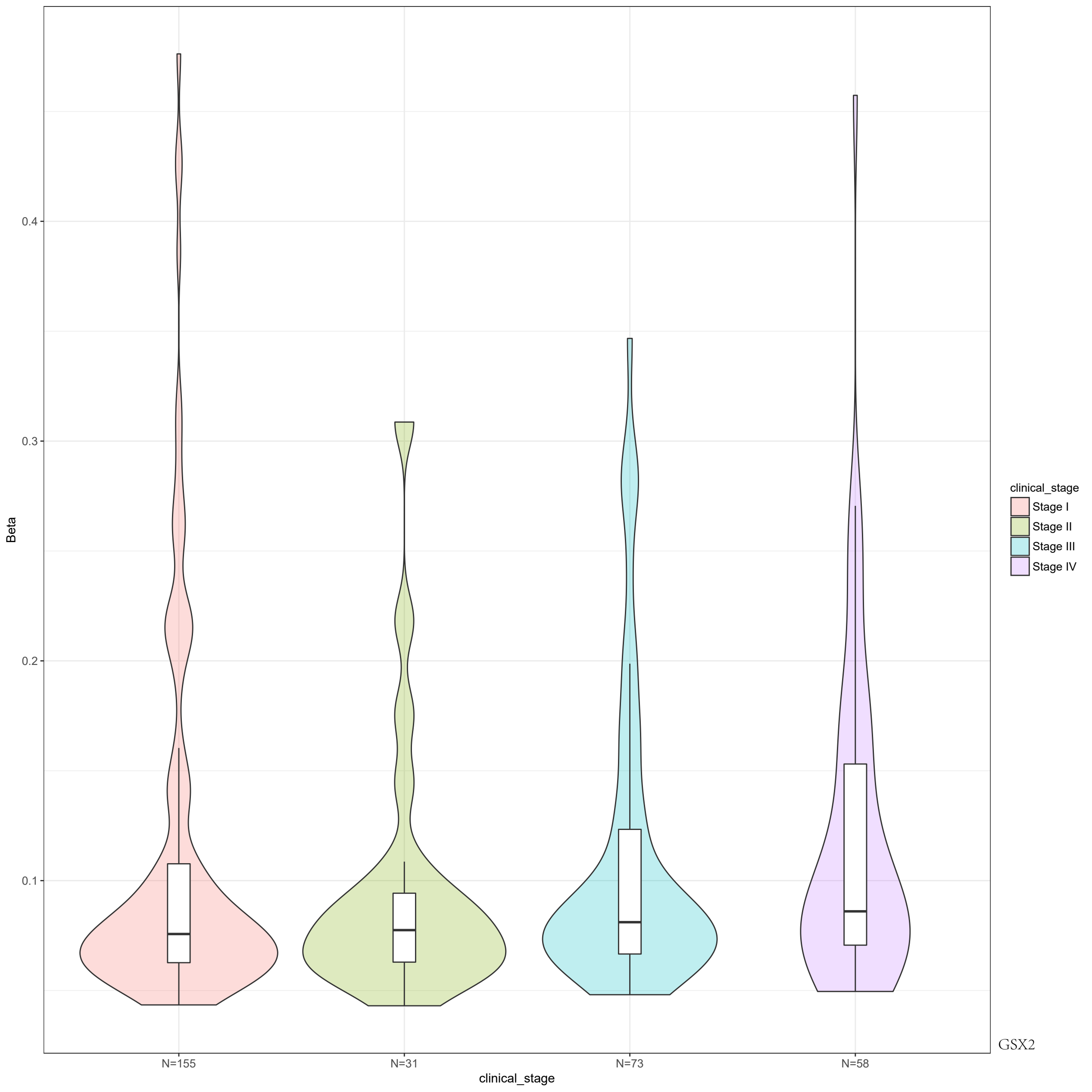

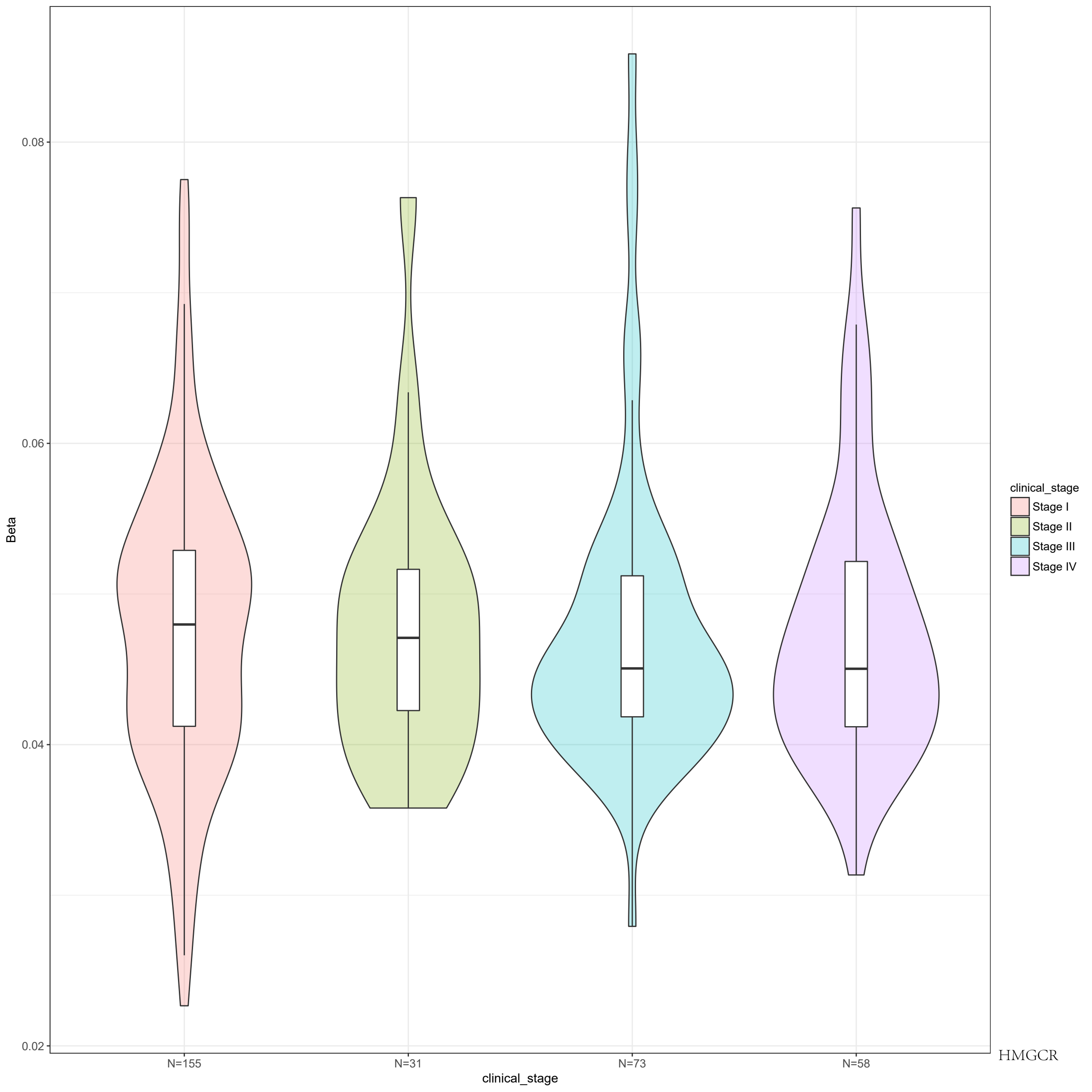

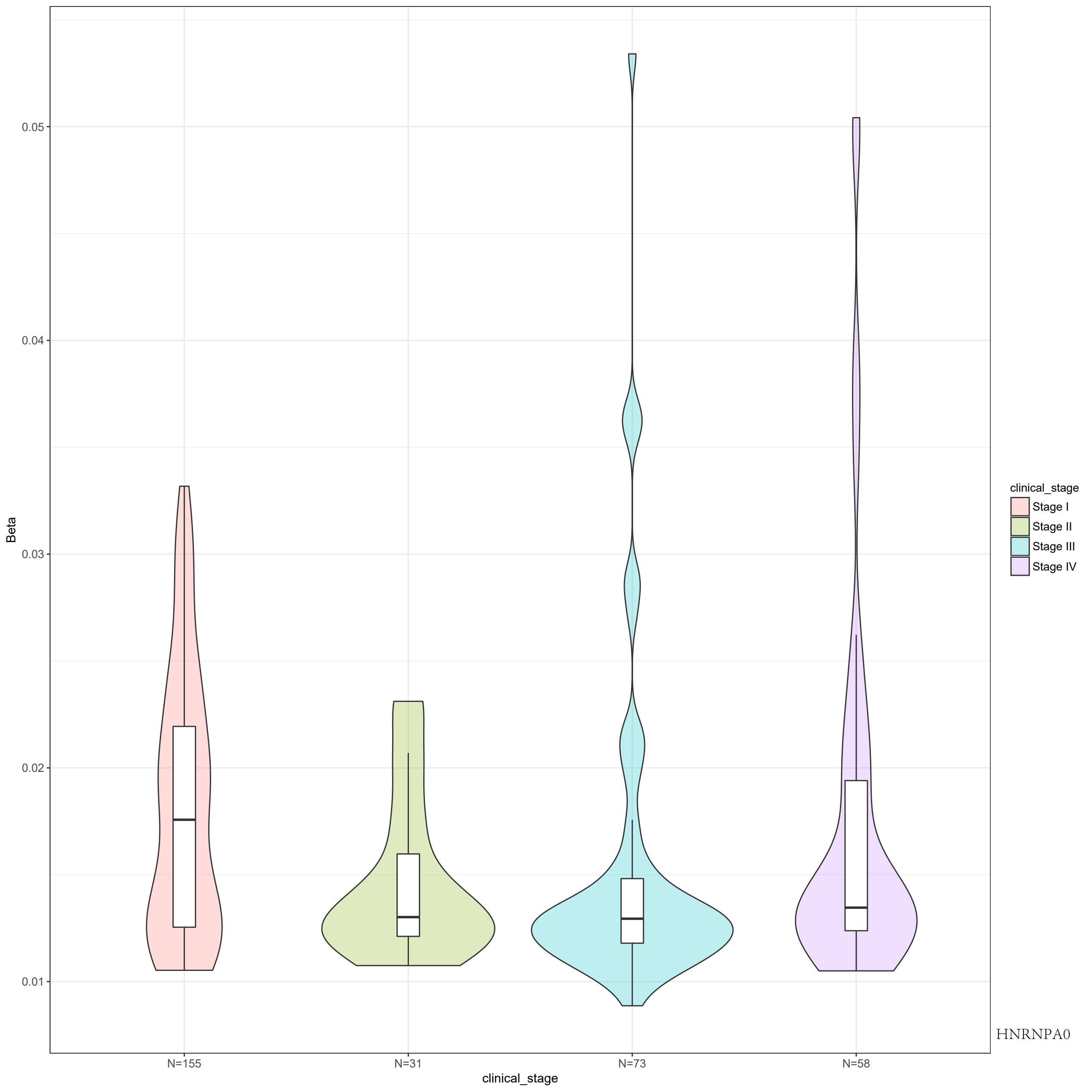

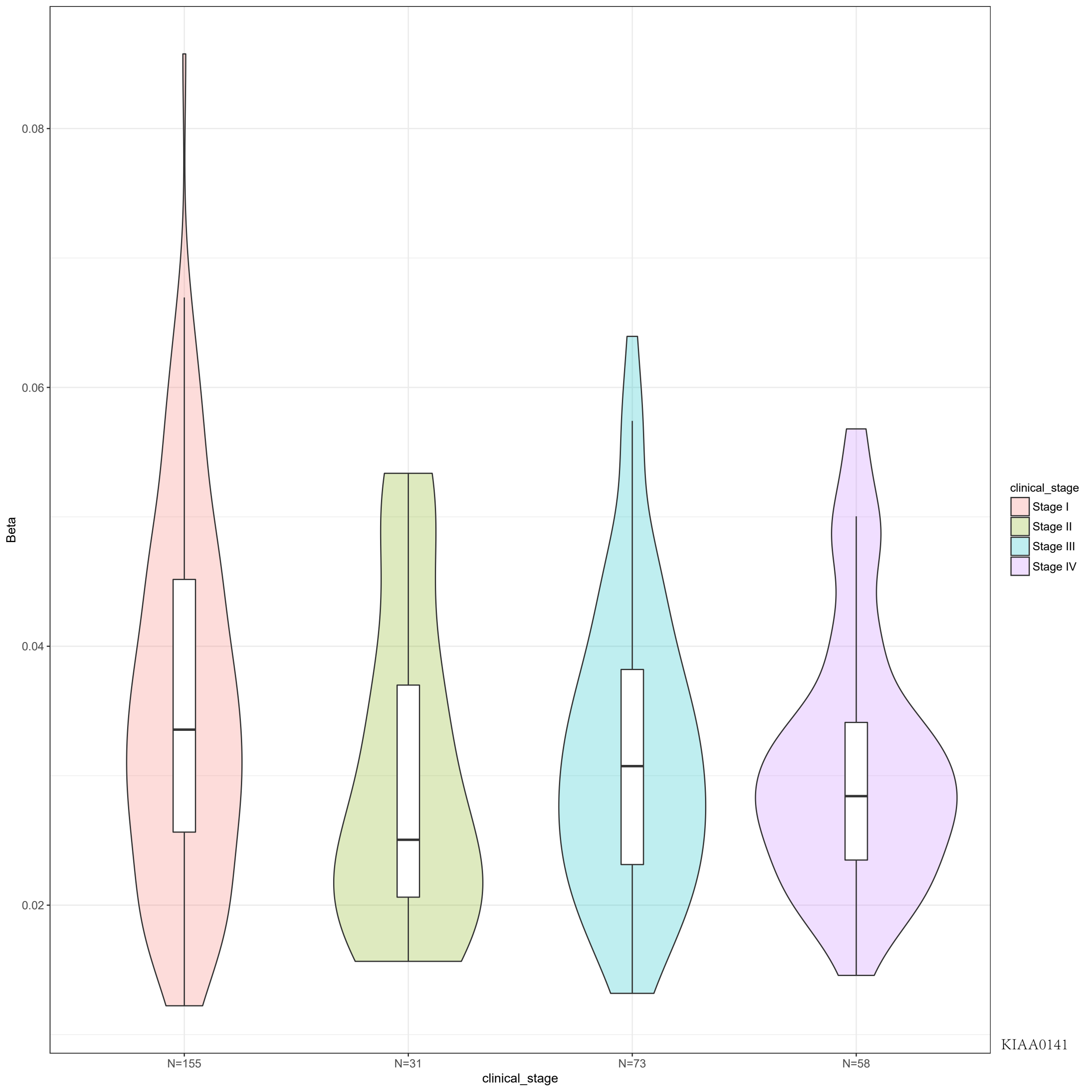

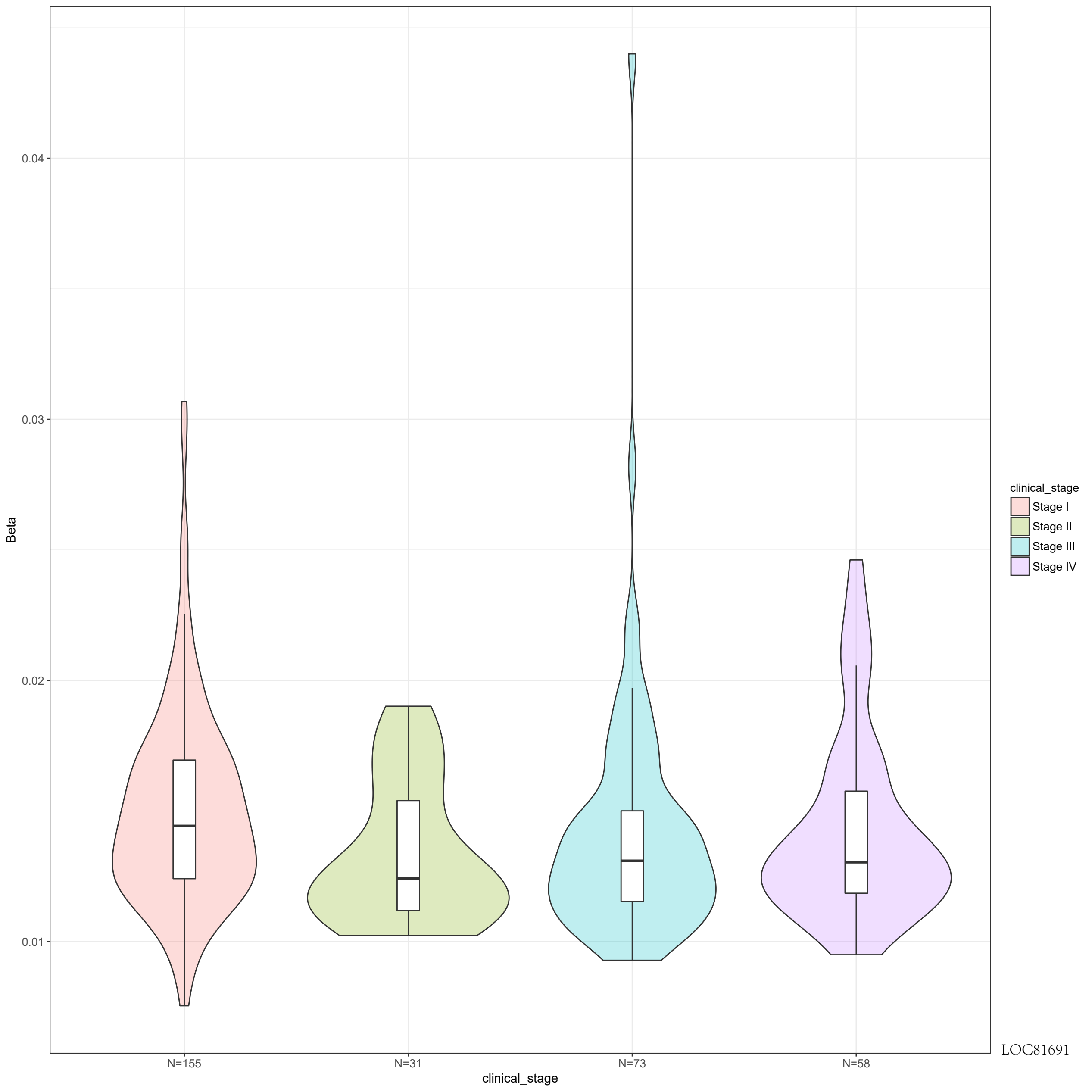

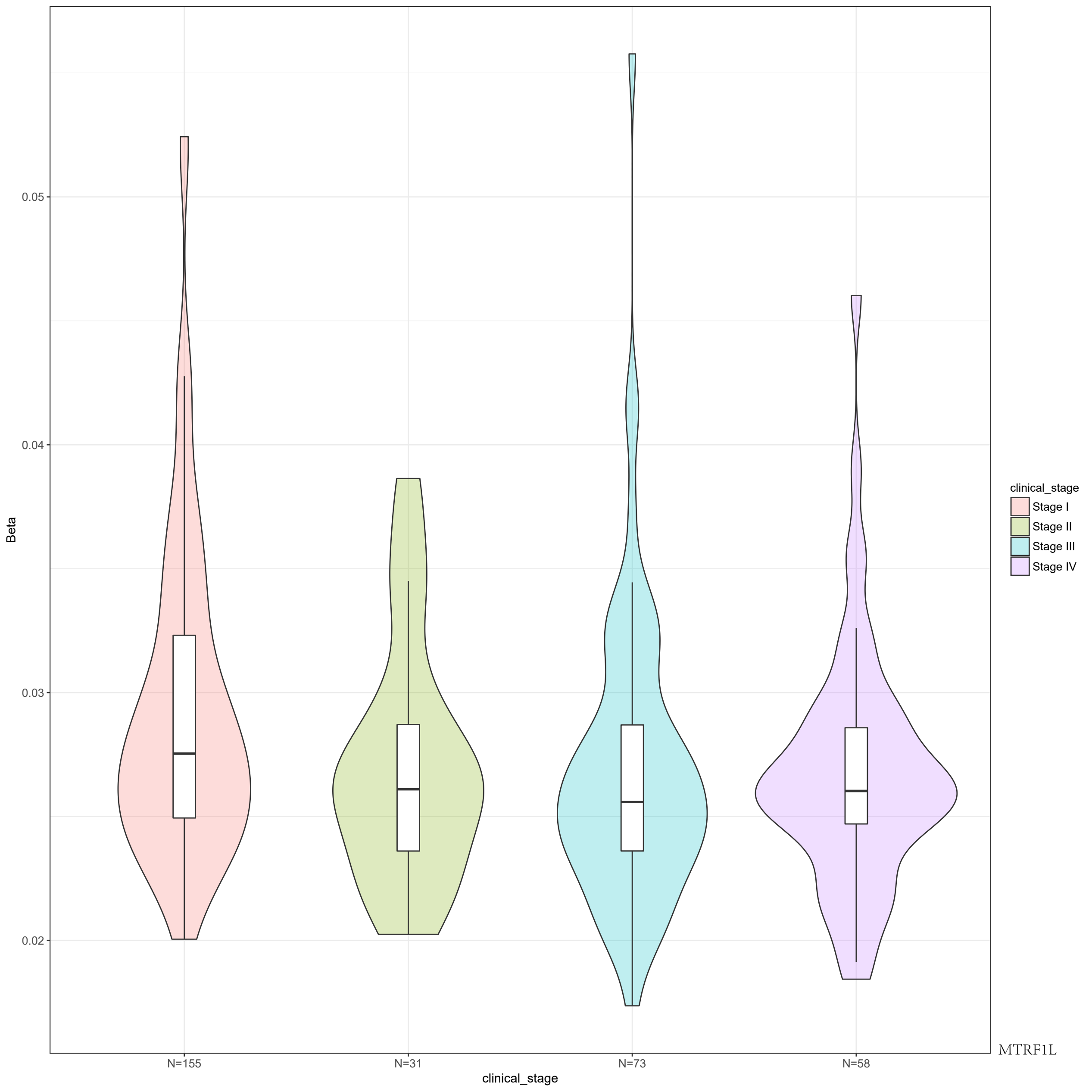

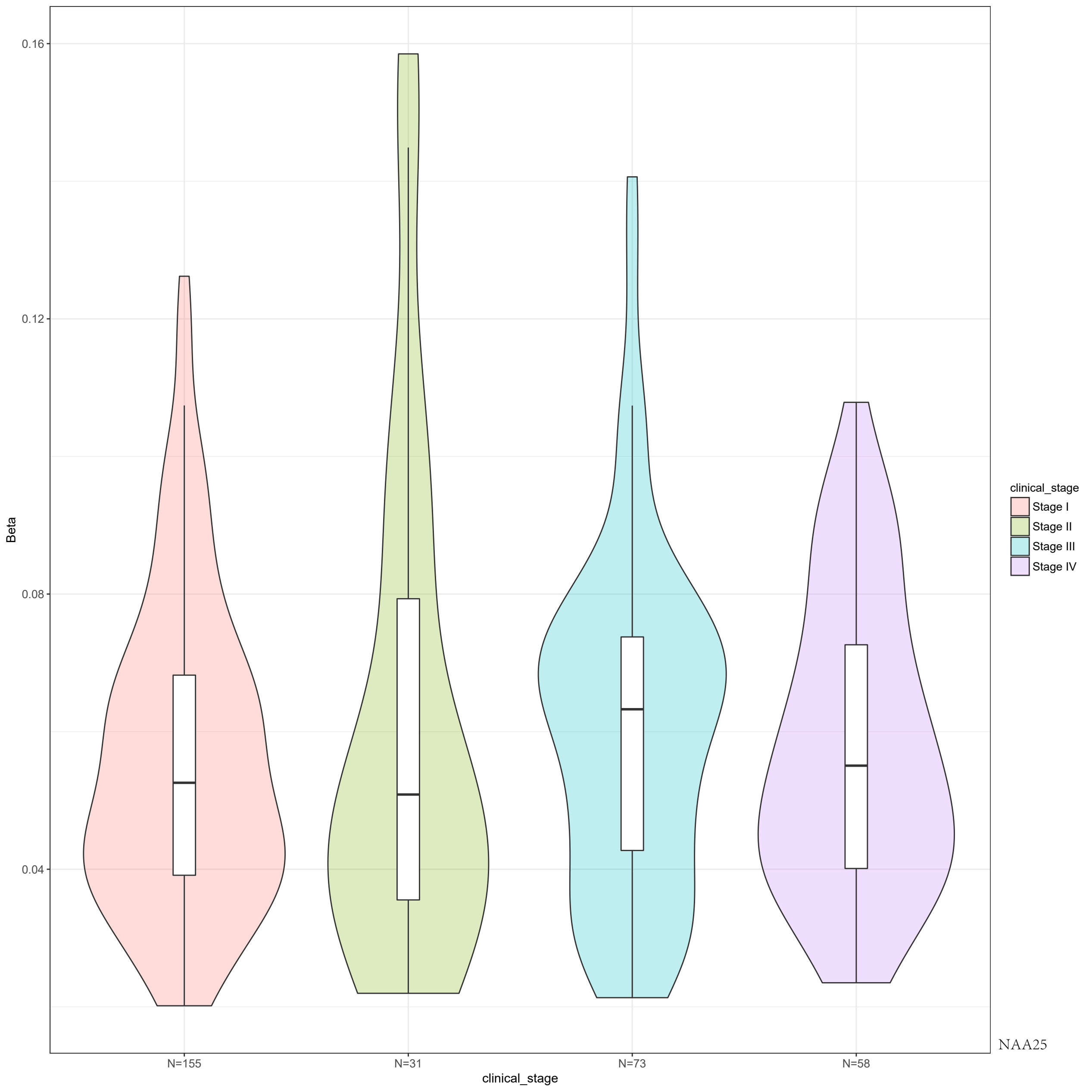

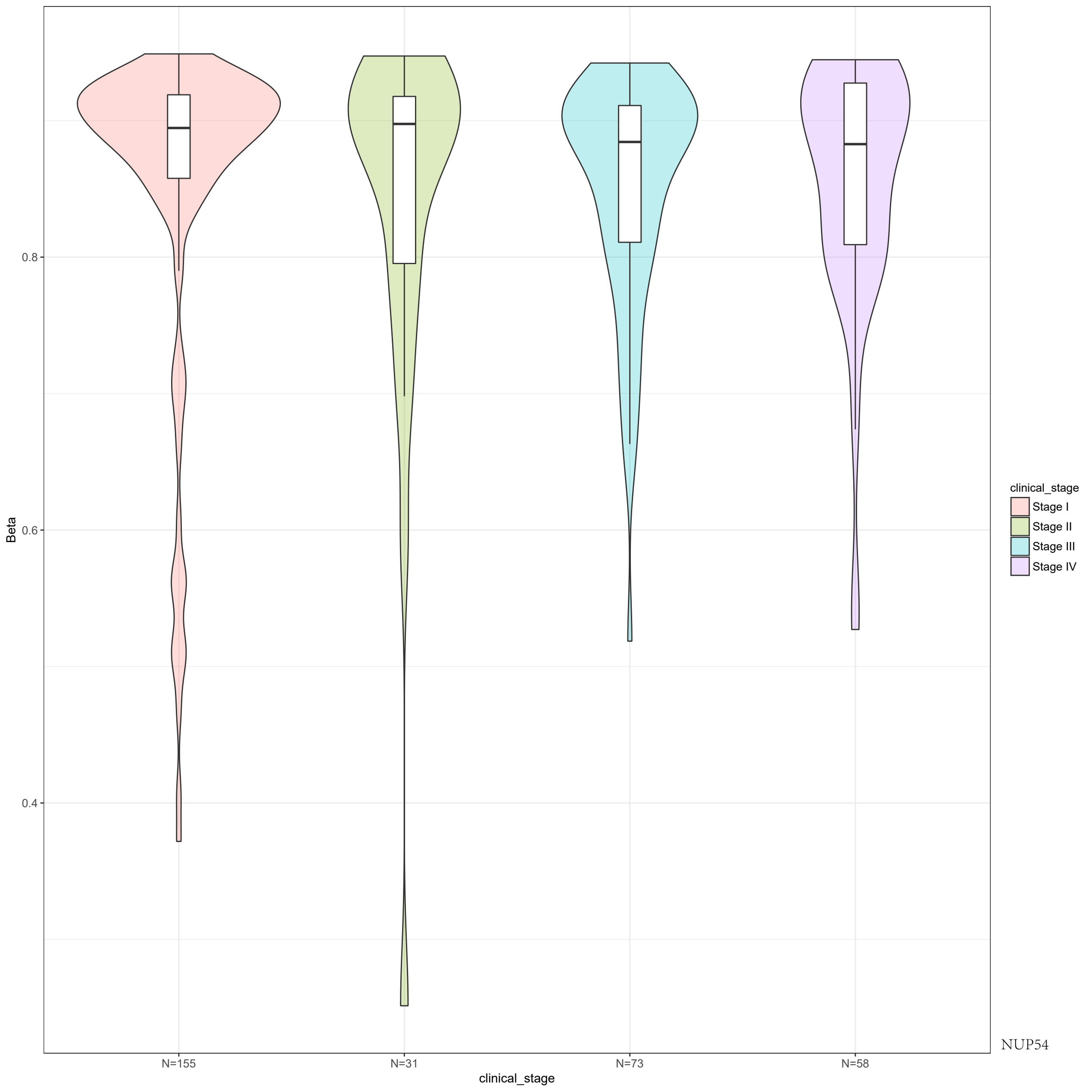

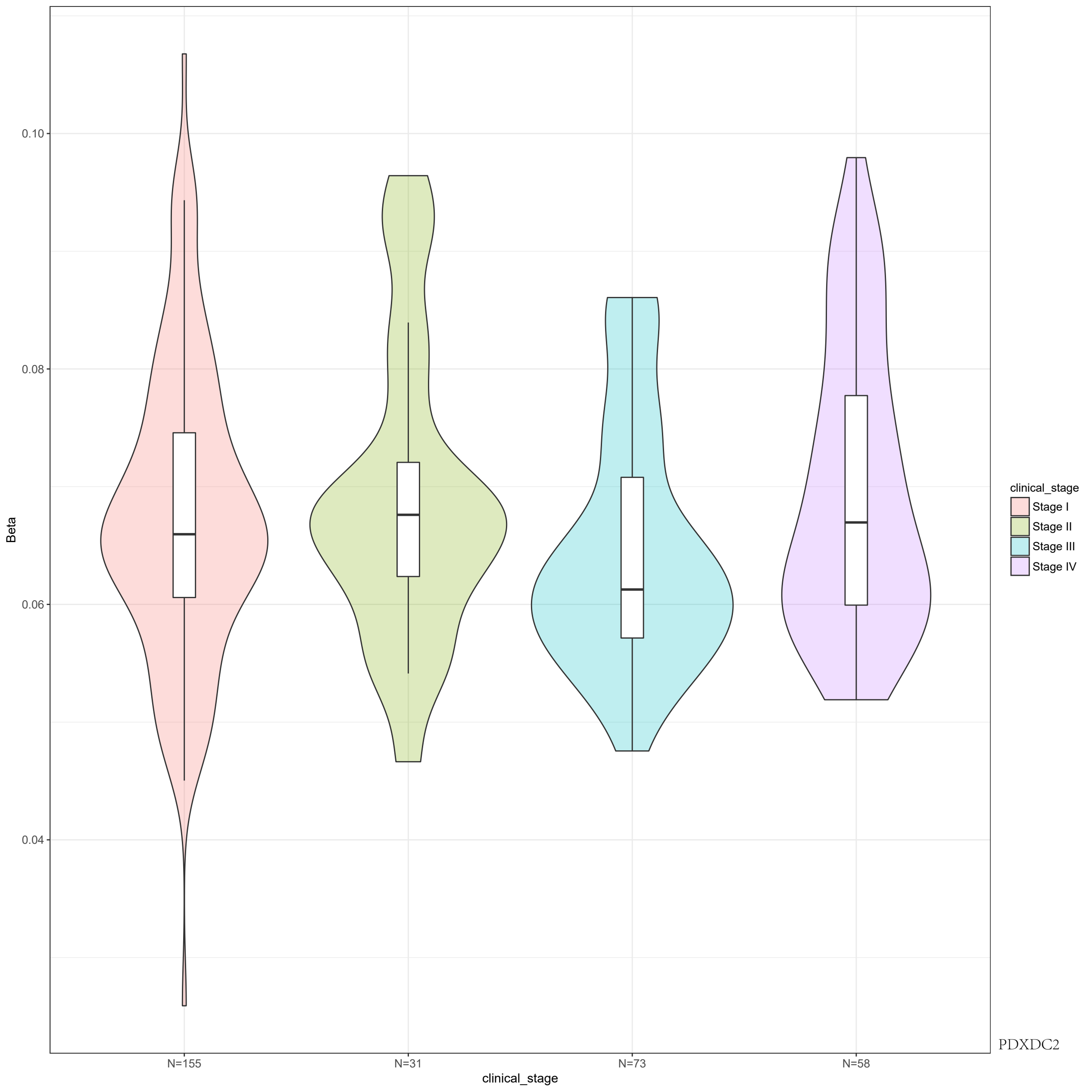

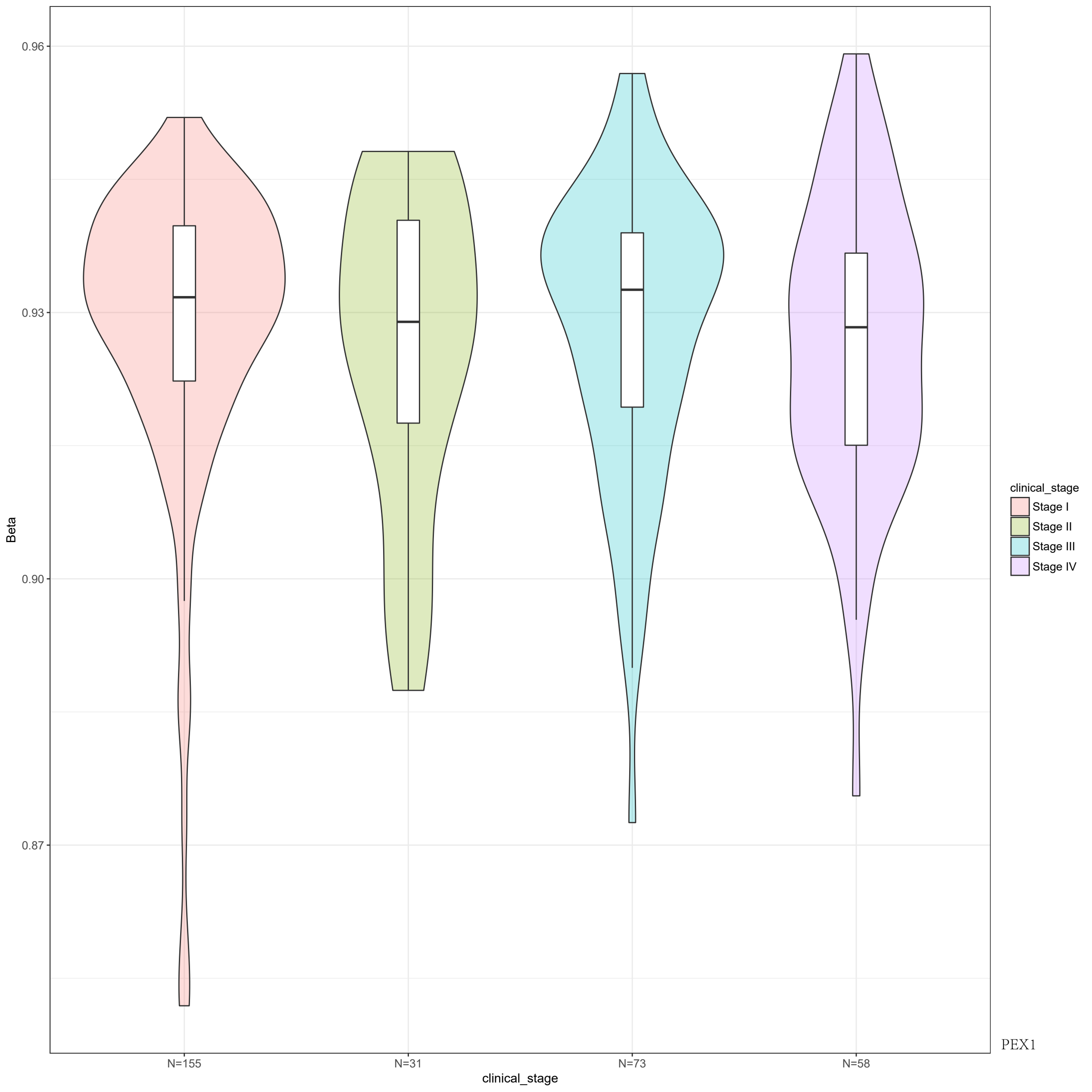

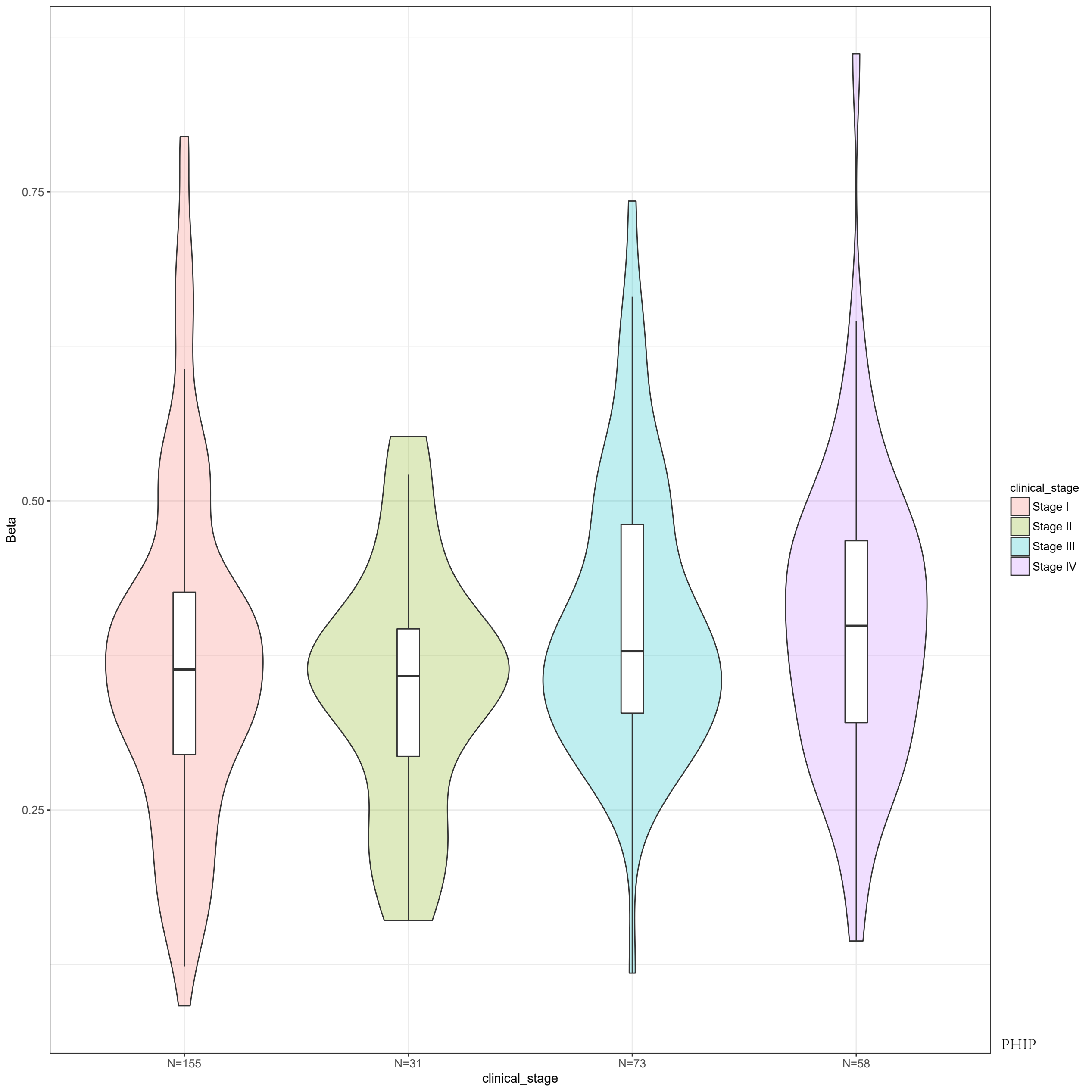

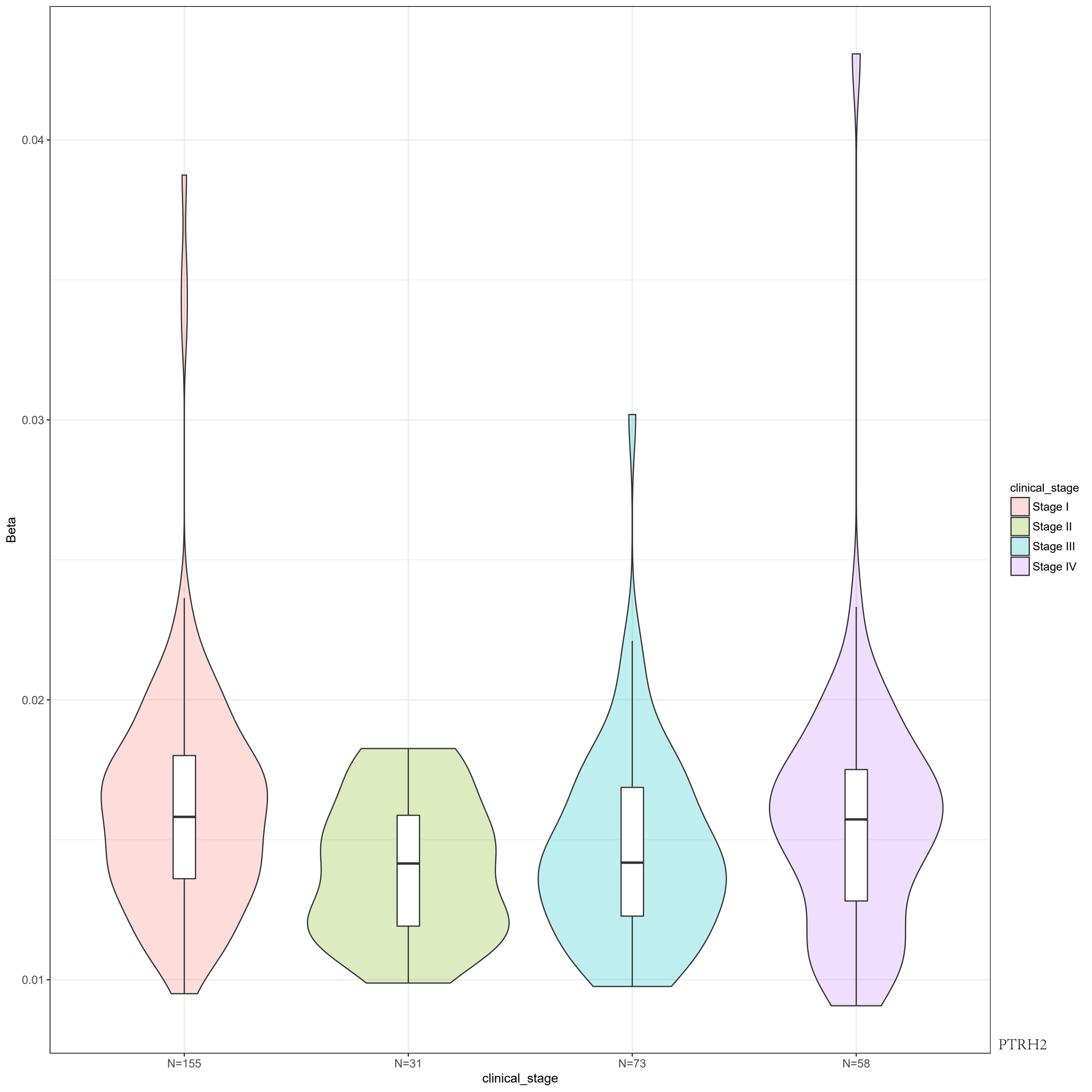

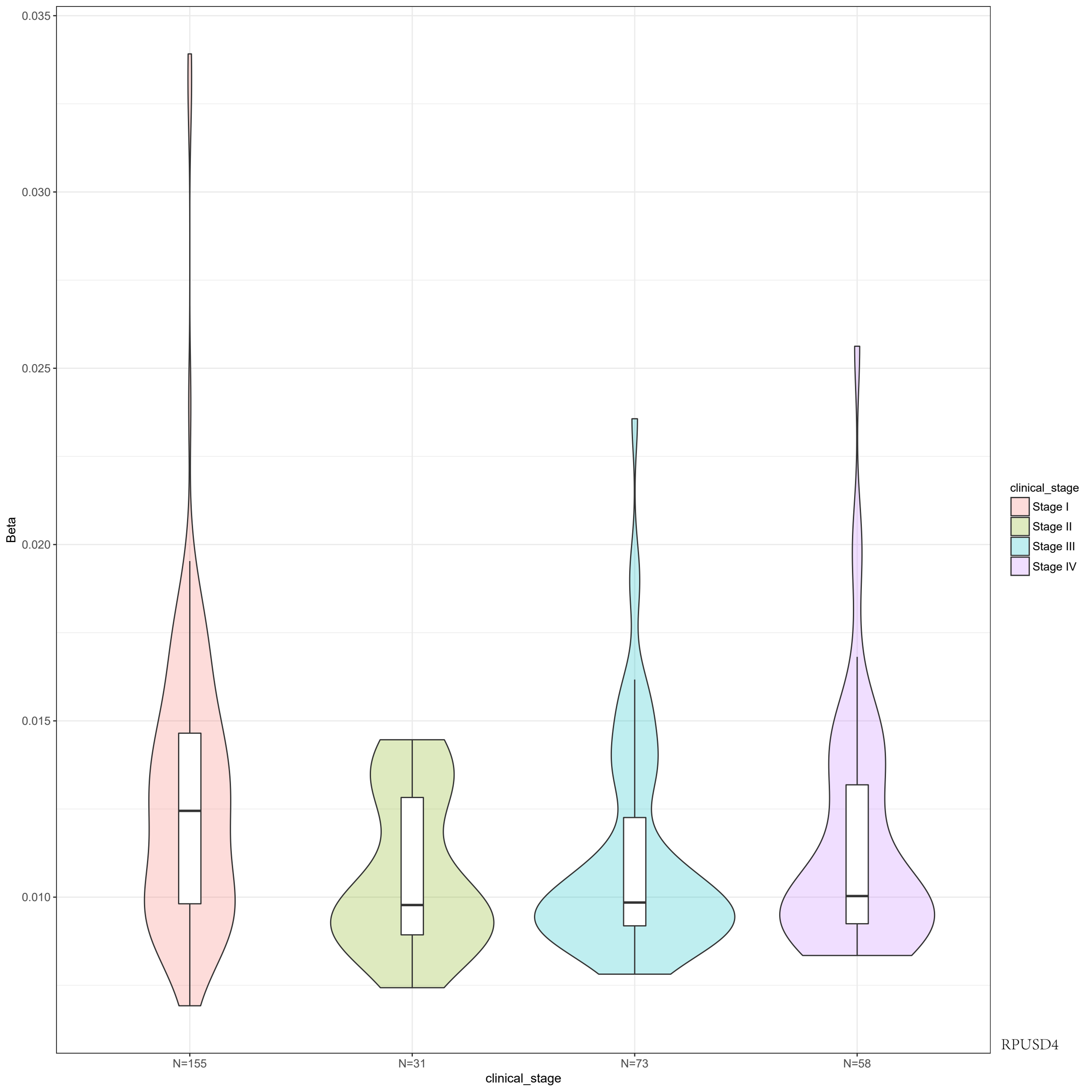

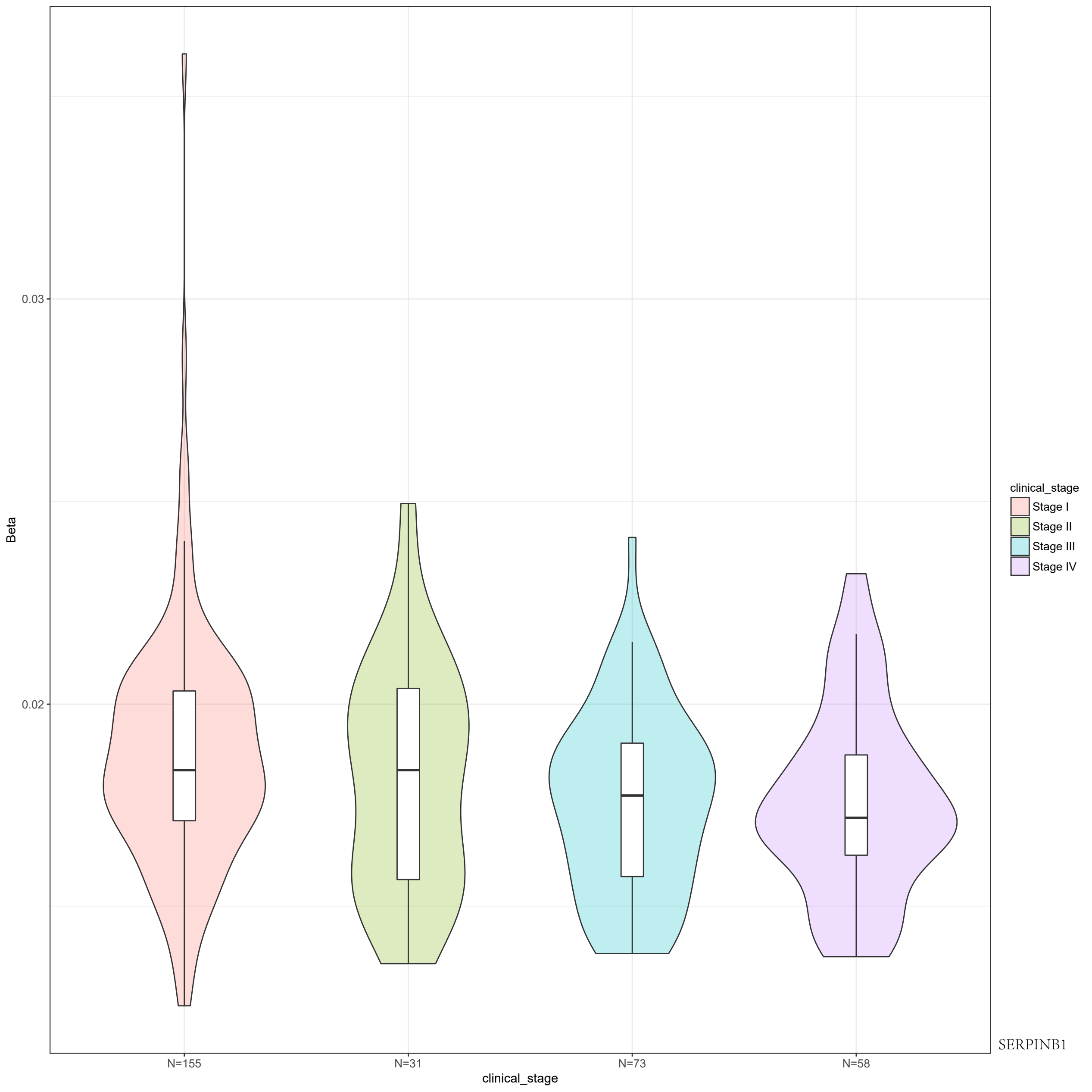

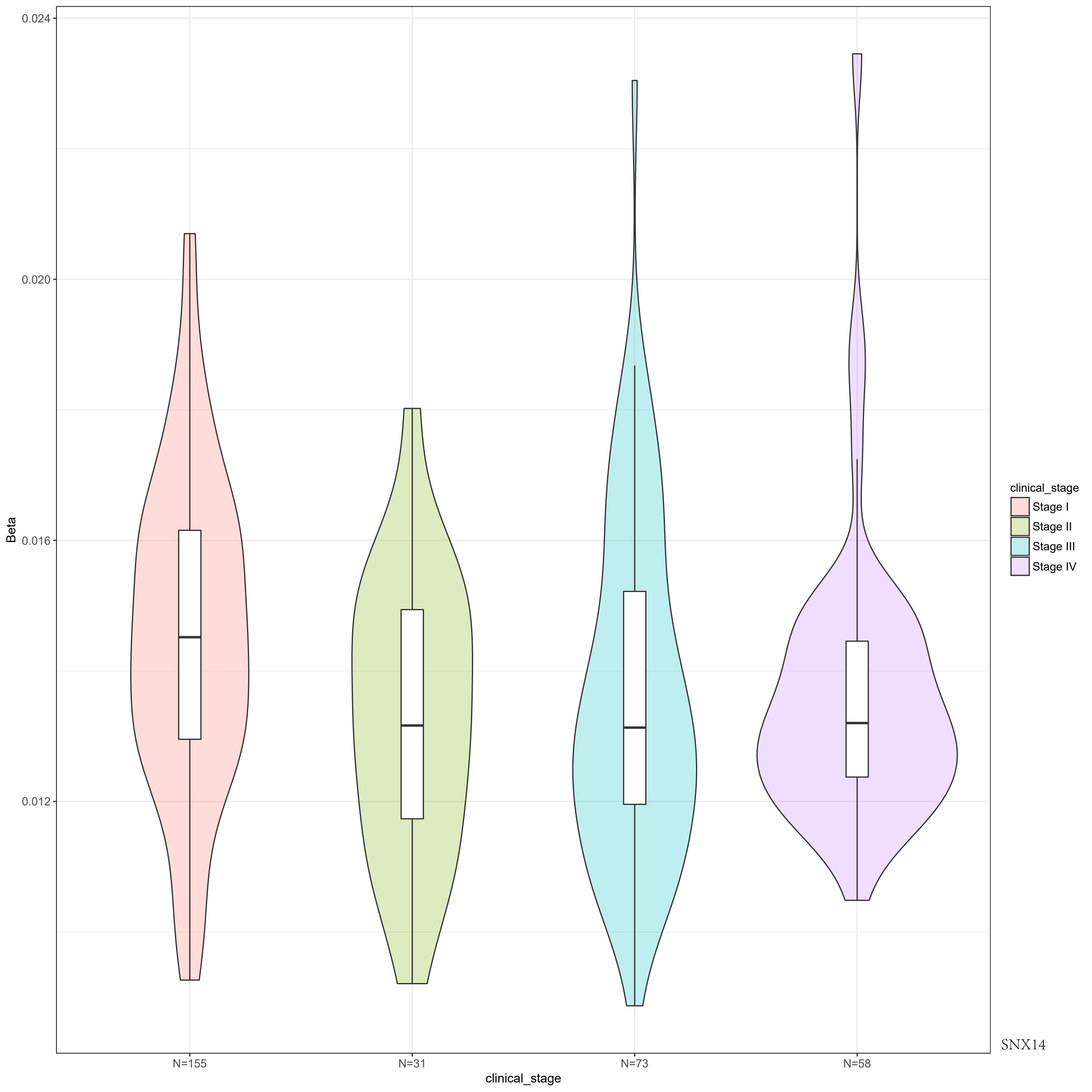

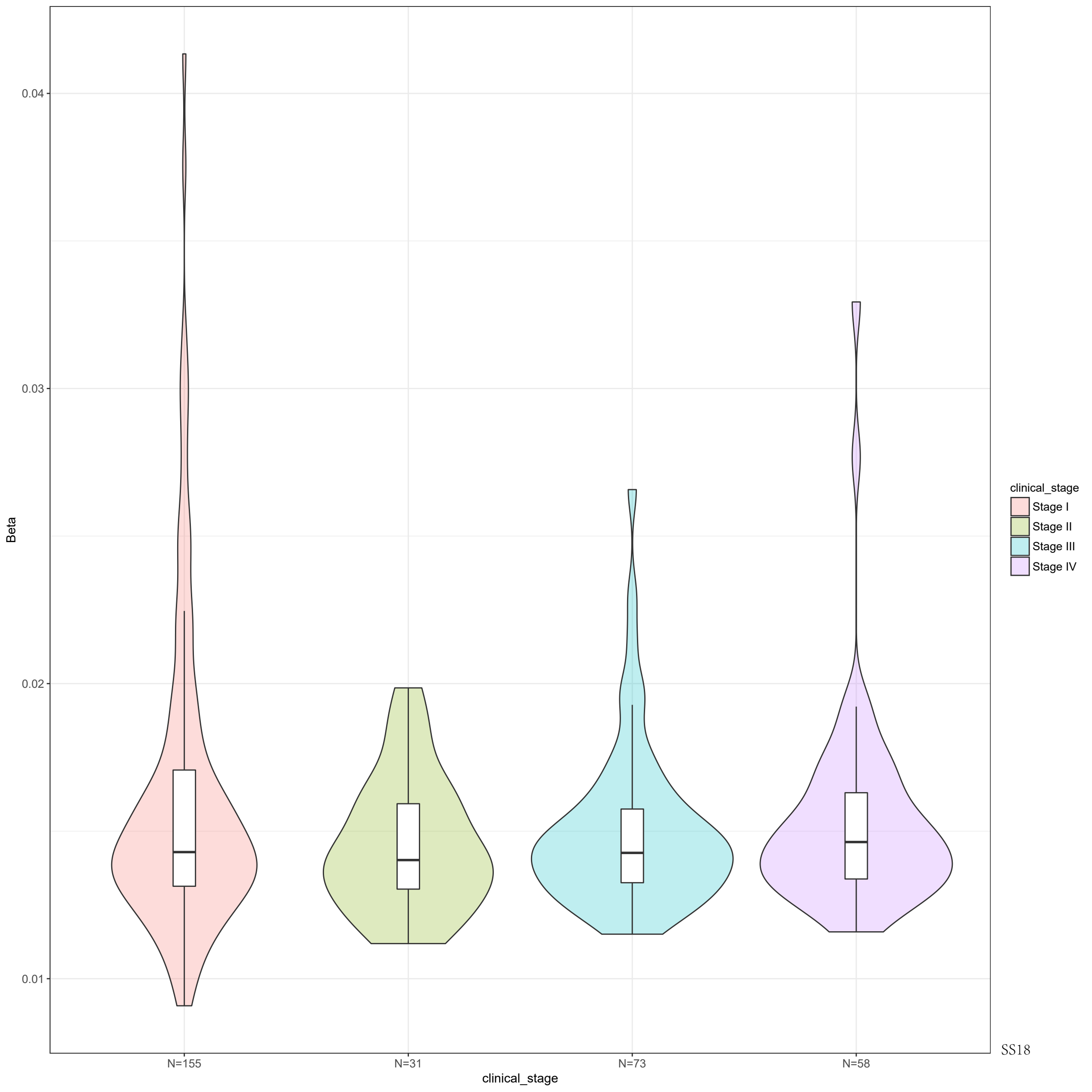

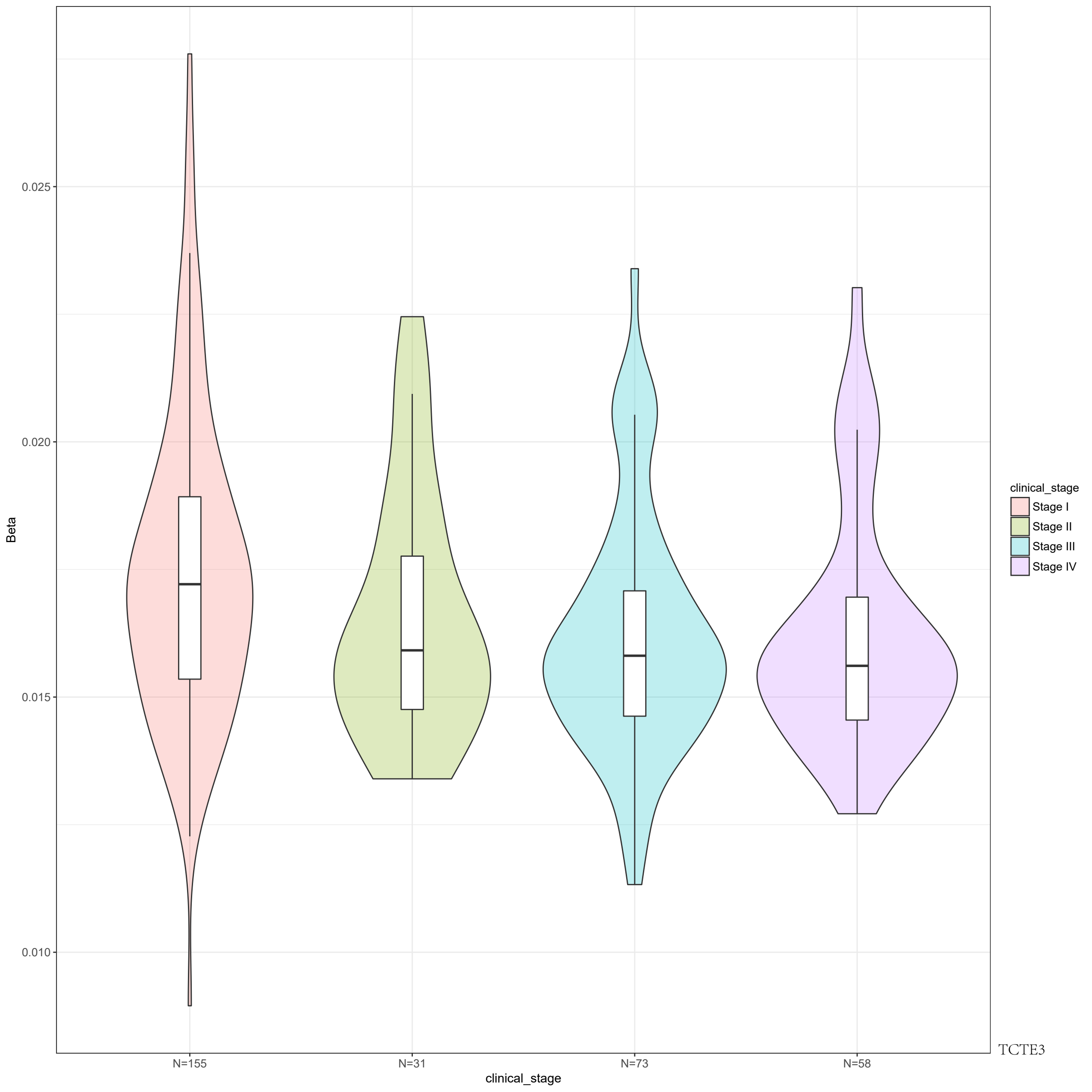

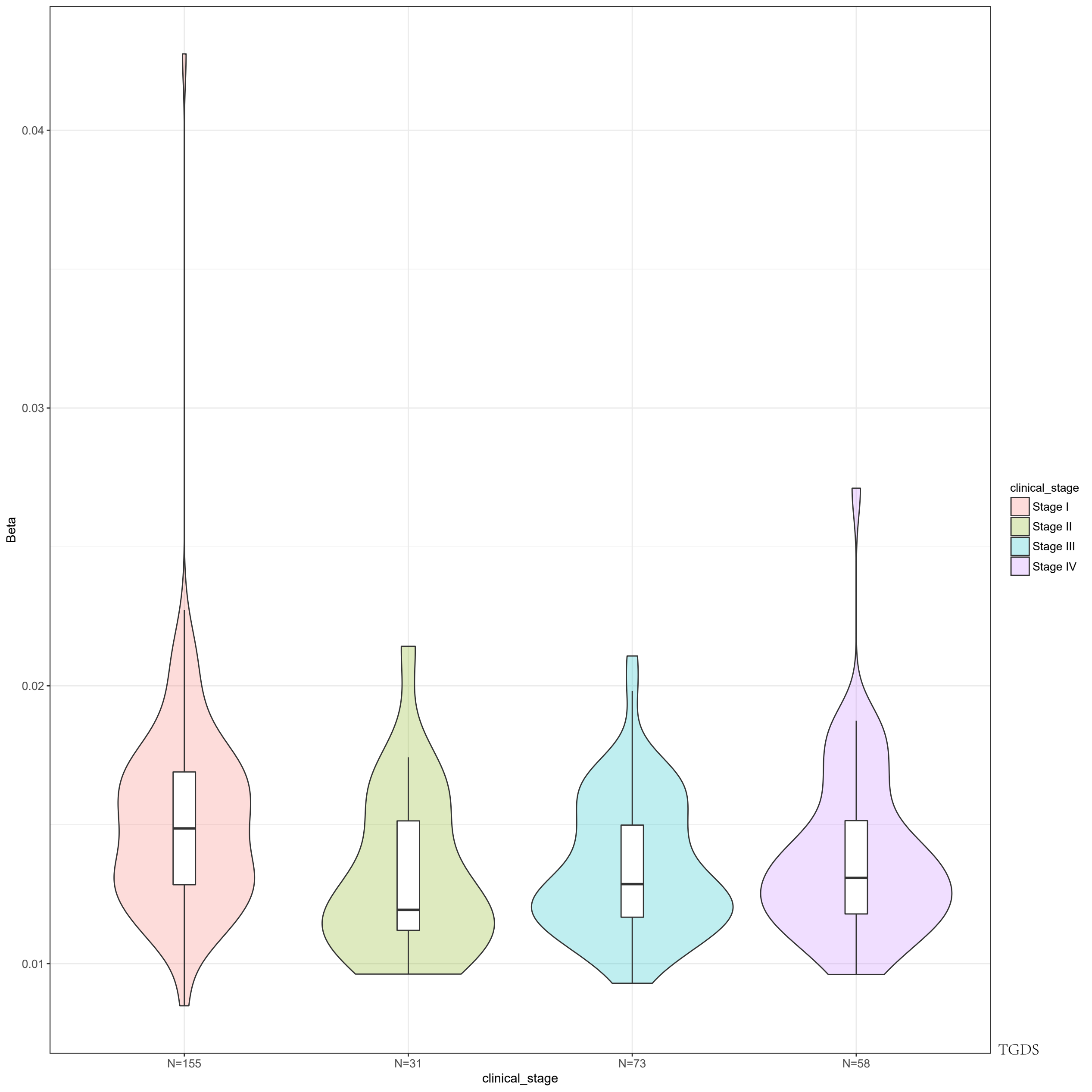

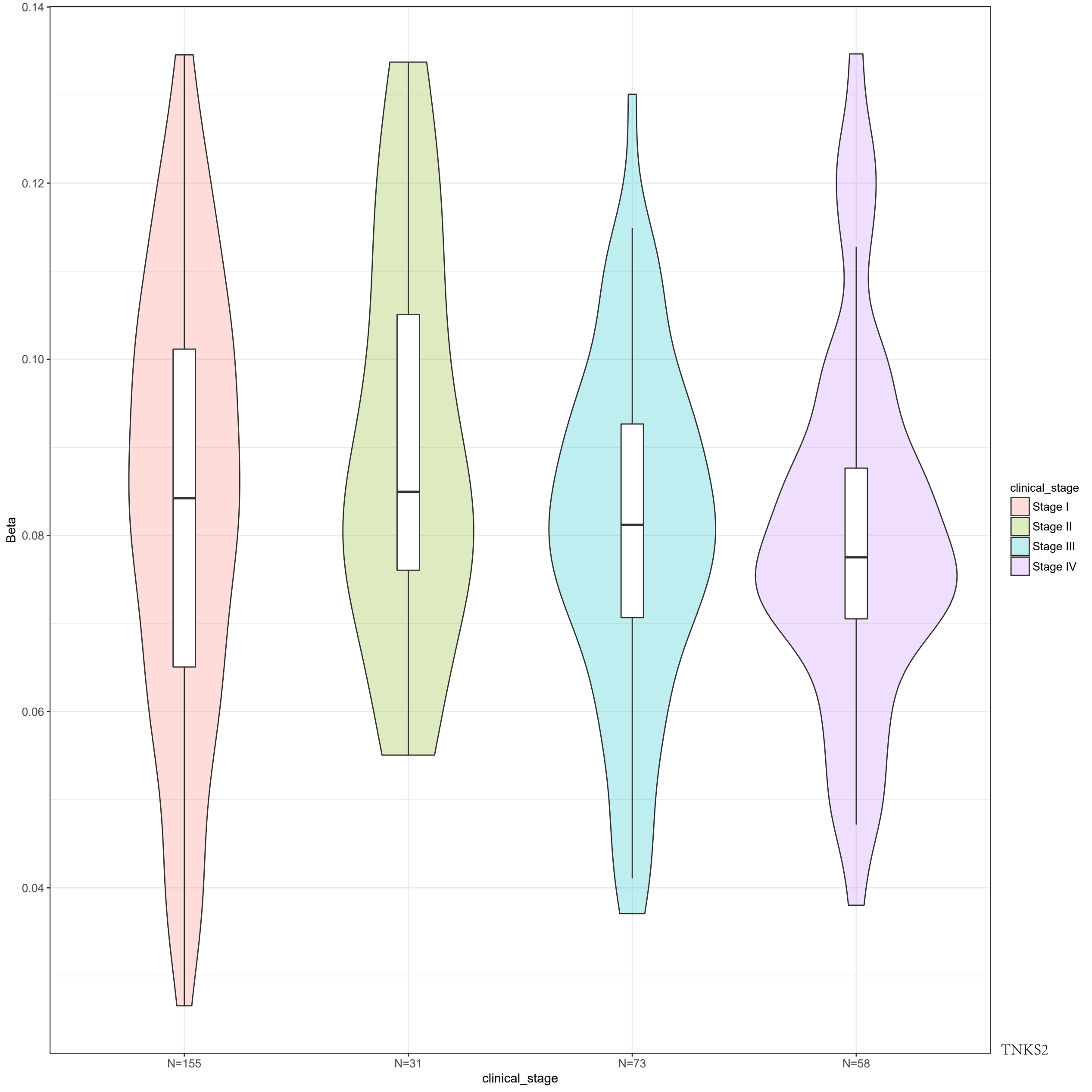

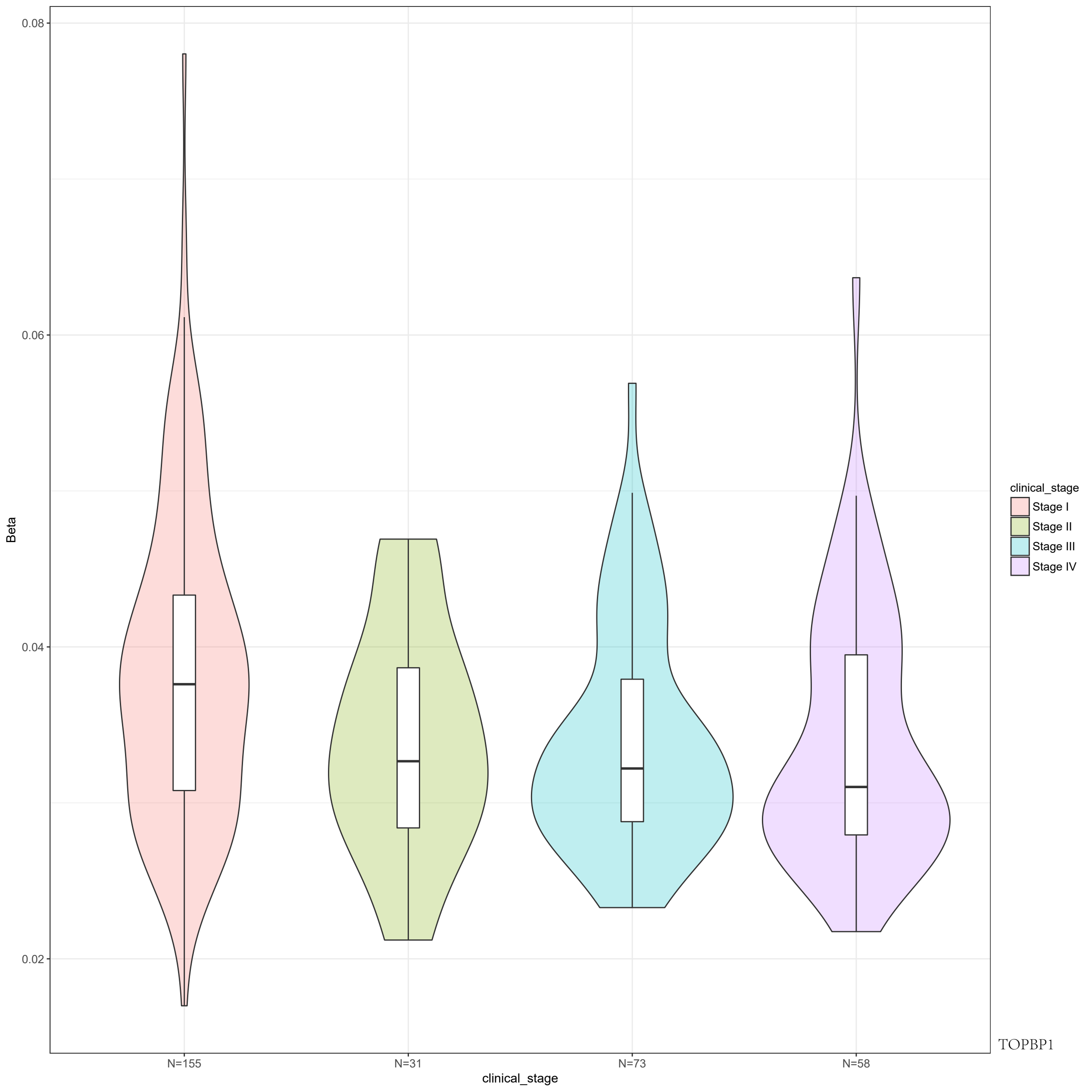

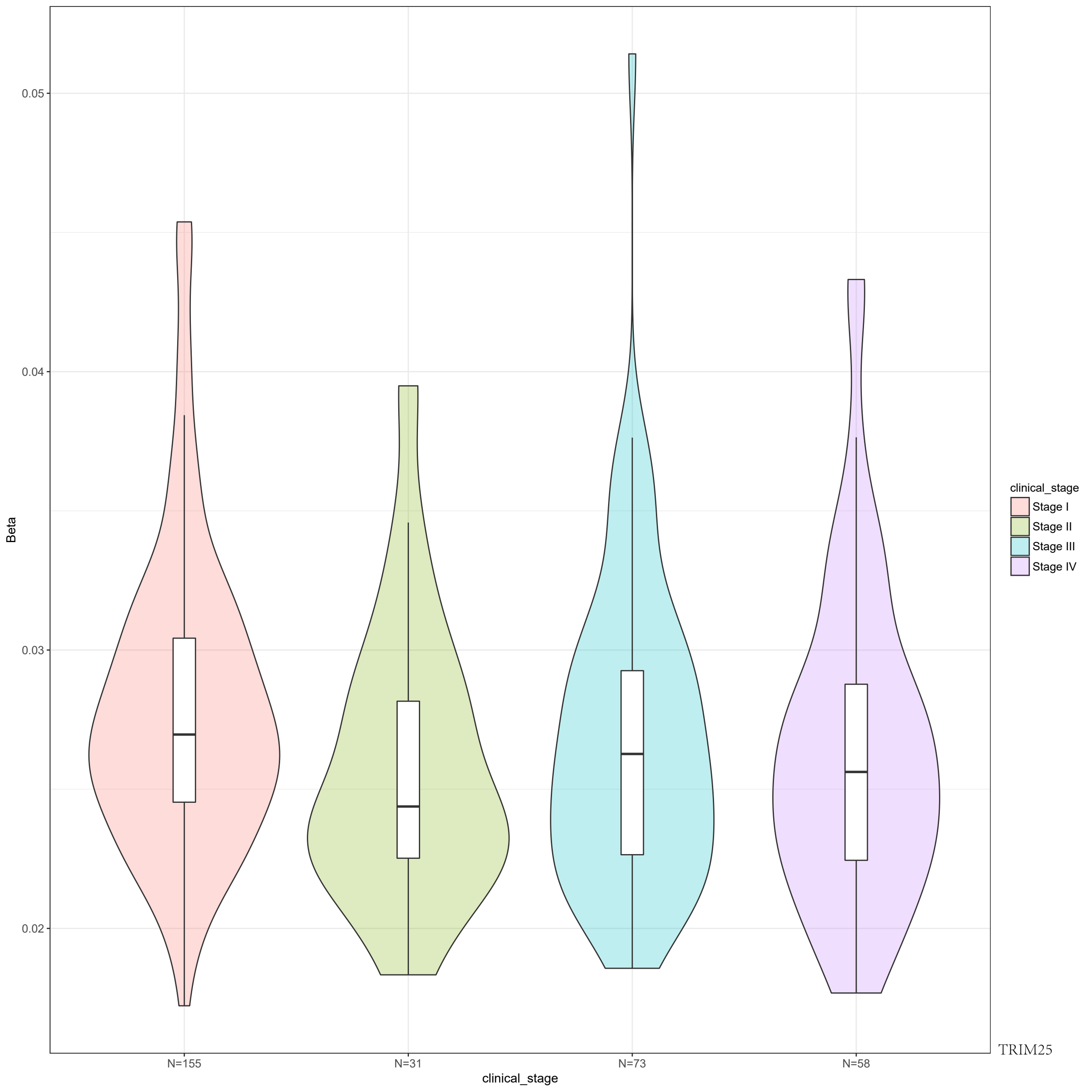

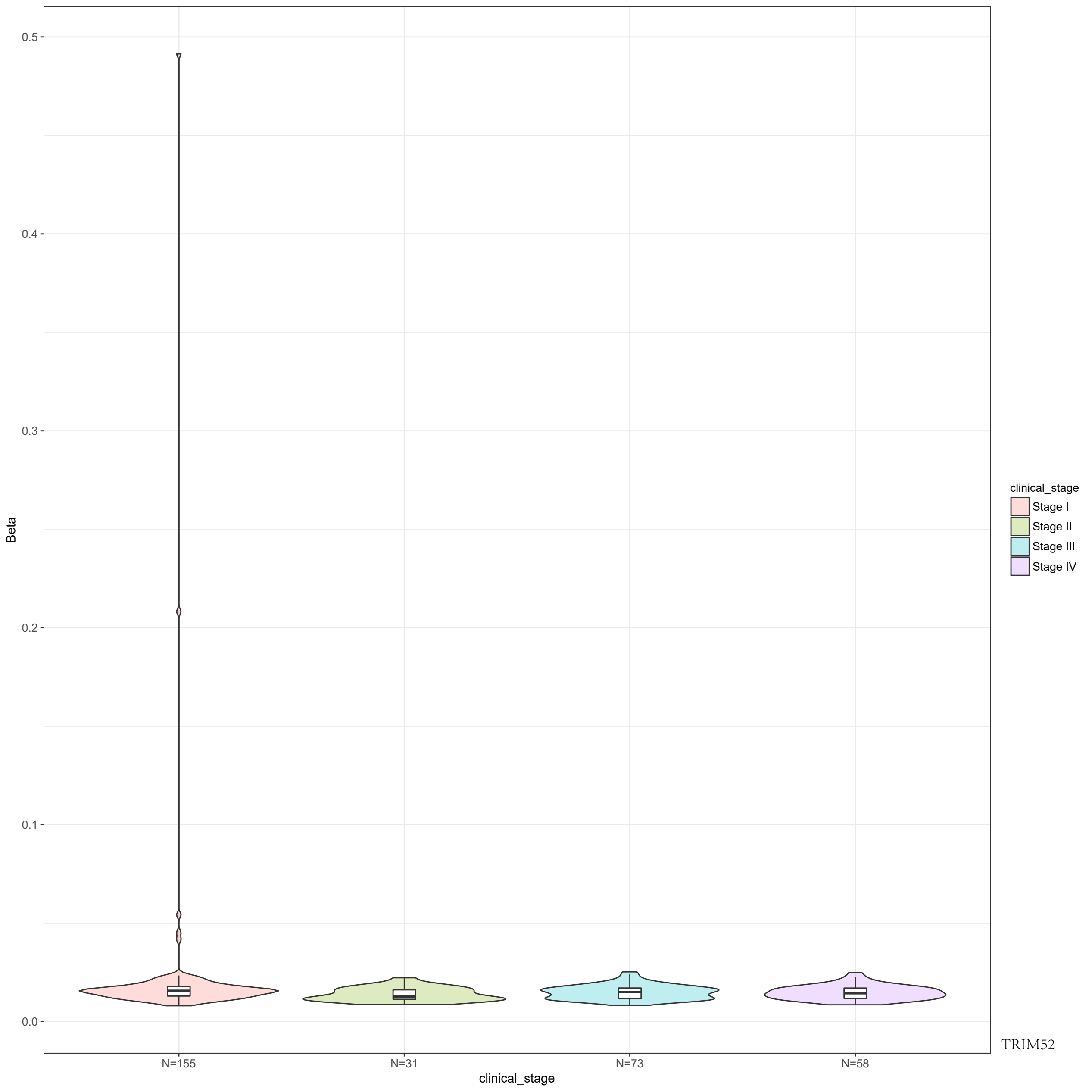

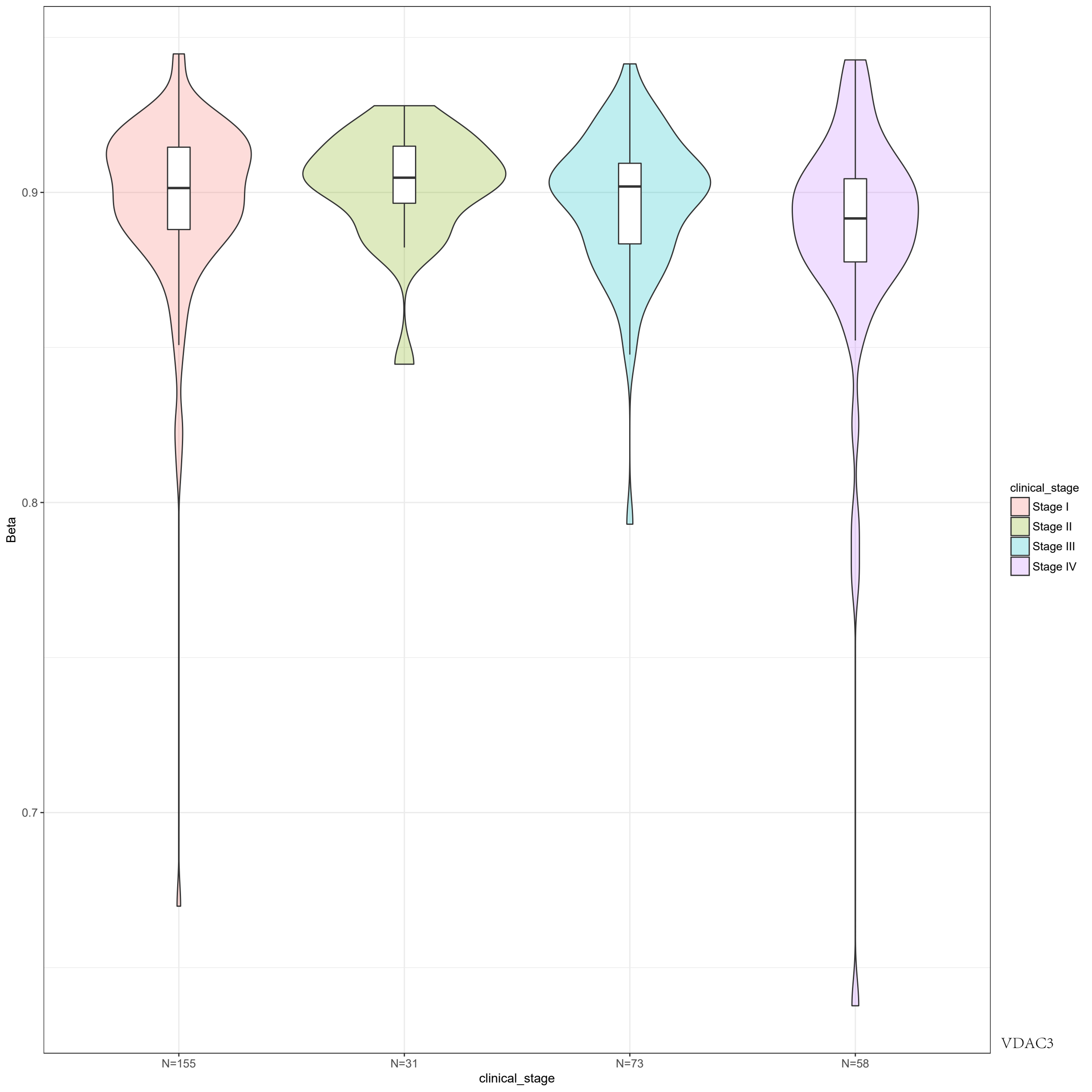

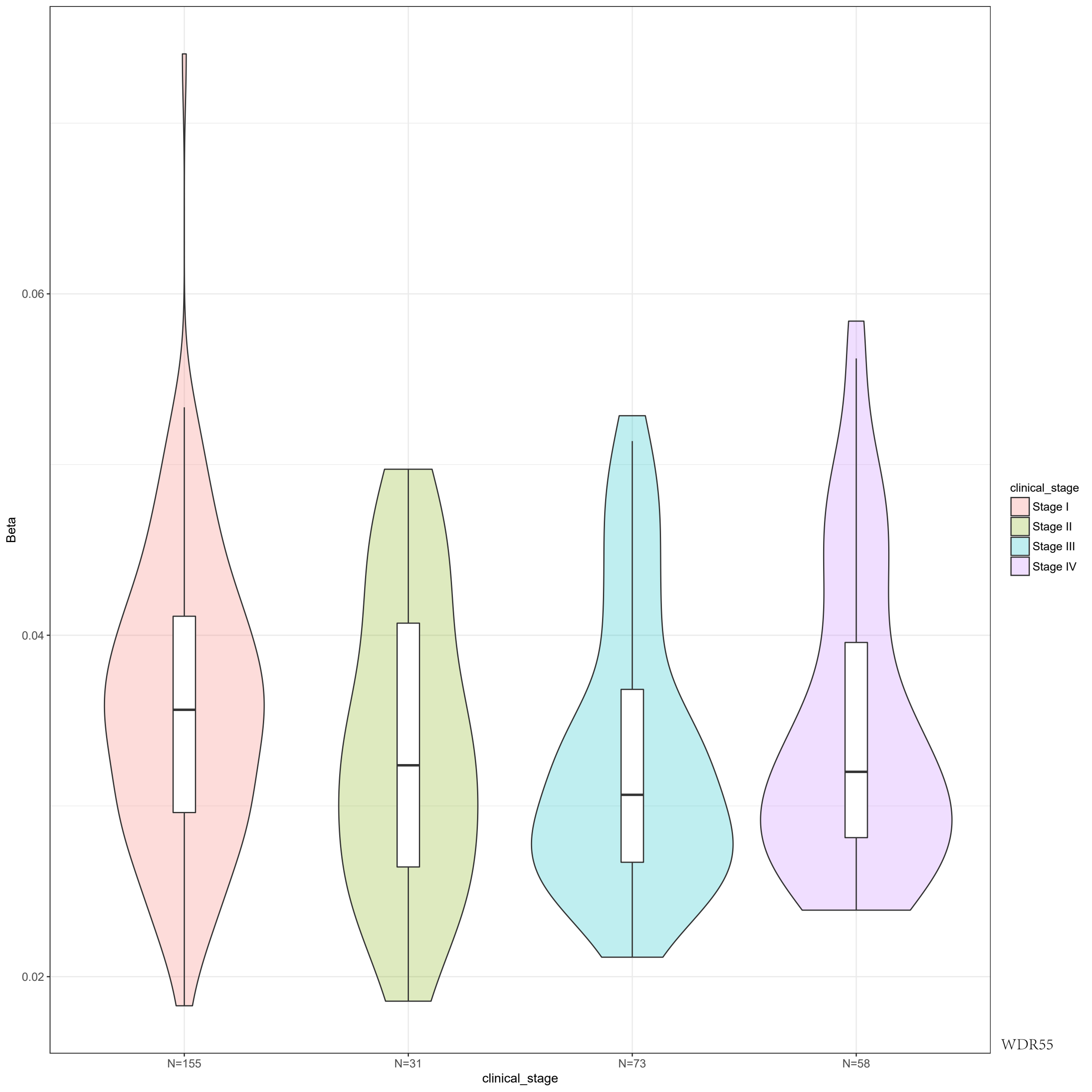

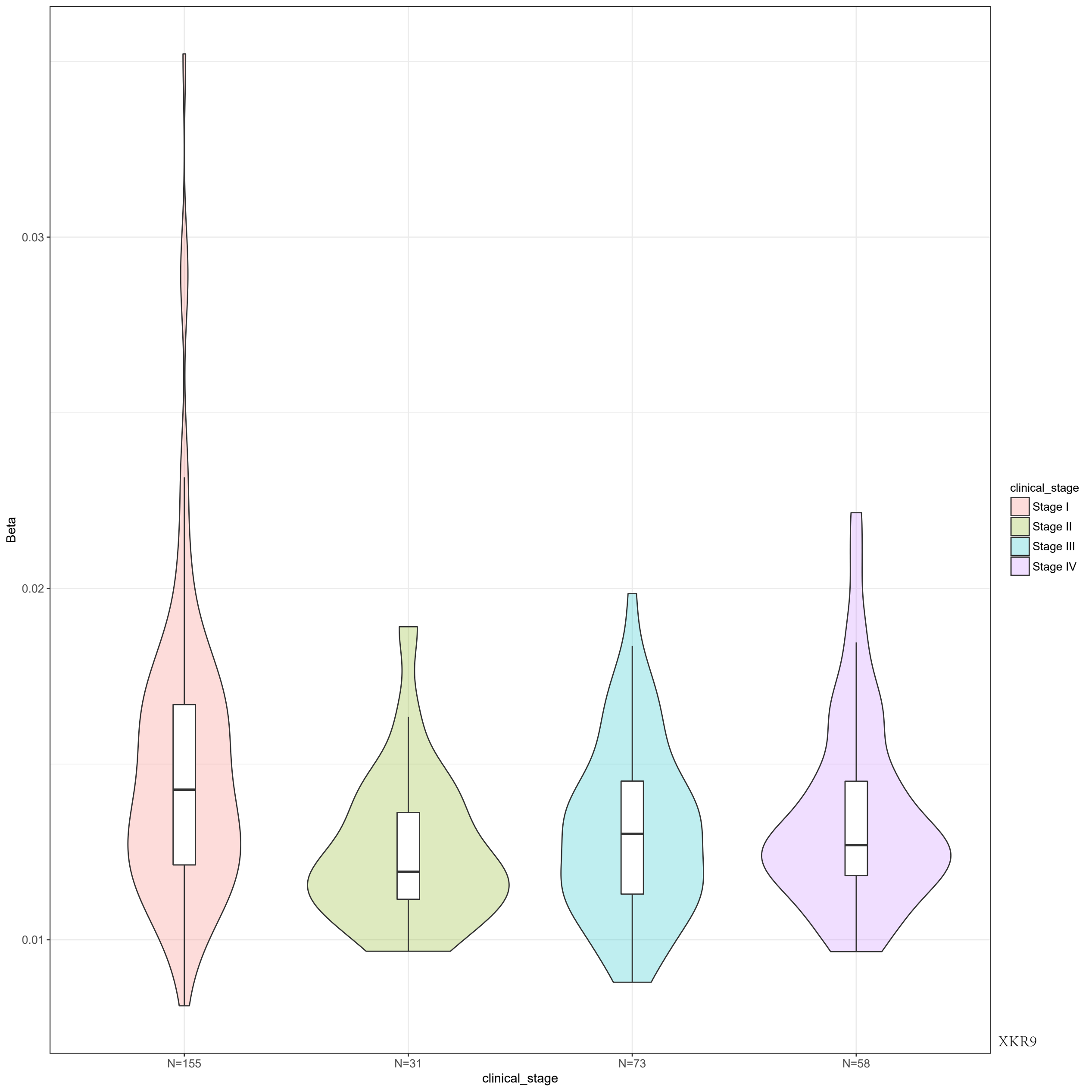

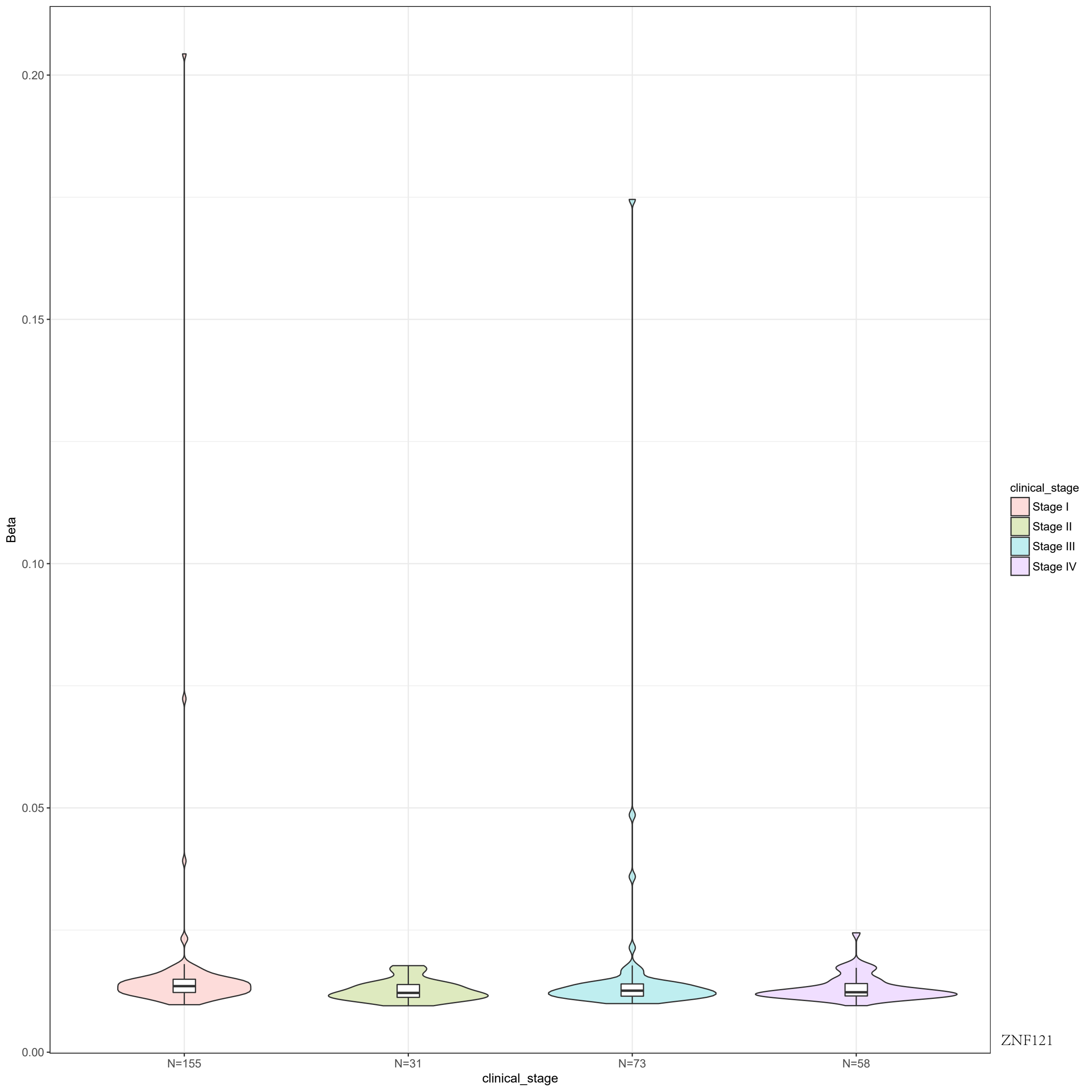

Supplement: Supplementary file 15 [file Data_Sheet_1.PDF]

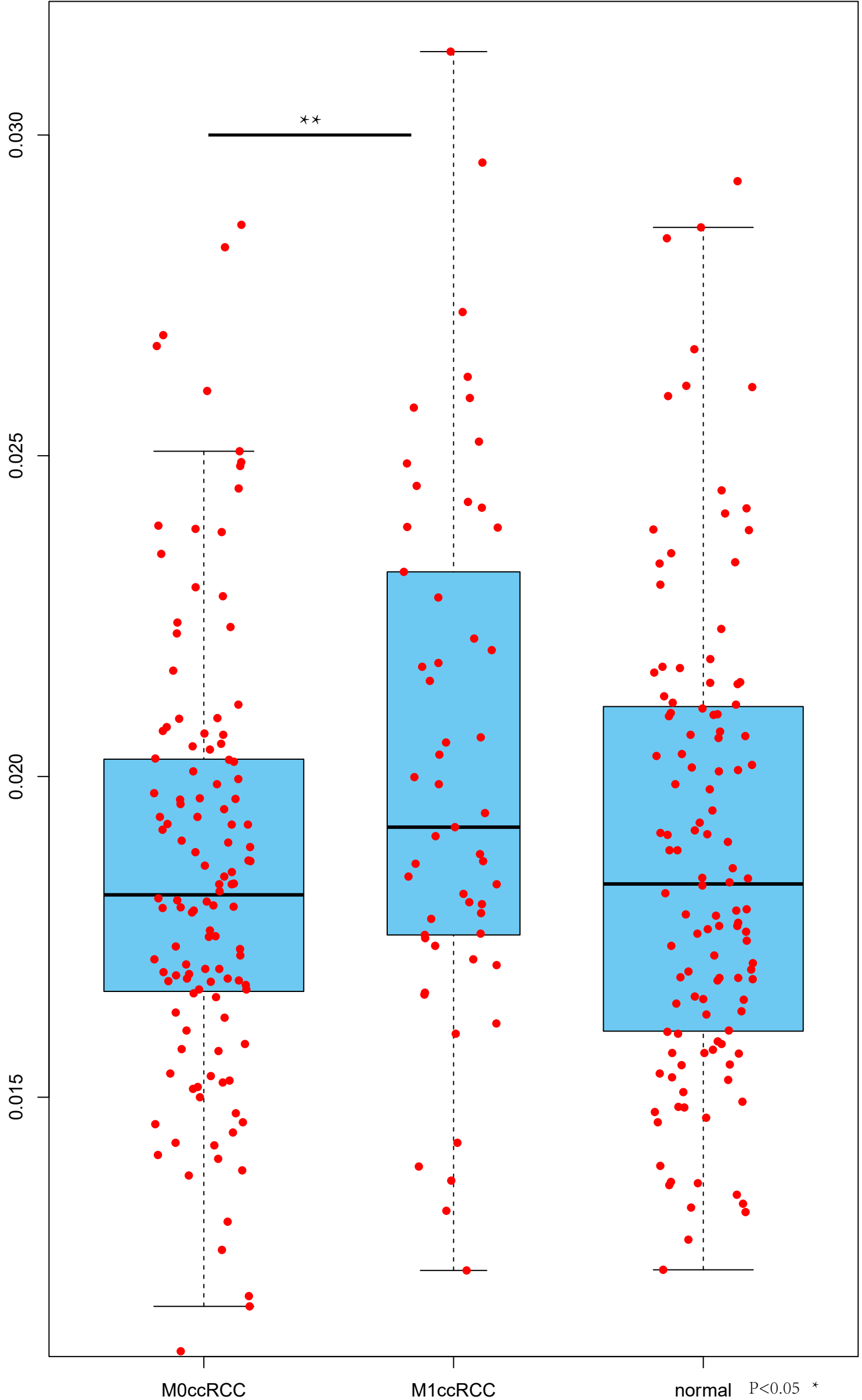

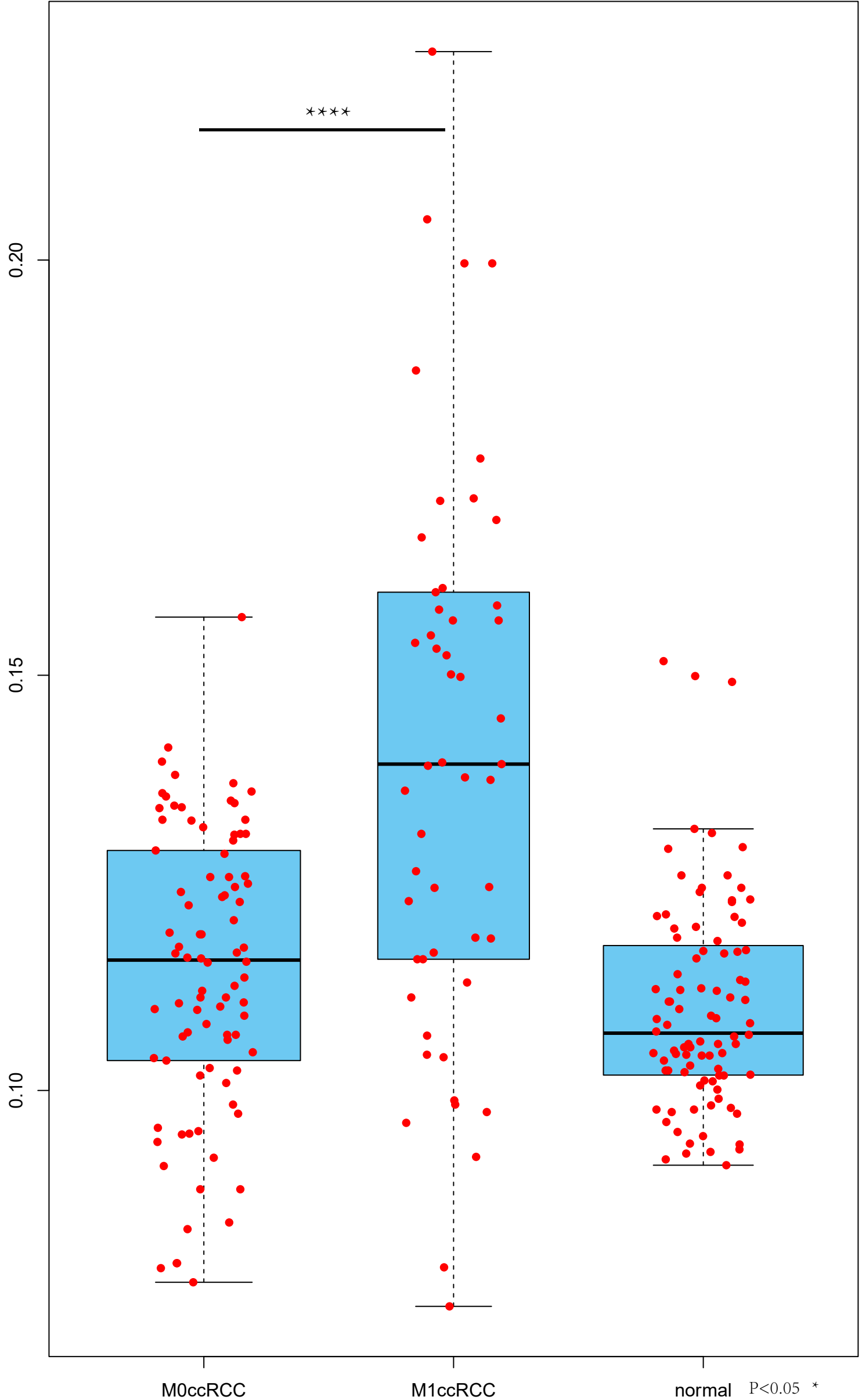

M0ccRCC

M1ccRCC

normal

P<0.05 \*  
P<0.01 \*\*  
P<0.001 \*\*\*  
P<0.0001 \*\*\*\*

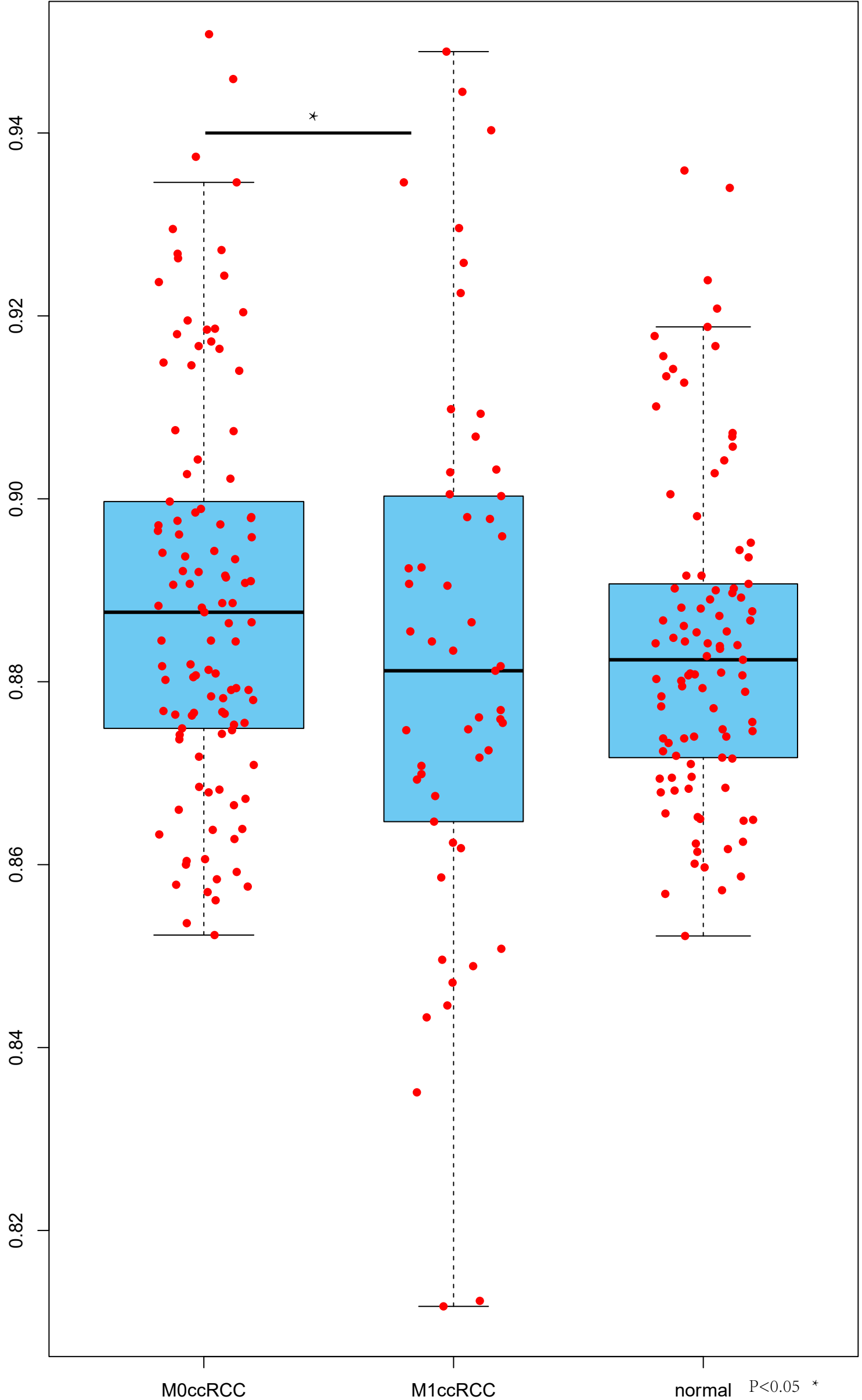

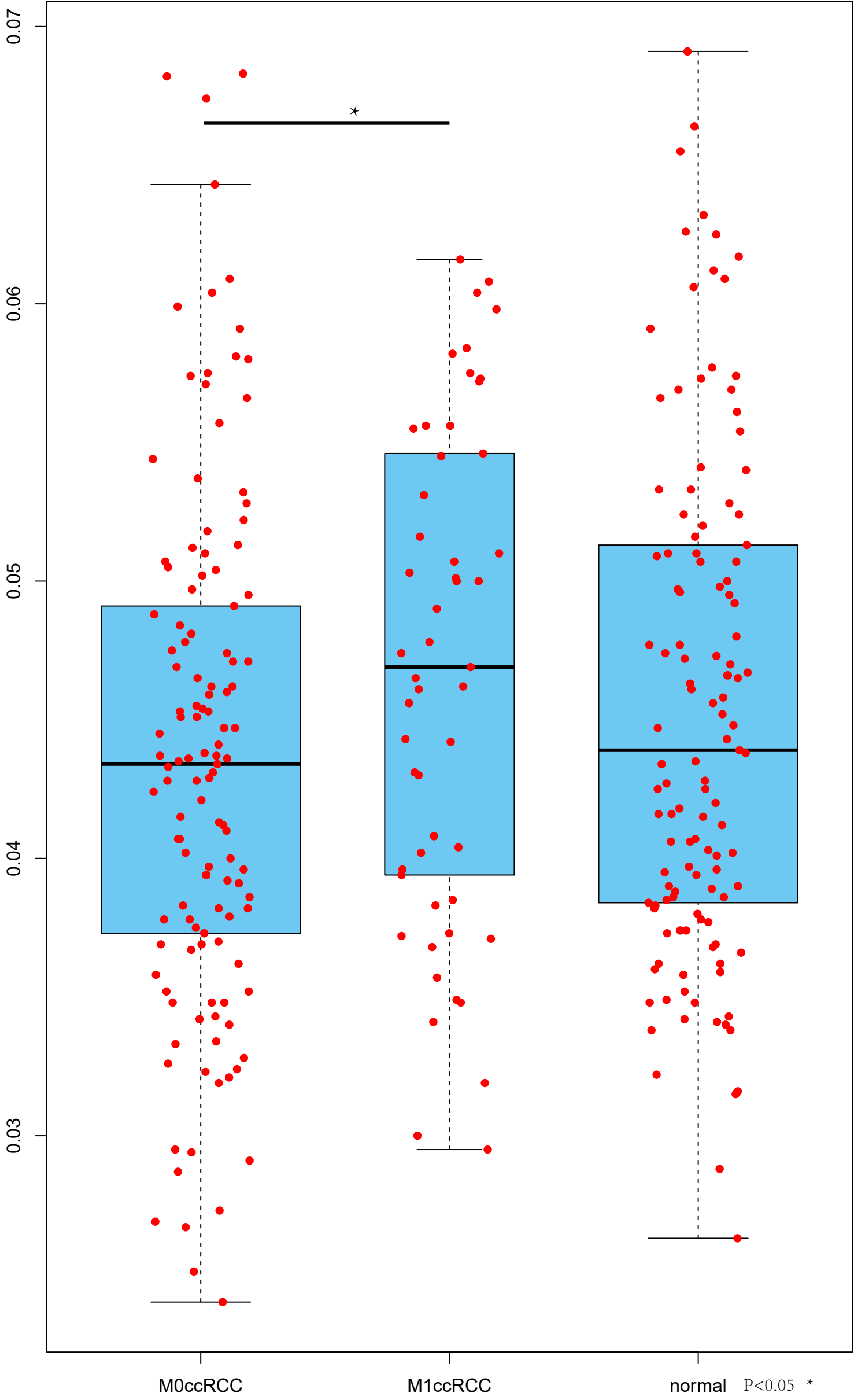

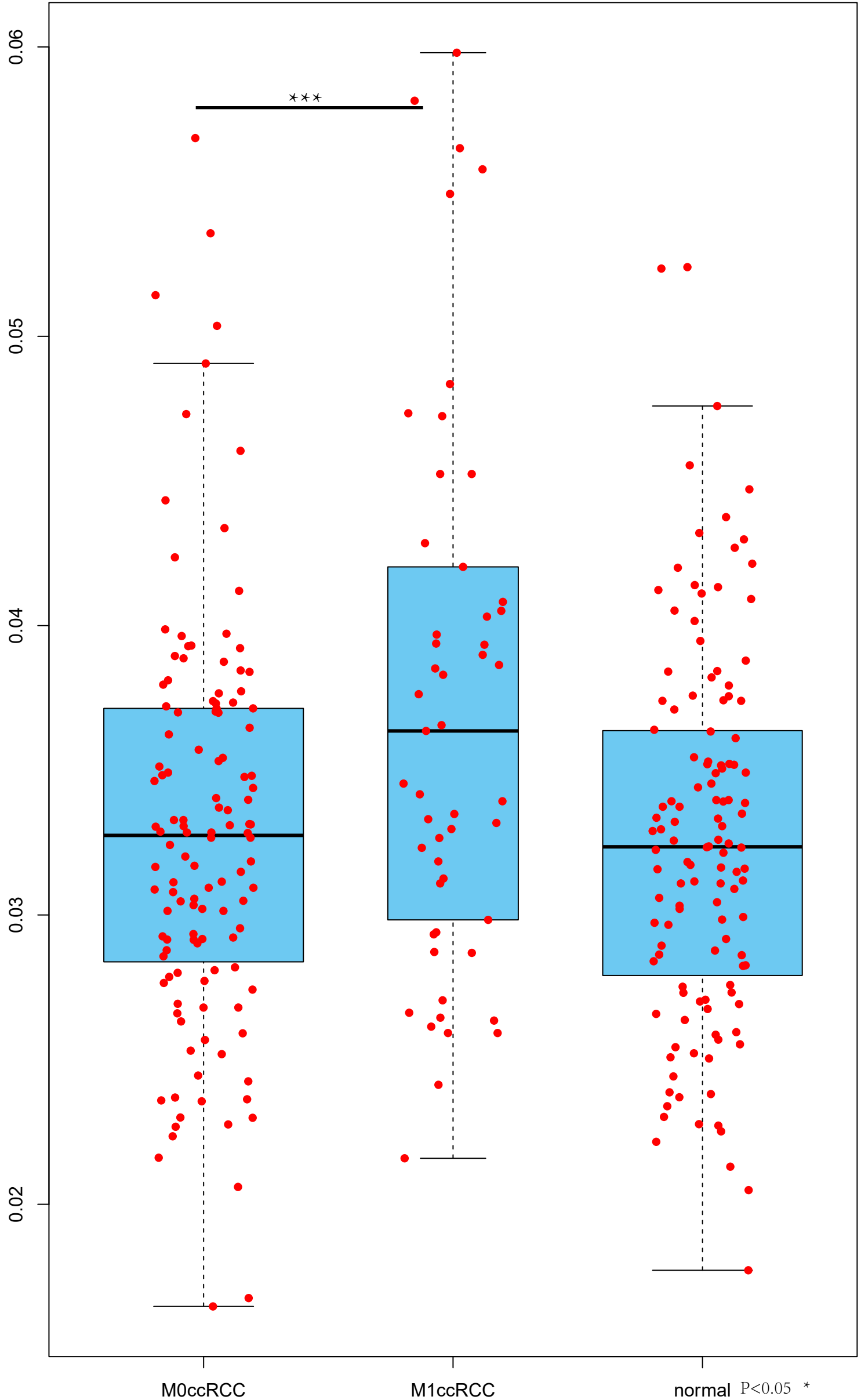

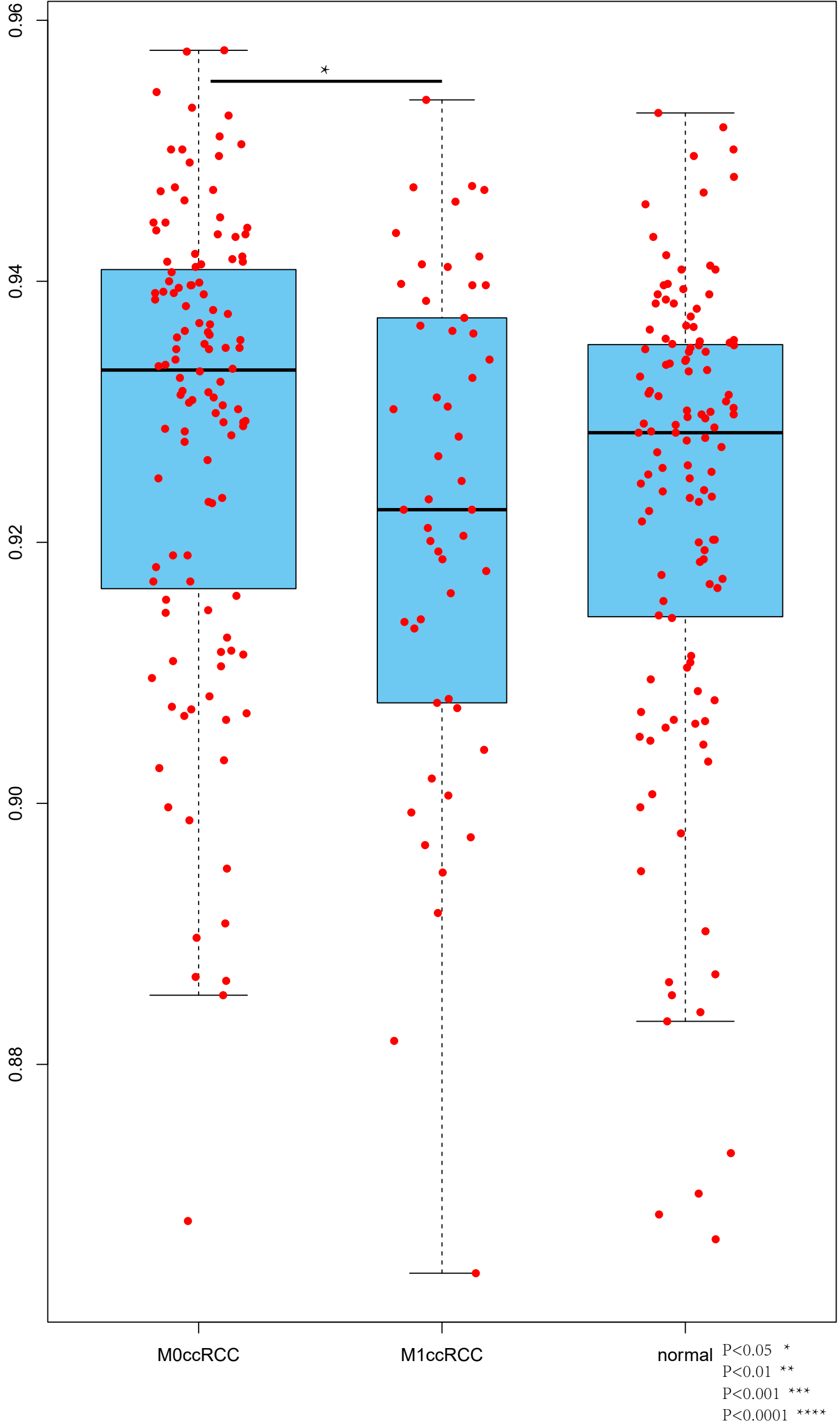

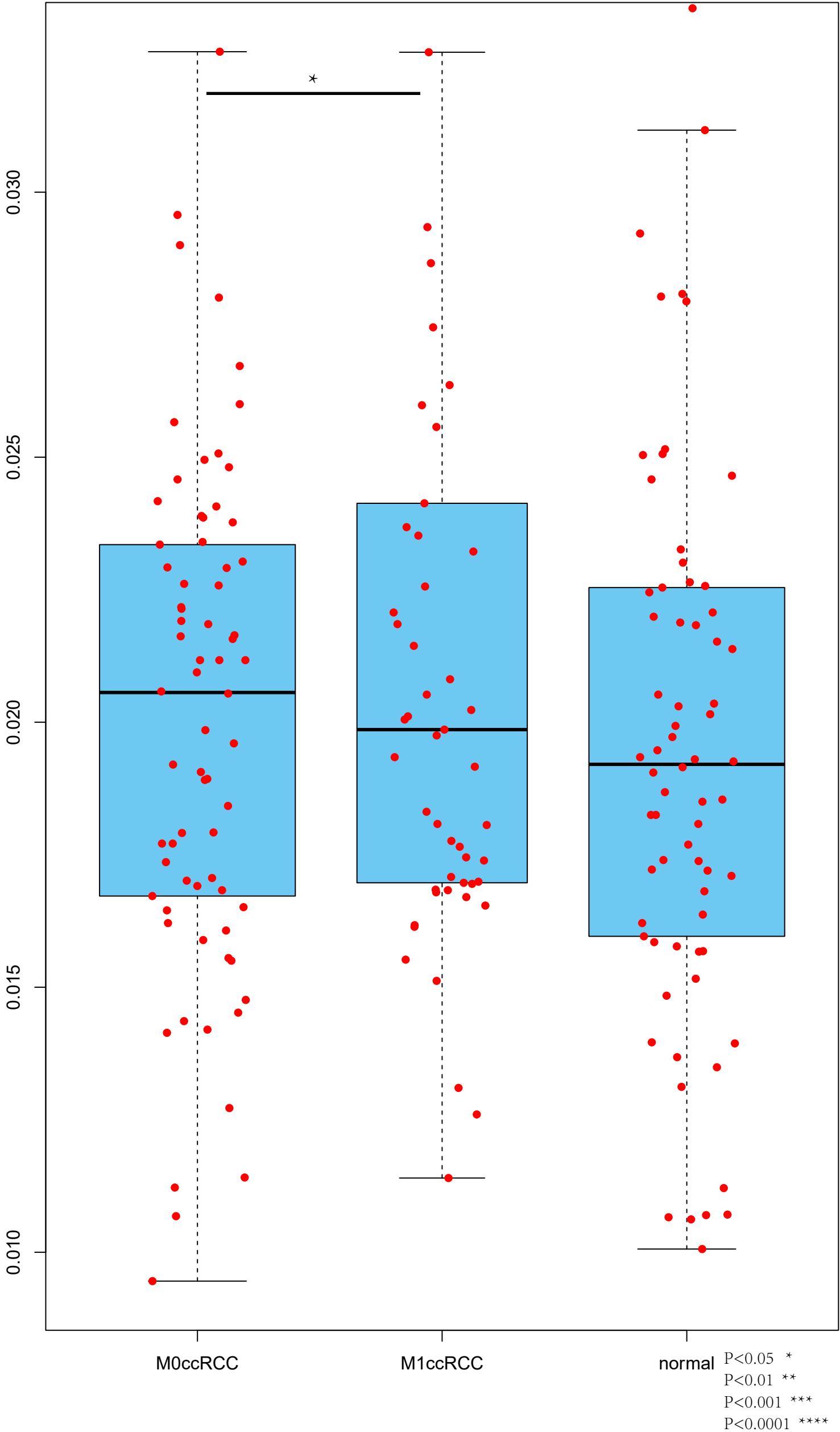

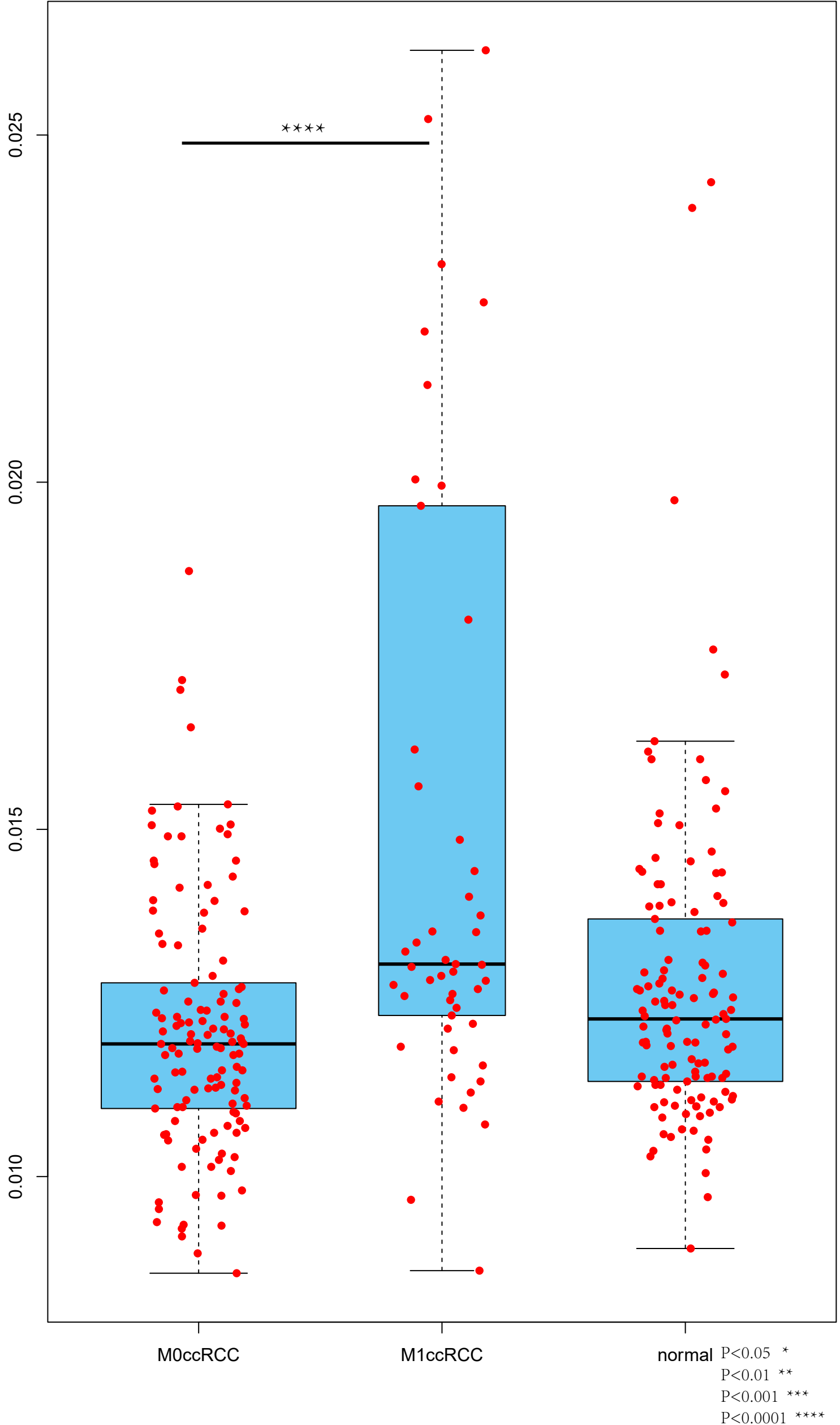

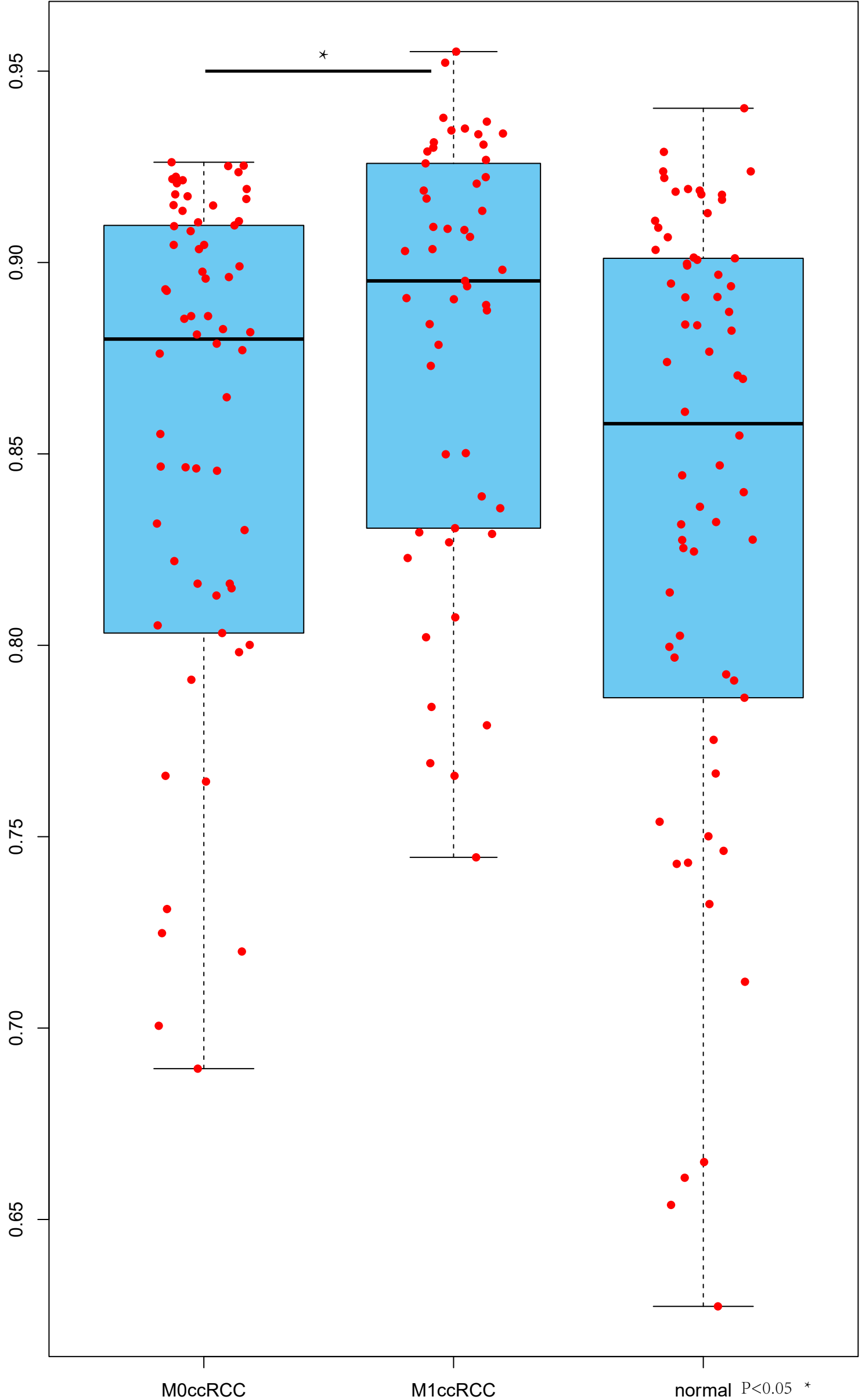

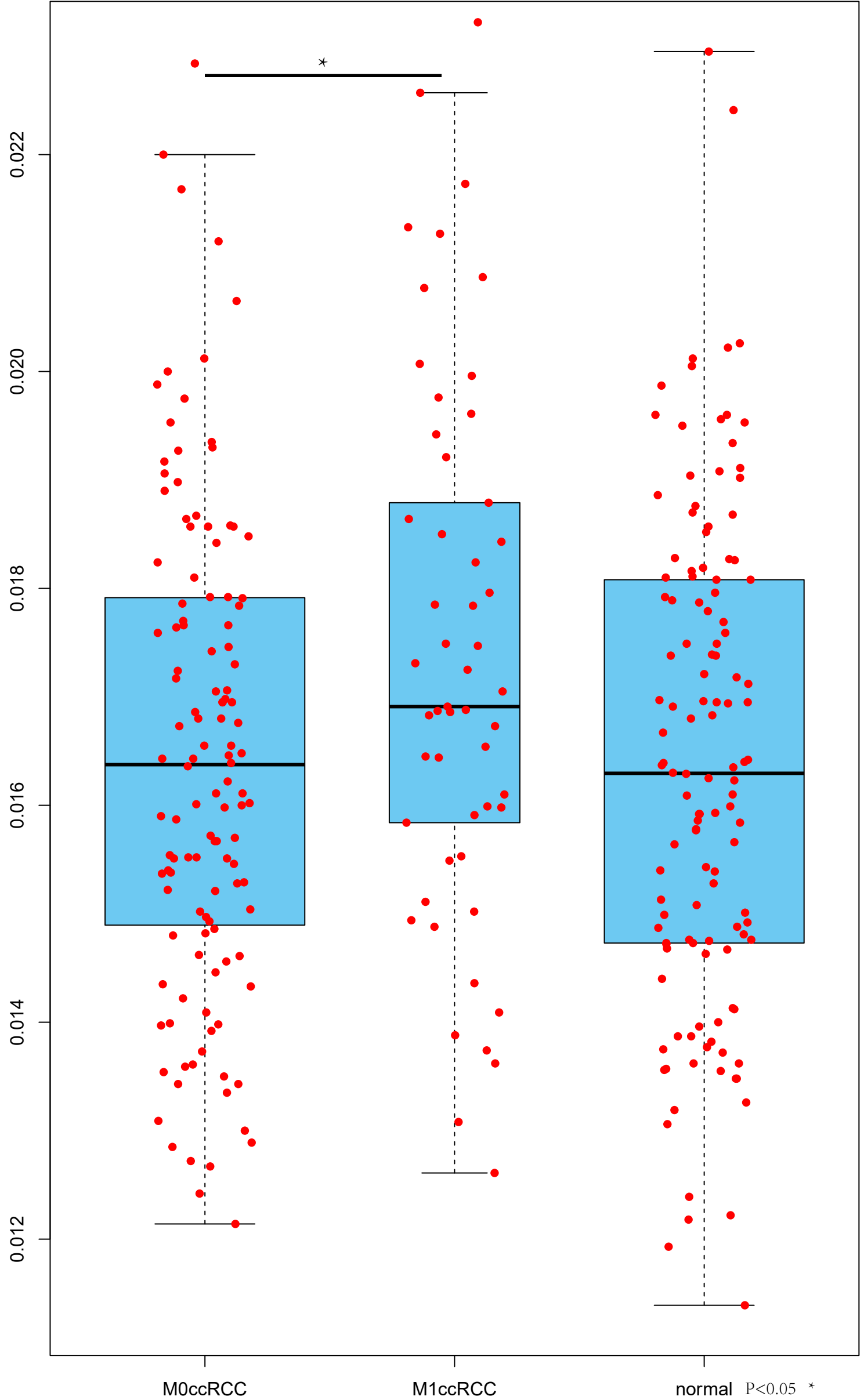

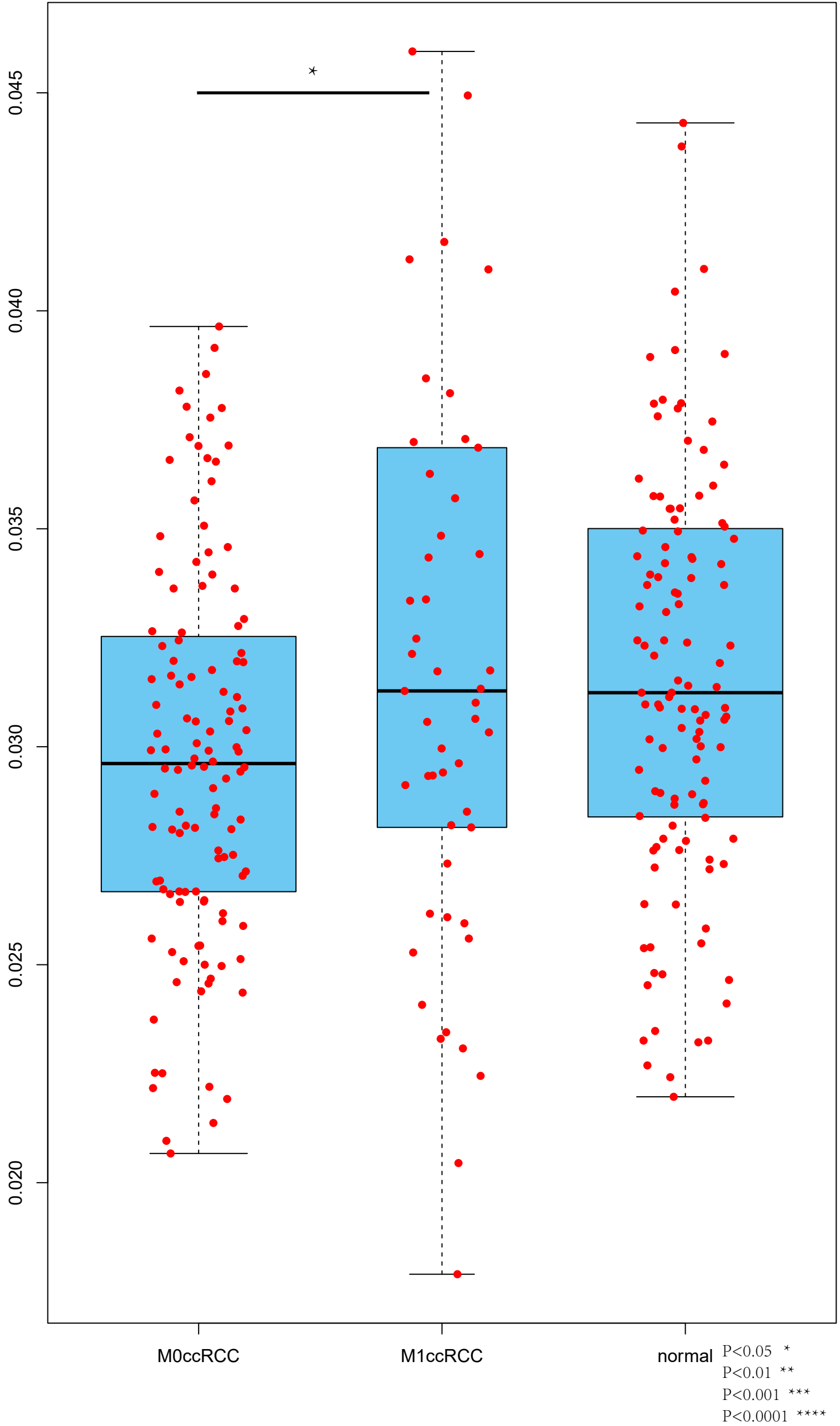

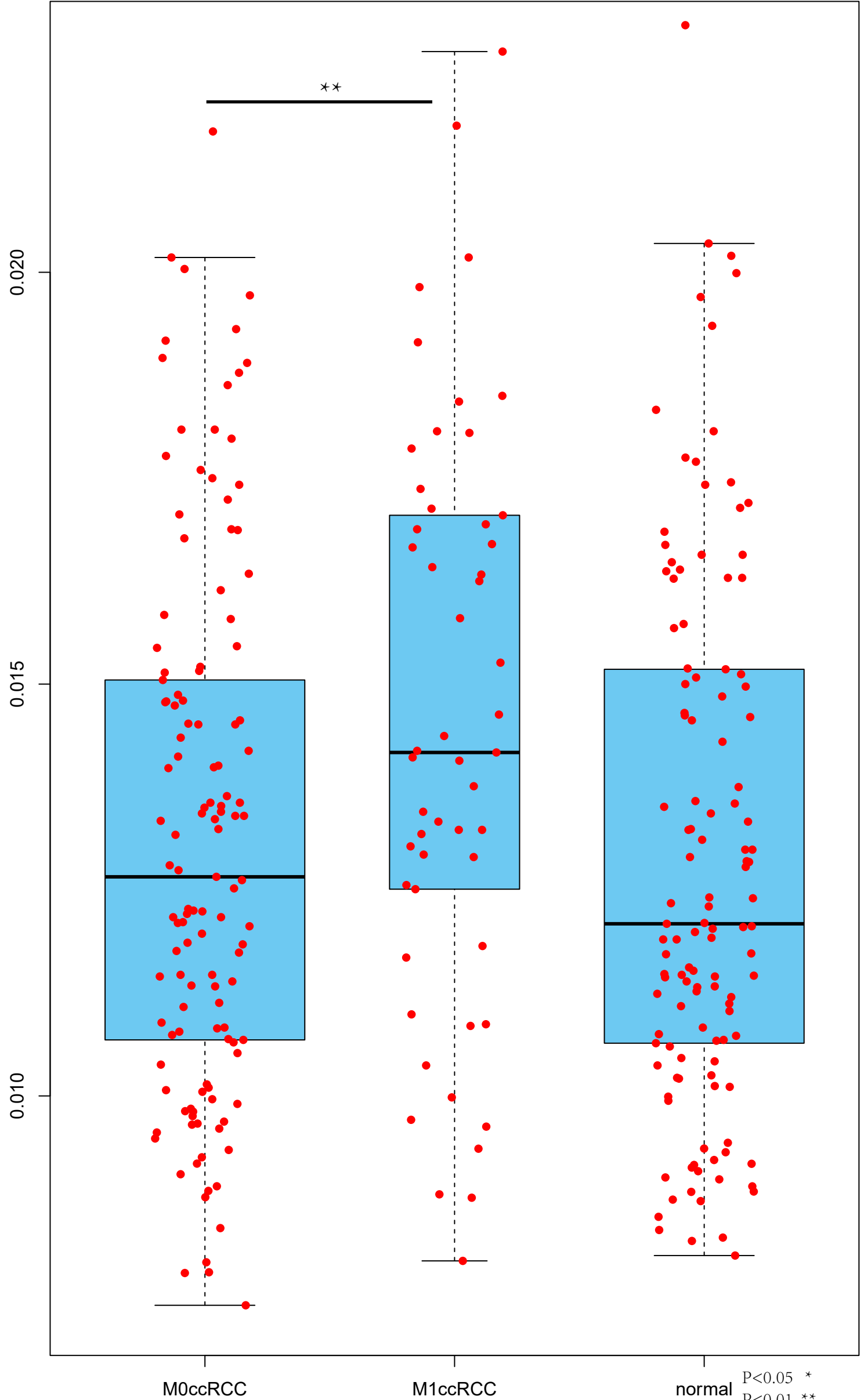

Supplement: Supplementary file 17 [file Data_Sheet_3.PDF]

C4orf3

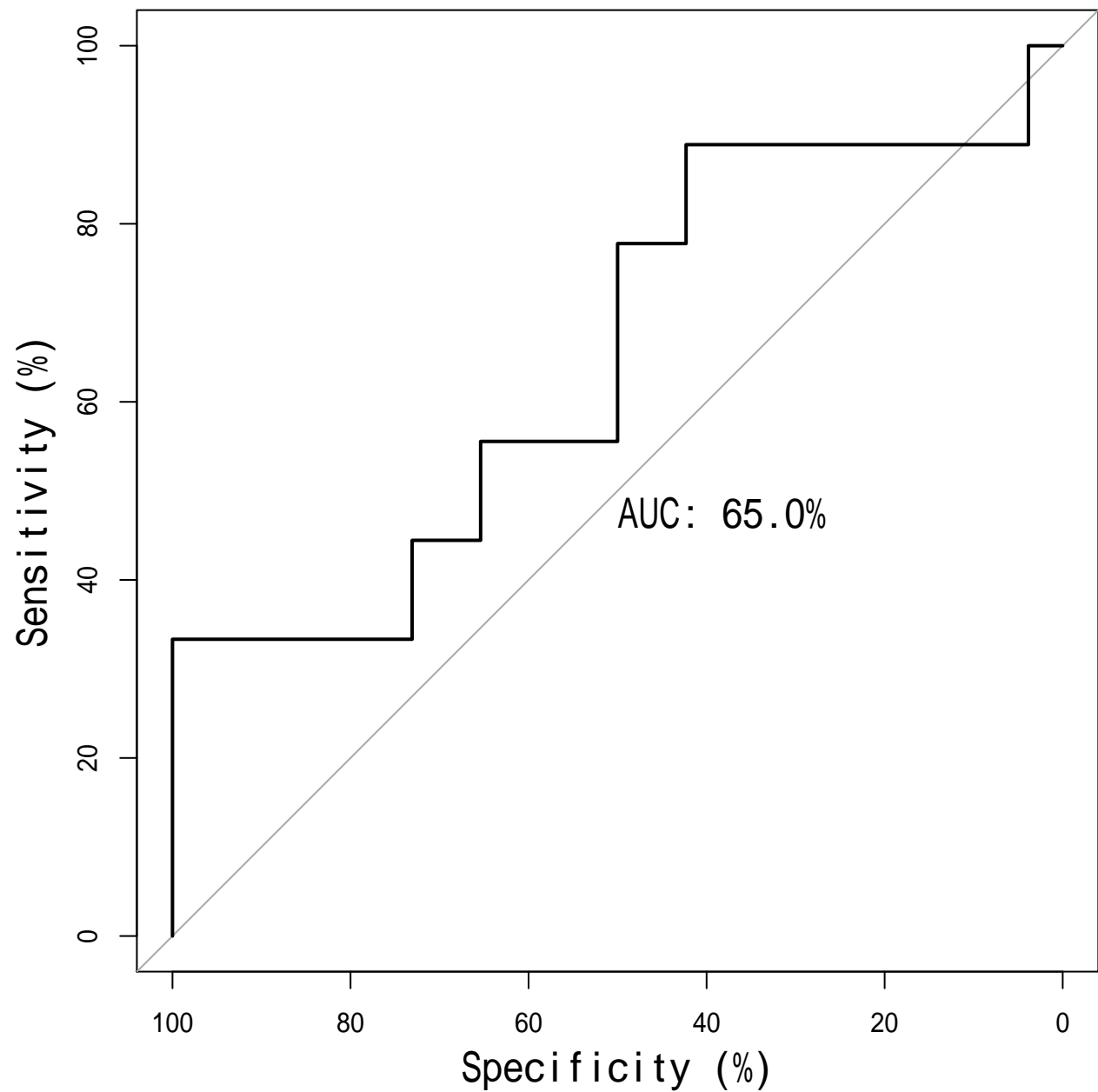

C12orf51

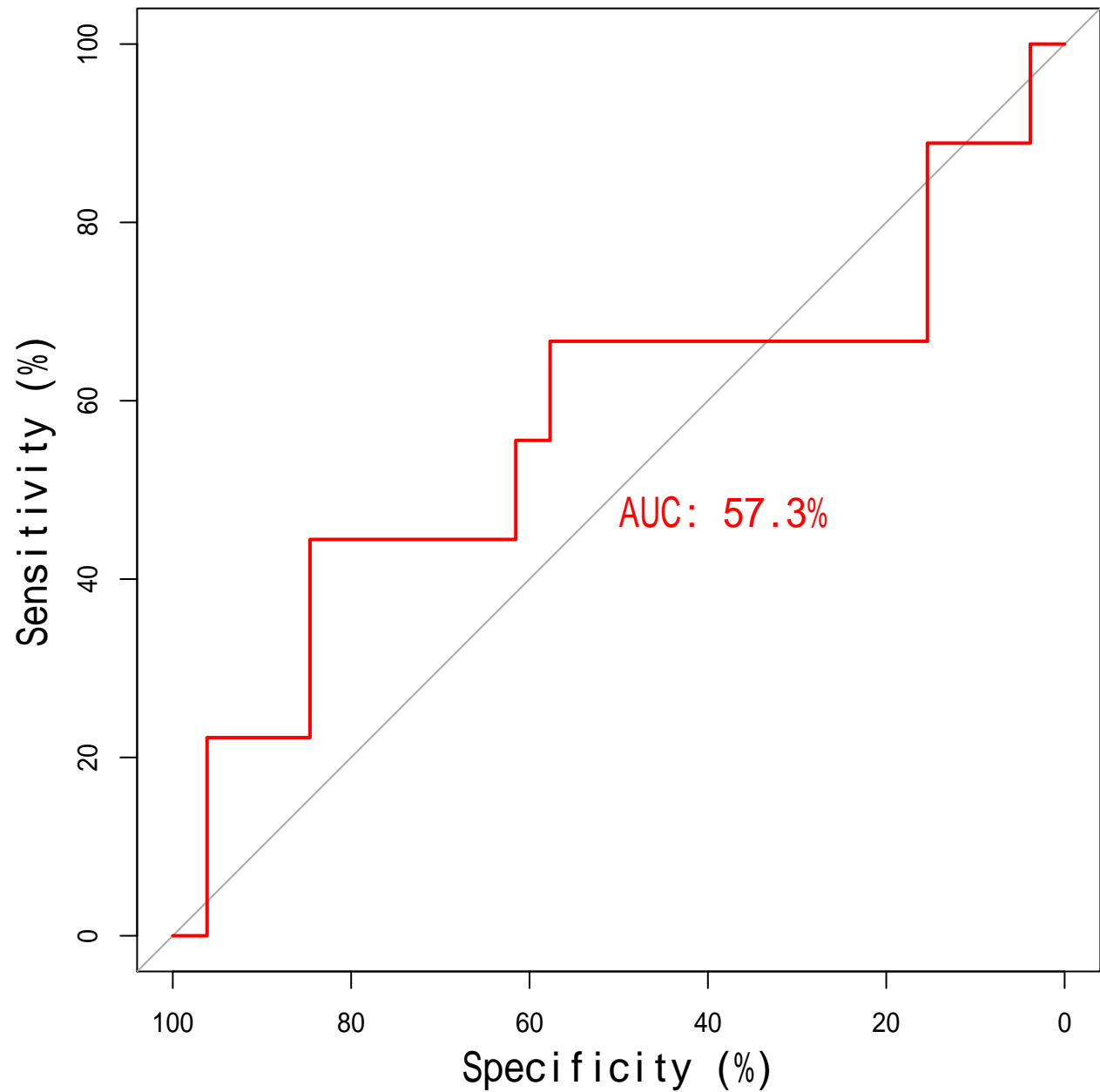

C17orf65

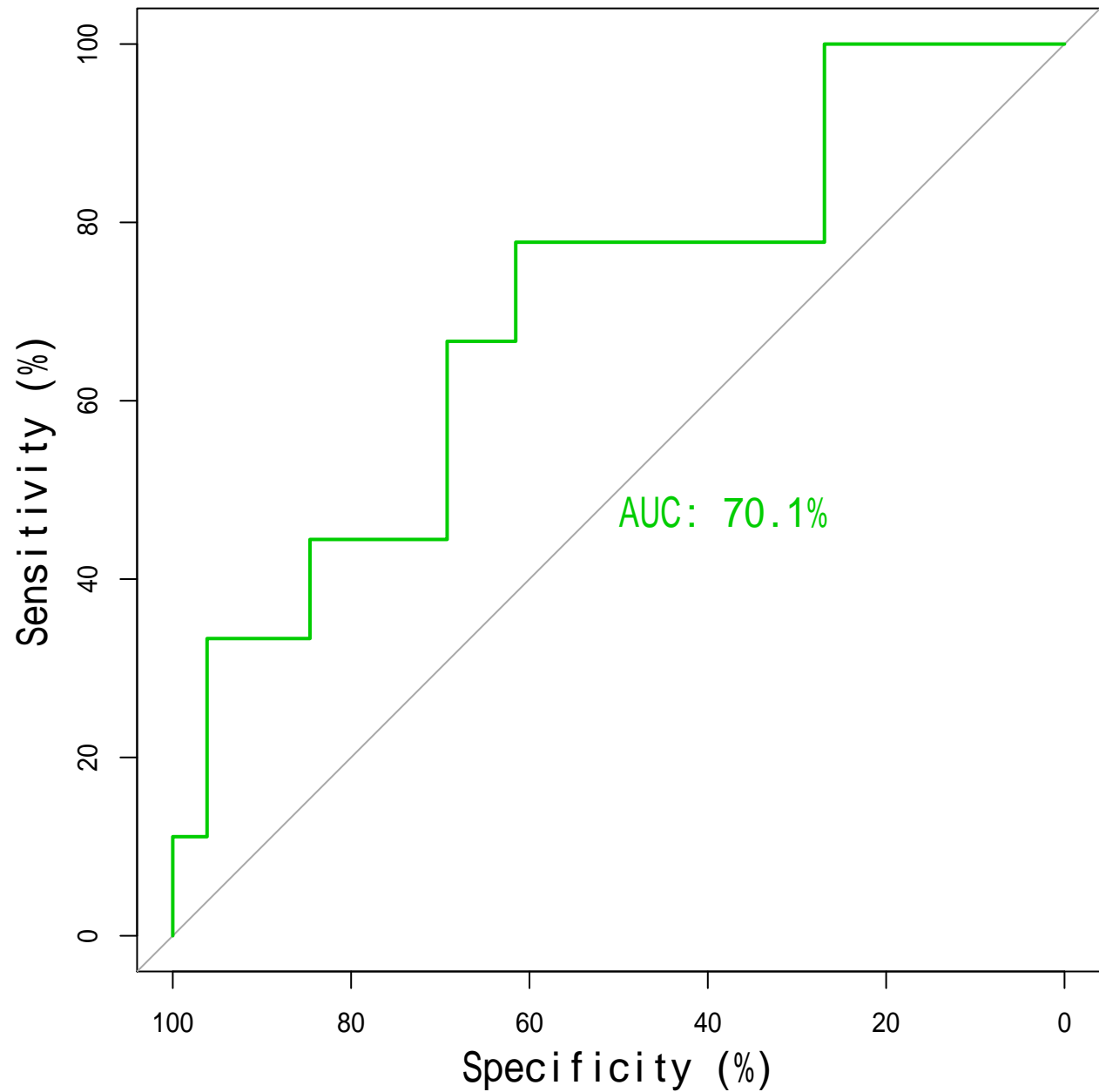

C21orf45

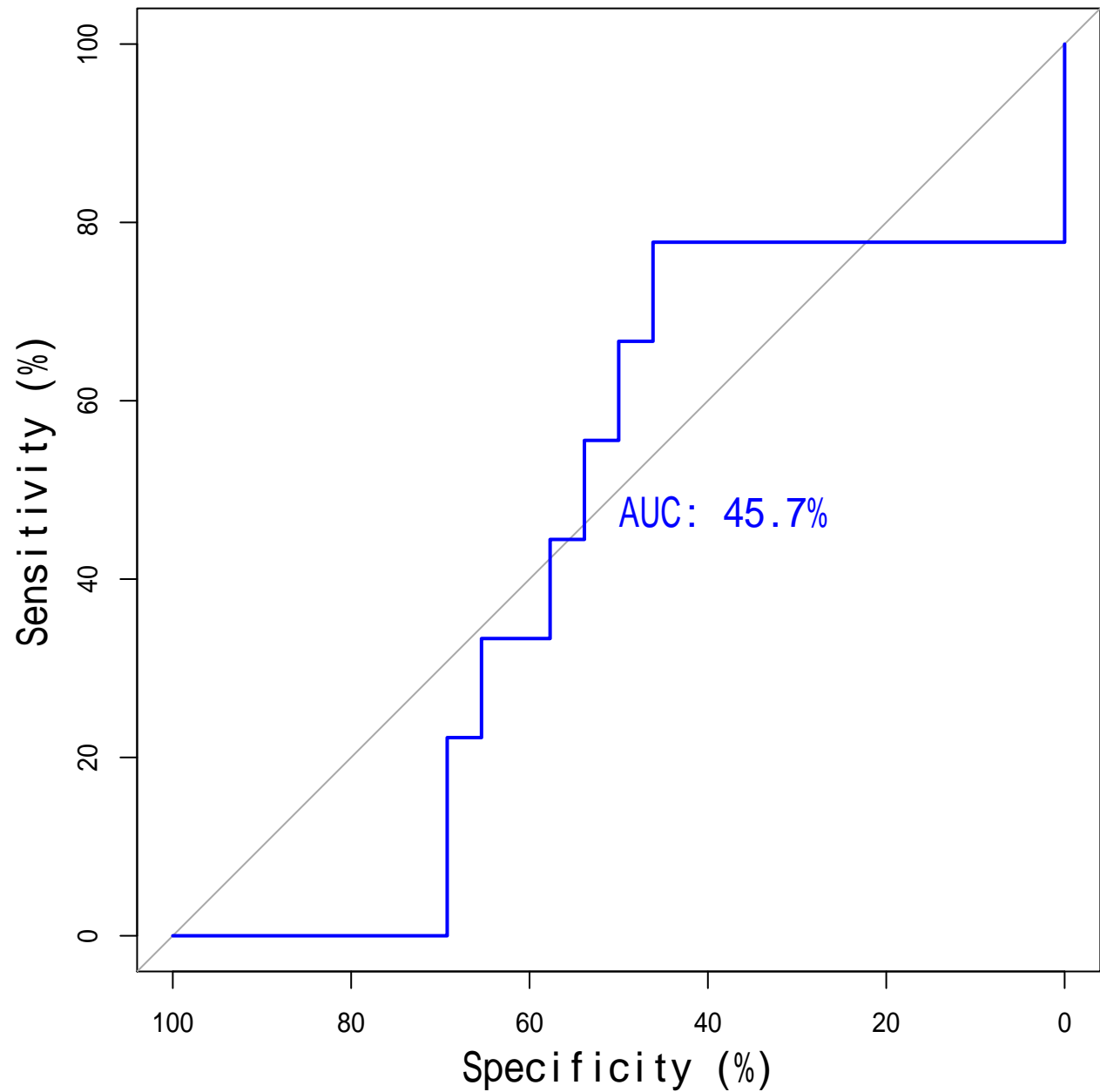

CETN3

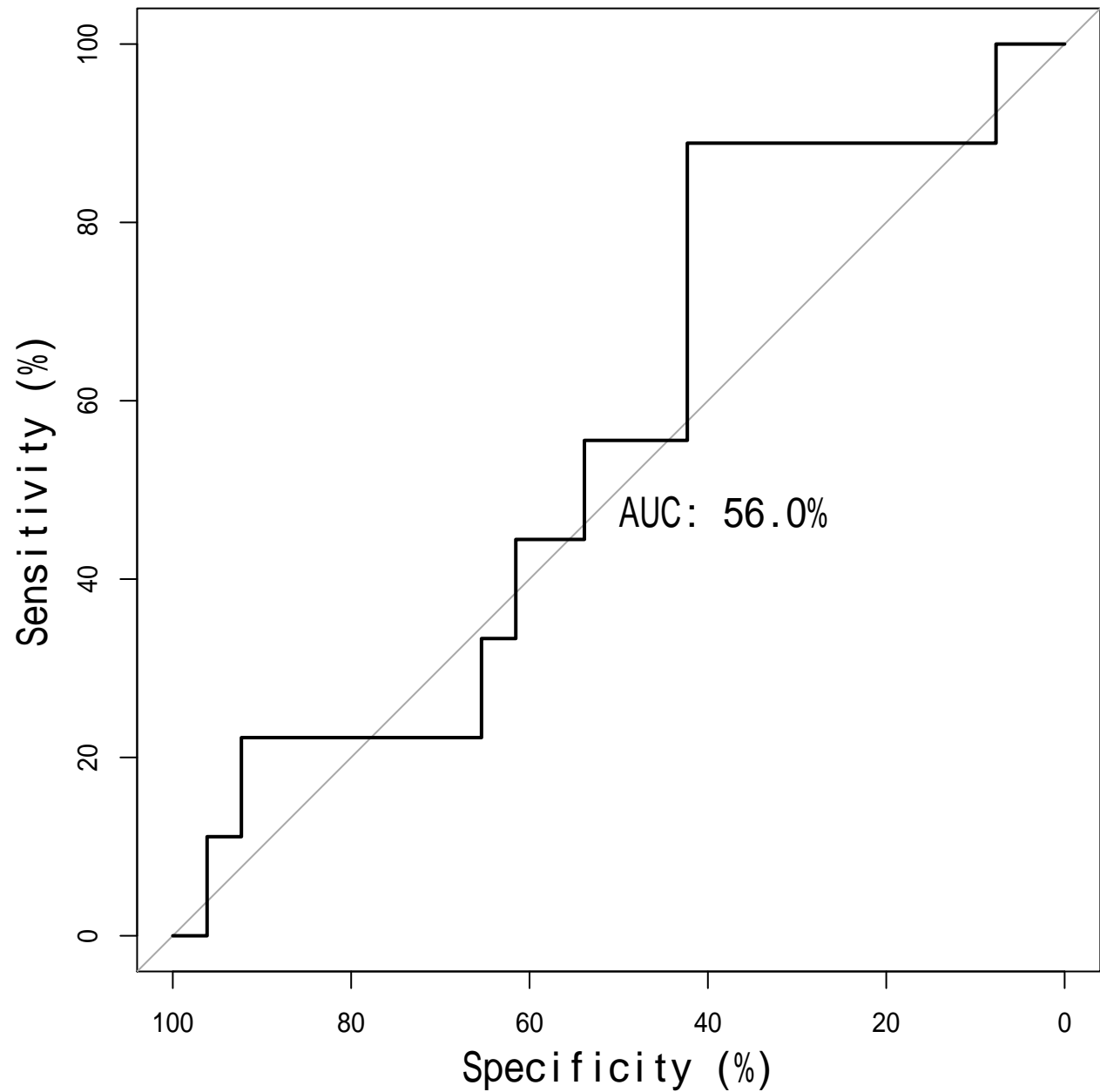

DCAF7

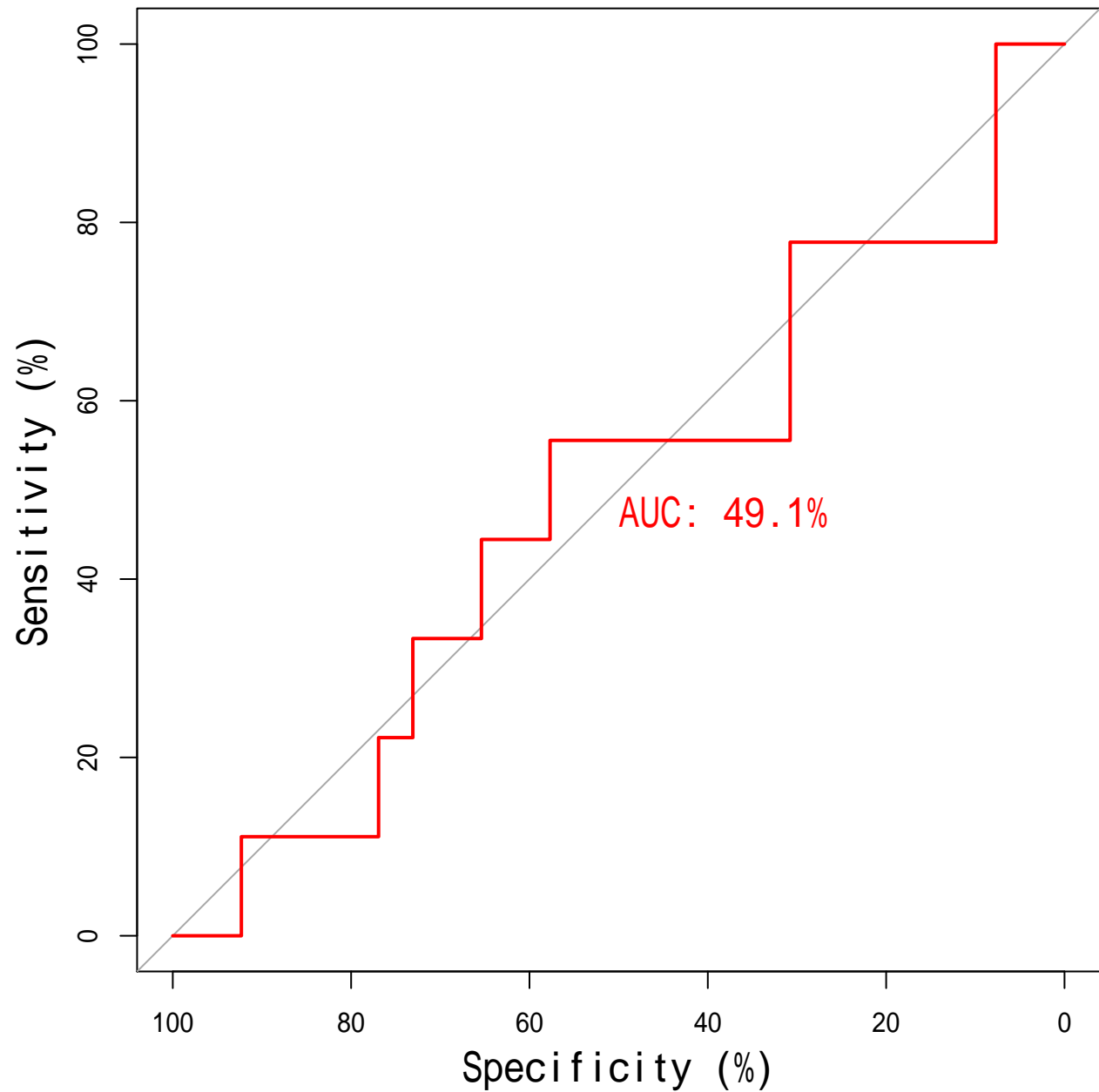

GPX4

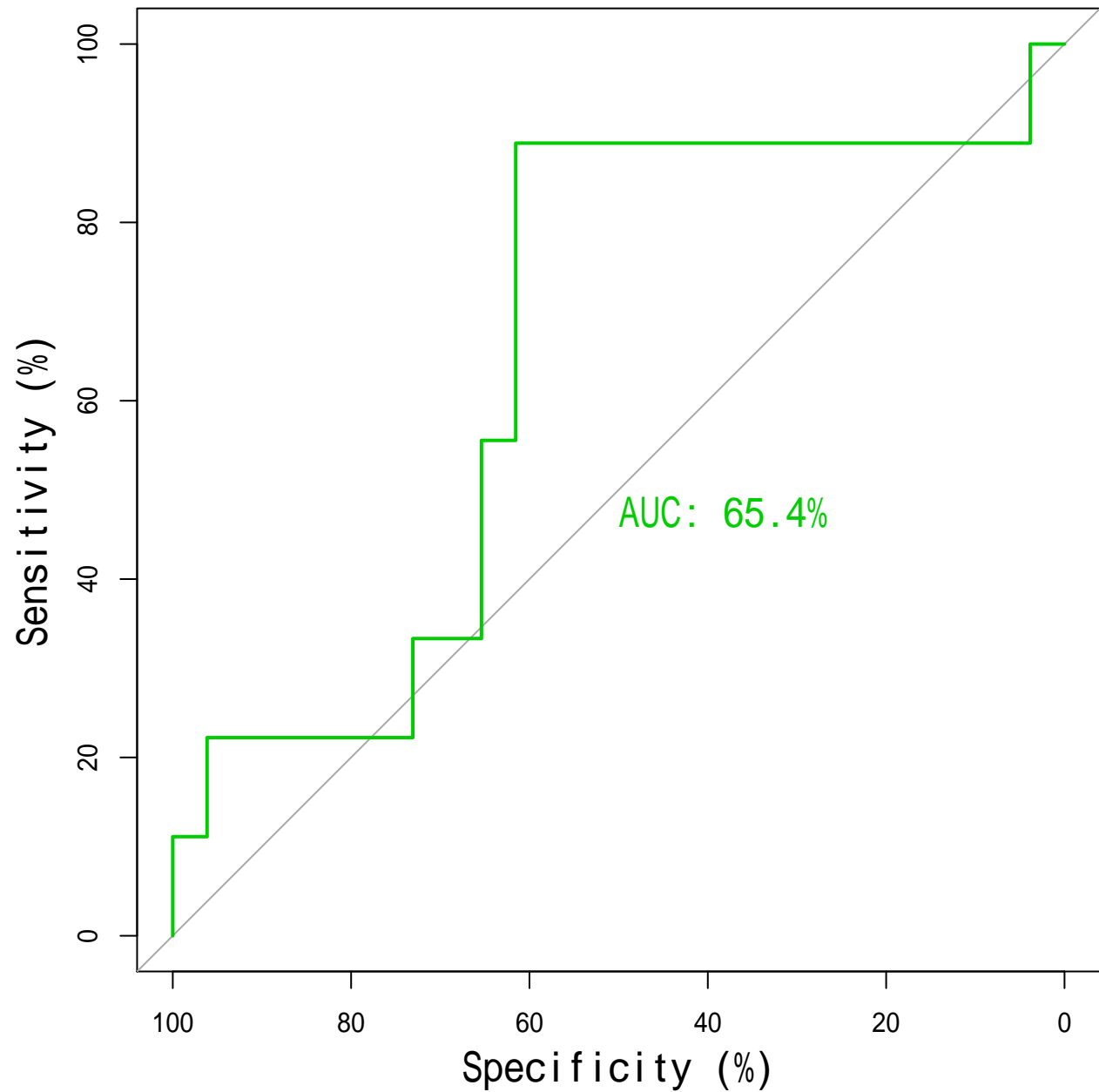

HNRNPA0

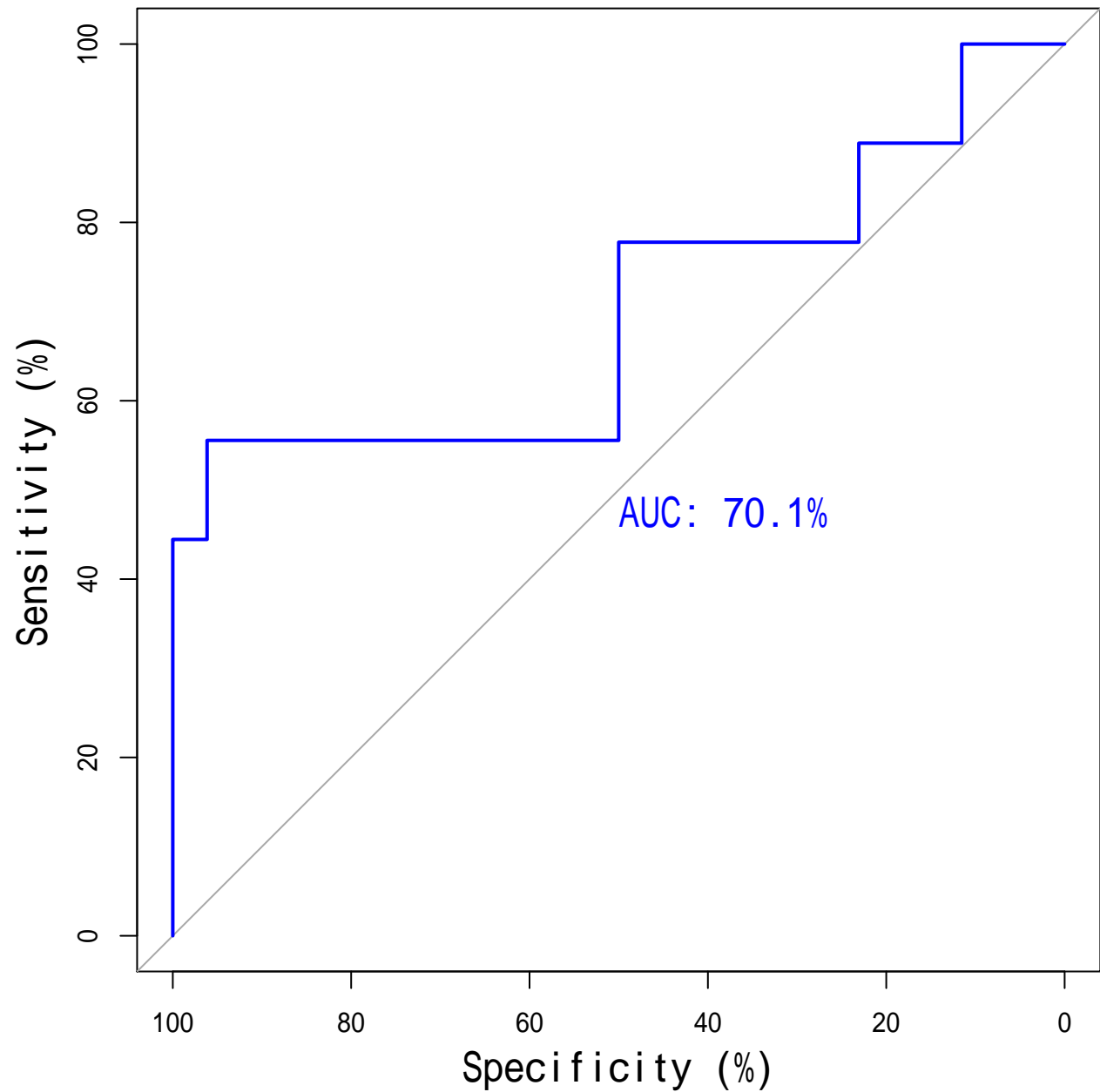

NUP54

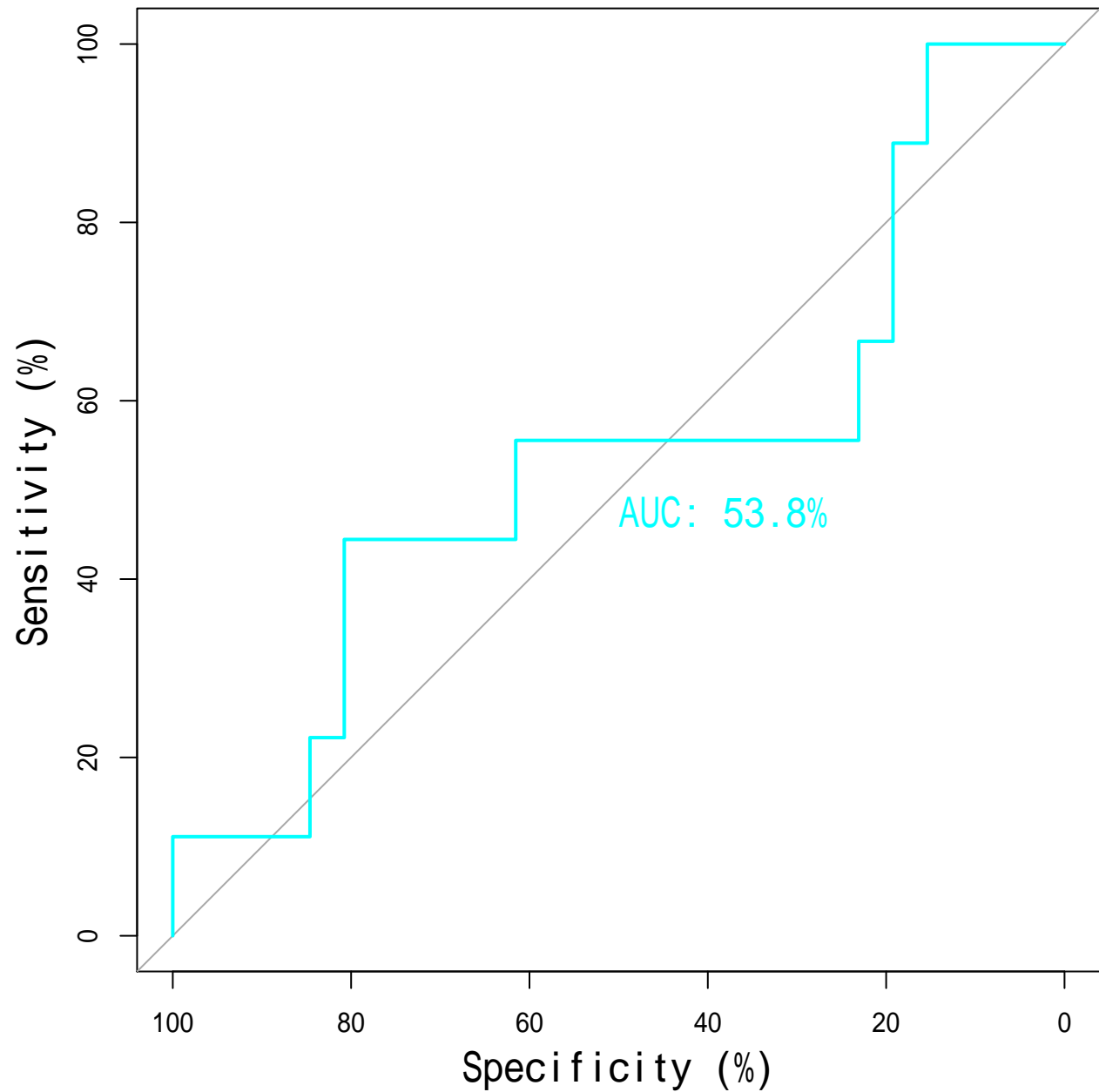

SERPINB1

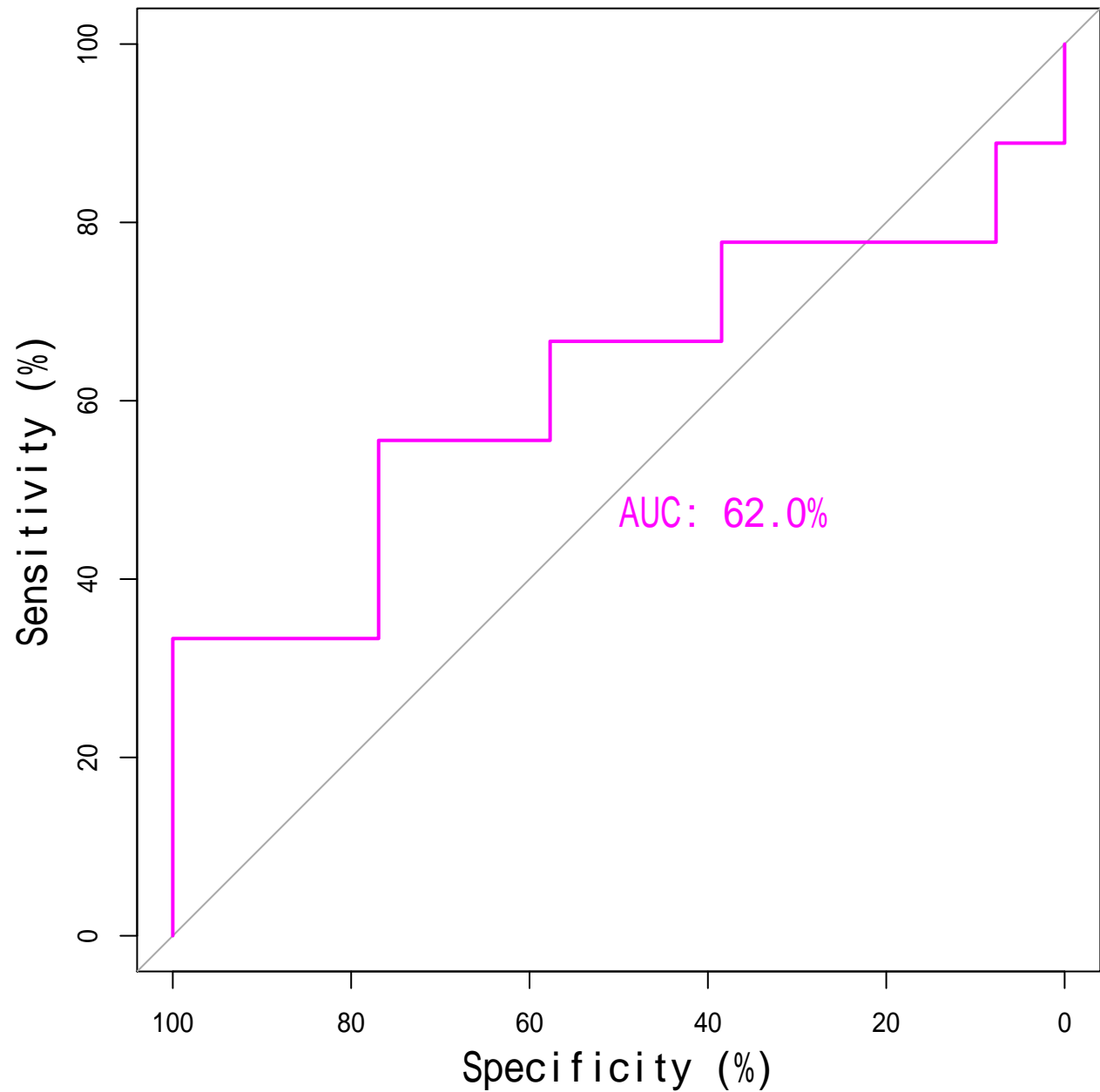

STARD5

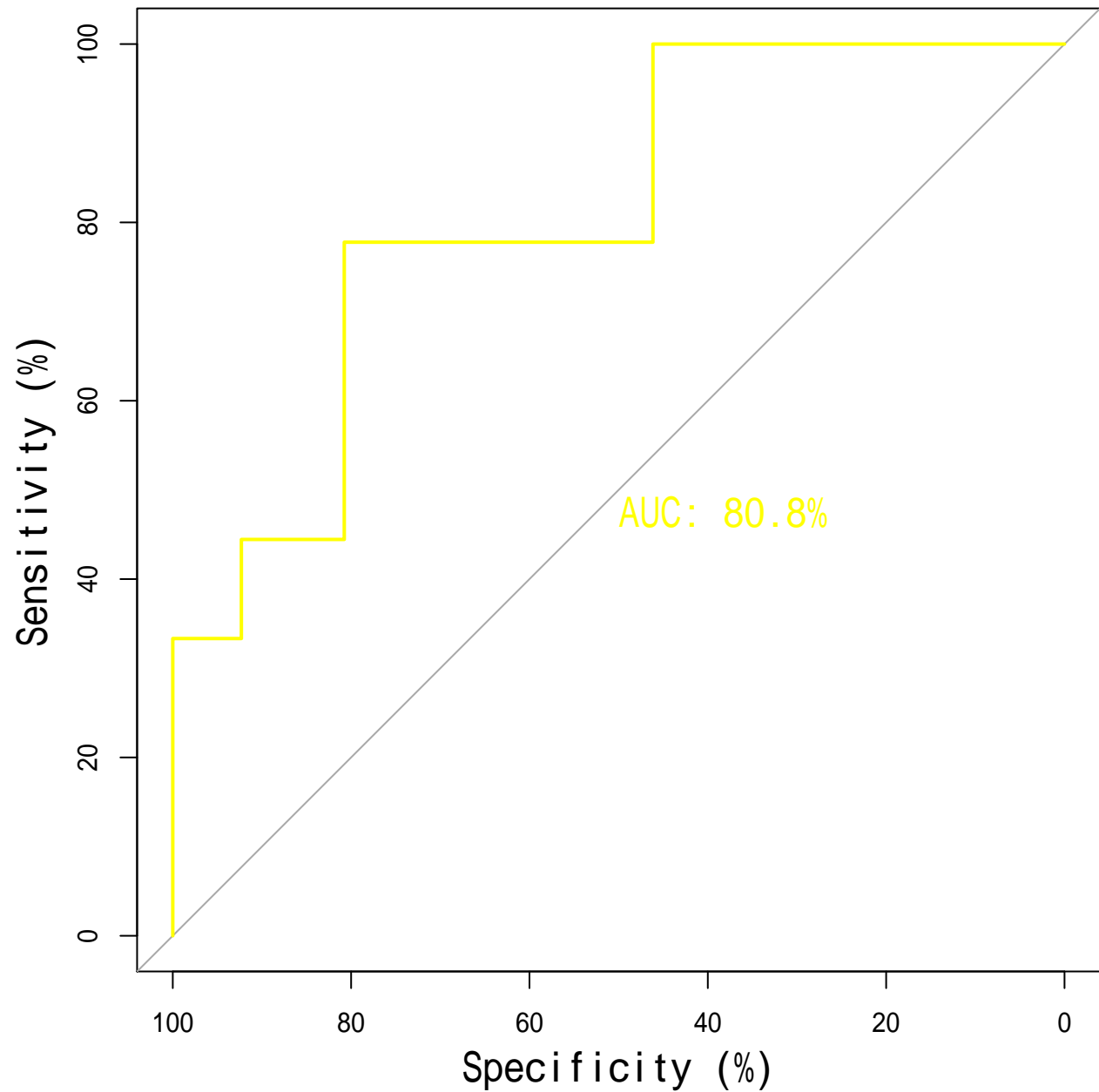

TRIM52

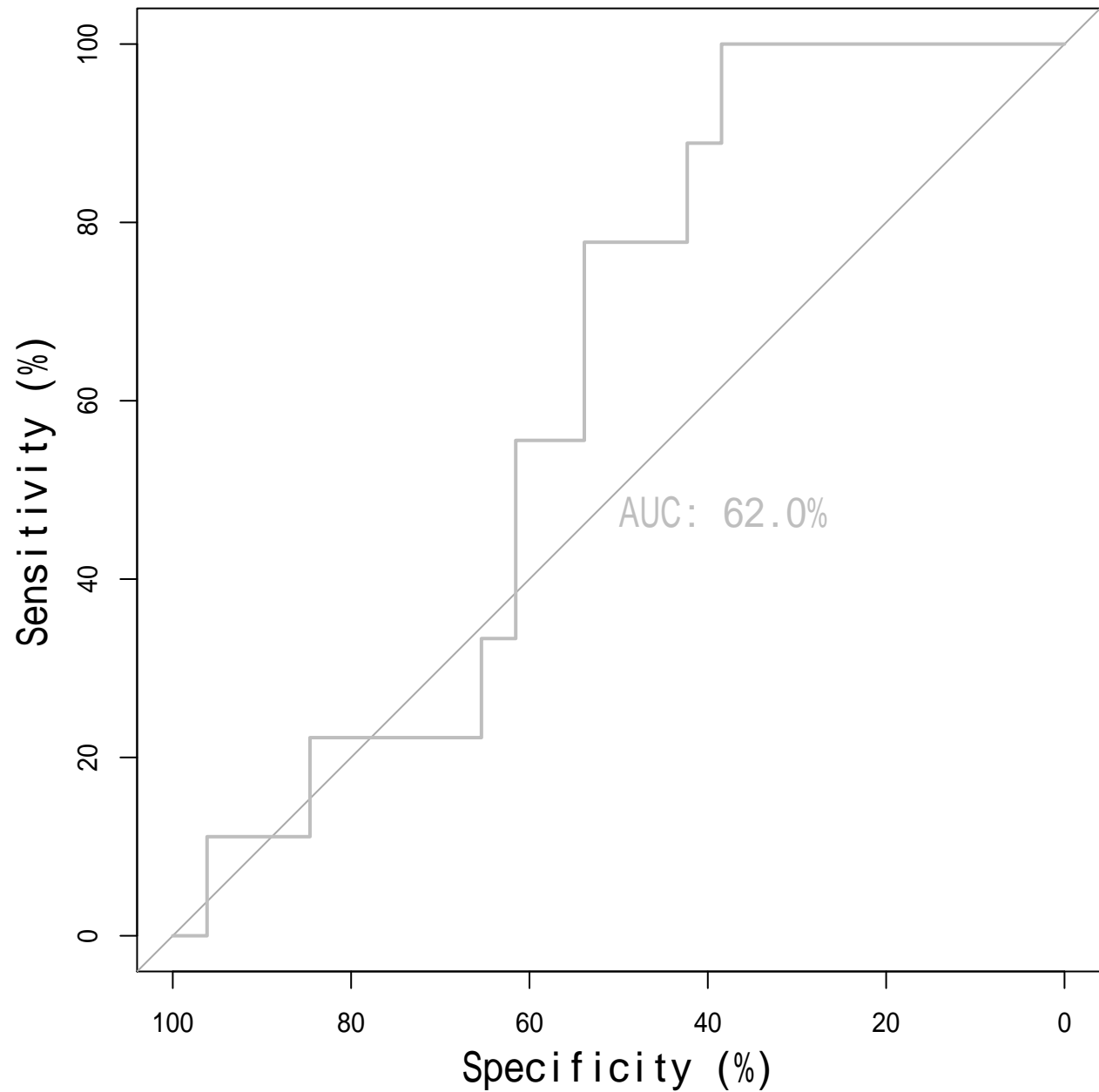

Supplement: Supplementary file 18 [file Data_Sheet_4.PDF]

C4orf3

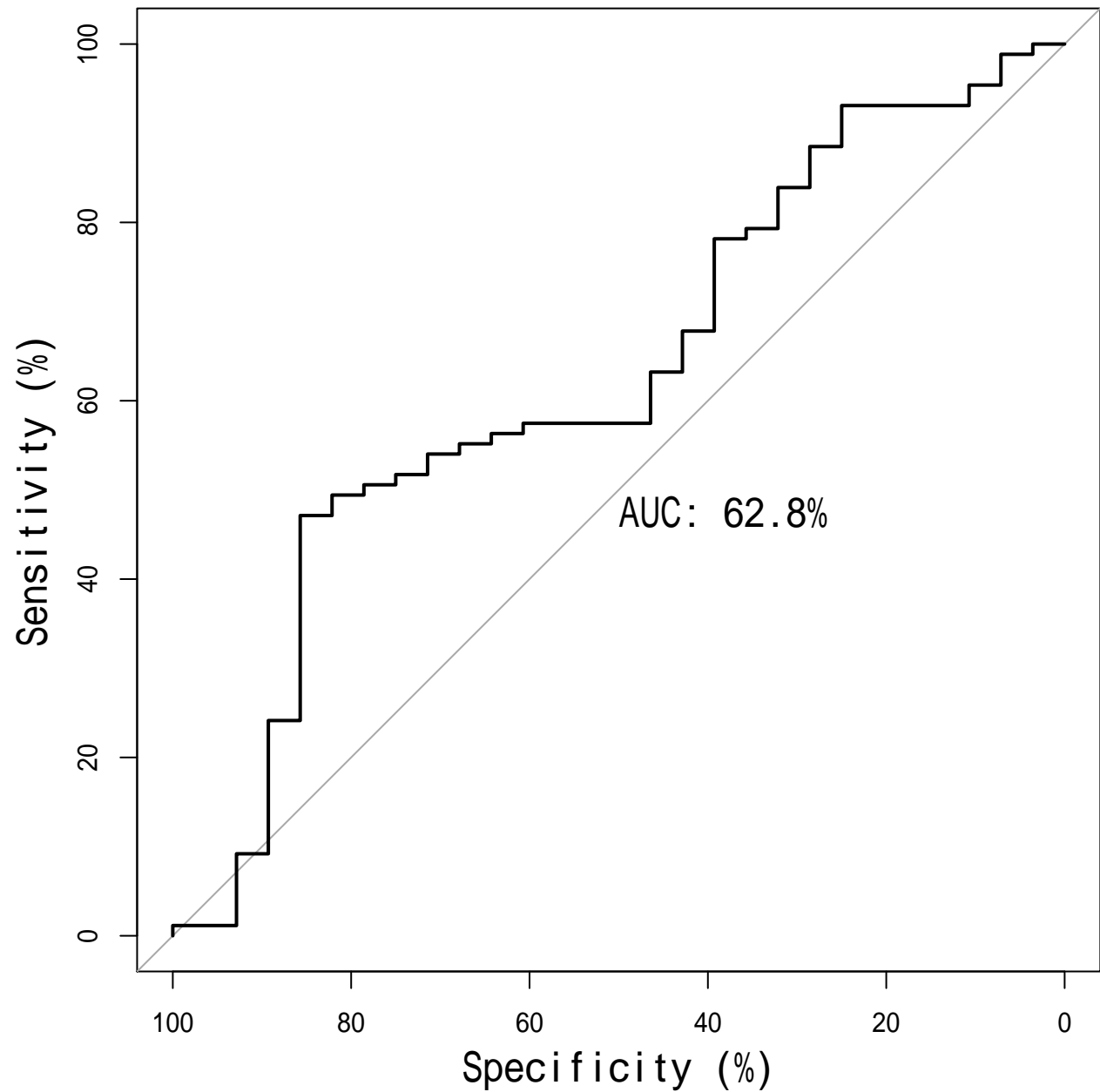

C12orf51

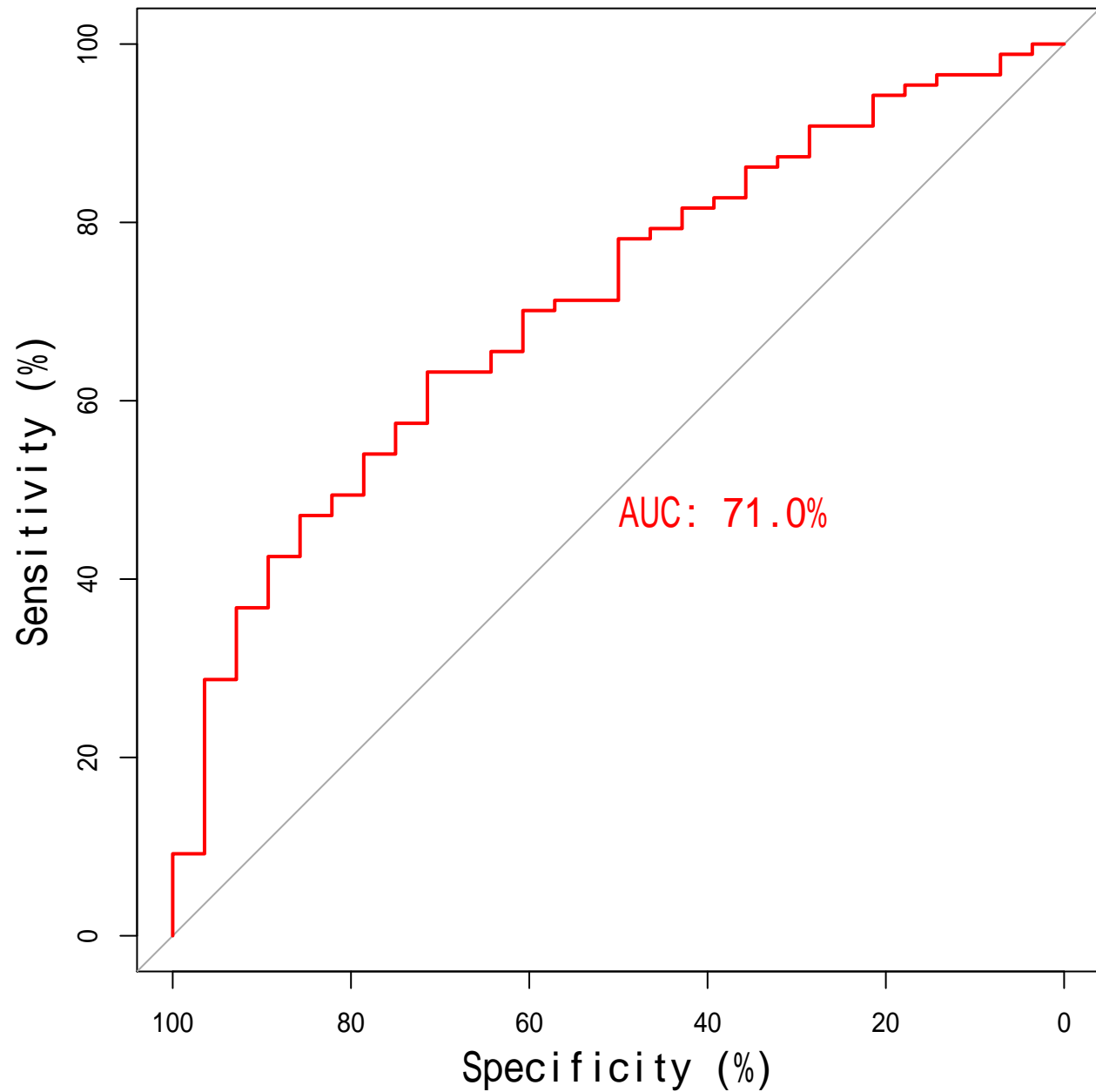

C17orf65

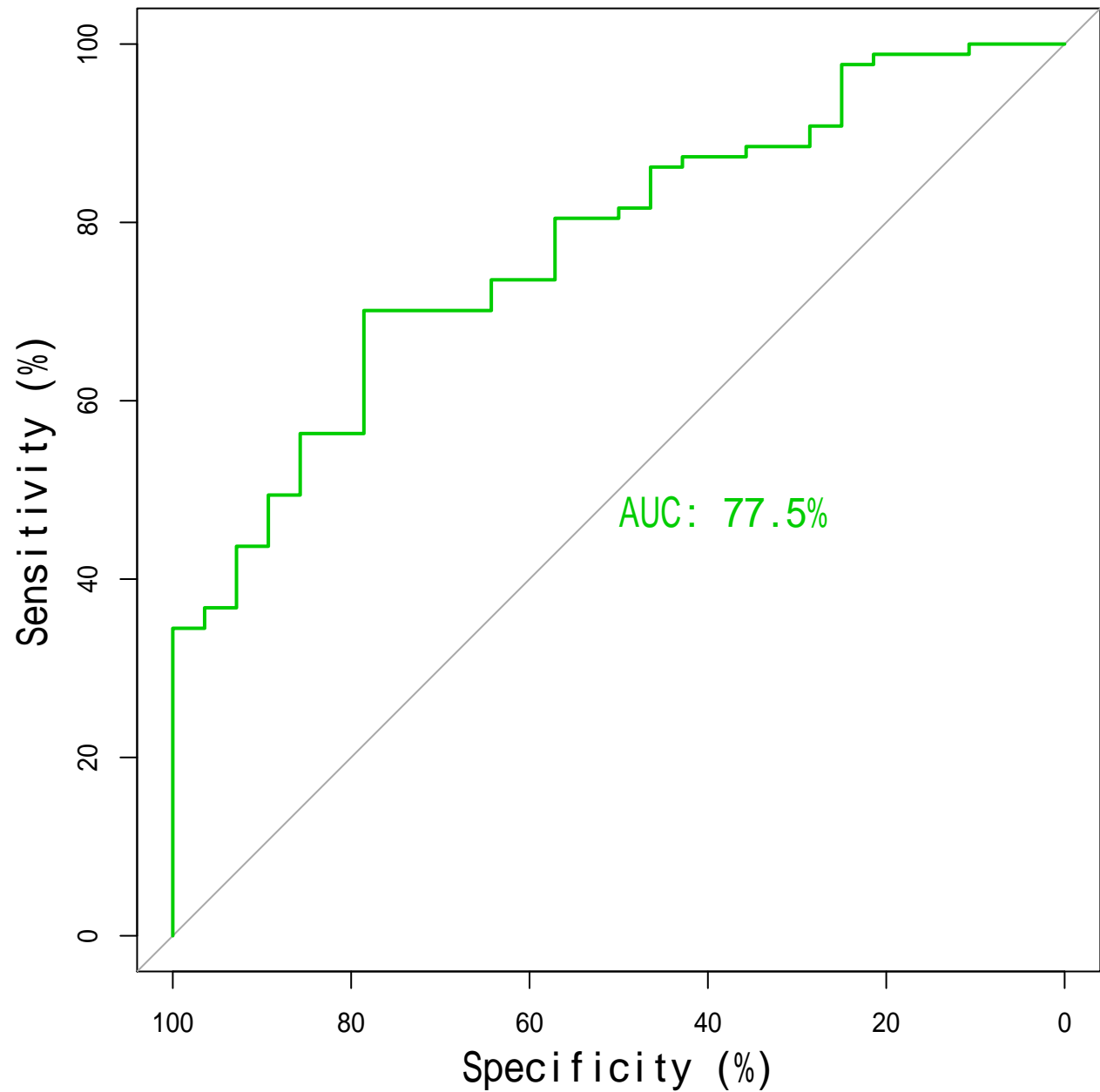

C21orf45

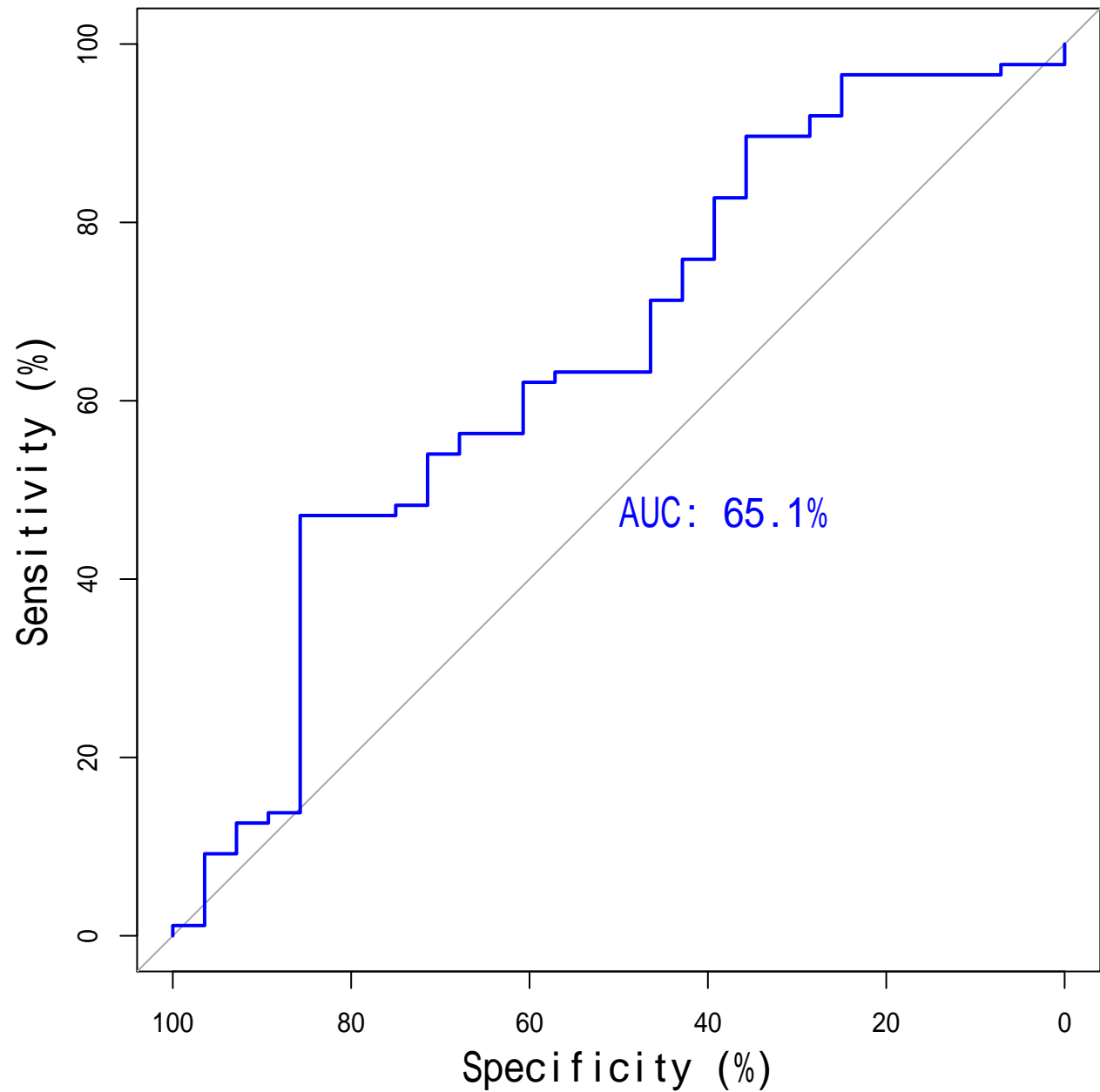

CETN3

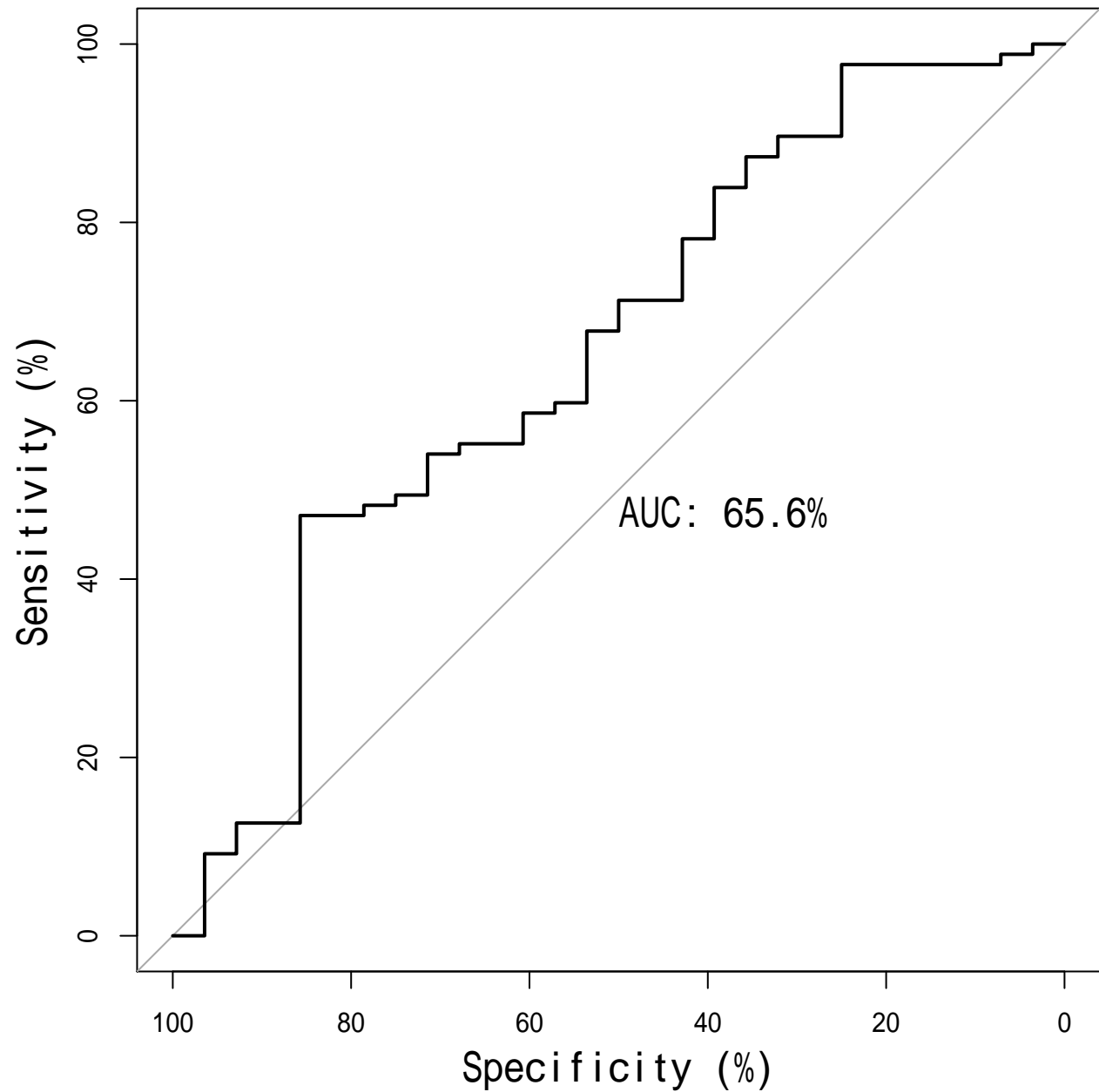

DCAF7

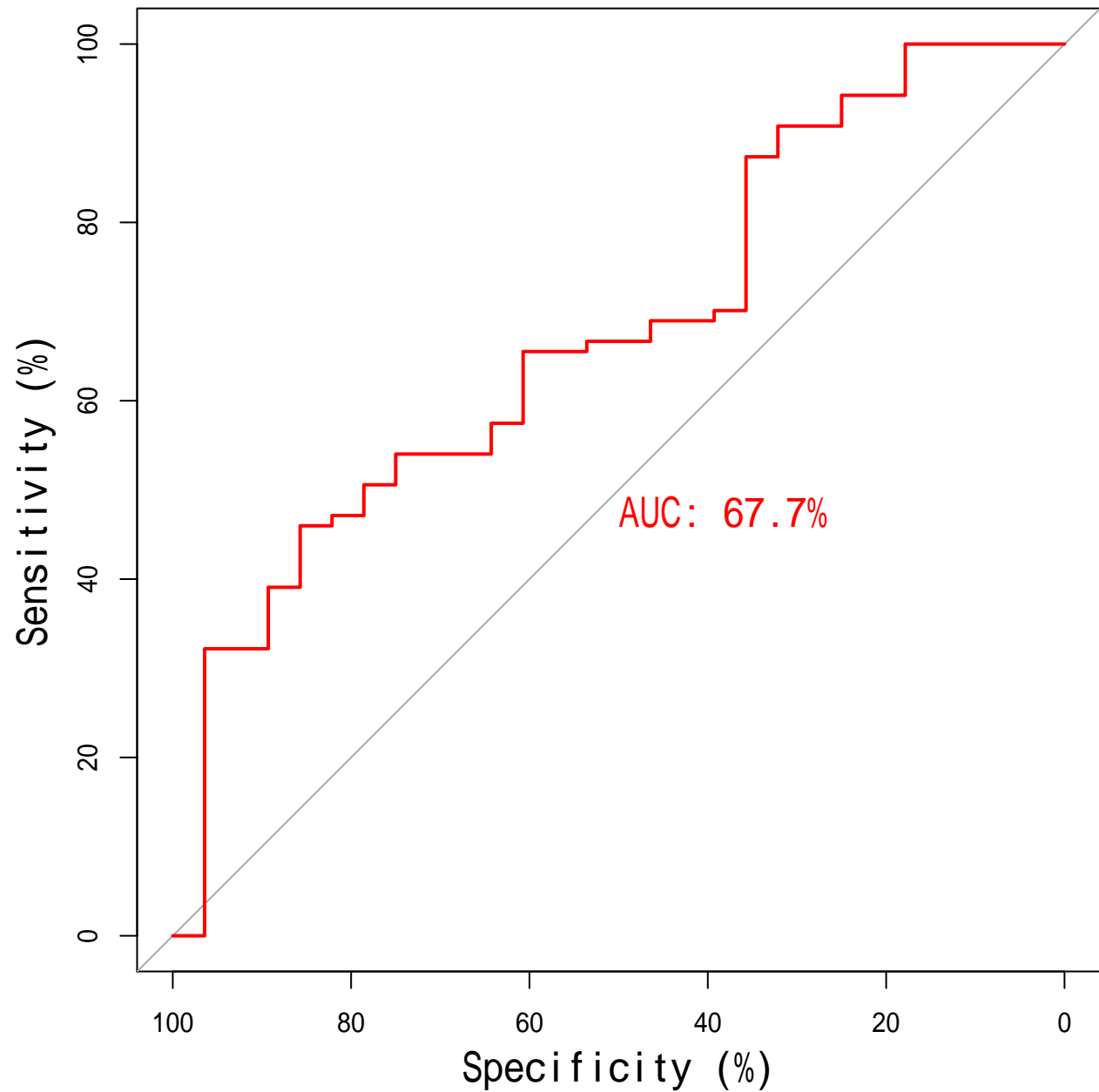

GPX4

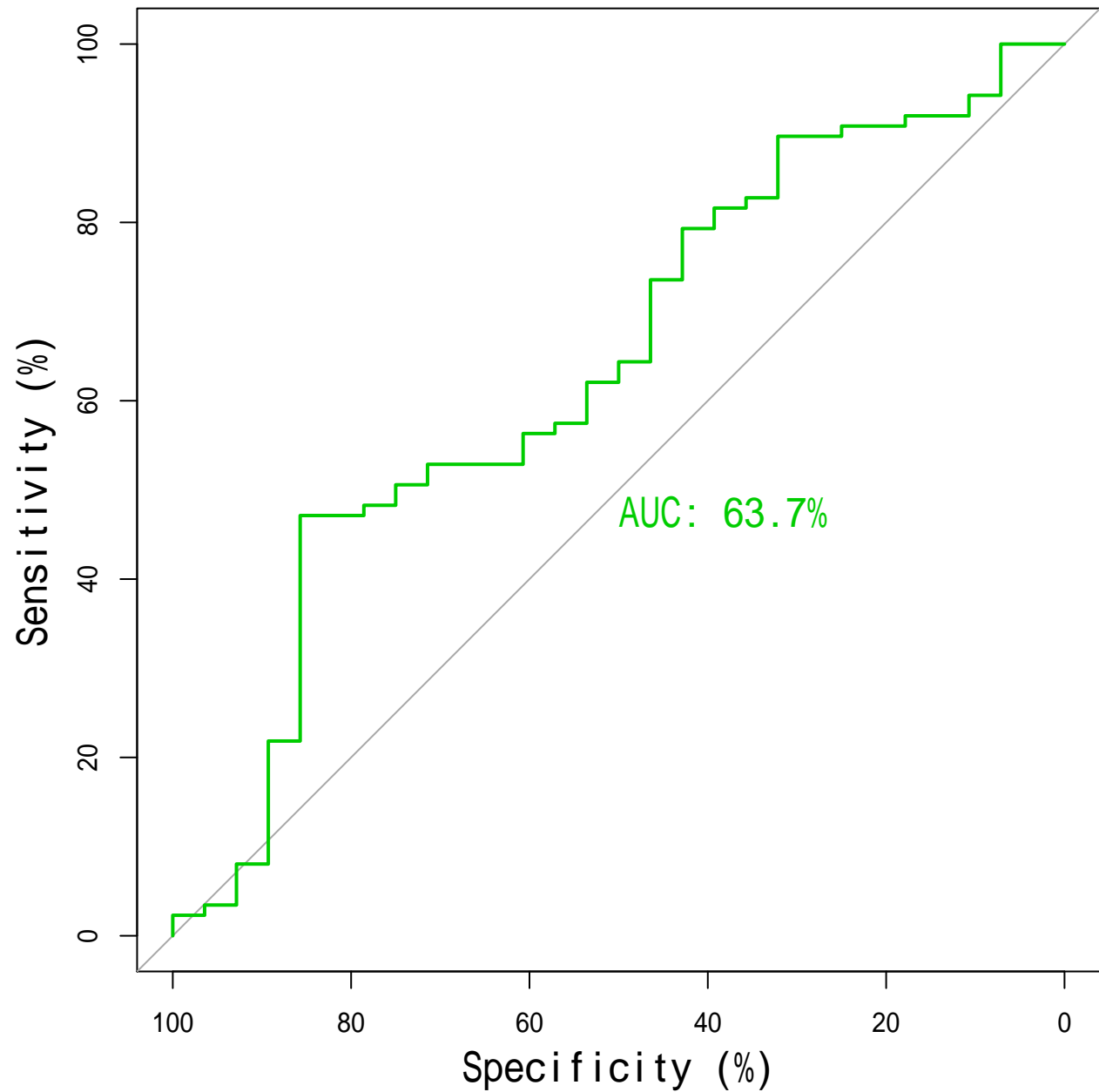

HNRNPA0

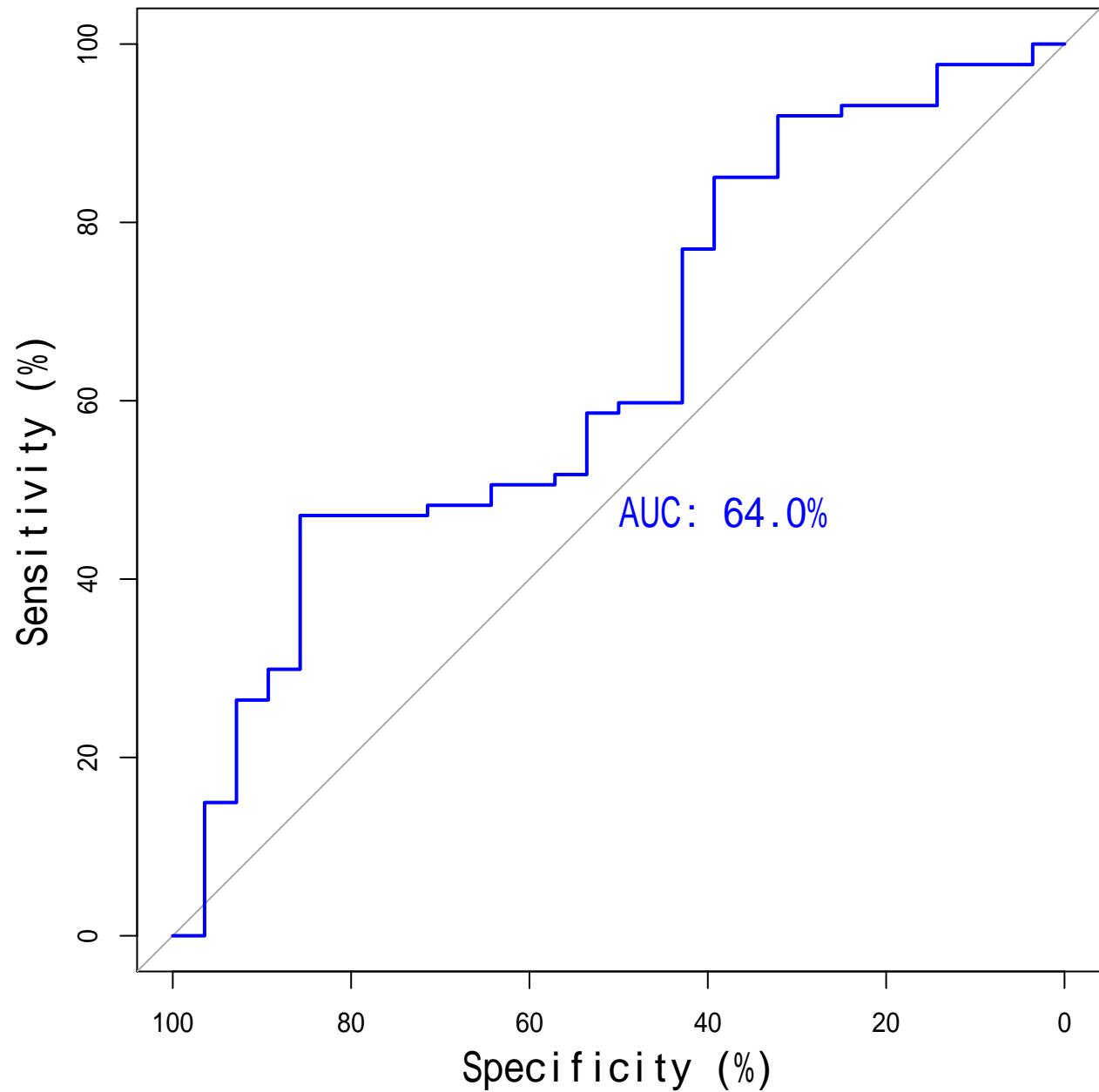

NUP54

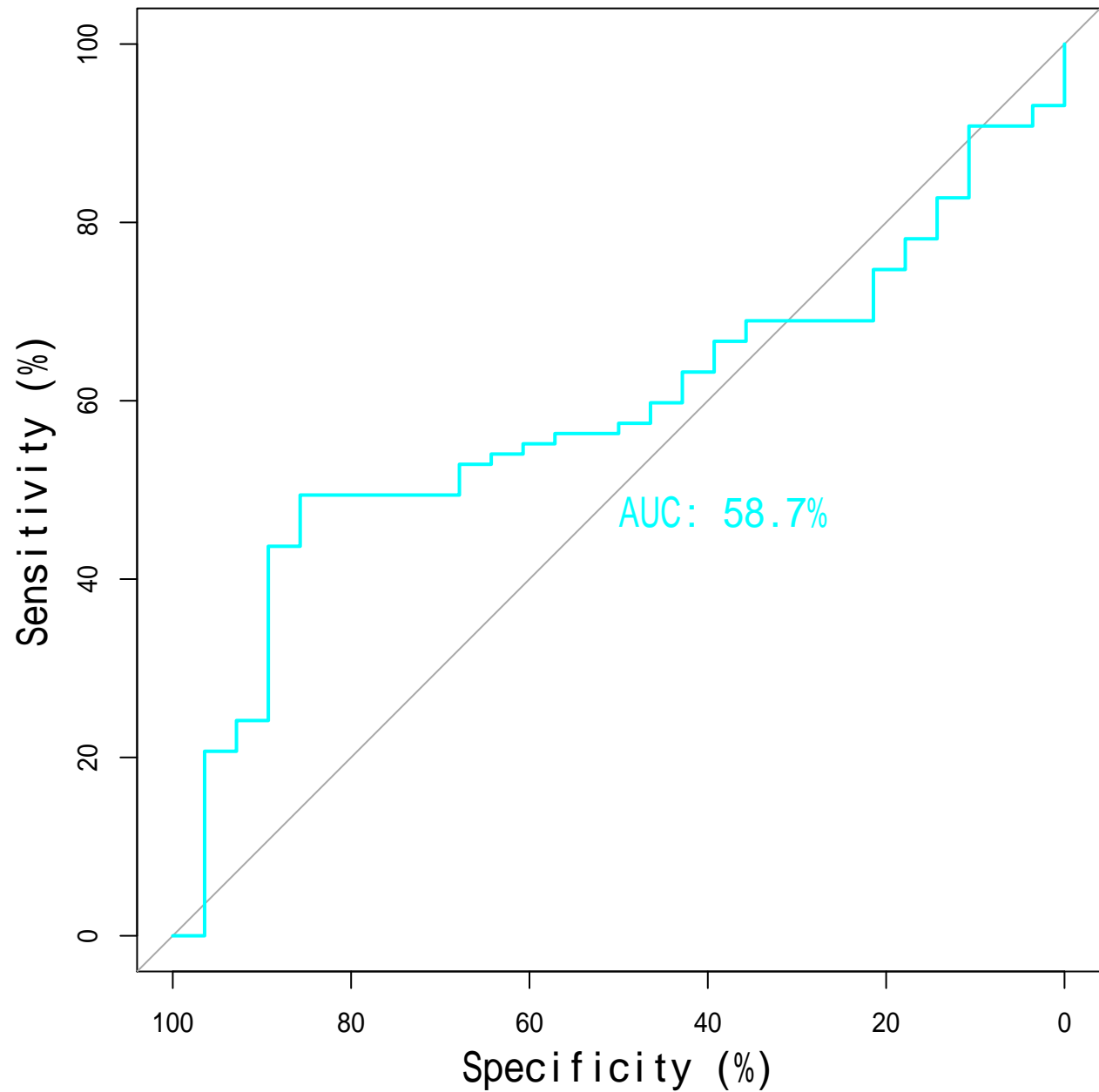

SERPINB1

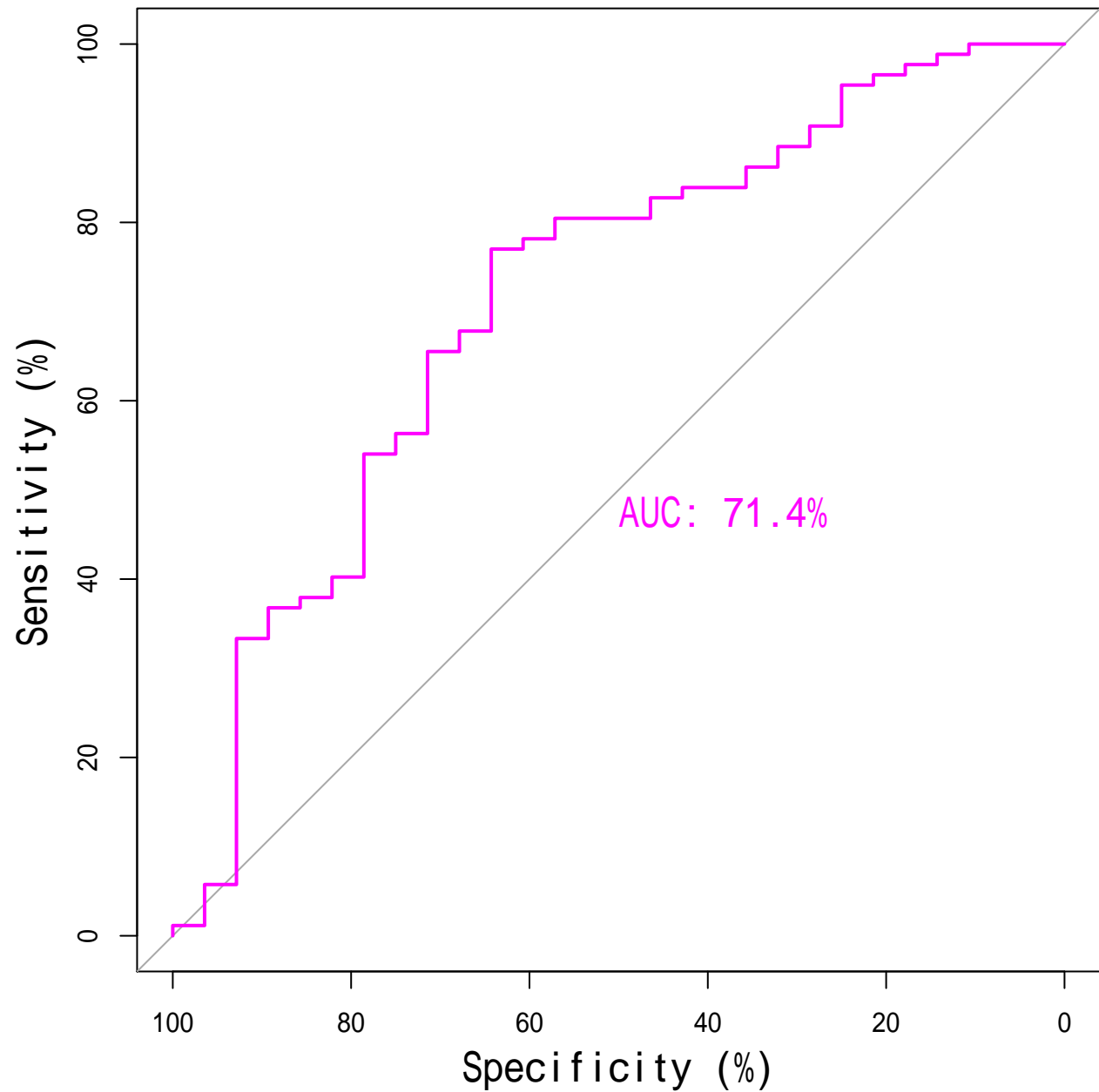

STARD5

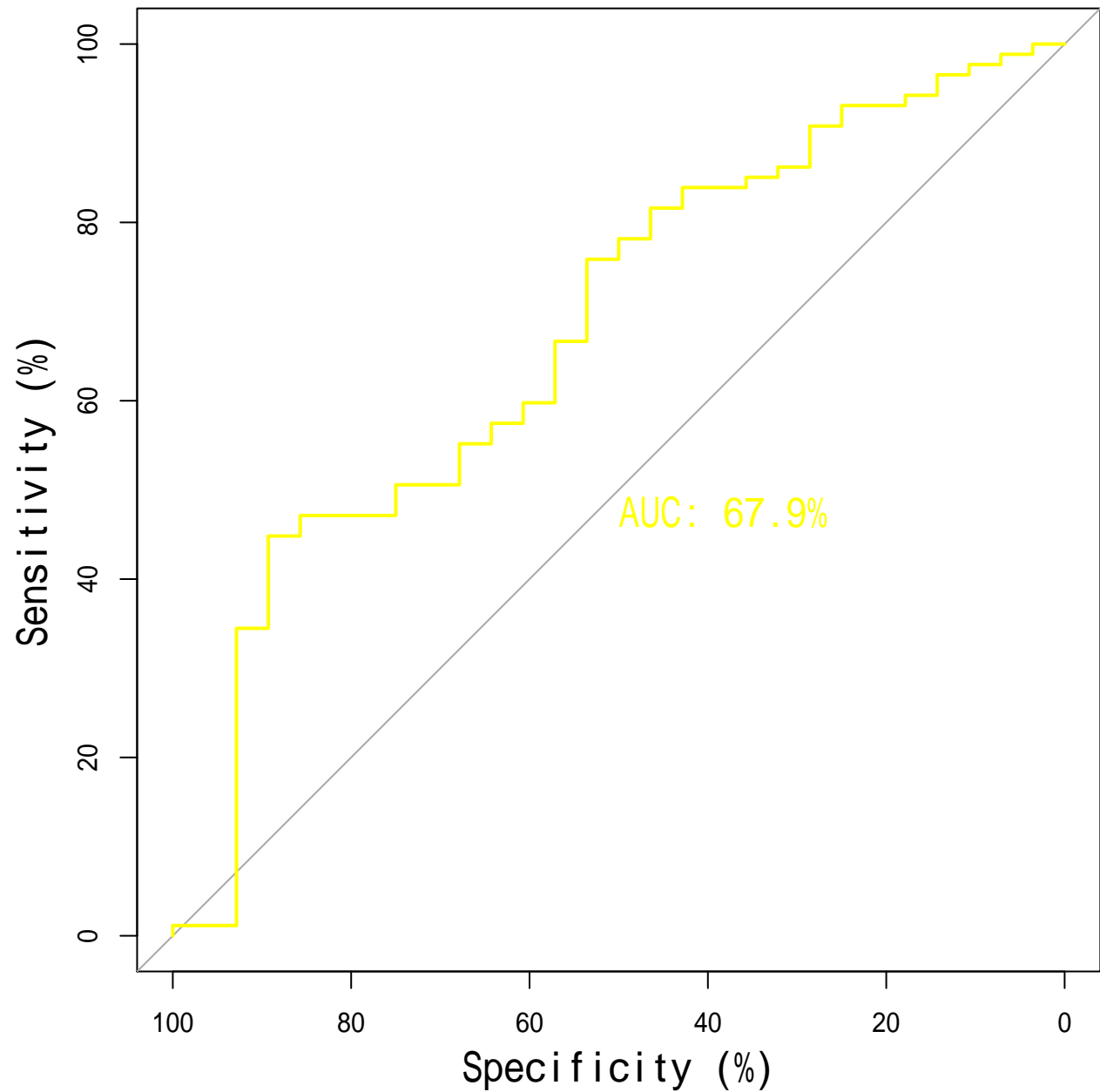

TRIM52

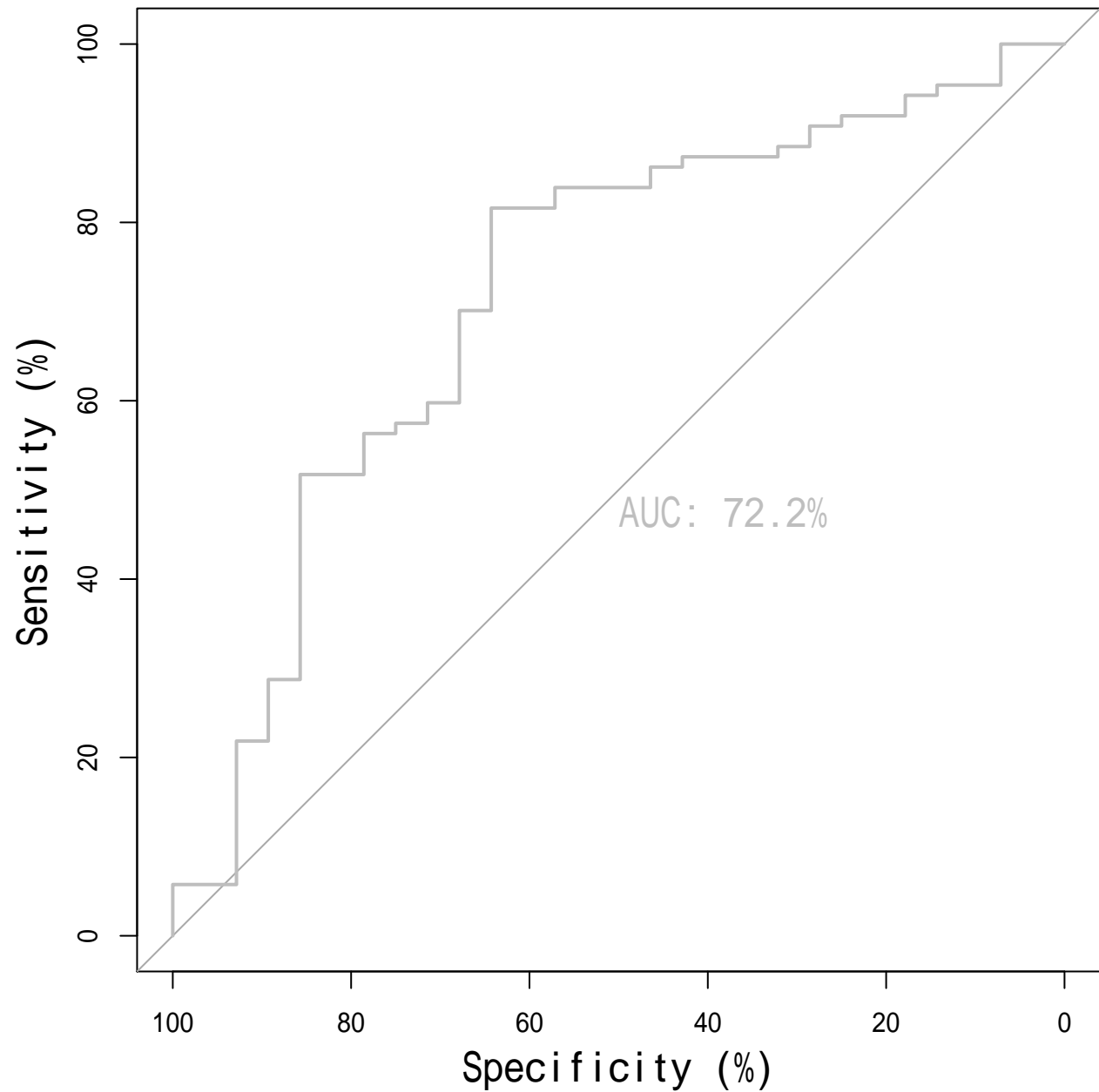

Supplement: Supplementary file 19 [file Data_Sheet_5.PDF]
