# Supplementary material for: Integrative Analysis of DNA Methylation Identified 12 Signature Genes Specific to Metastatic ccRCC
Source: Front Oncol. 2020 Oct 8;10:556018. doi: 10.3389/fonc.2020.556018 (PMC7578385; doi:10.3389/fonc.2020.556018)
Supplement: Supplementary file 16 [file Data_Sheet_2.PDF]

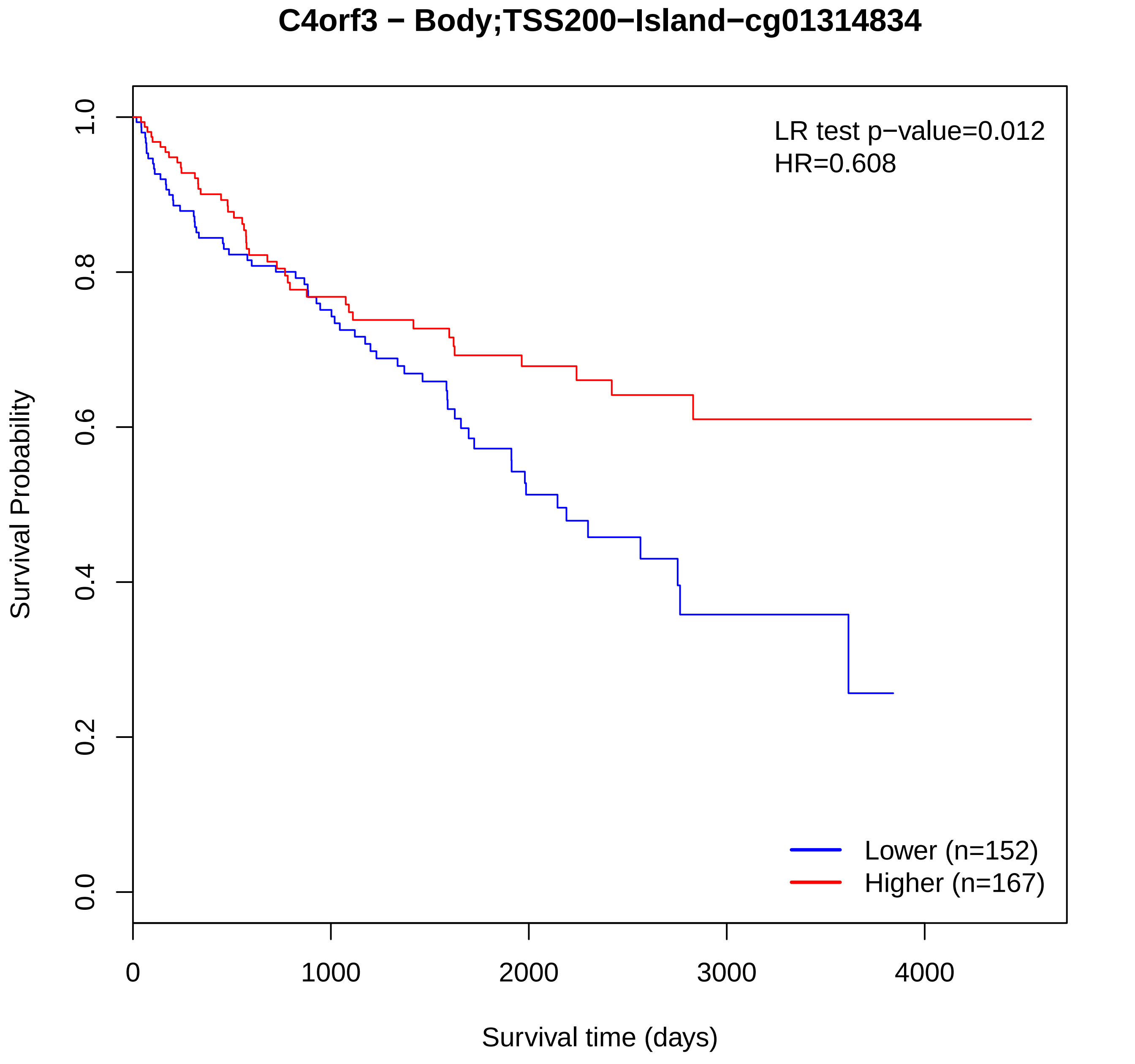
C4orf3 − Body;TSS200−Island−cg01314834
LR test p−value=0.012
HR=0.608
Lower (n=152)
Higher (n=167)
Survival Probability
0.0
0.2
0.4
0.6
0.8
1.0
0
1000
2000
3000
4000
Survival time (days)

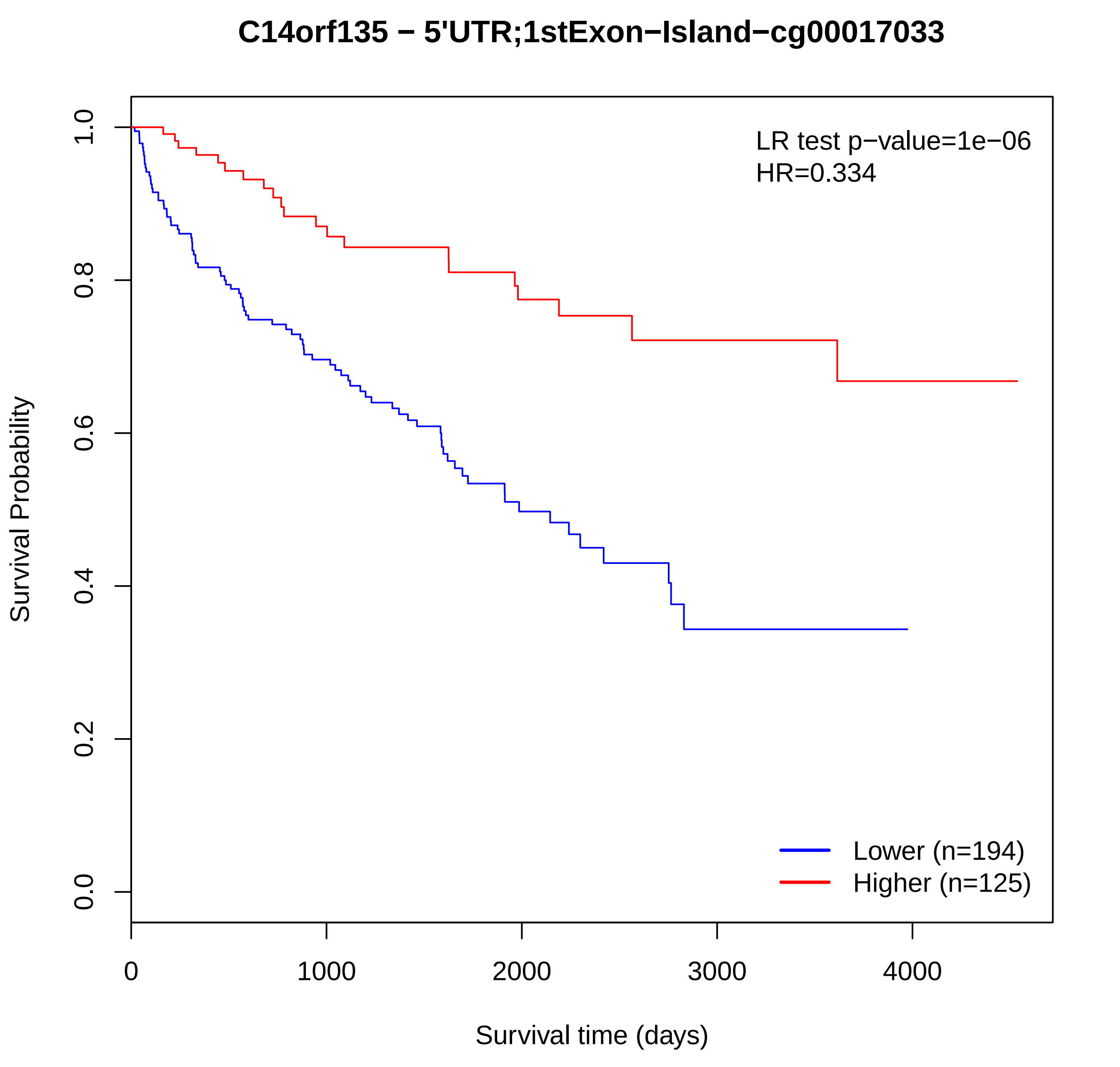
C14orf135 − 5'UTR;1stExon−Island−cg00017033
LR test p−value=1e−06
HR=0.334
Lower (n=194)
Higher (n=125)
Survival Probability
0.0
0.2
0.4
0.6
0.8
1.0
0
1000
2000
3000
4000
Survival time (days)

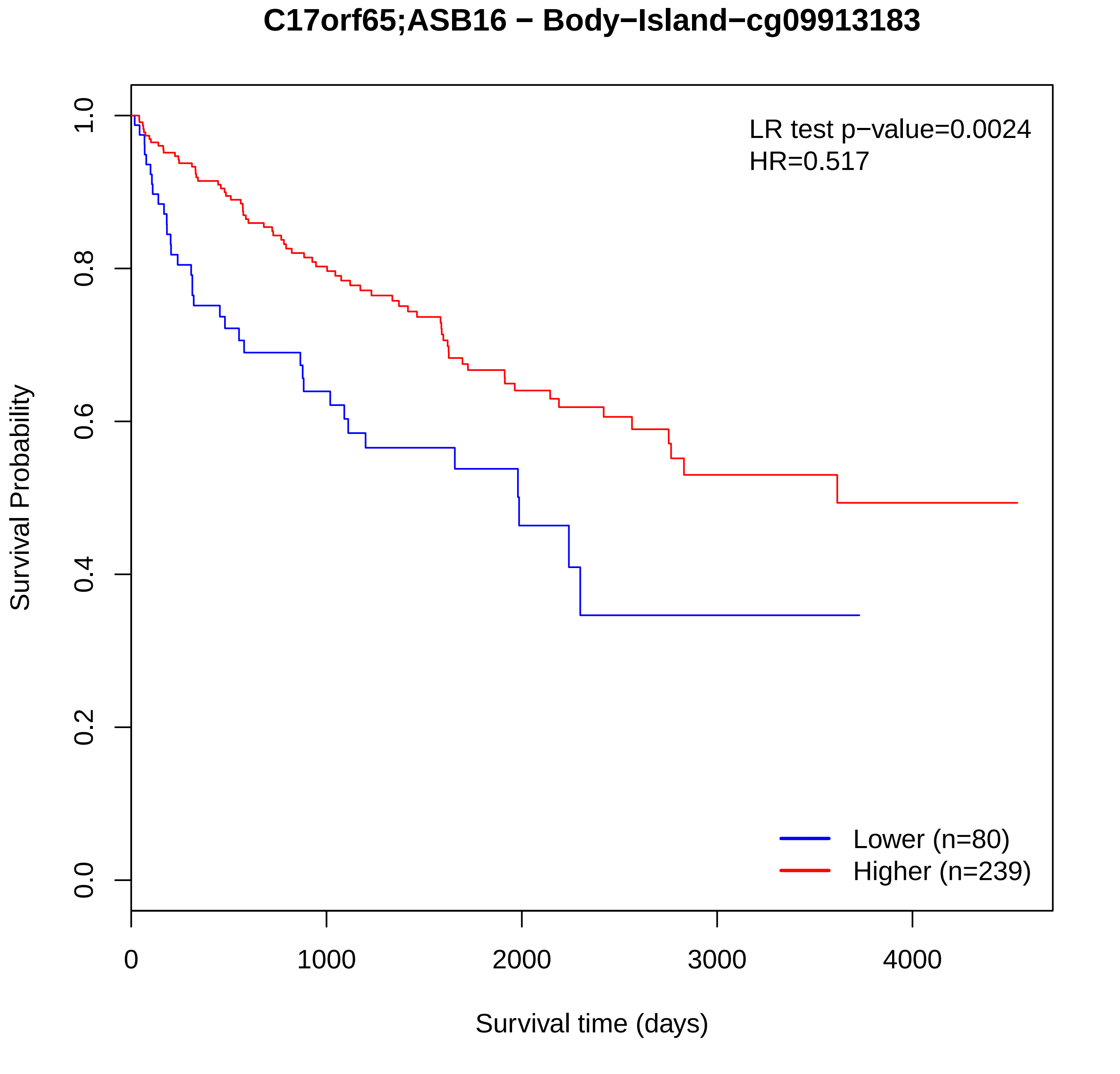

C17orf65;ASB16 − Body−Island−cg09913183
LR test p−value=0.0024
HR=0.517
Lower (n=80)
Higher (n=239)
Survival Probability
0.0
0.2
0.4
0.6
0.8
1.0
0
1000
2000
3000
4000
Survival time (days)

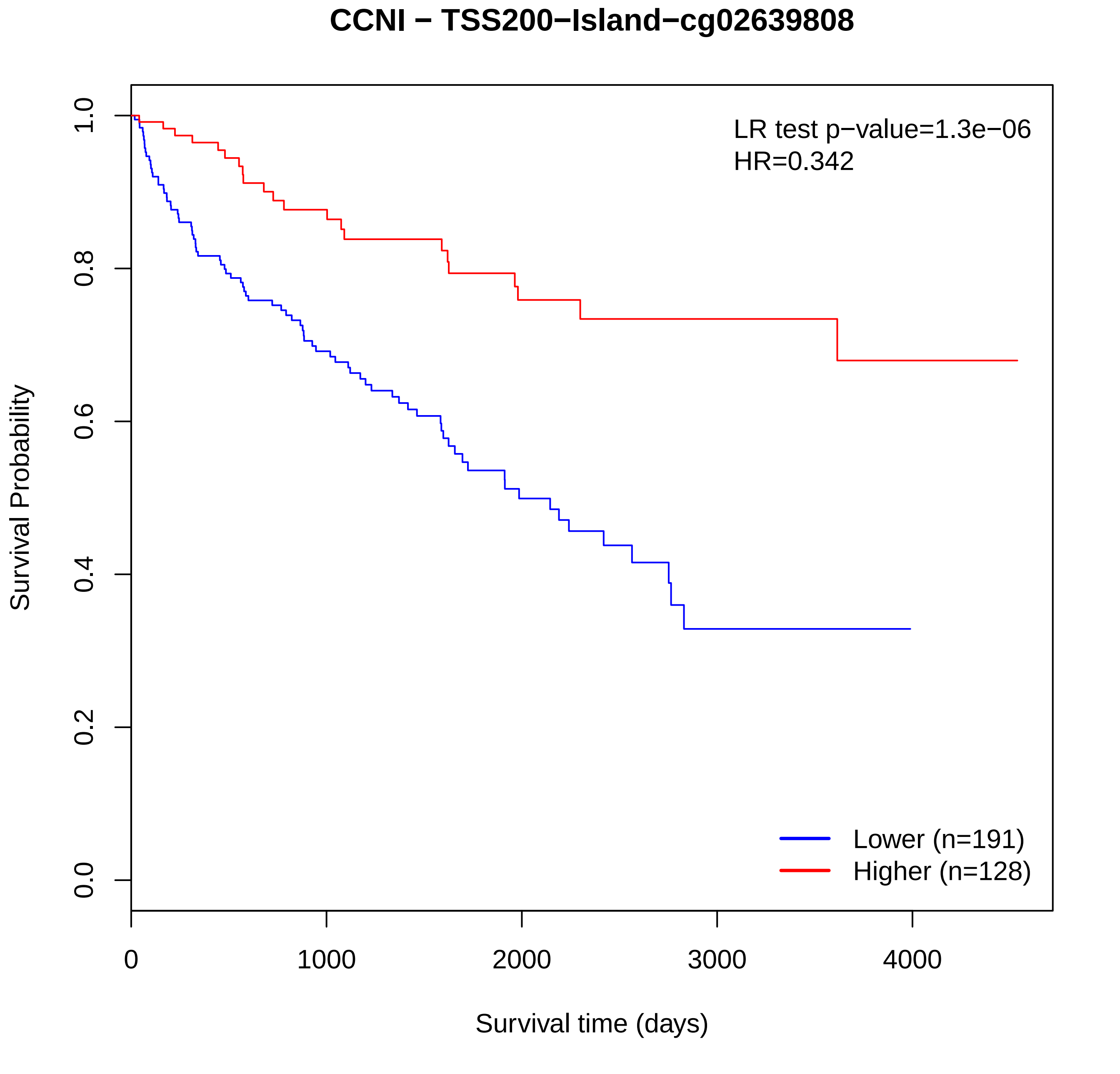

CCNI − TSS200−Island−cg02639808
LR test p−value=1.3e−06
HR=0.342
Lower (n=191)
Higher (n=128)
Survival Probability
Survival time (days)
0.0
0.2
0.4
0.6
0.8
1.0
0
1000
2000
3000
4000

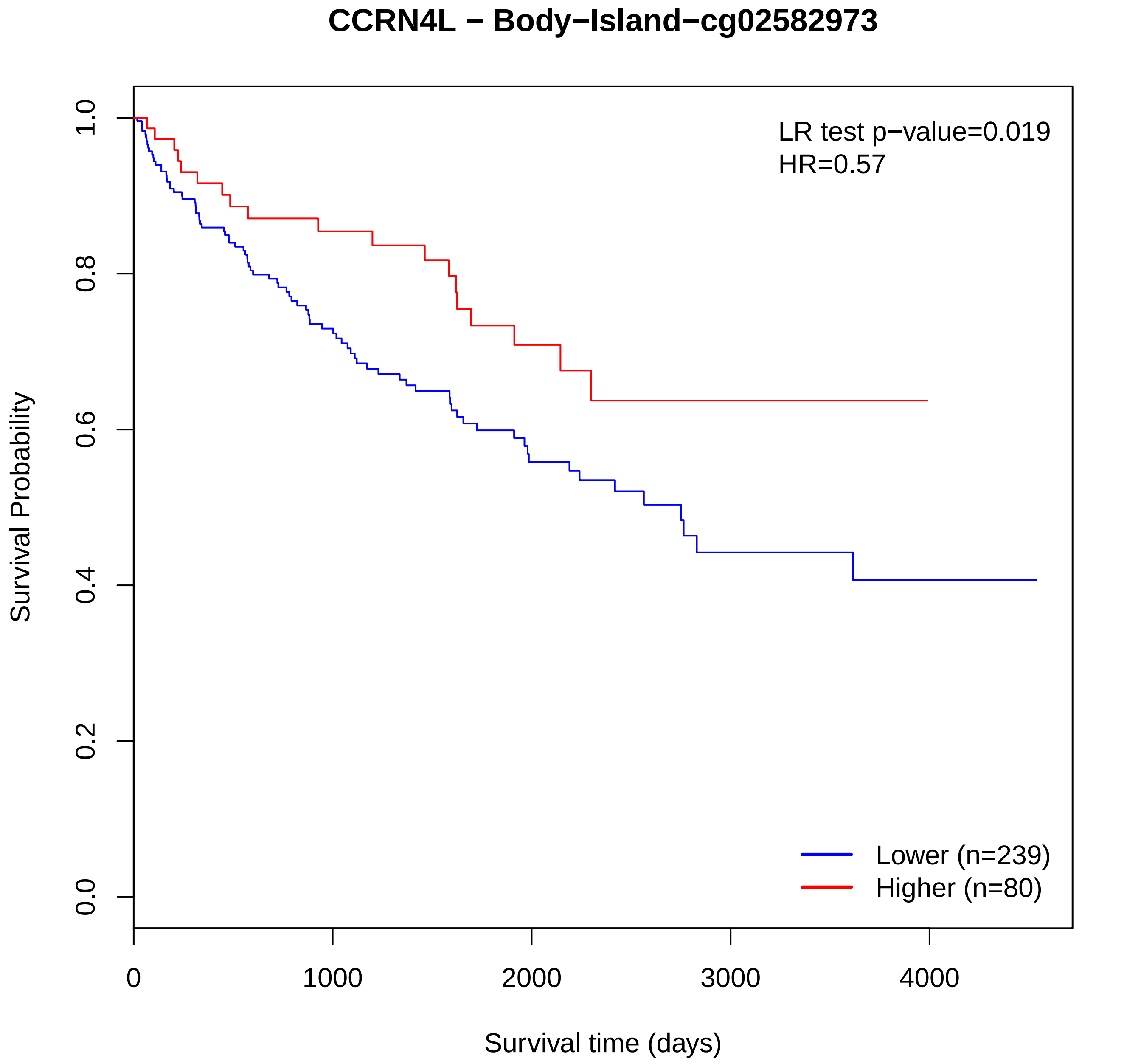
CCRN4L − Body−Island−cg02582973
LR test p−value=0.019
HR=0.57
Lower (n=239)
Higher (n=80)
Survival Probability
Survival time (days)
0.0
0.2
0.4
0.6
0.8
1.0
0
1000
2000
3000
4000

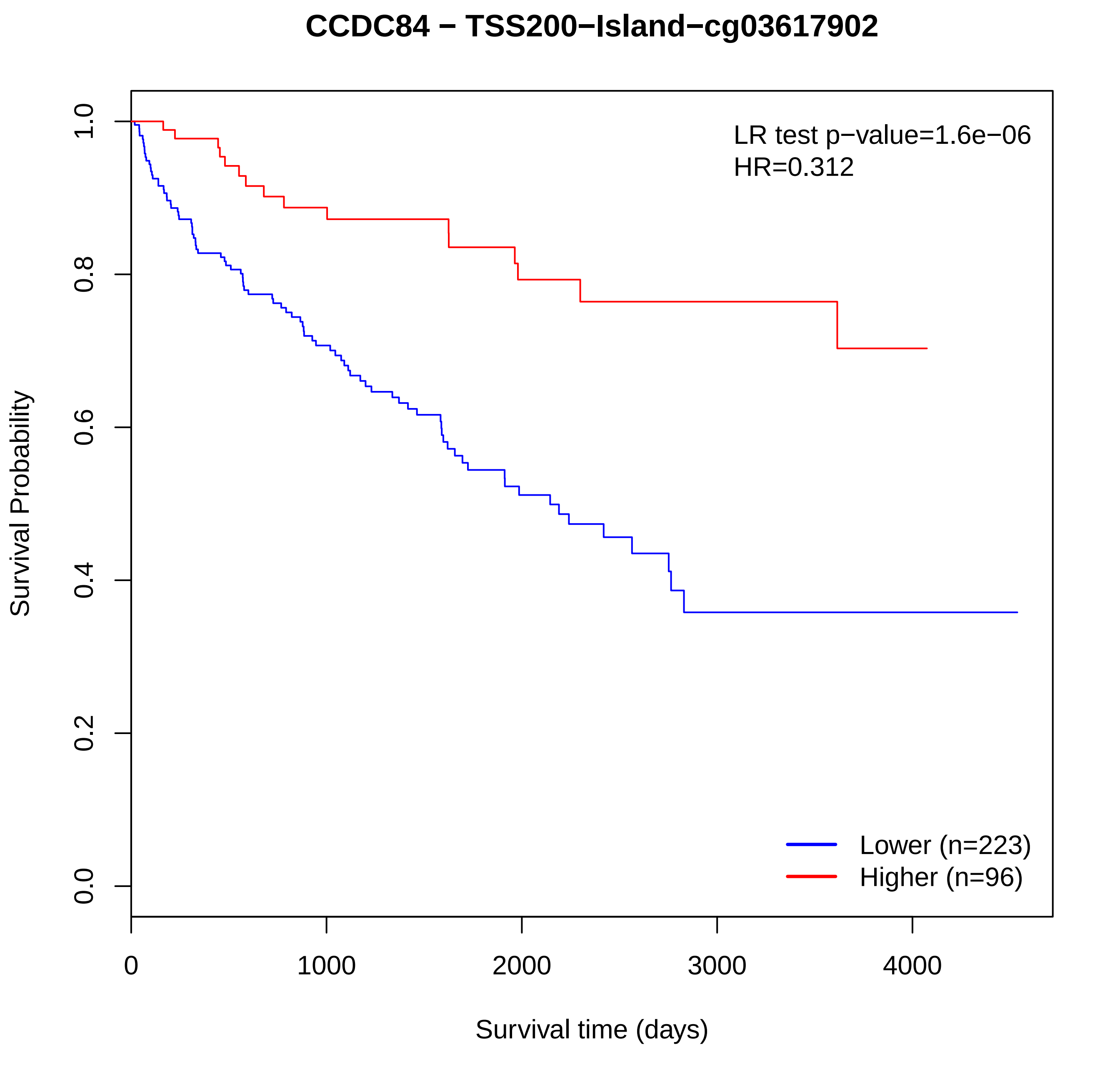
CCDC84 − TSS200−Island−cg03617902
LR test p−value=1.6e−06
HR=0.312
Lower (n=223)
Higher (n=96)
Survival Probability
Survival time (days)
0.0
0.2
0.4
0.6
0.8
1.0
0
1000
2000
3000
4000

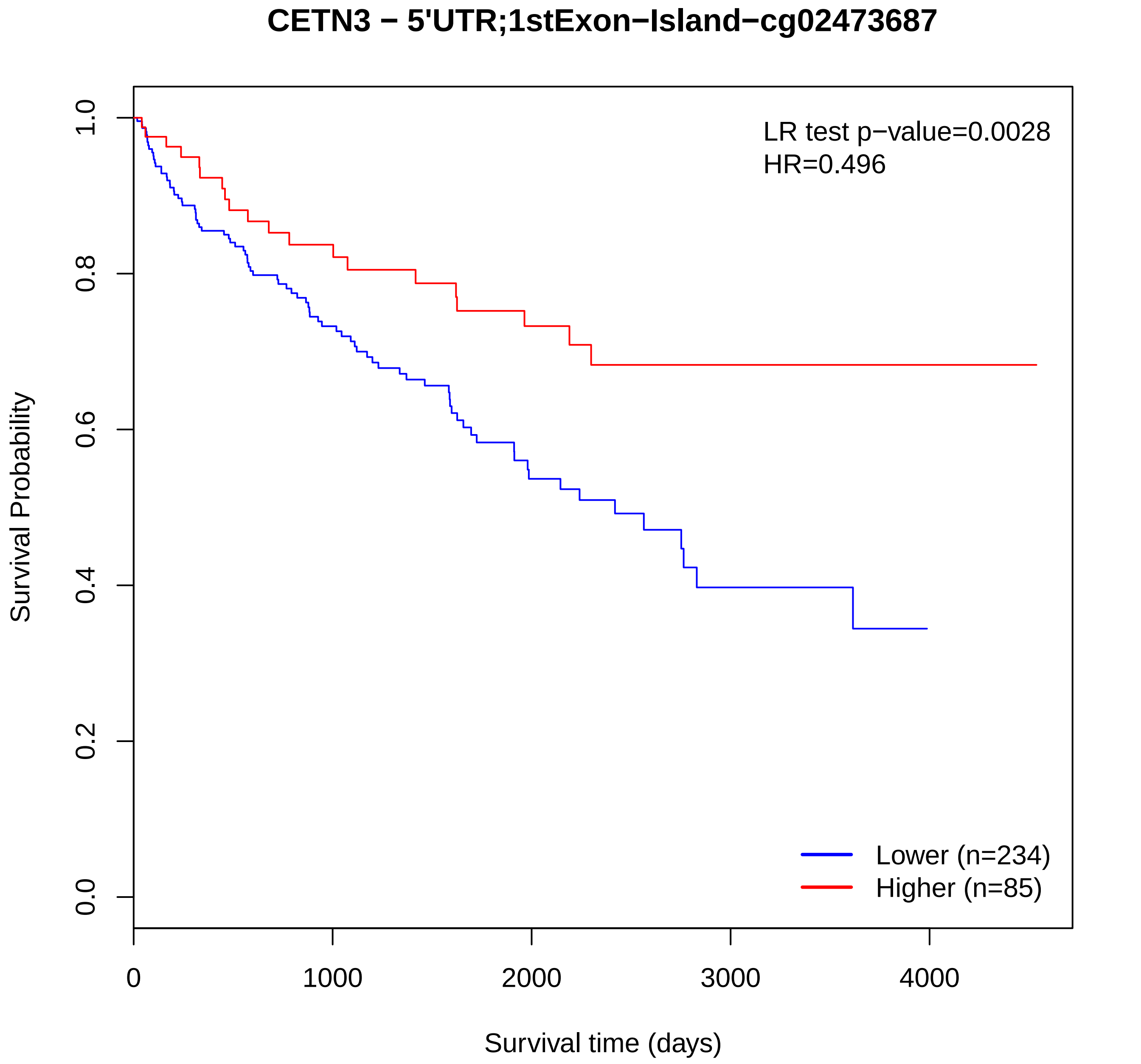
CETN3 − 5'UTR;1stExon−Island−cg02473687
LR test p−value=0.0028
HR=0.496
Lower (n=234)
Higher (n=85)
Survival Probability
0.0
0.2
0.4
0.6
0.8
1.0
0
1000
2000
3000
4000
Survival time (days)

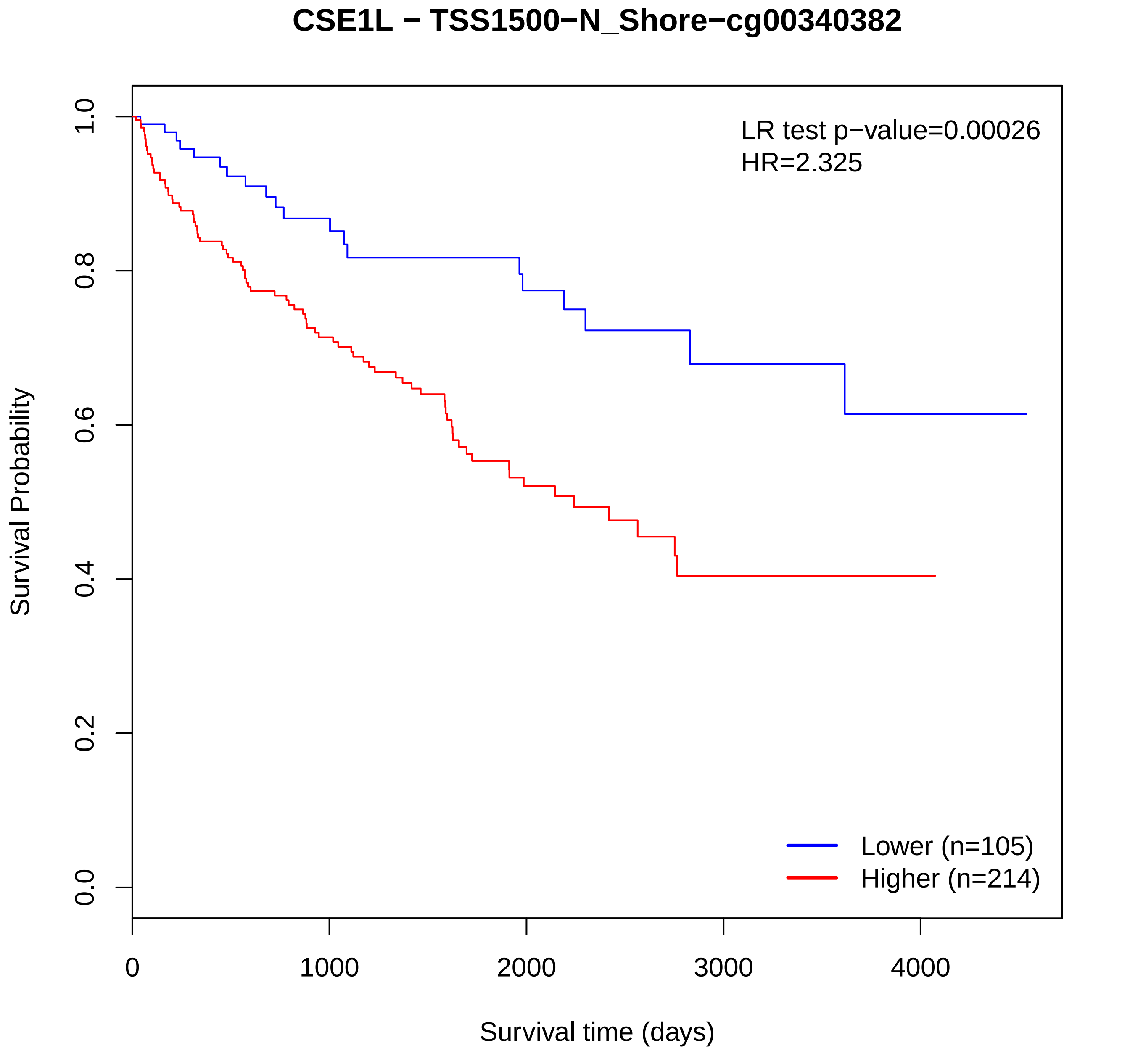

CSE1L − TSS1500−N_Shore−cg00340382
LR test p−value=0.00026
HR=2.325
Lower (n=105)
Higher (n=214)
Survival Probability
Survival time (days)
0.0
0.2
0.4
0.6
0.8
1.0
0
1000
2000
3000
4000

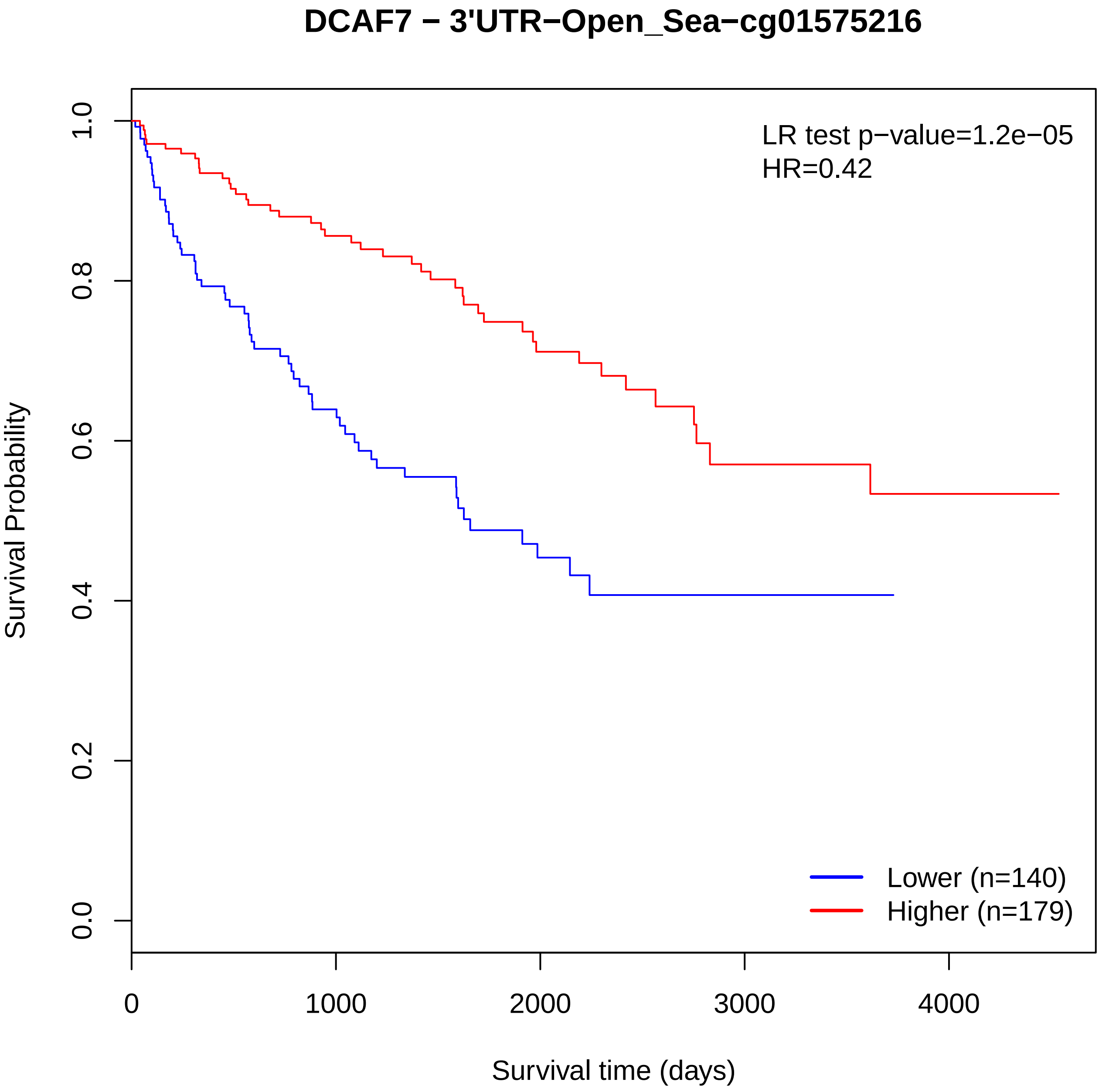

DCAF7 − 3'UTR−Open_Sea−cg01575216
LR test p−value=1.2e−05
HR=0.42
Lower (n=140)
Higher (n=179)
Survival Probability
0.0
0.2
0.4
0.6
0.8
1.0
0
1000
2000
3000
4000
Survival time (days)

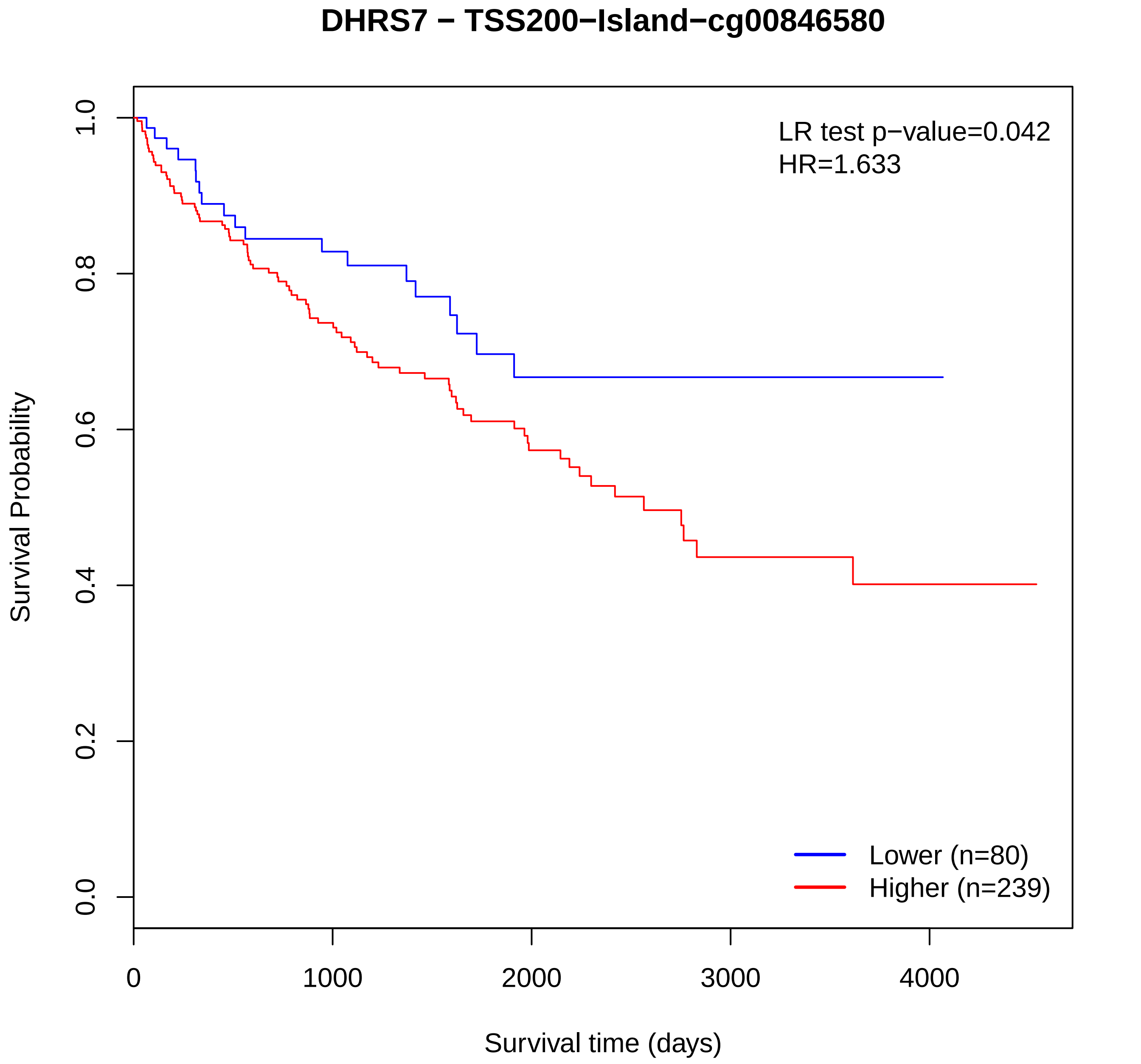
DHRS7 − TSS200−Island−cg00846580
LR test p−value=0.042
HR=1.633
Lower (n=80)
Higher (n=239)
Survival Probability
0.0
0.2
0.4
0.6
0.8
1.0
0
1000
2000
3000
4000
Survival time (days)

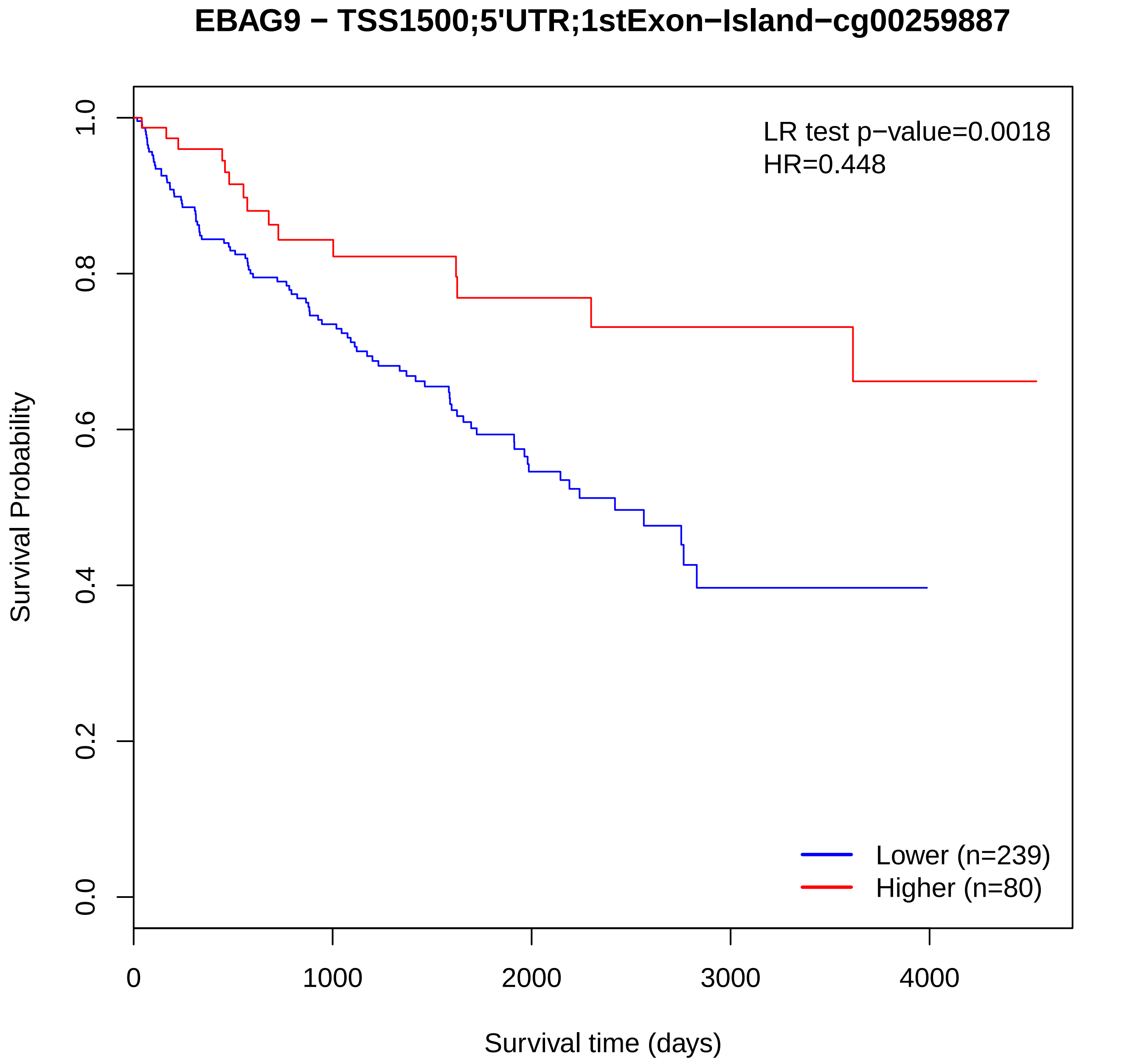
EBAG9 − TSS1500;5'UTR;1stExon−Island−cg00259887
LR test p−value=0.0018
HR=0.448
Survival Probability
0.0
0.2
0.4
0.6
0.8
1.0
0
1000
2000
3000
4000
Survival time (days)
Lower (n=239)
Higher (n=80)

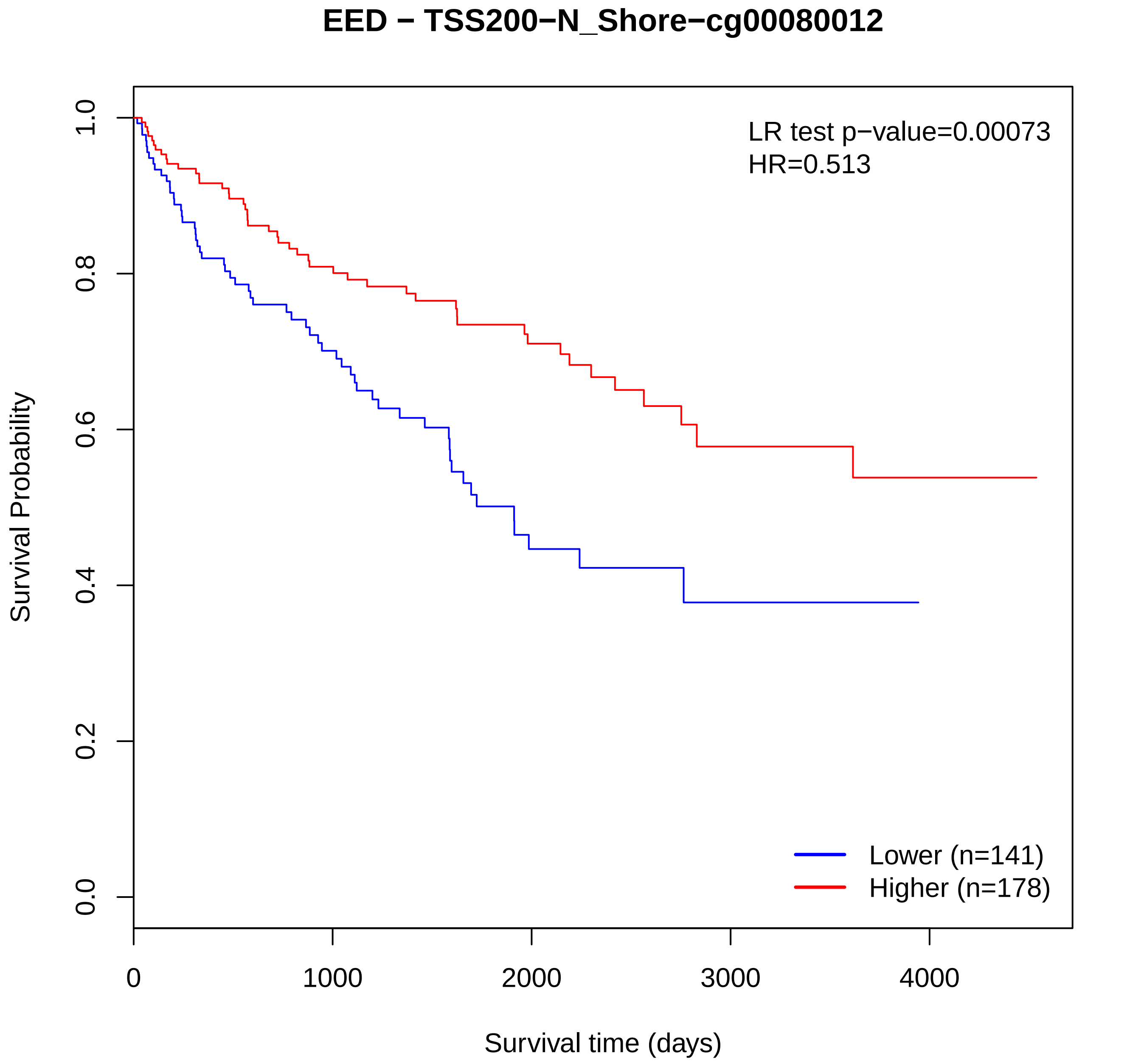

EED − TSS200−N_Shore−cg00080012
LR test p−value=0.00073
HR=0.513
Lower (n=141)
Higher (n=178)
Survival Probability
Survival time (days)
0.0
0.2
0.4
0.6
0.8
1.0
0
1000
2000
3000
4000

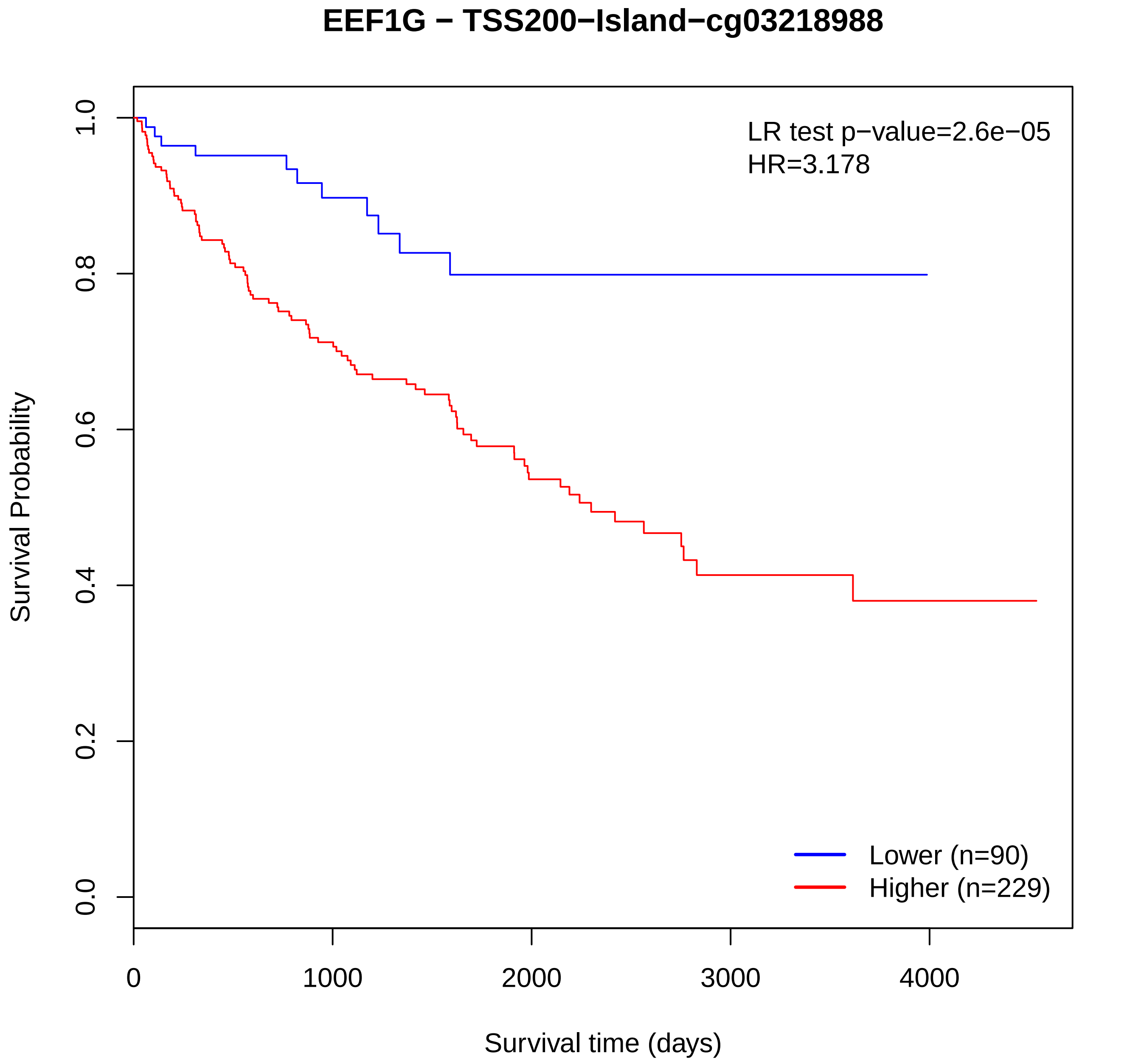
EEF1G − TSS200−Island−cg03218988
LR test p−value=2.6e−05
HR=3.178
Survival Probability
Survival time (days)
0.0
0.2
0.4
0.6
0.8
1.0
0
1000
2000
3000
4000
Lower (n=90)
Higher (n=229)

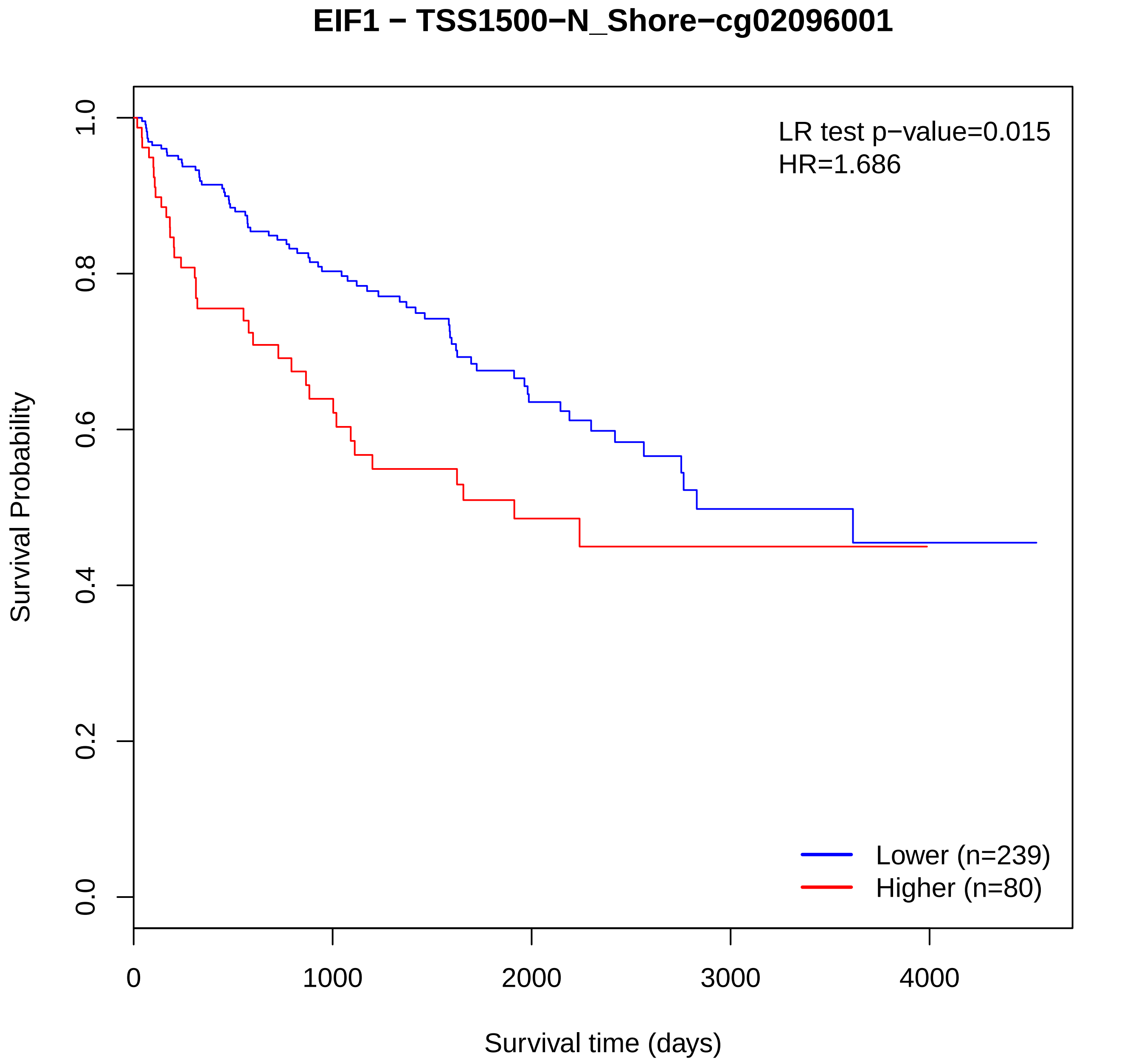

EIF1 − TSS1500−N_Shore−cg02096001
LR test p−value=0.015
HR=1.686
Survival Probability
0.0
0.2
0.4
0.6
0.8
1.0
0
1000
2000
3000
4000
Survival time (days)
Lower (n=239)
Higher (n=80)

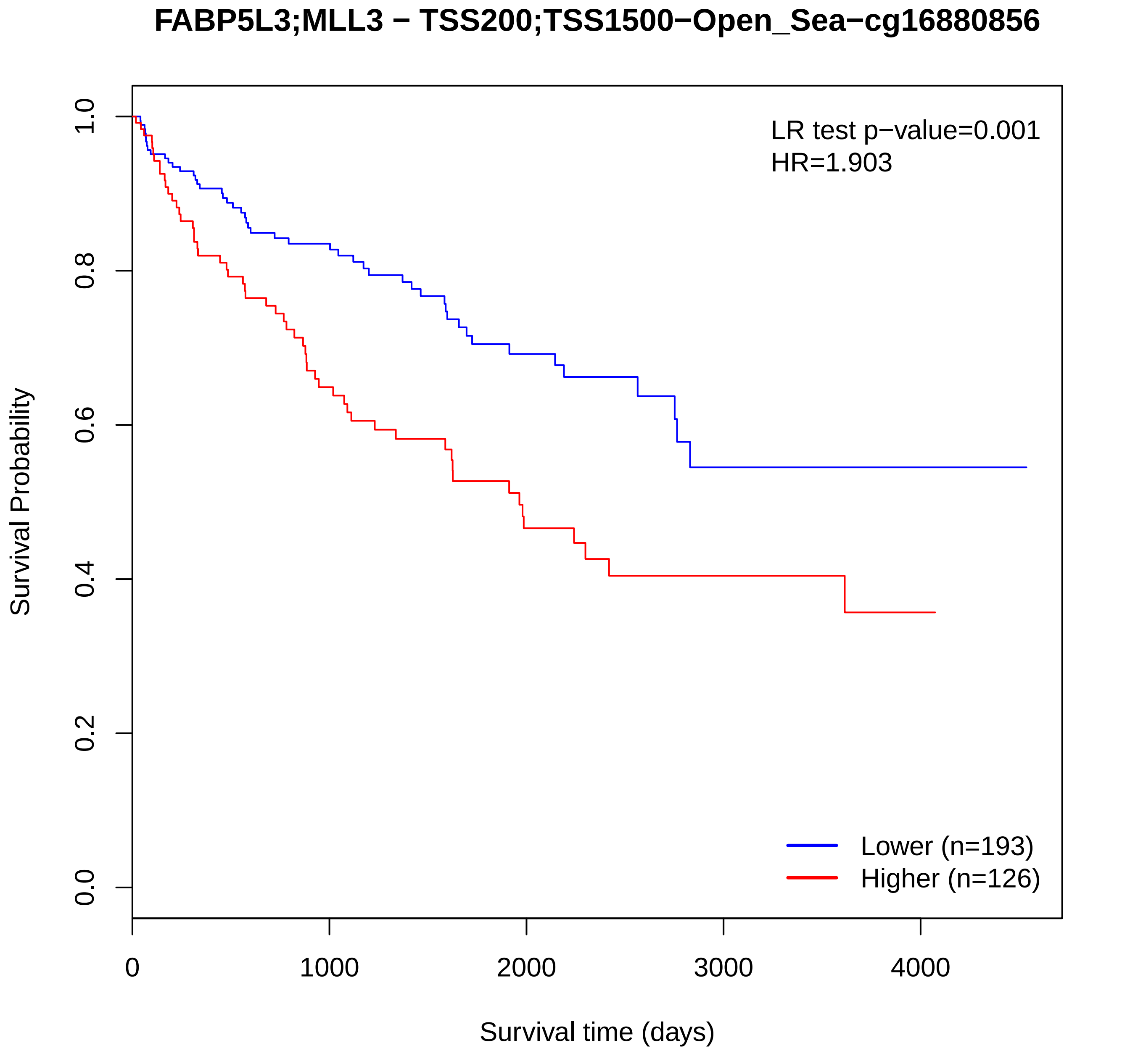
FABP5L3;MLL3 − TSS200;TSS1500−Open_Sea−cg16880856
LR test p−value=0.001
HR=1.903
Lower (n=193)
Higher (n=126)
Survival Probability
0.0
0.2
0.4
0.6
0.8
1.0
0
1000
2000
3000
4000
Survival time (days)

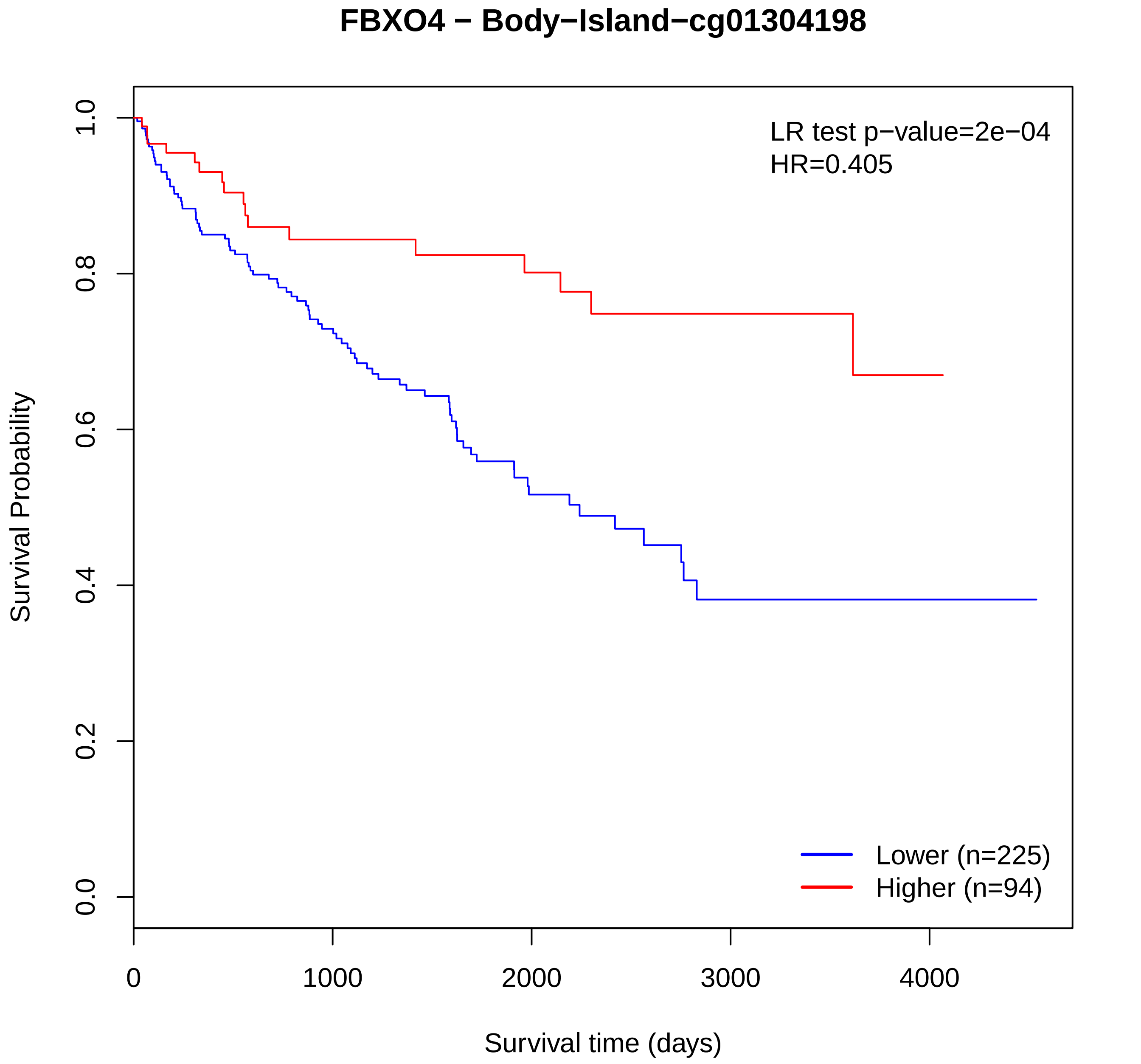
FBXO4 − Body−Island−cg01304198
LR test p−value=2e−04
HR=0.405
Lower (n=225)
Higher (n=94)
Survival Probability
0.0
0.2
0.4
0.6
0.8
1.0
0
1000
2000
3000
4000
Survival time (days)

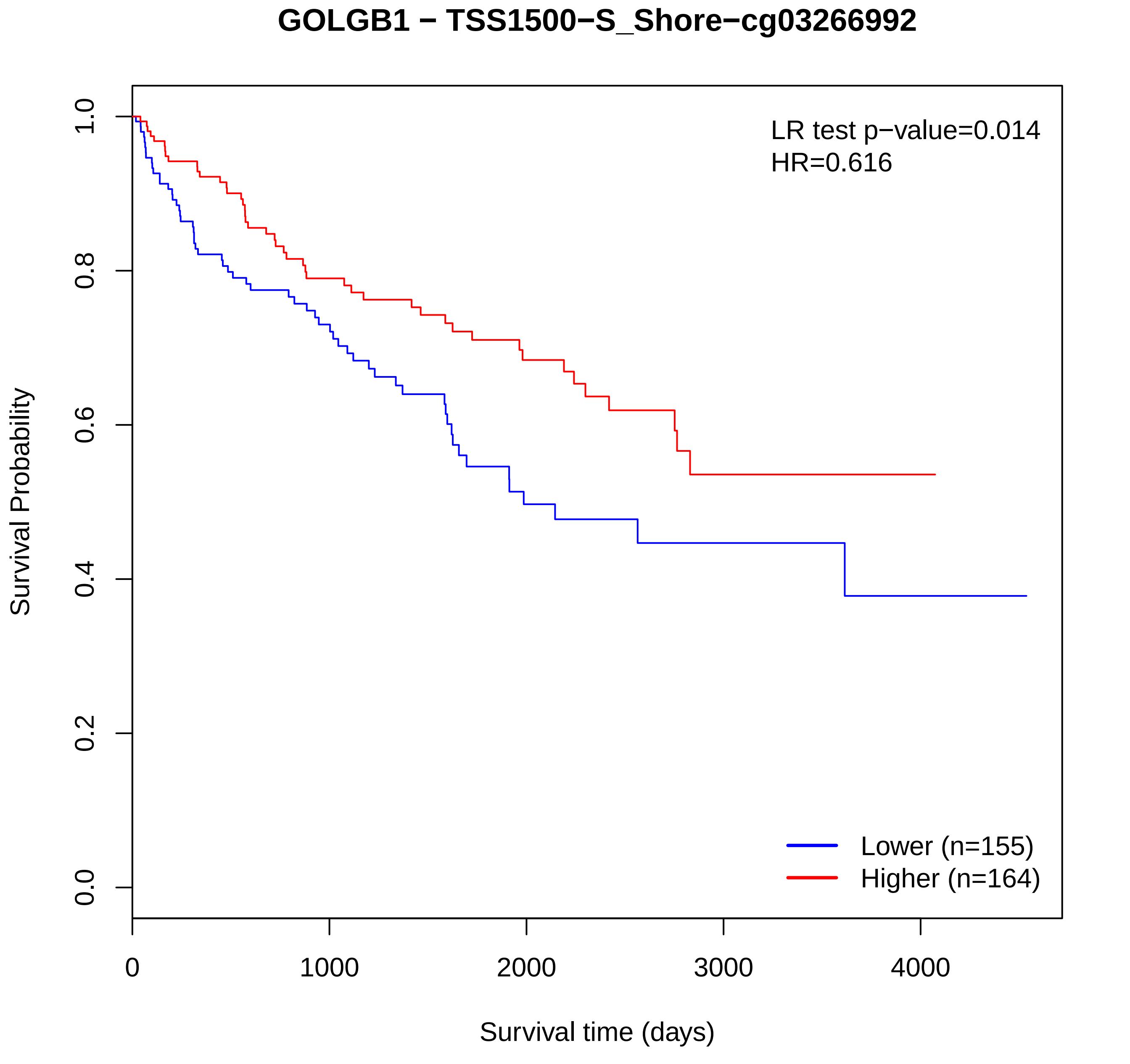
GOLGB1 − TSS1500−S_Shore−cg03266992
LR test p−value=0.014
HR=0.616
Lower (n=155)
Higher (n=164)
Survival Probability
Survival time (days)
0.0
0.2
0.4
0.6
0.8
1.0
0
1000
2000
3000
4000

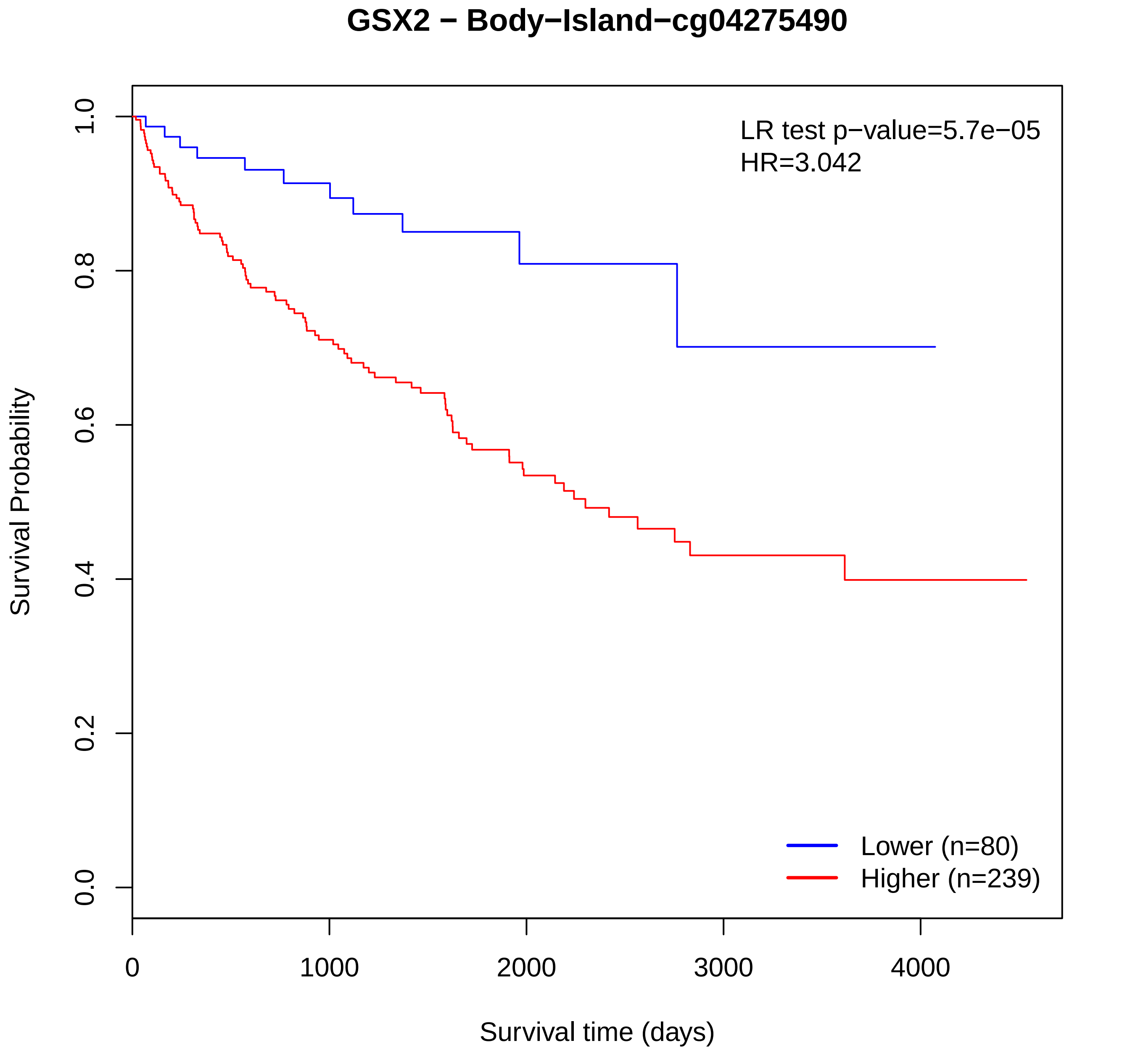

GSX2 − Body−Island−cg04275490
LR test p−value=5.7e−05
HR=3.042
Lower (n=80)
Higher (n=239)
Survival Probability
0.0
0.2
0.4
0.6
0.8
1.0
0
1000
2000
3000
4000
Survival time (days)

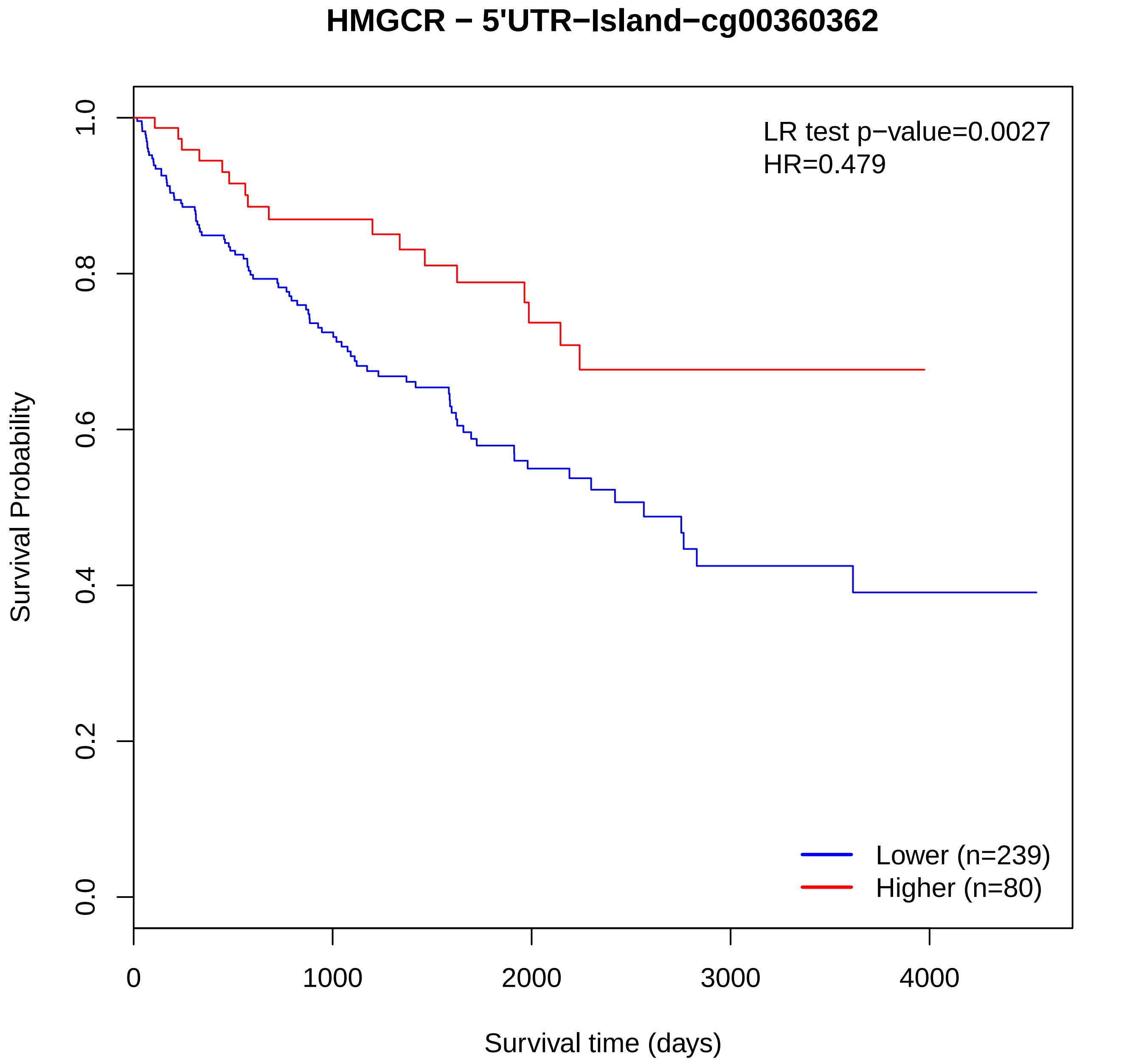
HMGCR − 5'UTR−Island−cg00360362
LR test p−value=0.0027
HR=0.479
Lower (n=239)
Higher (n=80)
Survival Probability
0.0
0.2
0.4
0.6
0.8
1.0
0
1000
2000
3000
4000
Survival time (days)

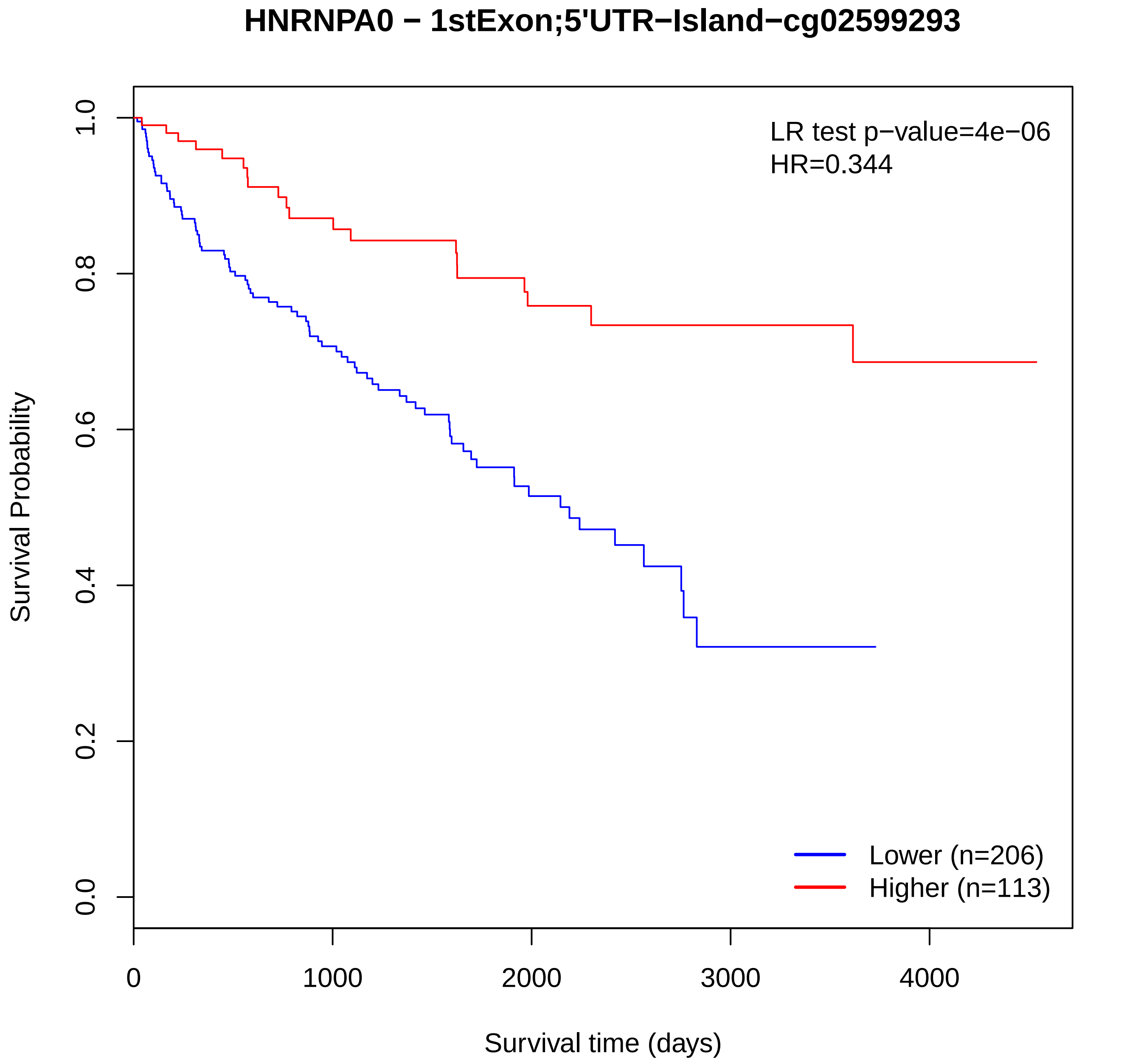
HNRNPA0 − 1stExon;5'UTR−Island−cg02599293
LR test p−value=4e−06
HR=0.344
Lower (n=206)
Higher (n=113)
Survival Probability
0.0
0.2
0.4
0.6
0.8
1.0
0
1000
2000
3000
4000
Survival time (days)

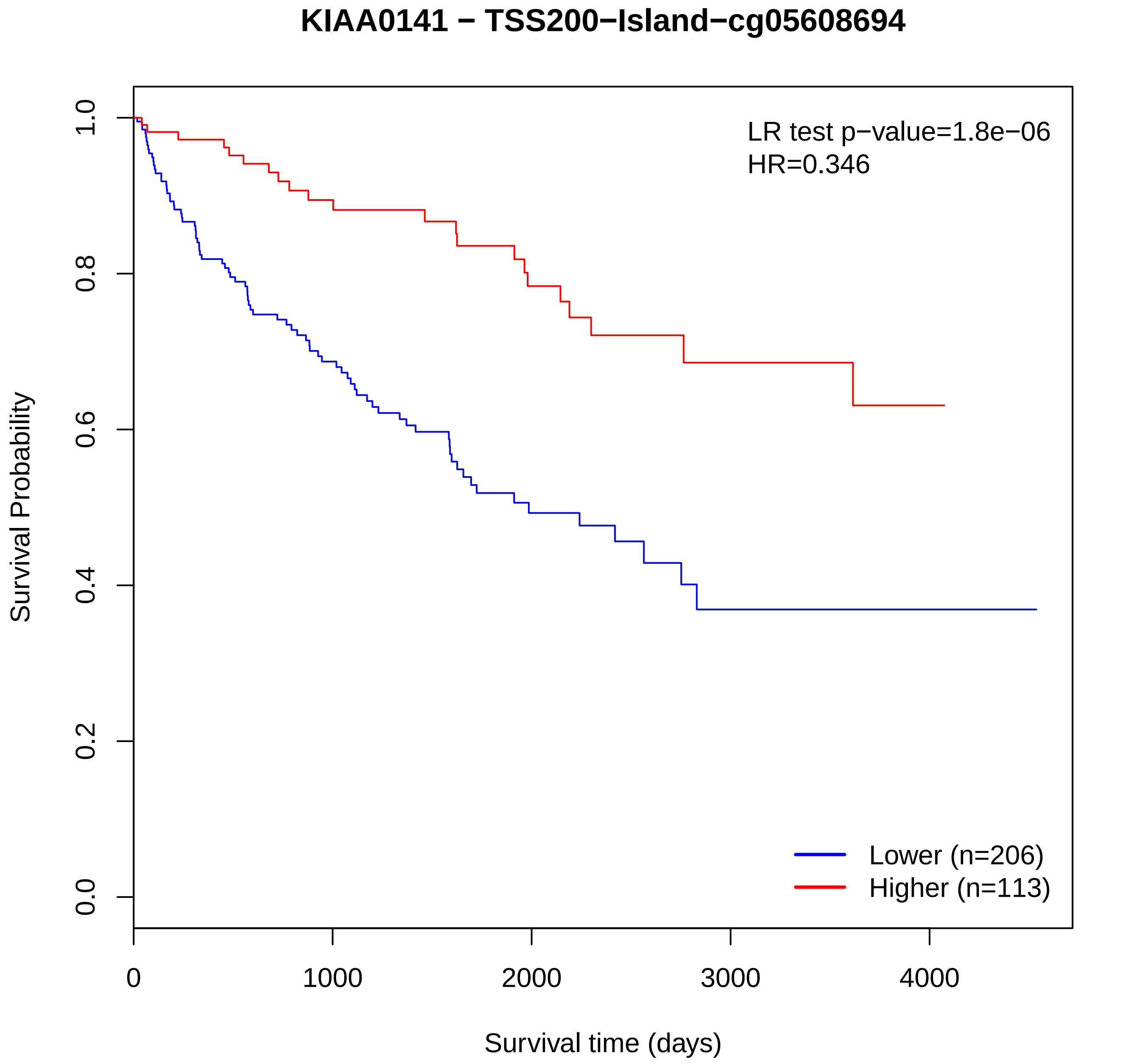
KIAA0141 − TSS200−Island−cg05608694
LR test p−value=1.8e−06
HR=0.346
Lower (n=206)
Higher (n=113)
Survival Probability
Survival time (days)
0.0
0.2
0.4
0.6
0.8
1.0
0
1000
2000
3000
4000

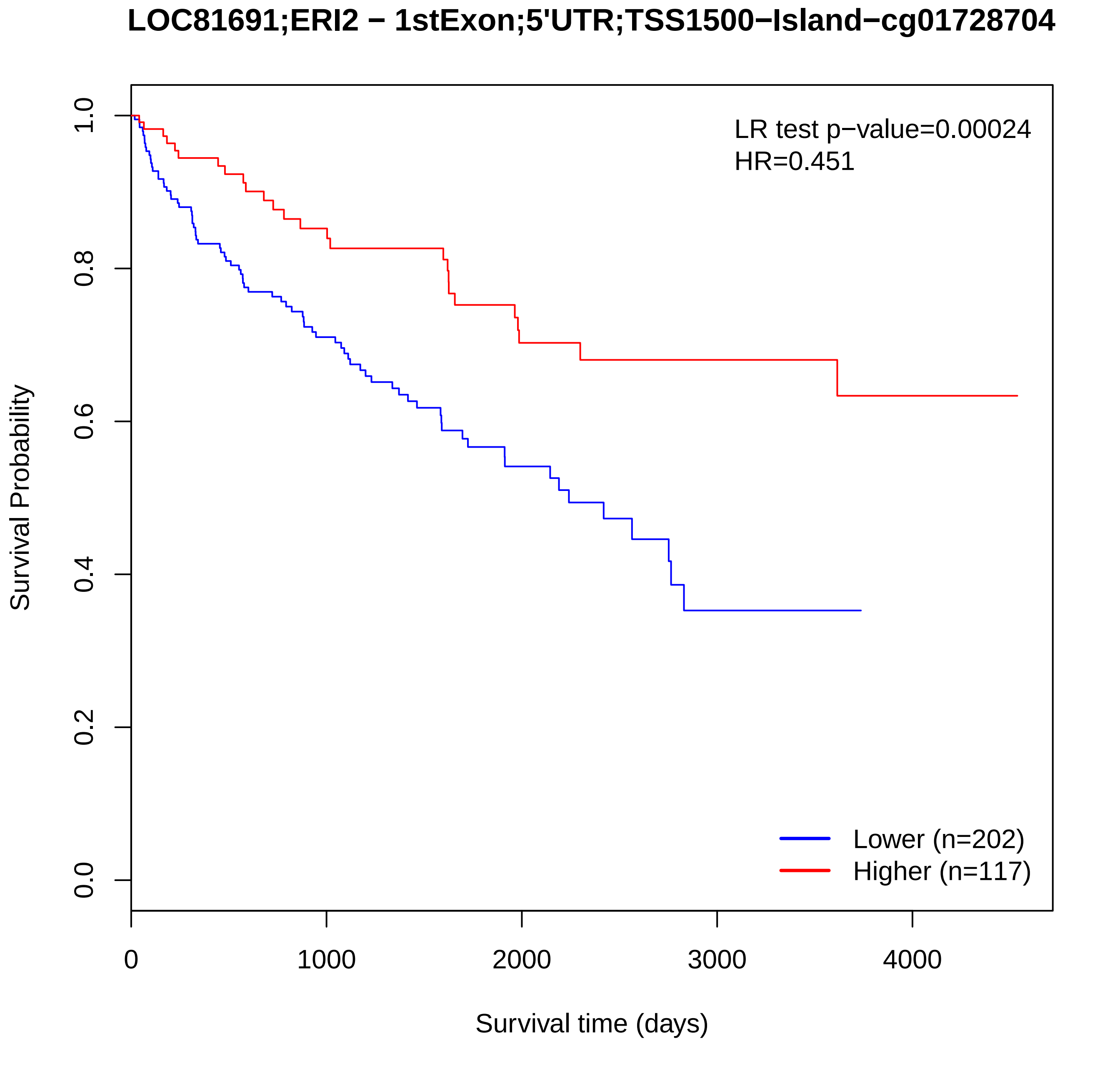

LOC81691;ERI2 − 1stExon;5'UTR;TSS1500−Island−cg01728704
LR test p−value=0.00024
HR=0.451
Lower (n=202)
Higher (n=117)
Survival Probability
0.0
0.2
0.4
0.6
0.8
1.0
0
1000
2000
3000
4000
Survival time (days)

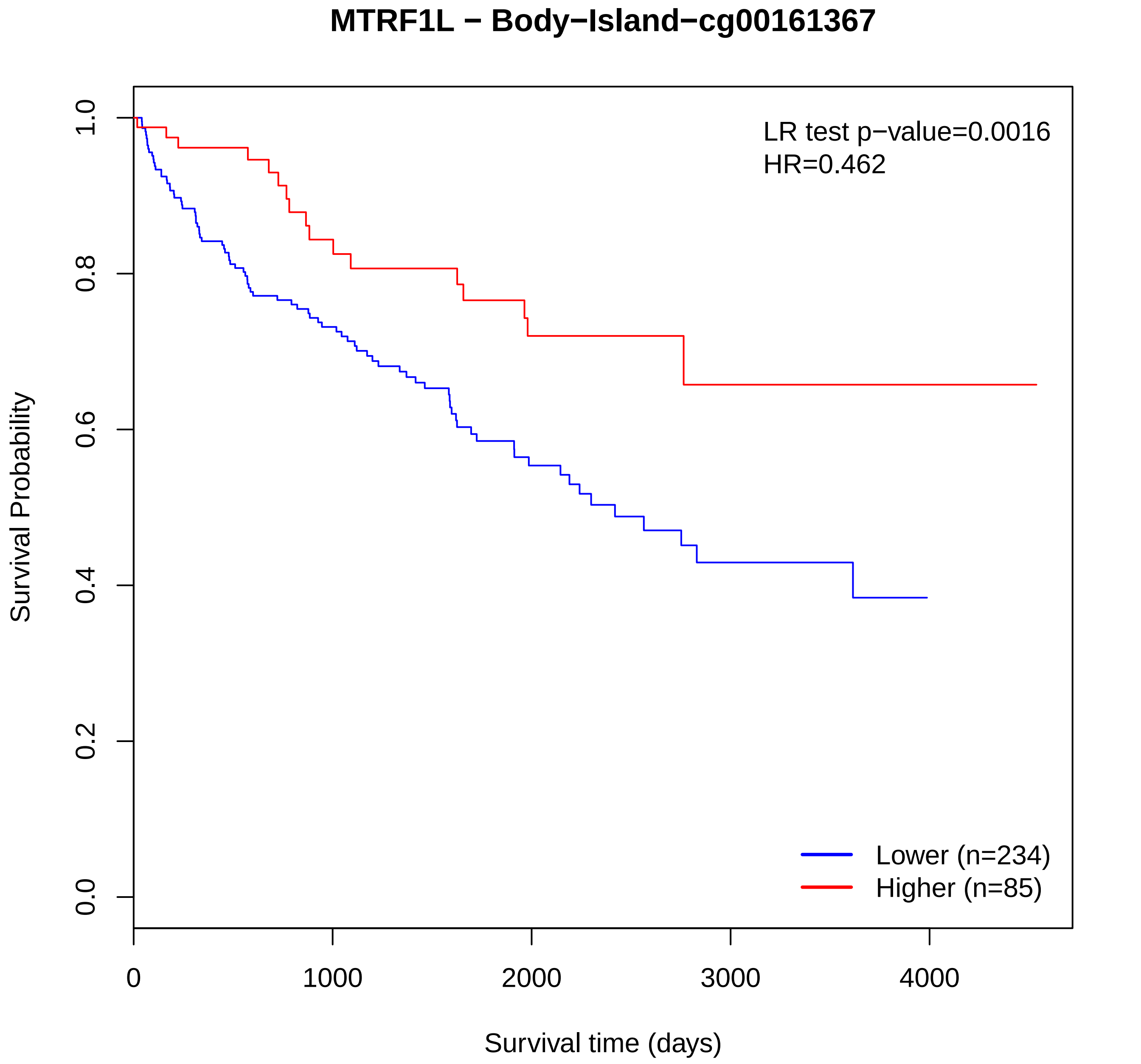
MTRF1L − Body−Island−cg00161367
LR test p−value=0.0016
HR=0.462
Lower (n=234)
Higher (n=85)
Survival Probability
0.0
0.2
0.4
0.6
0.8
1.0
0
1000
2000
3000
4000
Survival time (days)

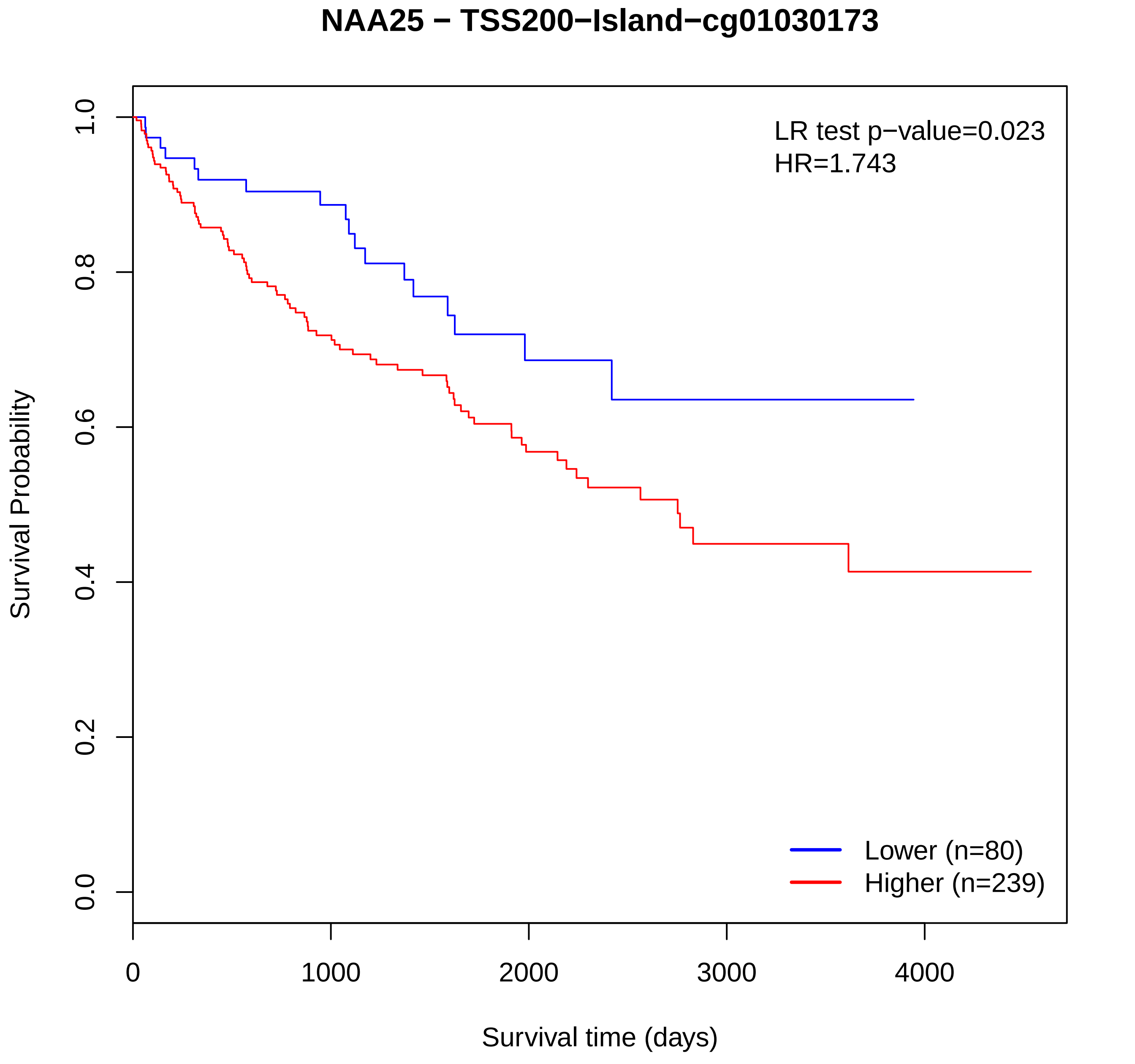

NAA25 − TSS200−Island−cg01030173
LR test p−value=0.023
HR=1.743
Lower (n=80)
Higher (n=239)
Survival Probability
0.0
0.2
0.4
0.6
0.8
1.0
0
1000
2000
3000
4000
Survival time (days)

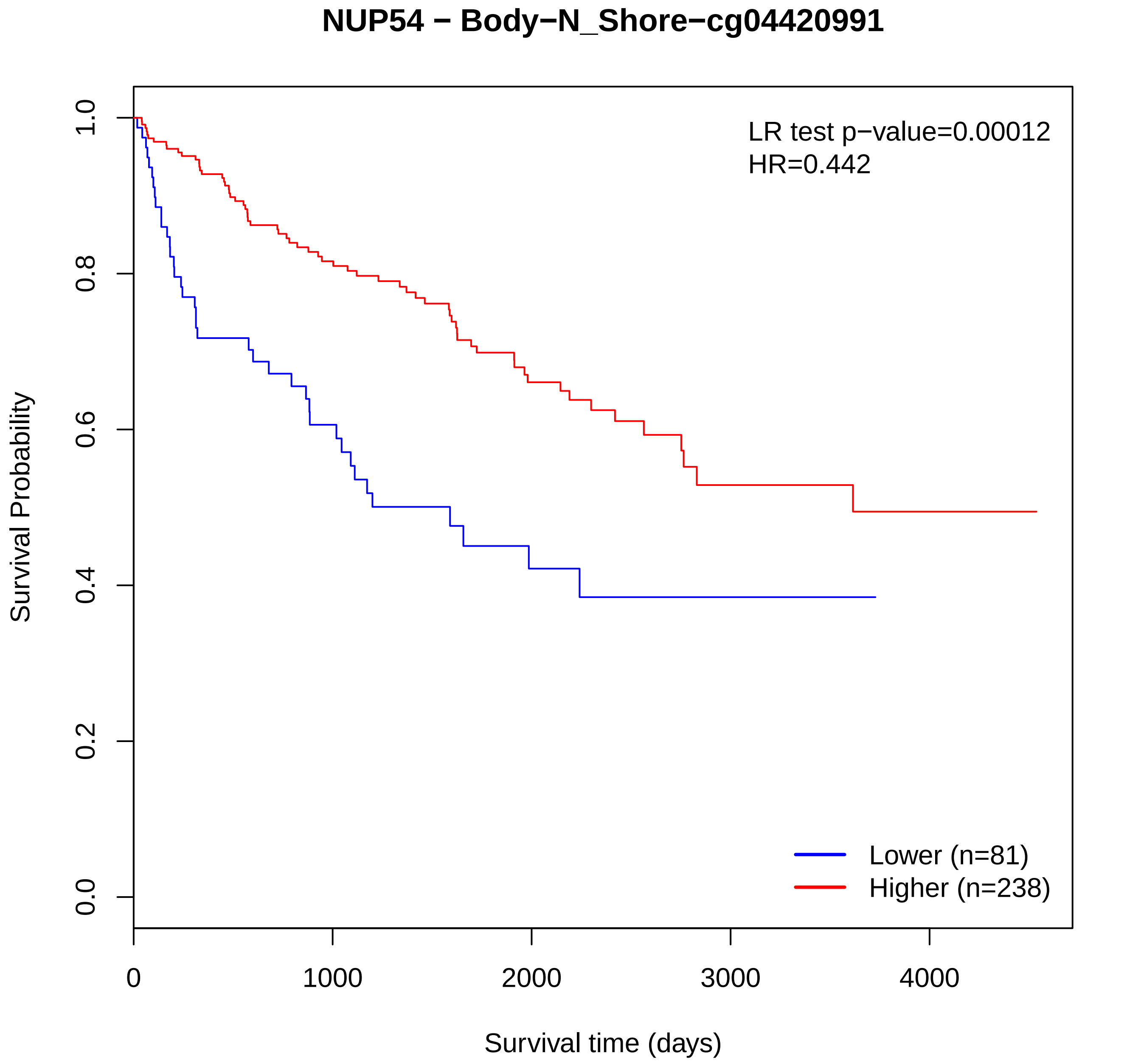
NUP54 − Body−N_Shore−cg04420991
LR test p−value=0.00012
HR=0.442
Lower (n=81)
Higher (n=238)
Survival Probability
0.0
0.2
0.4
0.6
0.8
1.0
0
1000
2000
3000
4000
Survival time (days)

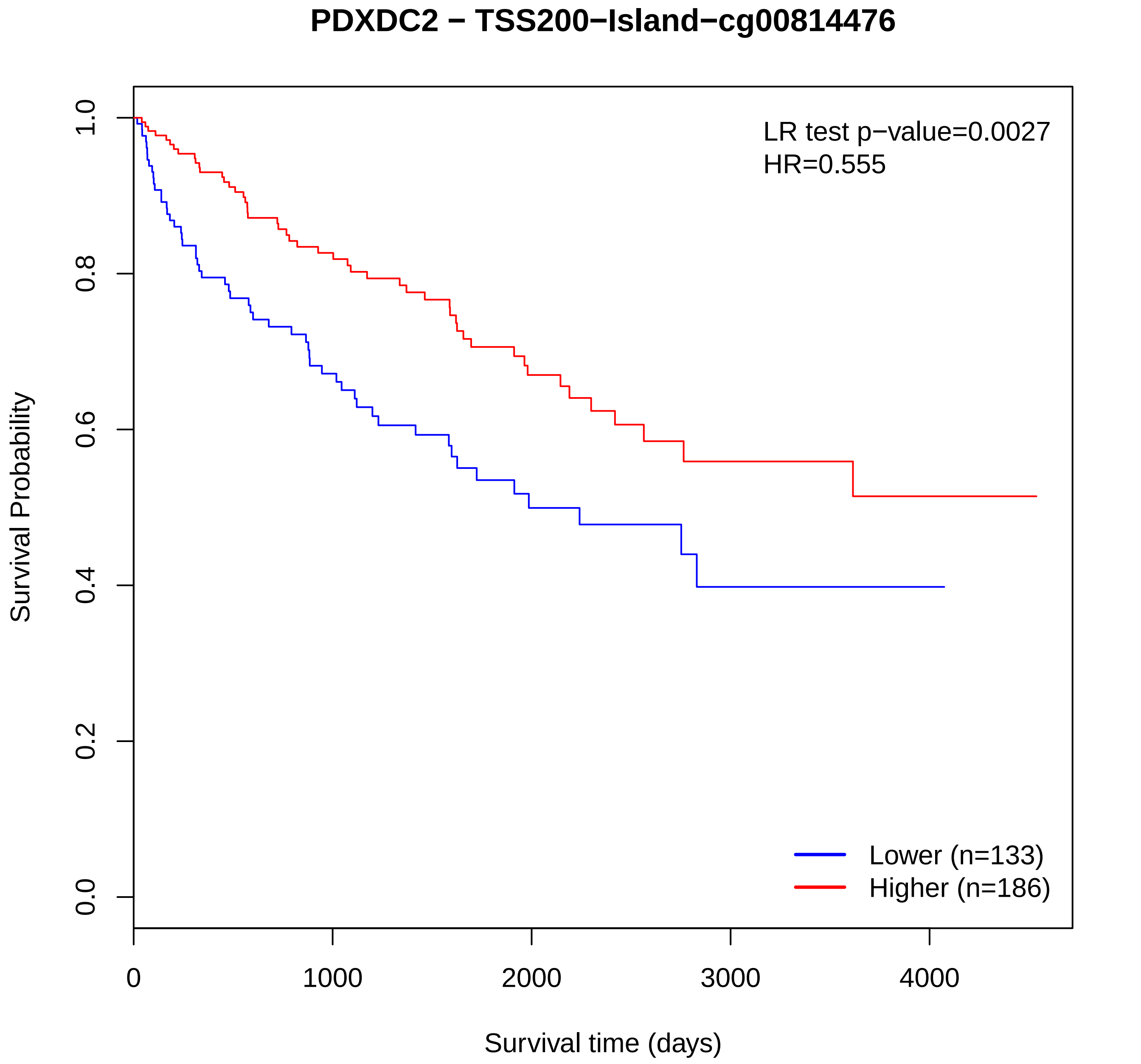

PDXDC2 − TSS200−Island−cg00814476
LR test p−value=0.0027
HR=0.555
Lower (n=133)
Higher (n=186)
Survival Probability
0.0
0.2
0.4
0.6
0.8
1.0
0
1000
2000
3000
4000
Survival time (days)

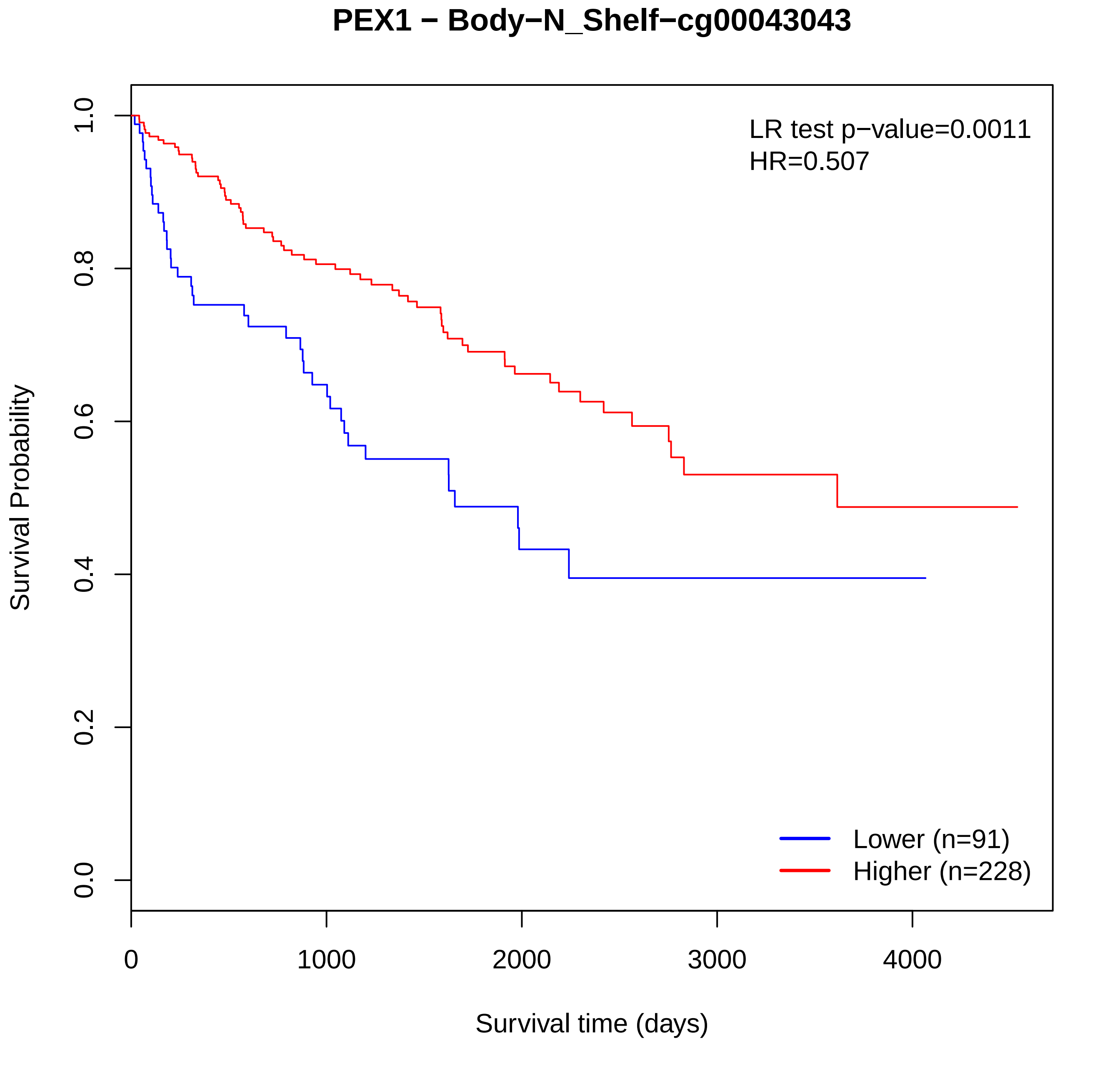
PEX1 − Body−N_Shelf−cg00043043
LR test p−value=0.0011
HR=0.507
Survival Probability
0.0
0.2
0.4
0.6
0.8
1.0
0
1000
2000
3000
4000
Survival time (days)
Lower (n=91)
Higher (n=228)

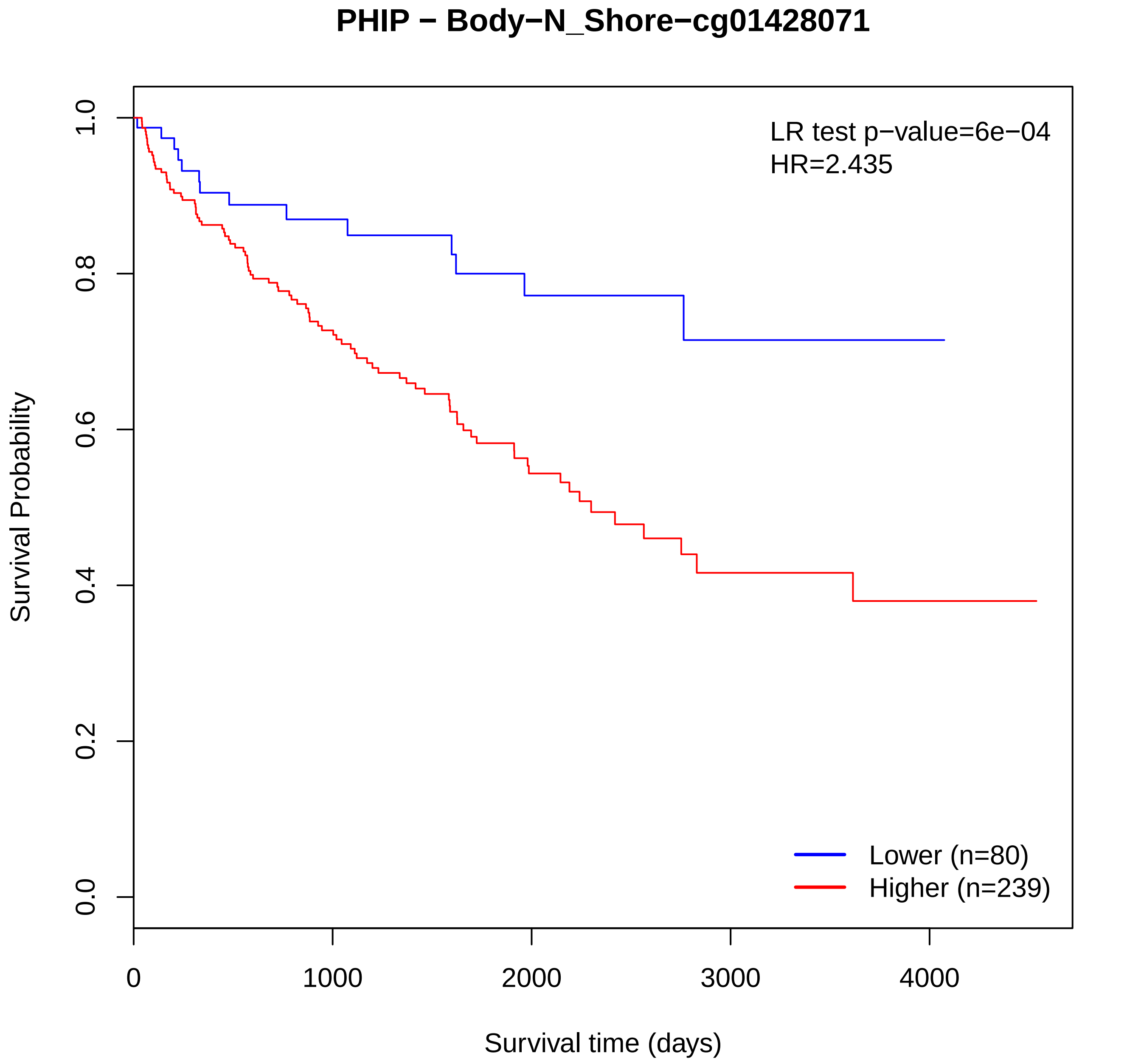

PHIP − Body−N_Shore−cg01428071
LR test p−value=6e−04
HR=2.435
Lower (n=80)
Higher (n=239)
Survival Probability
0.0
0.2
0.4
0.6
0.8
1.0
0
1000
2000
3000
4000
Survival time (days)

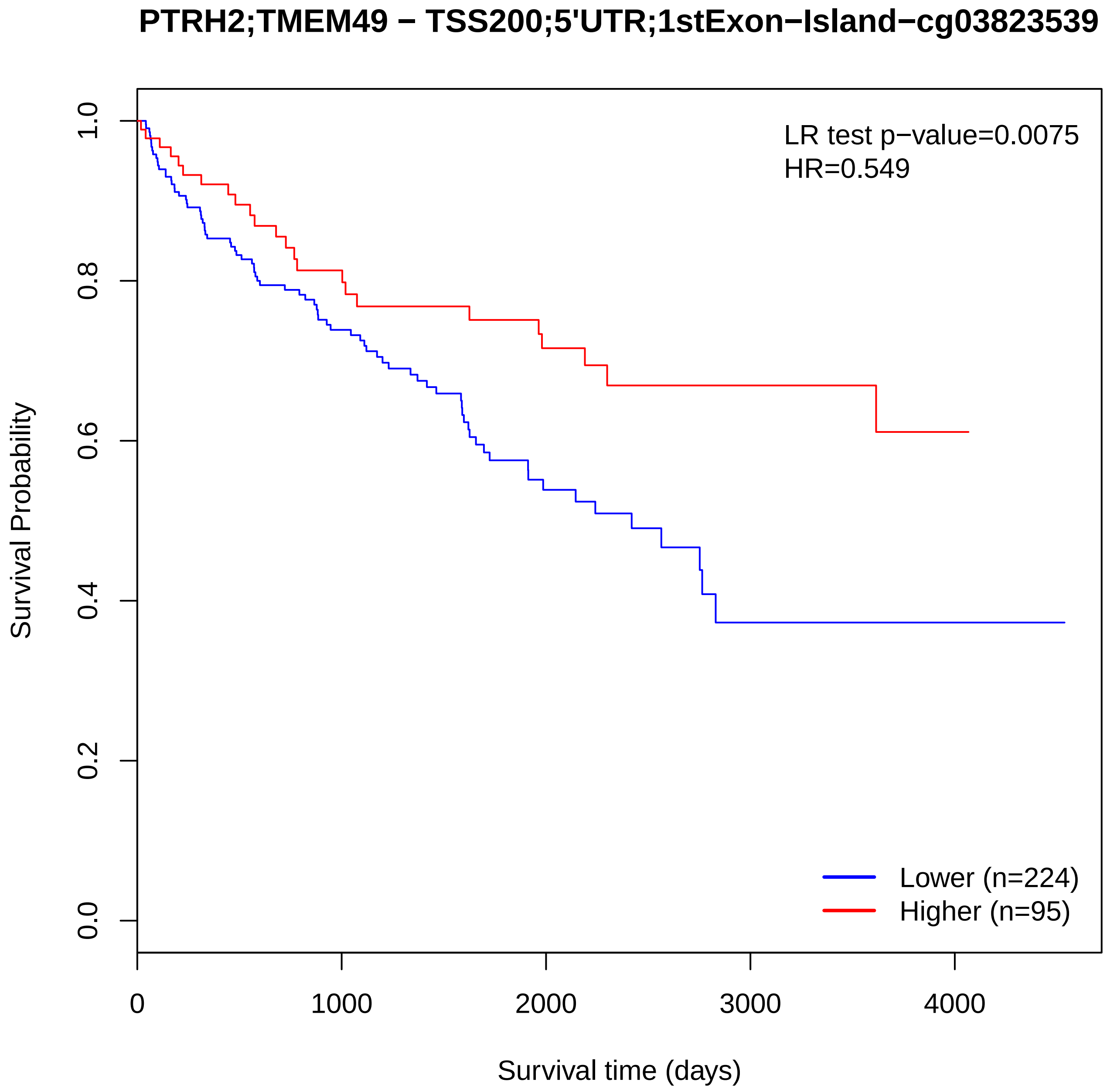
PTRH2;TMEM49 − TSS200;5'UTR;1stExon−Island−cg03823539
LR test p−value=0.0075
HR=0.549
Lower (n=224)
Higher (n=95)
Survival Probability
Survival time (days)
0.0
0.2
0.4
0.6
0.8
1.0
0
1000
2000
3000
4000

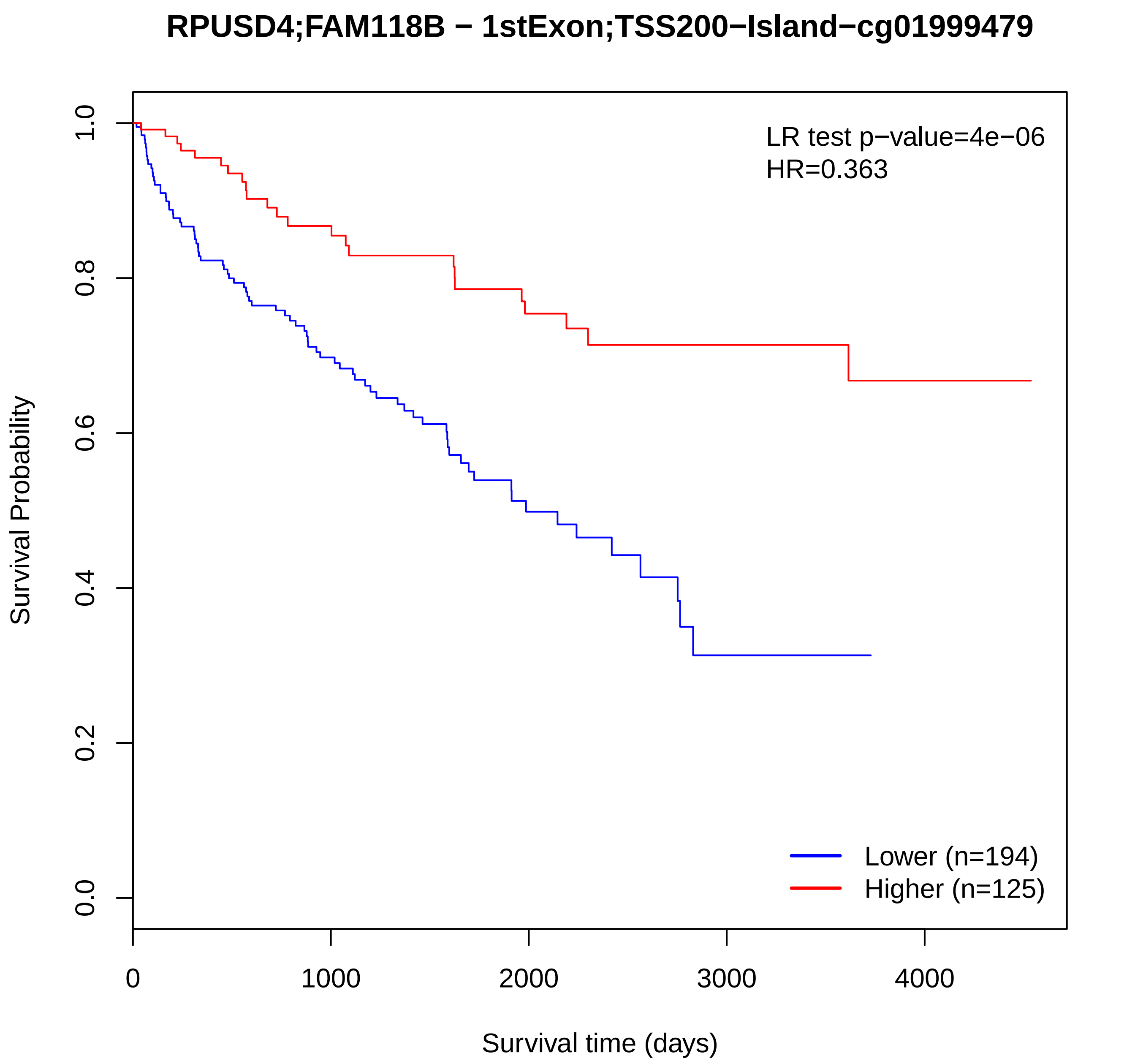
RPUSD4;FAM118B − 1stExon;TSS200−Island−cg01999479
LR test p−value=4e−06
HR=0.363
Lower (n=194)
Higher (n=125)
Survival Probability
0.0
0.2
0.4
0.6
0.8
1.0
0
1000
2000
3000
4000
Survival time (days)

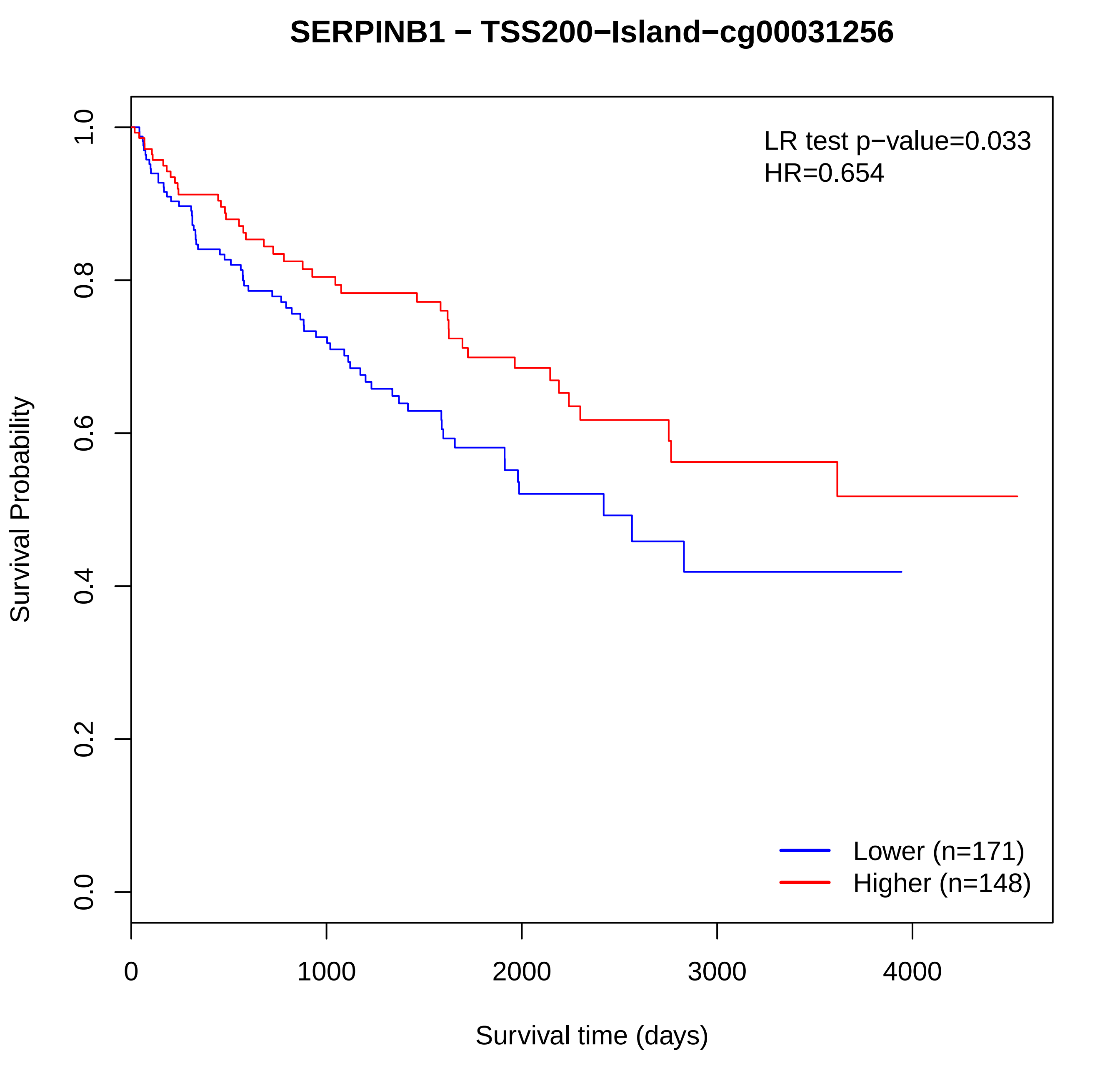
SERPINB1 − TSS200−Island−cg00031256
LR test p−value=0.033
HR=0.654
Lower (n=171)
Higher (n=148)
Survival Probability
0.0
0.2
0.4
0.6
0.8
1.0
0
1000
2000
3000
4000
Survival time (days)

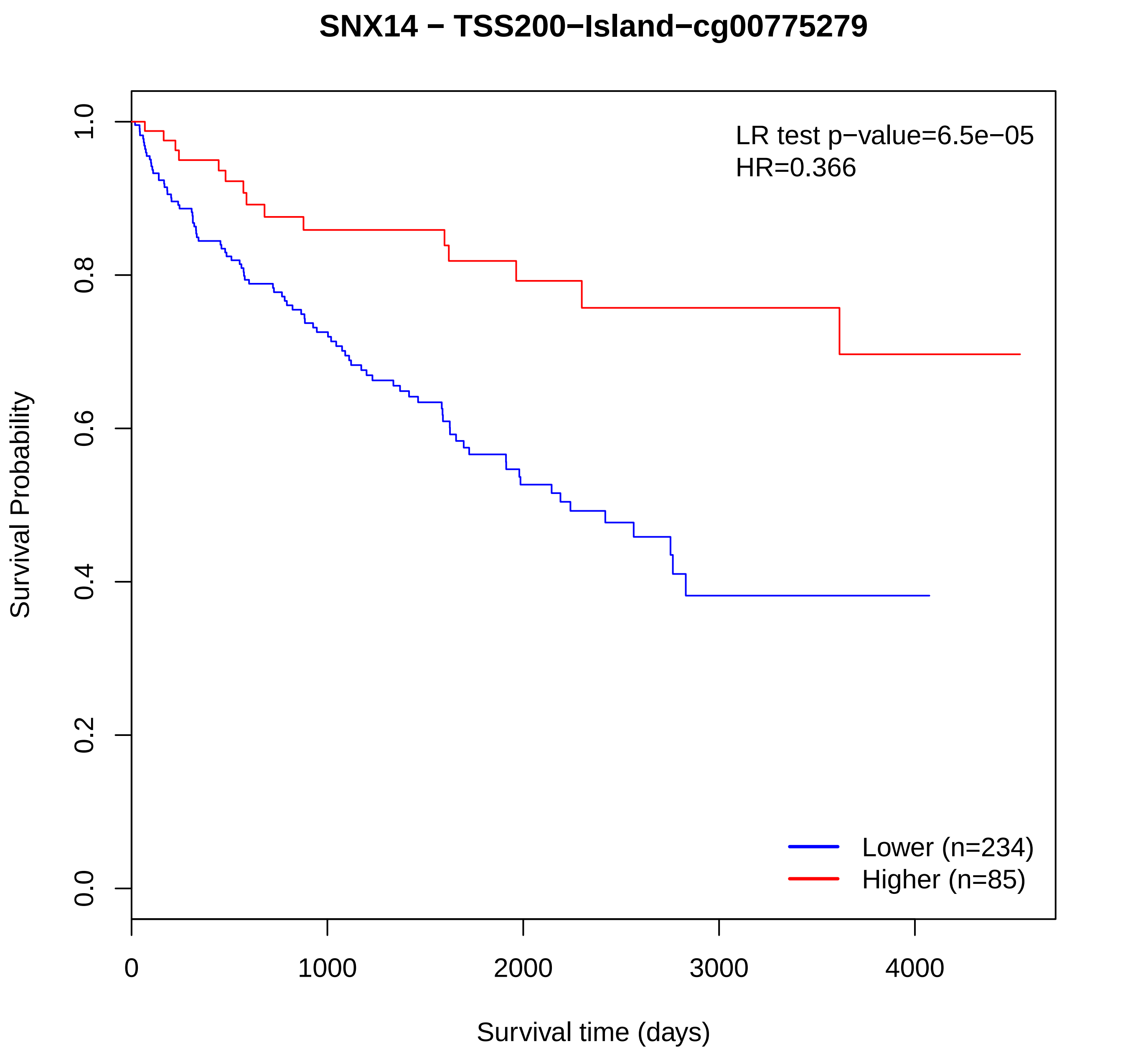
SNX14 − TSS200−Island−cg00775279
LR test p−value=6.5e−05
HR=0.366
Lower (n=234)
Higher (n=85)
Survival Probability
0.0
0.2
0.4
0.6
0.8
1.0
0
1000
2000
3000
4000
Survival time (days)

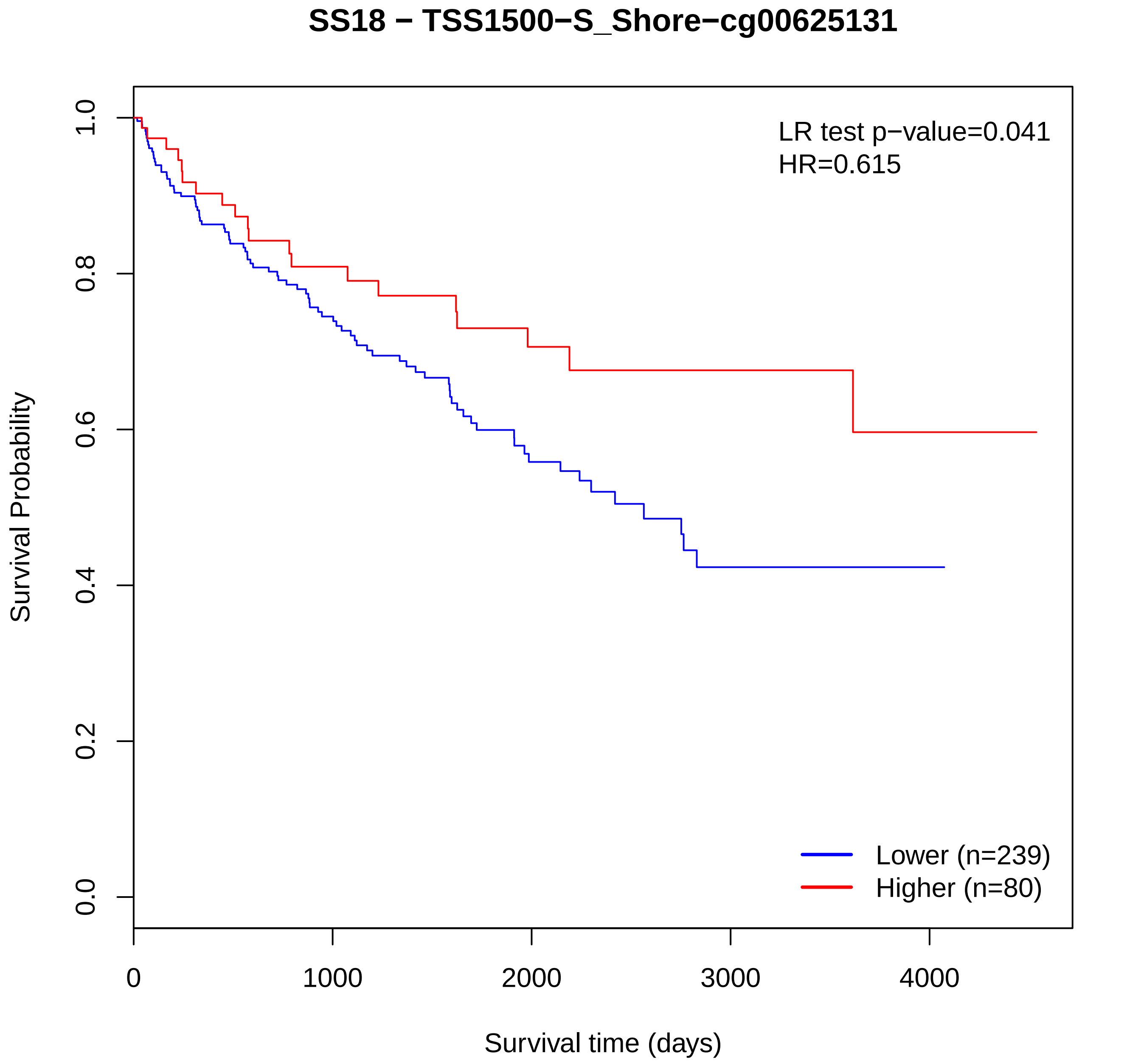
SS18 − TSS1500−S_Shore−cg00625131
LR test p−value=0.041
HR=0.615
Lower (n=239)
Higher (n=80)
Survival Probability
0.0
0.2
0.4
0.6
0.8
1.0
0
1000
2000
3000
4000
Survival time (days)

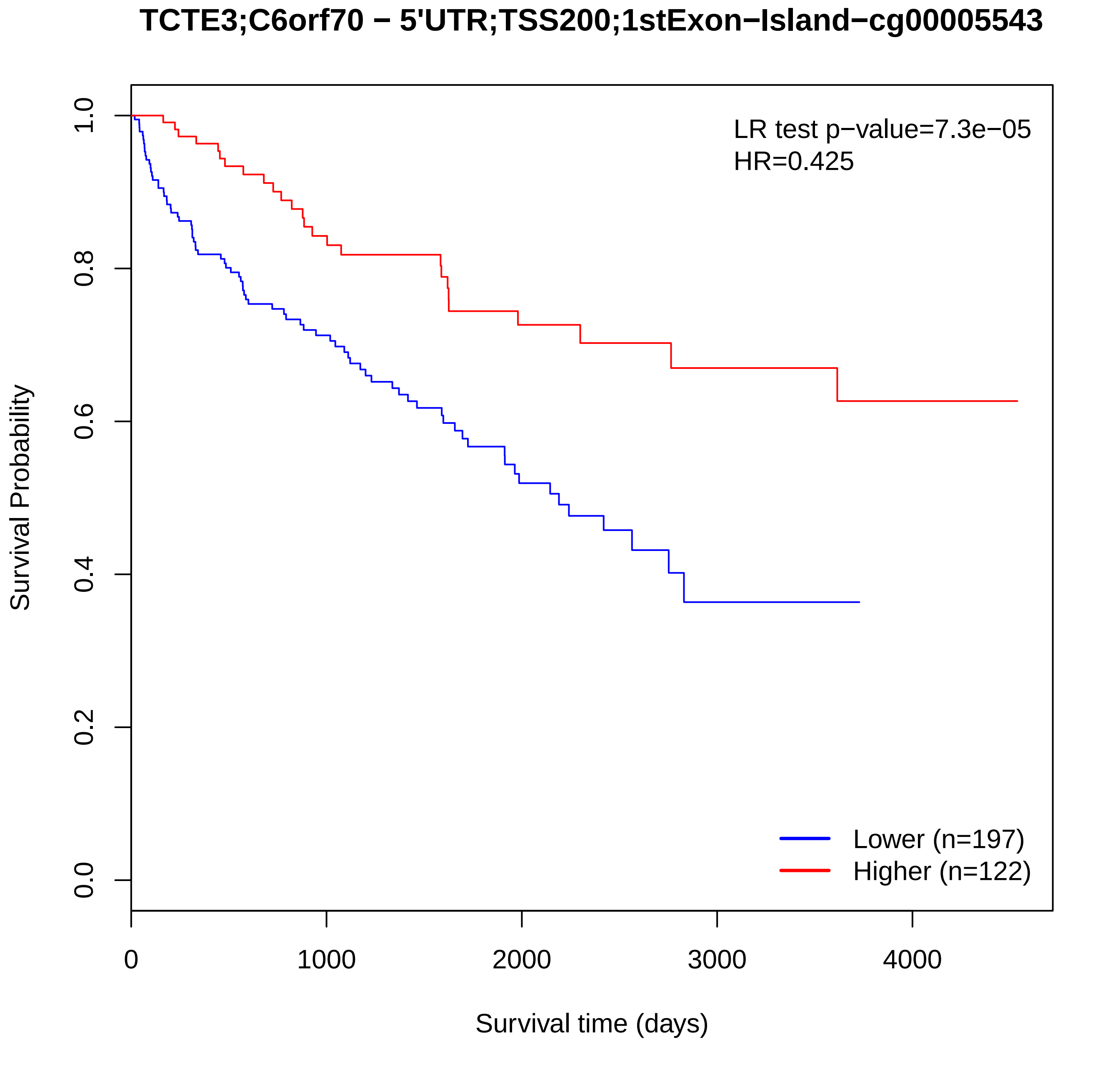

TCTE3;C6orf70 − 5'UTR;TSS200;1stExon−Island−cg00005543
LR test p−value=7.3e−05
HR=0.425
Lower (n=197)
Higher (n=122)
Survival Probability
0.0
0.2
0.4
0.6
0.8
1.0
0
1000
2000
3000
4000
Survival time (days)

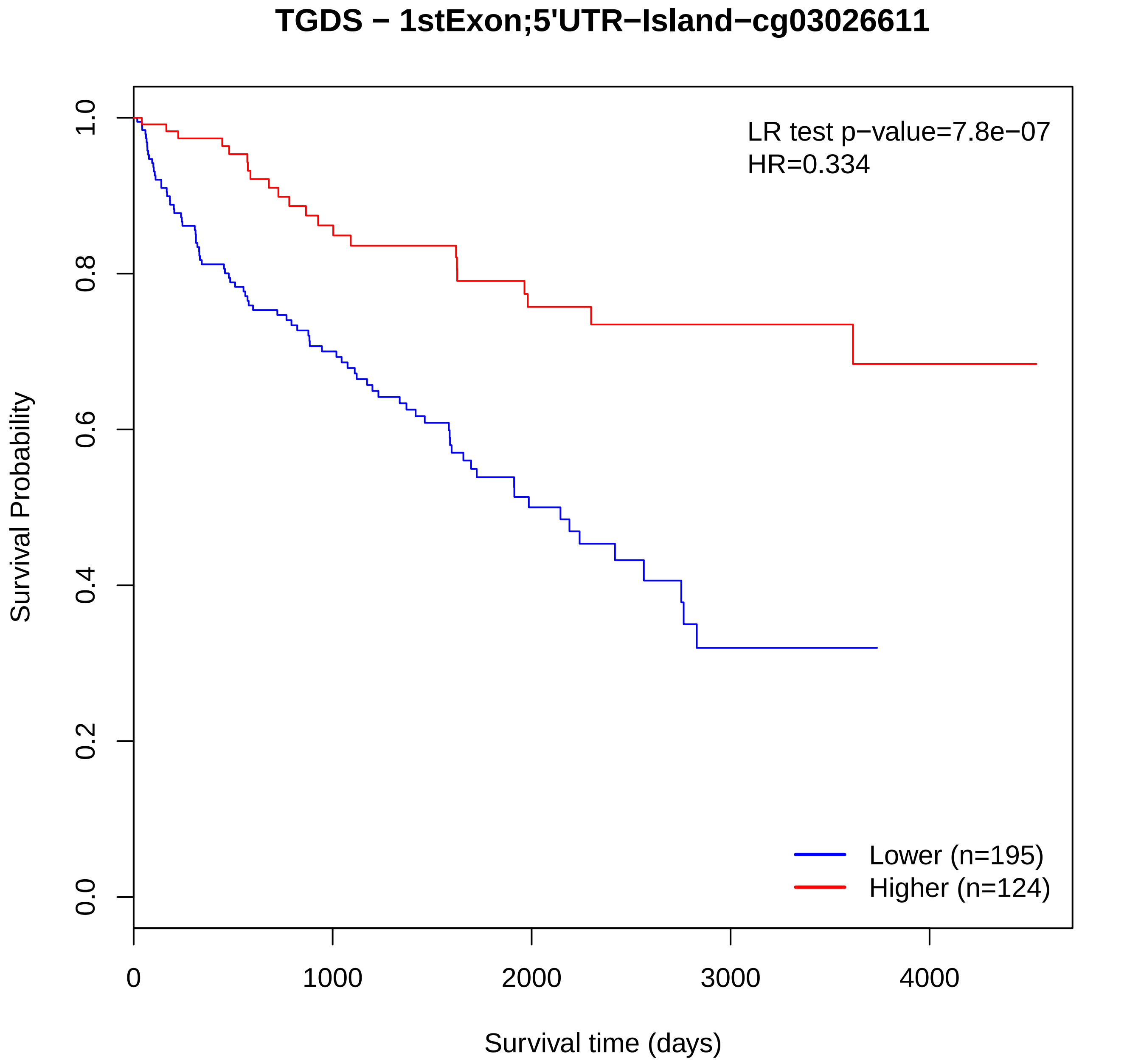
TGDS − 1stExon;5'UTR−Island−cg03026611
LR test p−value=7.8e−07
HR=0.334
Lower (n=195)
Higher (n=124)
Survival Probability
0.0
0.2
0.4
0.6
0.8
1.0
0
1000
2000
3000
4000
Survival time (days)

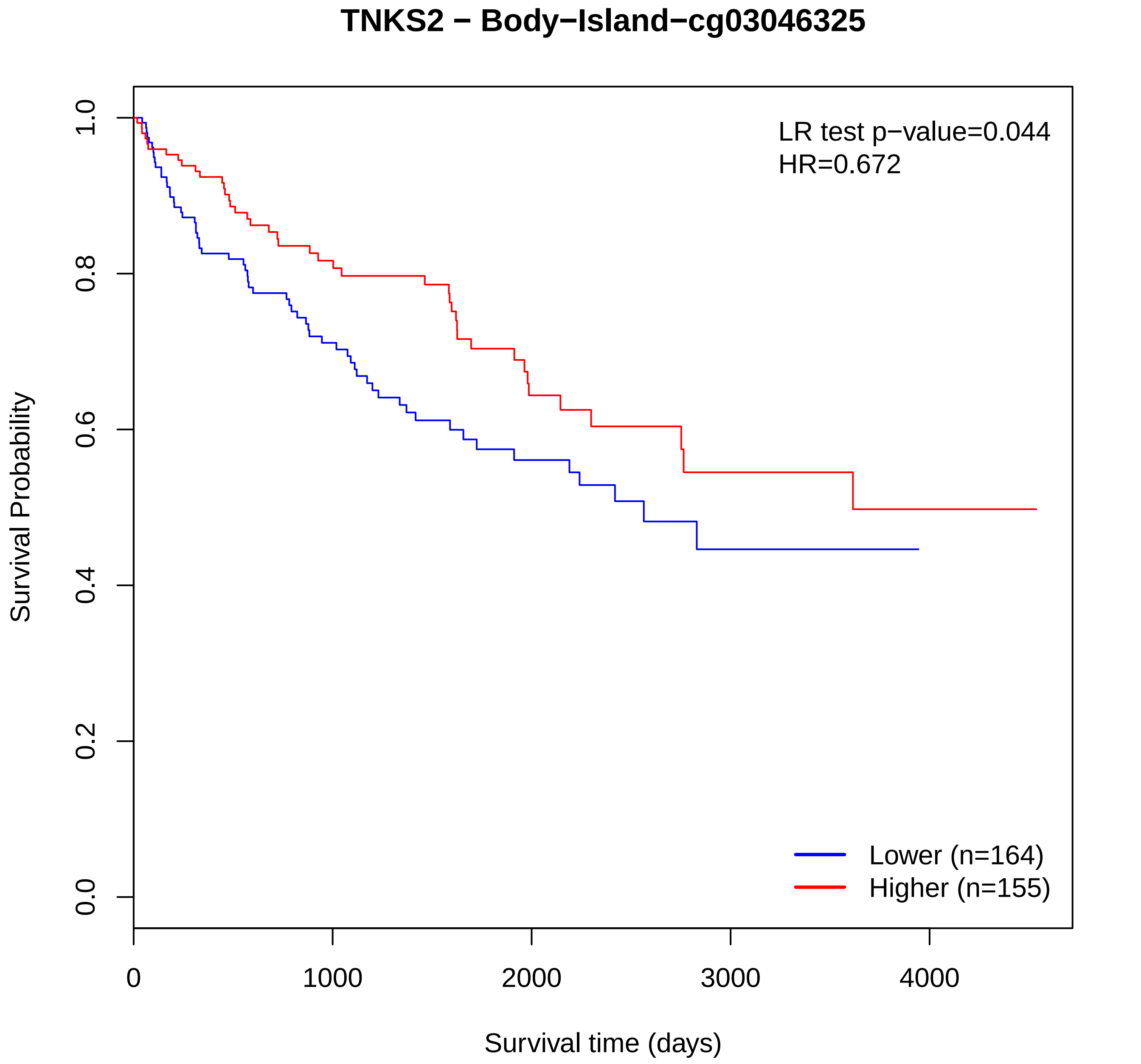

TNKS2 − Body−Island−cg03046325
LR test p−value=0.044
HR=0.672
Lower (n=164)
Higher (n=155)
Survival Probability
0.0
0.2
0.4
0.6
0.8
1.0
0
1000
2000
3000
4000
Survival time (days)

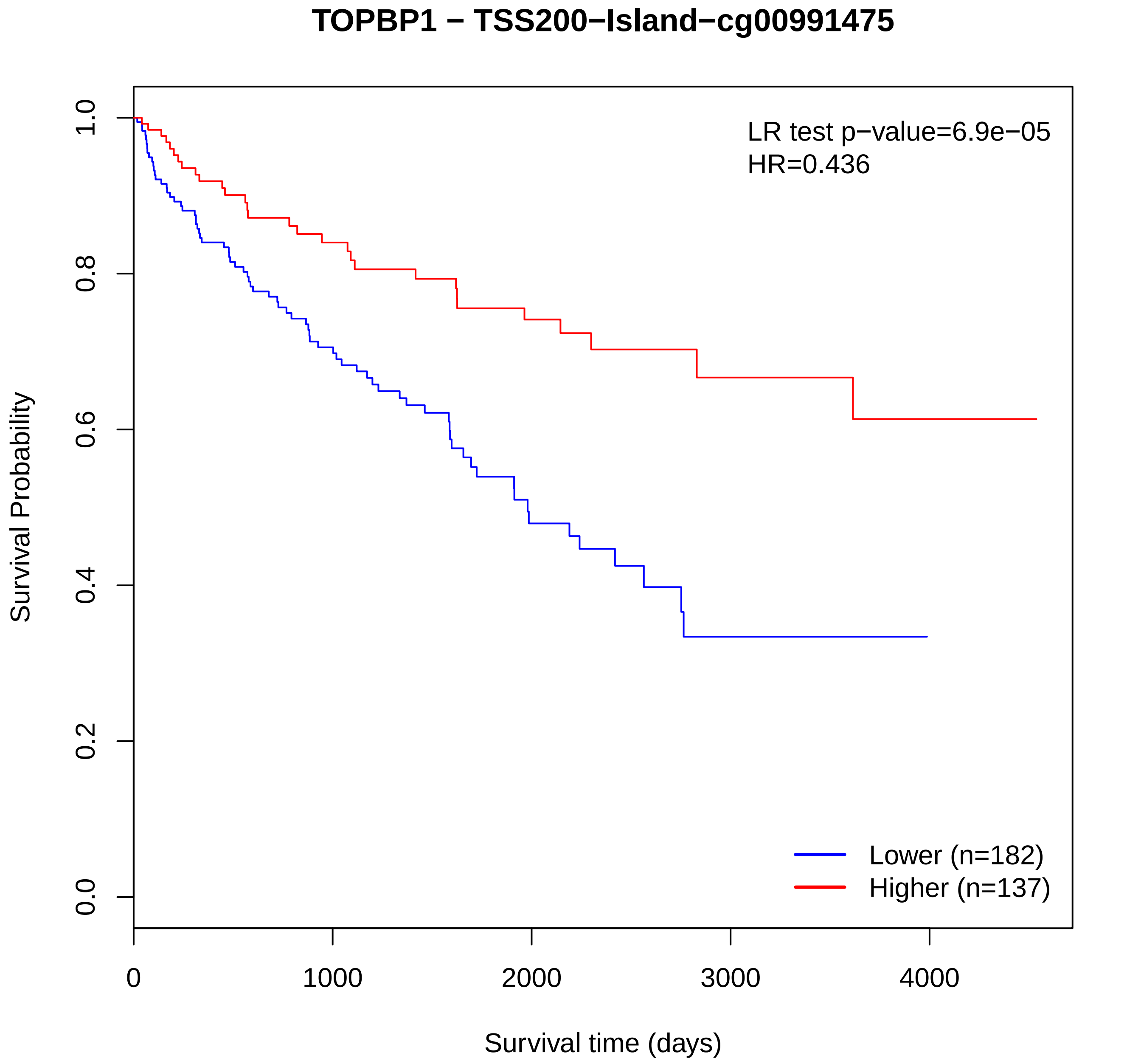
TOPBP1 − TSS200−Island−cg00991475
LR test p−value=6.9e−05
HR=0.436
Lower (n=182)
Higher (n=137)
Survival Probability
0.0
0.2
0.4
0.6
0.8
1.0
0
1000
2000
3000
4000
Survival time (days)

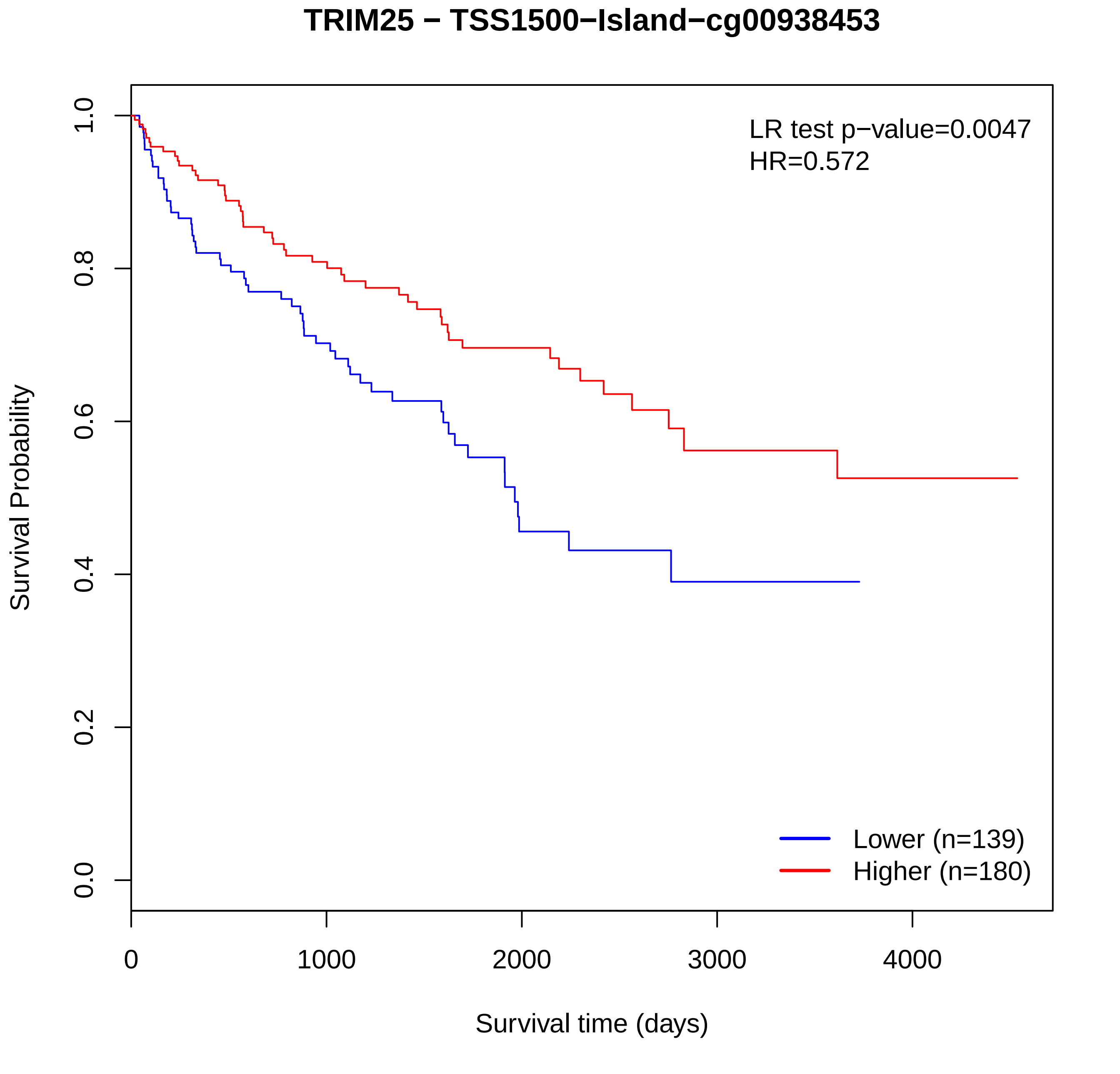

TRIM25 − TSS1500−Island−cg00938453
LR test p−value=0.0047
HR=0.572
Lower (n=139)
Higher (n=180)
Survival Probability
Survival time (days)
0.0
0.2
0.4
0.6
0.8
1.0
0
1000
2000
3000
4000

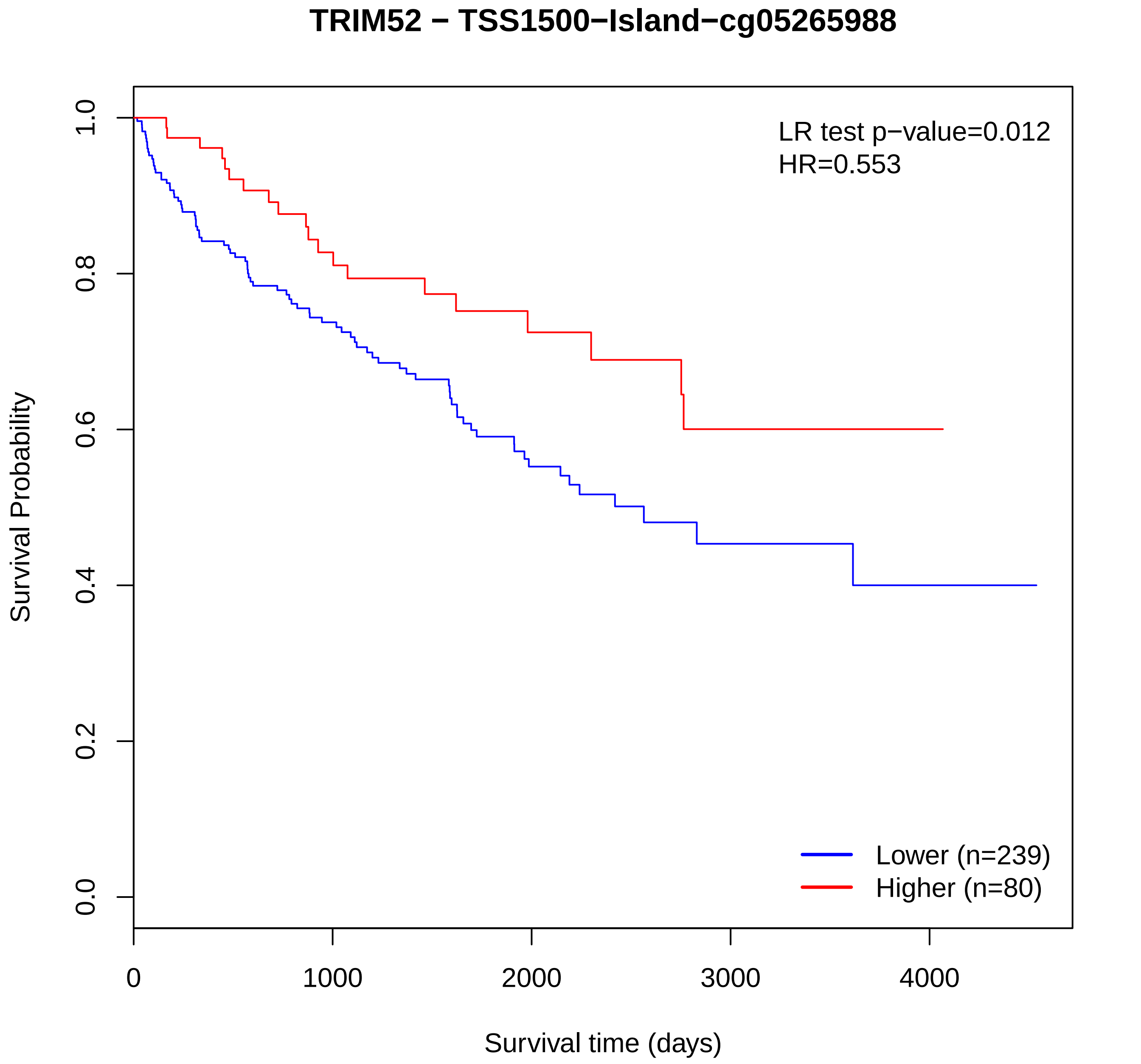

TRIM52 − TSS1500−Island−cg05265988
LR test p−value=0.012
HR=0.553
Lower (n=239)
Higher (n=80)
Survival Probability
0.0
0.2
0.4
0.6
0.8
1.0
0
1000
2000
3000
4000
Survival time (days)

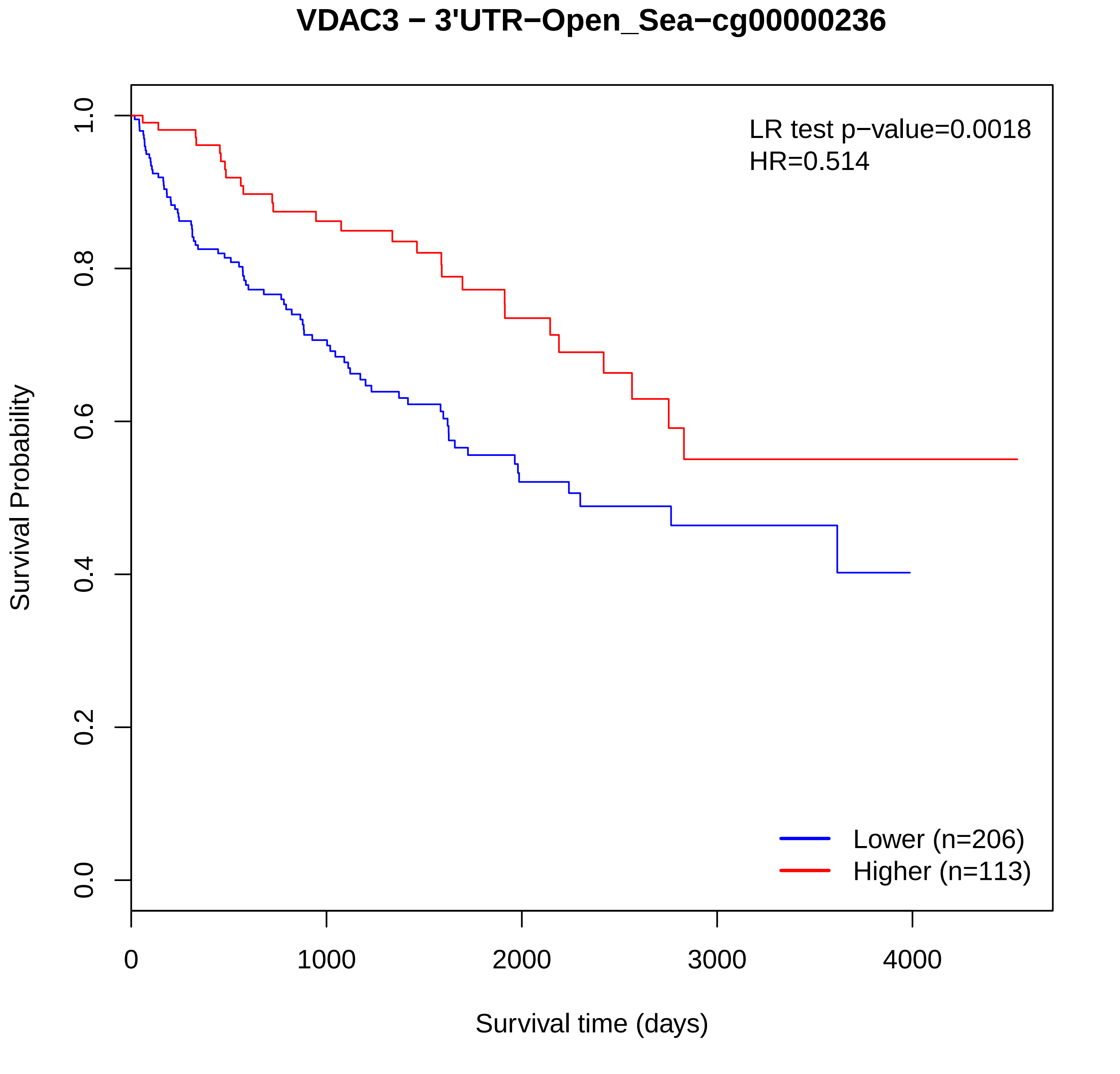
VDAC3 − 3'UTR−Open_Sea−cg00000236
LR test p−value=0.0018
HR=0.514
Lower (n=206)
Higher (n=113)
Survival Probability
0.0
0.2
0.4
0.6
0.8
1.0
0
1000
2000
3000
4000
Survival time (days)

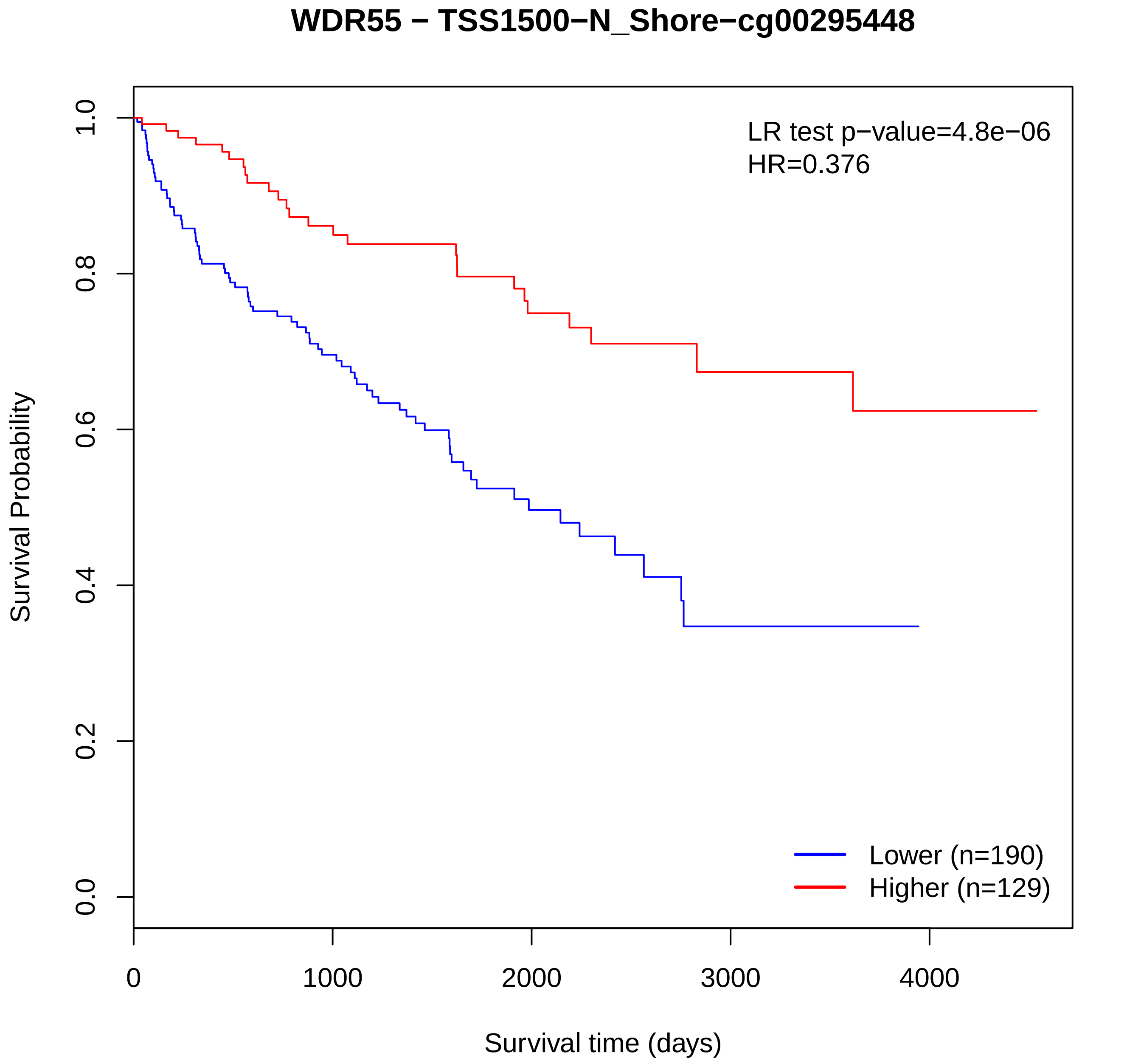

WDR55 − TSS1500−N_Shore−cg00295448
LR test p−value=4.8e−06
HR=0.376
Lower (n=190)
Higher (n=129)
Survival Probability
0.0
0.2
0.4
0.6
0.8
1.0
0
1000
2000
3000
4000
Survival time (days)

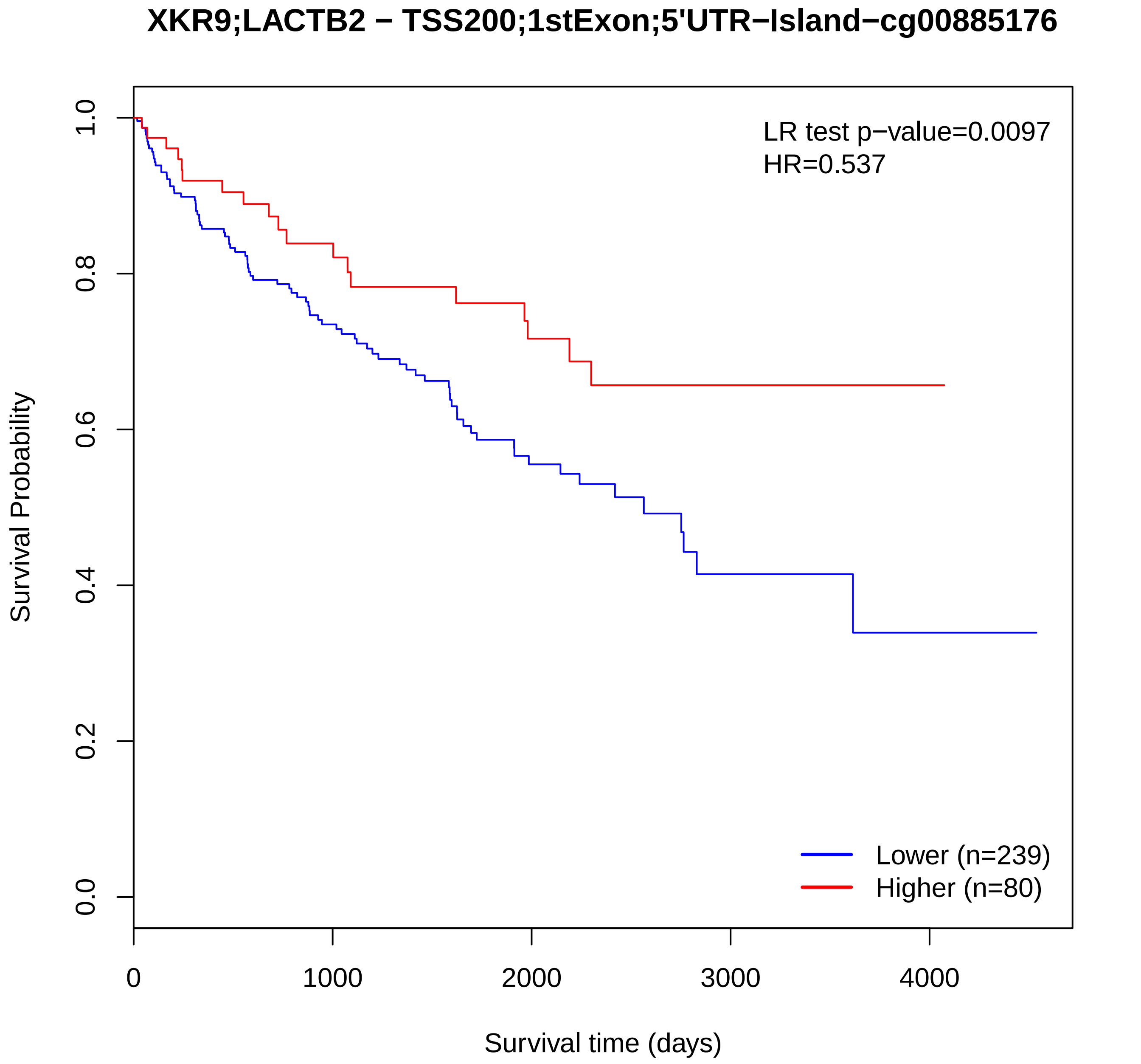

XKR9;LACTB2 − TSS200;1stExon;5'UTR−Island−cg00885176
LR test p−value=0.0097
HR=0.537
Survival Probability
0.0
0.2
0.4
0.6
0.8
1.0
0
1000
2000
3000
4000
Survival time (days)
Lower (n=239)
Higher (n=80)

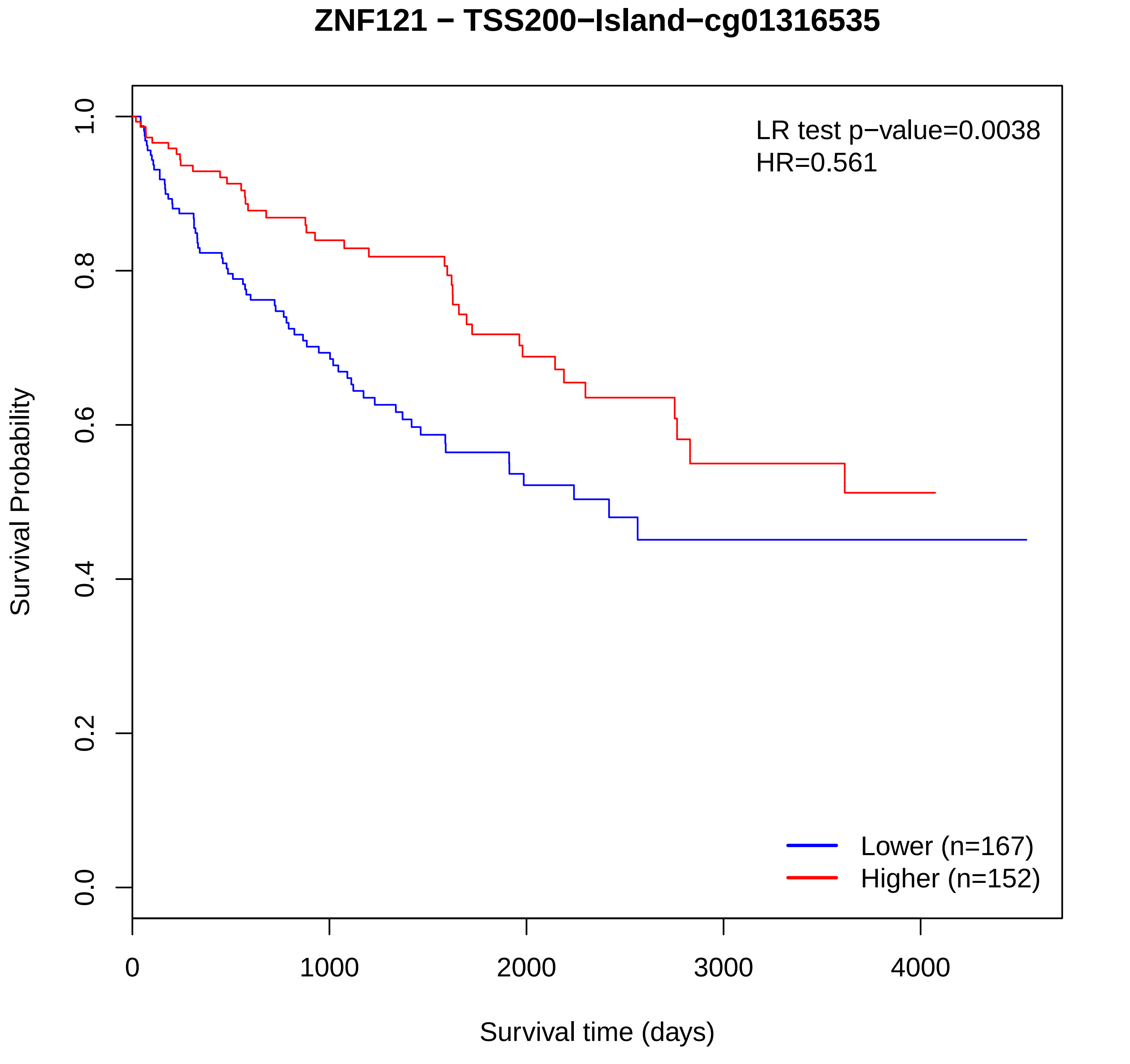
ZNF121 − TSS200−Island−cg01316535
LR test p−value=0.0038
HR=0.561
Lower (n=167)
Higher (n=152)
Survival Probability
0.0
0.2
0.4
0.6
0.8
1.0
0
1000
2000
3000
4000
Survival time (days)
