# Supplementary material for: Integrative Analysis of DNA Methylation Identified 12 Signature Genes Specific to Metastatic ccRCC
Source: Front Oncol. 2020 Oct 8;10:556018. doi: 10.3389/fonc.2020.556018 (PMC7578385; doi:10.3389/fonc.2020.556018)
Supplement: Supplementary file 21 [file Image_2.pdf]

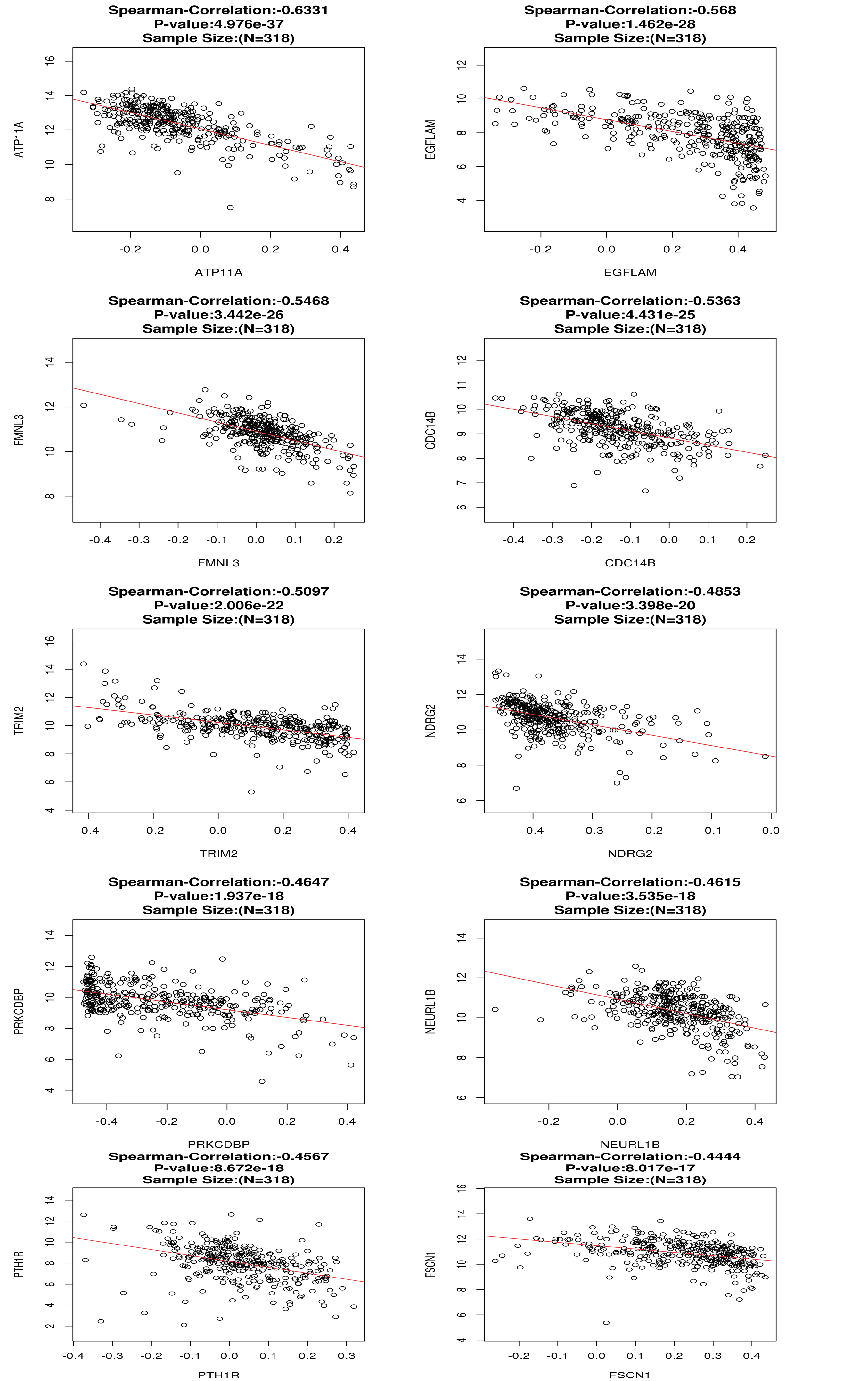

Supplementary figure 2 The overlapped genes between DMGs based on different region CpGs and DEGs with the top 10 correlation coefficients. Spearman's correlation analysis was performed between methylation (horizontal axis) and expression (vertical axis) of intersected genes. Spearman's correlation coefficient and Pvalues are shown in each plot.(Data was analyzed in the TCGA database.)
