# Supplementary material for: Integrative Analysis of DNA Methylation Identified 12 Signature Genes Specific to Metastatic ccRCC
Source: Front Oncol. 2020 Oct 8;10:556018. doi: 10.3389/fonc.2020.556018 (PMC7578385; doi:10.3389/fonc.2020.556018)
Supplement: Supplementary file 22 [file Image_3.pdf]

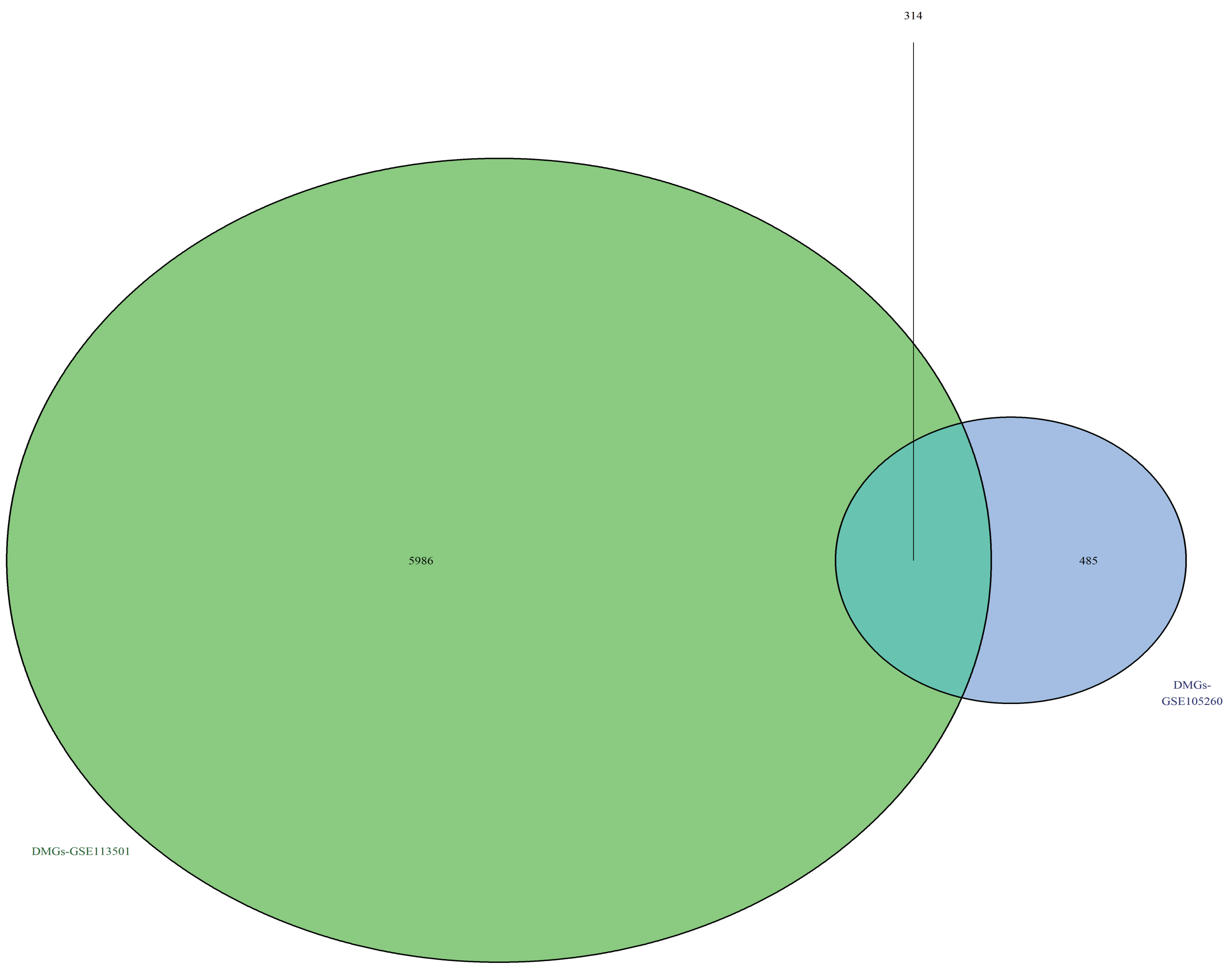

Supplementary figure 3 Venn diagram of overlapping genes among the DMGs based on all regions from GEOdataset GSE105260 and GEOdataset GSE113501.
