# Supplementary material for: Integrative Analysis of DNA Methylation Identified 12 Signature Genes Specific to Metastatic ccRCC
Source: Front Oncol. 2020 Oct 8;10:556018. doi: 10.3389/fonc.2020.556018 (PMC7578385; doi:10.3389/fonc.2020.556018)
Supplement: Supplementary file 23 [file Image_4.pdf]

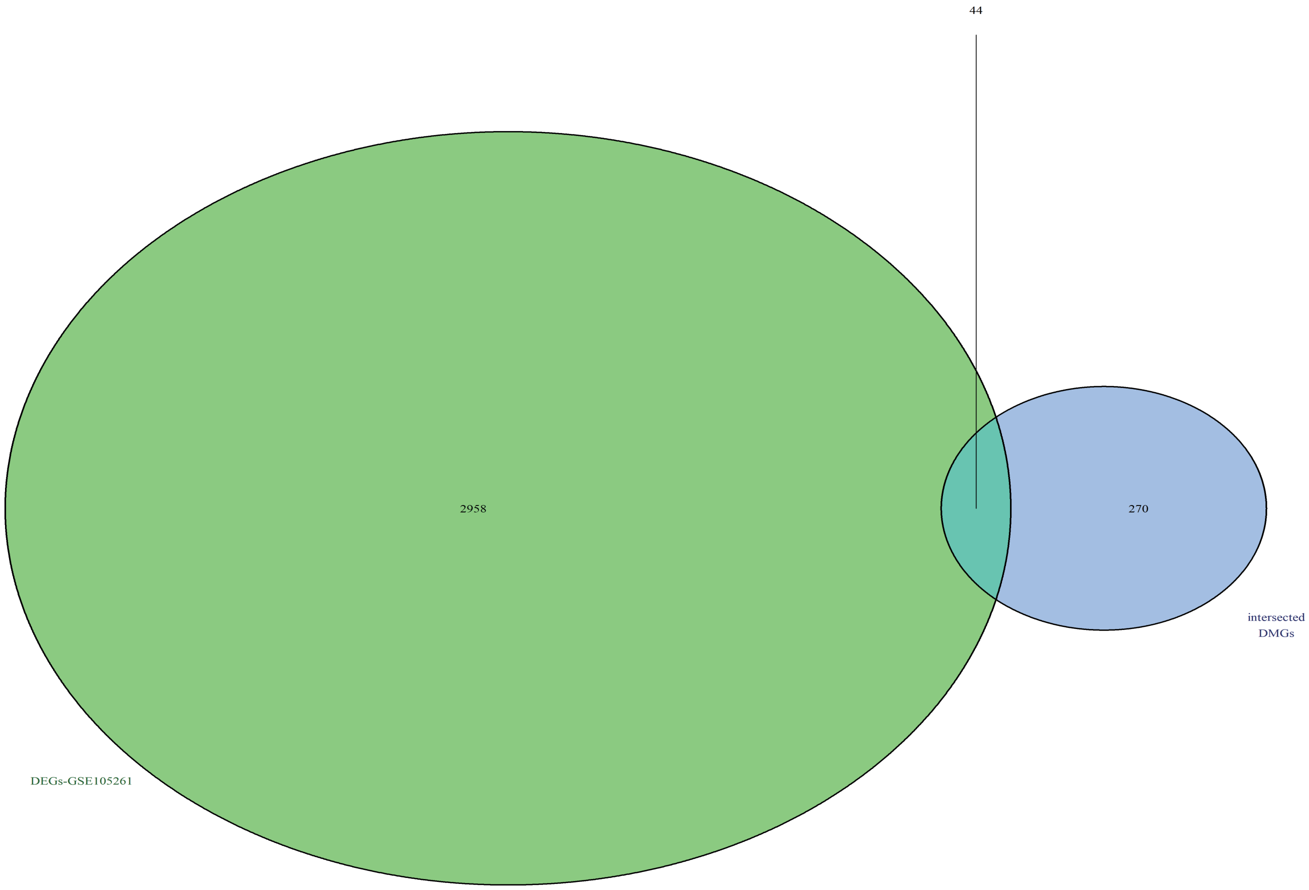

Supplementary figure 4 Venn diagram of overlapping genes among the intersected DMGs based on all regions and DEGs screened from GEOdataset GSE105261.
