# Supplementary material for: Integrative Analysis of DNA Methylation Identified 12 Signature Genes Specific to Metastatic ccRCC
Source: Front Oncol. 2020 Oct 8;10:556018. doi: 10.3389/fonc.2020.556018 (PMC7578385; doi:10.3389/fonc.2020.556018)
Supplement: Supplementary file 24 [file Image_5.pdf]

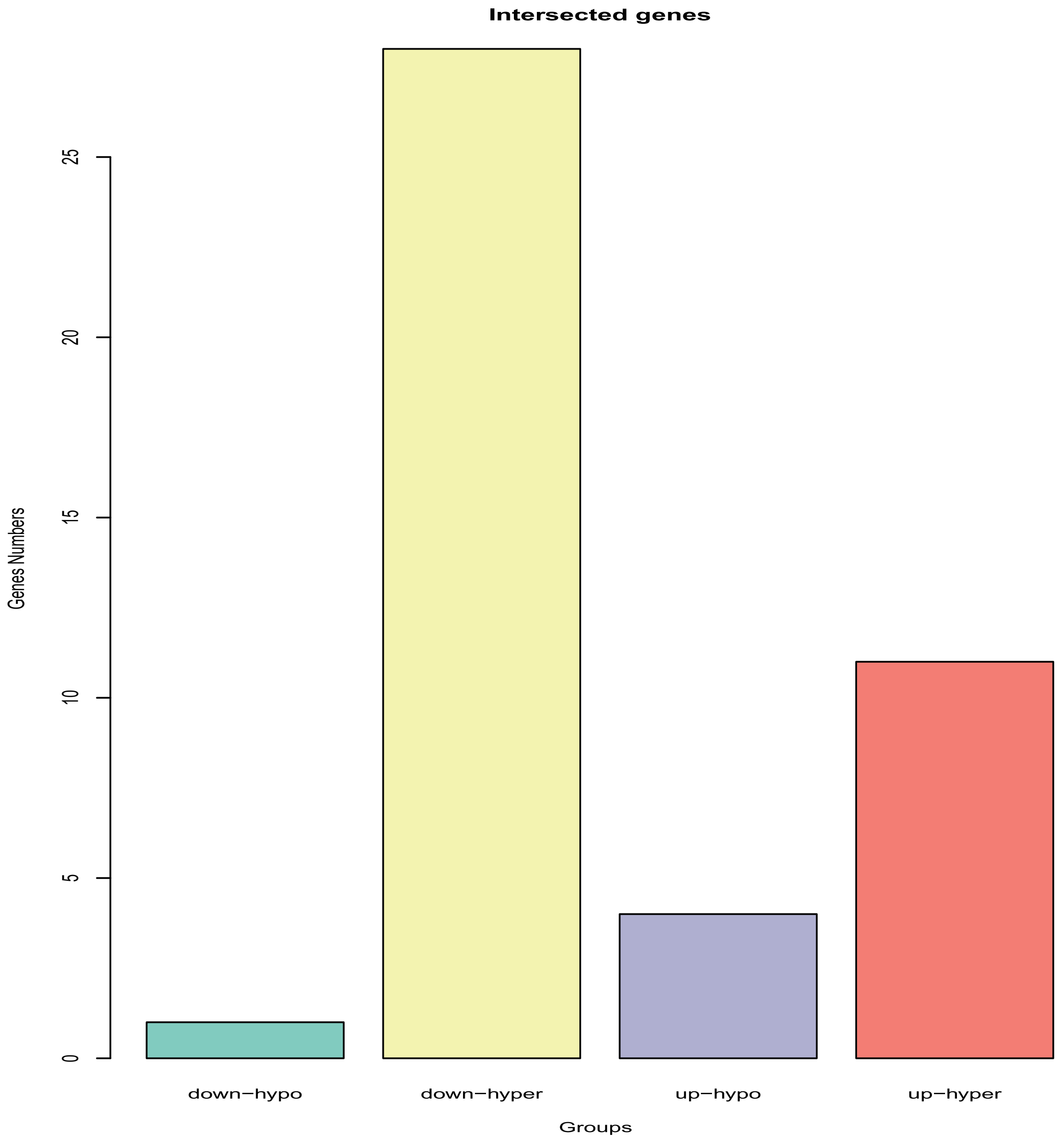

Supplementary figure 5 Characteristics of intersected genes between DEGs from GSE105261 and DMGs based on all region CpGs from GSE105260. **a** Barplot for four groups of intersected genes. Down-hyper represents downregulated and hyper-methylated genes. Up-hypo represents upregulated and hypo-methylated genes. Up-hyper represents upregulated and hyper-methylated genes. Down-hypo represents downregulated hypo-methylated genes. The y-axis is the number of genes.
