# Supplementary material for: Integrative Analysis of DNA Methylation Identified 12 Signature Genes Specific to Metastatic ccRCC
Source: Front Oncol. 2020 Oct 8;10:556018. doi: 10.3389/fonc.2020.556018 (PMC7578385; doi:10.3389/fonc.2020.556018)
Supplement: Supplementary file 25 [file Image_6.pdf]

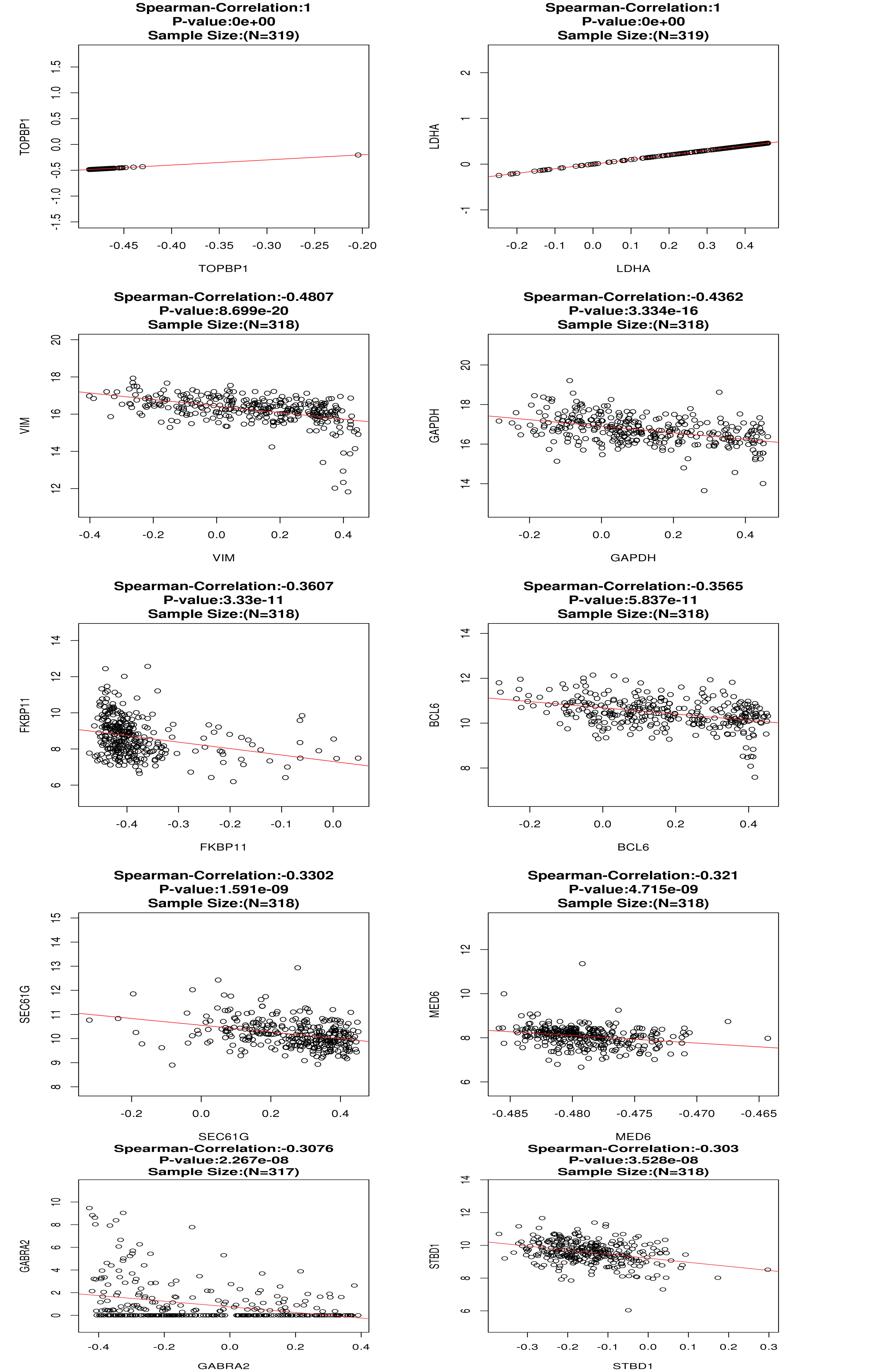

Supplementary figure 6 The overlapped genes between DMGs based on all region CpGs from GSE105260 and DEGs from GSE105261 with the top 10 correlation coefficients. Spearman's correlation analysis was performed between methylation (horizontal axis) and expression (vertical axis) of intersected genes. Spearman's correlation coefficient and Pvalues are shown in each plot.(Data was analyzed in the TCGA database.)
